# In Silico Mining and Characterization of High-Quality SNP/Indels in Some Agro-Economically Important Species Belonging to the Family Euphorbiaceae

**DOI:** 10.3390/genes14020332

**Published:** 2023-01-27

**Authors:** Surojit Sen, Sunayana Rathi, Jagajjit Sahu, Subhash C. Mandal, Supratim Ray, Petr Slama, Shubhadeep Roychoudhury

**Affiliations:** 1 Department of Zoology, Mariani College, Mariani 785634, India; 2 Department of Biochemistry and Agricultural Chemistry, Assam Agricultural University, Jorhat 785013, India; 3 GyanArras Academy, Gothapatna, Malipada, Bhubaneswar 751003, India; 4 Department of Pharmaceutical Technology, Division of Pharmacognosy, Jadavpur University, Kolkata 700032, India; 5 Department of Pharmaceutical Sciences, Assam University, Silchar 788011, India; 6 Laboratory of Animal Immunology and Biotechnology, Department of Animal Morphology, Physiology and Genetics, Faculty of AgriSciences, Mendel University in Brno, 61300 Brno, Czech Republic; 7 Department of Life Science and Bioinformatics, Assam University, Silchar 788011, India

**Keywords:** EST, potential SNP, nucleotide substitution, C↔T transition, A↔T transversion, indel

## Abstract

(1) Background: To assess the genetic makeup among the agro-economically important members of Euphorbiaceae, the present study was conducted to identify and characterize high-quality single-nucleotide polymorphism (SNP) markers and their comparative distribution in exonic and intronic regions from the publicly available expressed sequence tags (ESTs). (2) Methods: Quality sequences obtained after pre-processing by an EG assembler were assembled into contigs using the CAP3 program at 95% identity; the mining of SNP was performed by QualitySNP; GENSCAN (standalone) was used for detecting the distribution of SNPs in the exonic and intronic regions. (3) Results: A total of 25,432 potential SNPs (pSNP) and 14,351 high-quality SNPs (qSNP), including 2276 indels, were detected from 260,479 EST sequences. The ratio of quality SNP to potential SNP ranged from 0.22 to 0.75. A higher frequency of transitions and transversions was observed more in the exonic than the intronic region, while indels were present more in the intronic region. C↔T (transition) was the most dominant nucleotide substitution, while in transversion, A↔T was the dominant nucleotide substitution, and in indel, A/- was dominant. (4) Conclusions: Detected SNP markers may be useful for linkage mapping; marker-assisted breeding; studying genetic diversity; mapping important phenotypic traits, such as adaptation or oil production; or disease resistance by targeting and screening mutations in important genes.

## 1. Introduction

Euphorbiaceae is a diverse group of flowering plants distributed worldwide, particularly in tropical and subtropical regions. The family is well-known for both its medicinal and commercial relevance due to the presence of a large variety of unique secondary metabolites. For instance, *Euphorbia tirucalli* L. is well-known for its healing properties against disorders (such as arthritis, asthma, warts, toothache, gonorrhoea, cough, earache, neuralgia, rheumatism, cancer, and tumors [1,2,3]) as well as for biodiesel production [2]. *E. lathyris* L., *Jatropha curcas* L., *Ricinus communis* L., and *Vernicia fordii* Hemsl. are used in the production of paints, varnishes, polymers, and tung oil [3,4,5,6]. Some members of Euphorbiaceae are sources of food, e.g., *Manihot esculenta* Crantz. [7], while some others also have agro-economic importance, for example, as a source of rubber (*Hevea brasiliensis* Müll.Arg.), medicine (*E tirucalli* L.), fruits (*Phyllanthus emblica* L.), and insecticides (*E. tirucalli* L., *Sebastiania corniculata* (Vahl) Muell. Arg.) [8].

The family is also an important source of traditional medicine to cure liver disease, sprains, snake bites, convulsion, asthma, tumors, and rheumatism, as documented in the Indian traditional system of medicine—*Ayurveda* [9]. Other medicinal products include castor oil and sangre de grado (*Croton* spp.). Ethnobotanical leads from a study in Samoa led to the development of the anti-AIDS (acquired immune deficiency syndrome, i.e., AIDS) drug prostratin from *Homalanthus nutans* [10]. Due to its perennial nature and long growing cycles, conventional breeding in Euphorbiaceae is a slow and time-consuming process; therefore, marker-assisted breeding is preferable. Compared to morphological or chemical markers, molecular markers relying on DNA sequencing and polymerase chain reaction (PCR) are a more robust, reliable, and time- and cost-effective resource for molecular characterization. With the advancement of molecular biology techniques, crop breeders have been able to screen huge populations of plants for desired characteristics by using tools like molecular markers, thus adding value to their study of comparative mapping and crop improvement by marker-assisted breeding. Due to a lack of efficient molecular markers, however, little is known about the population genetic diversity and genetic relationships among members of the Euphorbiaceae family. Efficient and robust molecular markers are increasingly needed for the breeding and improvement of varieties in the family. Marker development using the conventional genetic library-dependent method is a lengthy and expensive process. Large amounts of publicly accessible DNA sequence information have been produced since the emergence of modern genomics.

In particular, expressed sequence tags (ESTs) provide valuable resources to develop gene-associated markers. Compared to other DNA markers, such as amplified fragment length polymorphisms (AFLPs) and randomly amplified polymorphic DNAs (RAPDs), EST-derived simple sequence repeat (SSR) markers are advantageous because they show the highest level of polymorphism and are co-dominant. They are, therefore, more informative and more conserved and are potentially transferable between genera [11,12,13,14]. SNPs demonstrate single-base allelic variation between any homologous pair of chromosomes or between two haplotype sequences. They are widely distributed in the genomic DNA of eukaryotic cells (both in coding and non-coding regions) [15]. Although the bi-allelic nature of SNP is a disadvantage compared to multi-allelic highly polymorphic microsatellites, the usefulness of SNPs as genetic markers in eukaryotic genomes has been well established. Recently, attention has been geared towards the use of SNPs as genetic markers [16].

The traditional method of SNP identification is done by sequencing the selected DNA fragment and comparing it with a reference genome in cases wherein whole-genome sequences are available. But this method of SNP identification has limitations like a high rate of sequencing error, a high cost, and the limited availability of complete genome sequences. Therefore, the growing availability of ESTs represents an alternative in silico approach for de novo SNP discovery in species whose whole-genome sequences are not presently available. Moreover, because EST-derived SNPs are found in both coding and 3′-UTR regions, they are regarded as gene-derived SNPs [17].

EST sequences are frequently employed in quick and affordable ways to find new genes and as a resource for cDNA array construction and gene mapping [18]. Many bioinformatics tools/programs/pipelines are available for mining SNP, each adopting various strategies, algorithms, filtration, evaluation methods, and input/output formats. Each strategy has advantages and disadvantages of its own. Some of the available programs/pipelines for SNP detection are Polyphred [19], PolyBayes [20], SEAN [21], TRACE_DIFF [22], HarvEST [23], AutoSNP [24], QualitySNP [25], HaploSNPer [26], and QualitySNPng [27]. Among these, QualitySNPng is the most recent and advanced, but it can handle only next-generation sequencing data as input. On the contrary, QualitySNP pipeline has a triple filtering system to eliminate unreliable variation and sequencing errors when the sequenced reference genome is not available, and it can handle EST data with or without quality files. A haplotype-based strategy adopted in QualitySNP makes full use of the redundancy in sequences by reclustering them, minimizes the sequencing error, and removes the sequences with poor quality. The QualitySNP pipeline has been successfully used for mining out reliable SNPs in various crop plants [28], zebra finch [29], waterfleas [30], snakes [31], scallops [32], and potato [25]. Quality SNPs have successfully been detected in diploid as well as polyploid species. Therefore, in the present study, an attempt was made to identify and characterize high-quality (intra-specific) SNP markers from the ESTs of nine species, viz., *E. esula* L. (leafy spurge), *E. fischeriana* Steud. (spurge), *E. lagascae* Spreng. (spurge)*, E. tirucalli* L. (milk bush), *H. brasiliensis* Müll.Arg. (rubber tree), *J. curcas* L. (Barbados nut), *M. esculenta* Crantz. (cassava), *R. communis* L. (castor oil plant), and *V. fordii* Hemsl. (airy shaw)***,*** available at dbEST of NCBI. These species are of high agro-economic importance but lack robust molecular markers for breeding. The distribution of nucleotide substitution and the comparative distribution of these SNP markers in the exonic and intronic areas were both investigated. The proper analysis of SNP markers in EST sequences will provide a better insight into the genetic architecture and will correlate the genes with their functions, as well as with the physiological process involved. The revealed SNP markers may also be employed for genome-wide association studies, QTL mapping, and marker-assisted breeding programs for improving varieties with desired traits.

## 2. Materials and Methods

### 2.1. Data Retrieval

A total of 291,693 EST sequences of nine selected species of the Euphorbiaceae family was retrieved from dbEST [33], hosted by GeneBank (NCBI), using the keyword “Euphorbiaceae” and saved in individual FASTA format files.

### 2.2. Sequence Pre-Processing and Assembly

Utilizing the online server EGassembler [34], which offers automatic and user-customized analysis tools for cleaning, repeat masking, vector trimming, organelle masking, clustering, and assembling, raw ESTs were pre-processed. EGassembler consists of a pipeline of five components. The TIGR Gene Indices, CAP3, RepeatMasker, Cross Match [35,36,37,38], and a non-redundant database of repeats and vectors spanning nearly all publicly available vector and repeat databases are dependable open-source tools used by the EGassembler’s five pipeline components. All of the processes in the pipeline, except the assembling step, were performed step by step, and the output of each step was used as input in the next step of the pipeline. Sequence cleansing comprises trimming low-quality ends, eliminating the polyA/polyT tail, and rejecting short sequences (less than 100 bases) or sequences that appear to be primarily of low-complexity.

During the process of repeat masking, the query sequence was compared to one or more files of FASTA sequences (library for masking). Vectors and organelles were masked by the program Cross_Match [38]. Finally, using the CAP3 tool [36], high-quality sequences acquired after pre-processing were assembled into contigs with 95% identity.

### 2.3. High-Quality SNPs’ Discovery

The Linux-based command-line program QualitySNP (http://www.bioinformatics.nl/tools/snpweb/, accessed on 4 March 2022) pipeline was used for the extraction of SNPs [25]. Three filters were used by QualitySNP to find reliable SNPs. Filter 1 locates variance between or within genotypes while screening for all possible SNPs. The core filter, Filter 2, employs a haplotype-based approach to find reliable SNPs. Clusters with putative paralogs, as well as spurious SNPs caused by sequencing errors, were identified. By determining a confidence score based on sequence redundancy and quality, Filter 3 filters out high-quality SNPs. Through the re-clustering of ESTs, QualitySNP identified the haplotypes that were present in the contigs. In the current study, nucleotide differences between the identified haplotypes of a contig were extracted. QualitySNP discovered the haplotypes that were present in the contigs by the re-clustering of ESTs. In the present study, nucleotide differences between the identified haplotypes of a contig were identified [25]. The QualitySNP algorithm was used to calculate the percentage of nucleotide discrepancies from the qSNP/pSNP ratios (Supplementary Files S1–S4).

### 2.4. Prioritizing High Quality SNPs

For detecting the distribution of SNPs in the exonic and intronic regions, GENSCAN (genes.mit.edu/GENSCAN.html, accessed on 4 March 2022) was used. A standalone version was downloaded, and executables for the Linux platform were used for locating the exonic and intronic regions among the available contigs with SNPs. All available contigs of nine different species of the Euphorbiaceae family were processed through GENSCAN [39] to locate possible positive or negative open reading frames. Further screening was done manually by comparing the output file of GENSCAN (showing the exonic region) with the output file of QualitySNP (file name SNP quality, showing the position of true high-quality SNP) to learn the distribution of transition, transversion, and indel in the exonic and intronic regions.

## 3. Results

**Figure 1:**
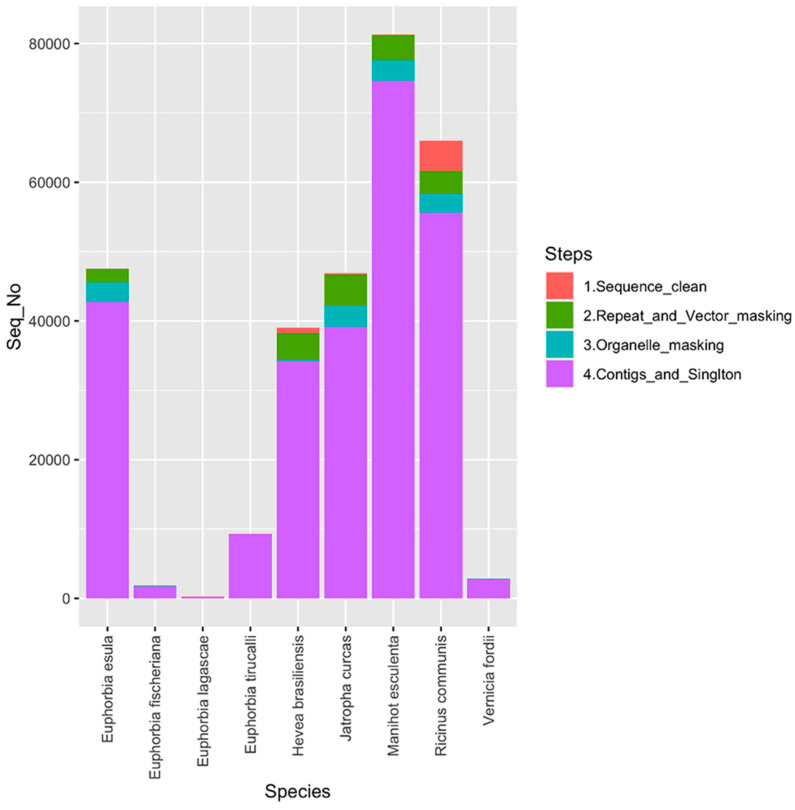
Summary of pre-processing and assembly of expressed sequence tags (ESTs) of nine selected species belonging to the family Euphorbiaceae.

In this study, a total of 291,693 raw EST sequences belonging to nine selected species of Euphorbiaceae family was pre-processed to produce a total of 260,479 cleansed EST sequences. An overview of sequence cleansing (pre-processing) and assembly detail is provided in Figure 1. 

The cleansed sequences were then assembled by Cap3 (similarity score 95) to obtain a total of 34,736 contigs and 77,341 singletons. The obtained contigs of each species were further analyzed individually by QualitySNP for the detection of potential SNP sites and then filtered to find high-quality SNPs. The detected high-quality SNPs were further screened to find their distribution in the exonic and intronic regions.

In this study, a total of 25,432 potential SNPs and 20,753 real SNPs were discovered. The real SNPs were further filtered (i.e., removal of single haplotypes and paralogs), and, finally, based on the confidence score; the numbers were reduced to a total of 14,351 high-quality SNPs, including 2276 indel polymorphisms. The ratio of qSNP to pSNP ranged from 0.22 to 0.75 among the nine species under study. The highest (qSNP/pSNP) ratio was found to be 0.75 in the case of *E. esula*. L. The highest number of SNPs per kbp was found to be 203.47 in the case of *E. tirucalli L.,* and the lowest number of SNPs per kbp was found to be 0.03 in the case of *E. fischeriana* Steud. The ratio of transitions to transversions ranged from 0.59 to 2.57 and was found to be highest in *E. fischeriana* Steud. (2.57). The highest number of indels was found in *R. communis L.* (804), and the maximum indels per kbp was found in *E. tirucalli L.* (35.84). A summary of SNPs detection, indels, and other parameters is depicted in Table 1.

**Figure 2:**
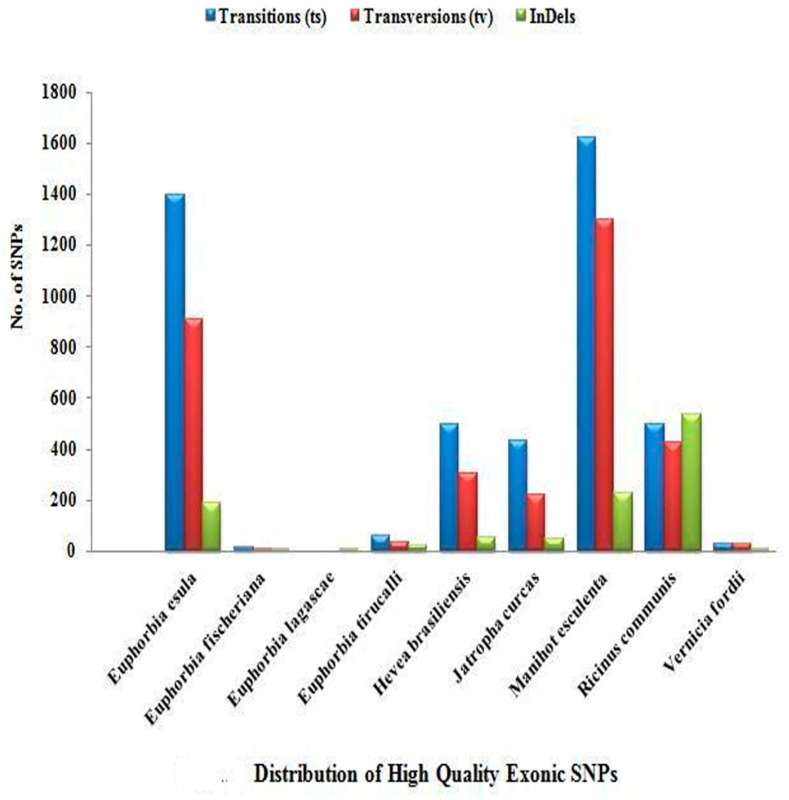
Distribution of high-quality exonic single-nucleotide polymorphisms (SNPs).

**Figure 3:**
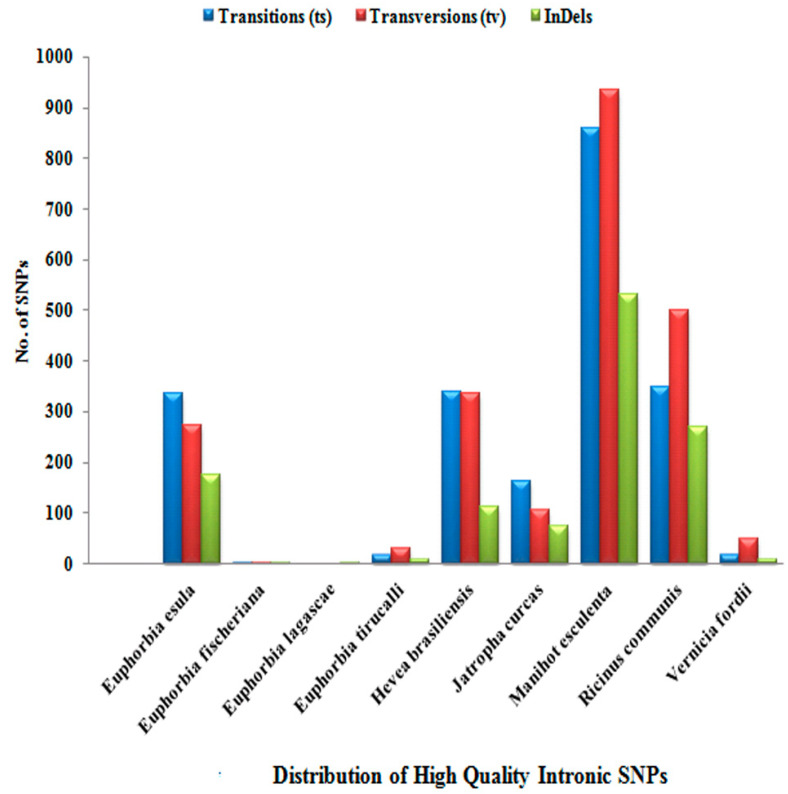
Distribution of high-quality intronic single-nucleotide polymorphisms (SNPs).

A high frequency of transition was observed among all of the members of Euphorbiaceae under study. Out of 14,351 high-quality SNPs, only 8849 high-quality SNPs were found to be located in the exonic/orf region, while 5502 high-quality SNPs were present in the intronic region. The prevalence of exonic and intronic SNPs as per their occurrence is detailed in Supplementary Table S1 and Table S2. Figure 2 and Figure 3 show the distribution of transition/transversion, which is higher in the exonic region than the intronic region in all of the species except for *R. communis* L. and *V. fordii* Hemsl., in which transversions are more frequent in the intronic region. The distribution of indels was found to be higher in the intronic region in most of the species, including *H. brasiliensis* Müll. Arg., *J. curcas* L., *M. esculenta* Crantz., *R. communis* L., and *V. fordii* Hemsl.

**Figure 4:**
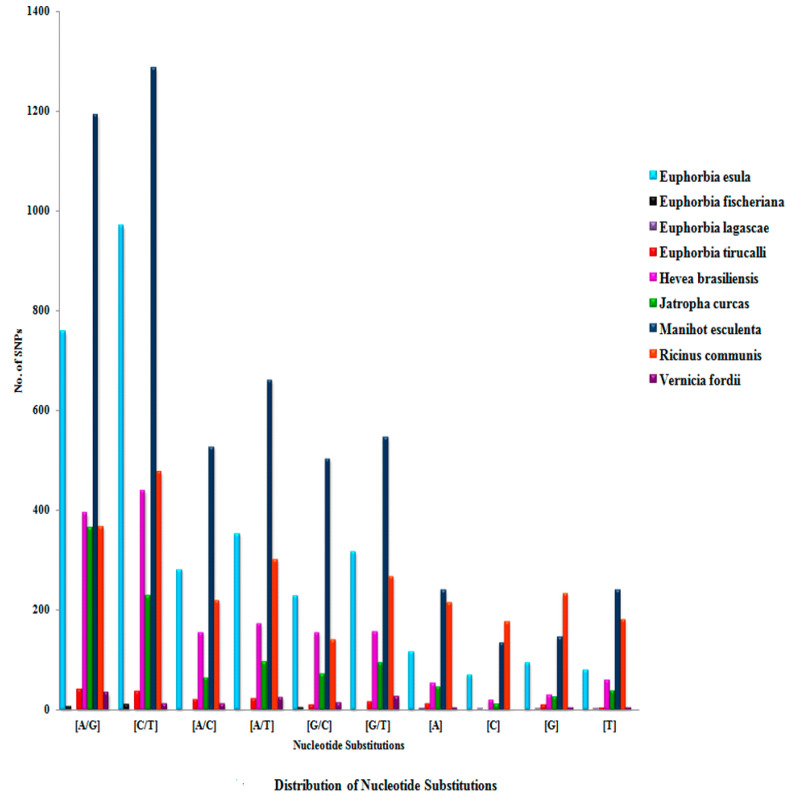
Distribution of nucleotide substitutions in nine selected species belonging to the family Euphorbiaceae.

The details of nucleotide substitutions of all of the nine selected species belonging to the family Euphorbiaceae are shown in Supplementary Table S3 and Figure 4. The frequency of C↔T mutation (transition) was observed to be higher in all members of Euphorbiaceae, while in the case of transversion, the A↔T ratio was found to be most abundant, and among the indels, (A/−, −/A) was found to be more abundant.

## 4. Discussion

Since SNPs are present throughout the genome, both in the coding and non-coding regions, even though they are less informative than microsatellites due to their bi-allelic nature, they are still regarded as a highly reliable and valuable molecular marker system for genotyping and selective breeding. Low genetic variation is a key feature of many agro-economically important families like Euphorbiaceae [40,41,42,43]. SNPs are efficiently used for assessing population genetic structure because heterozygosity can be easily measured for their binary, co-dominant nature, and once a rare SNP is detected, population discrimination can be found even in low-diversity species. 

In this study, a total of 25,432 potential SNPs, and 14,351 high-quality SNPs, including 2276 indels, were detected from 26,0479 EST sequences of nine different species belonging to the family Euphorbiaceae. Maximum number of high-quality SNPs was found in *M. esculenta* (5475), followed by *E. esula* L. (3270), *R. communis* (2574), *H. brasiliensis* (1635), *J. curcas* (1043), *E. tirucalli* (176), *V. fordii* (140), and *E. fischeriana* (31), and the lowest number was detected in *E. lagascae* (7), as shown in Supplementary Table S2. Compared to the results of these nine species belonging to the family Euphorbiaceae, a total of 37,344 SNPs were detected in Arabidopsis [44], and a total of 31,815 potential SNPs; 16,772 high-quality SNPs; and 1815 indels were found from 83,565 EST sequences in potato [25].

SNP occurrence was found to be 1 per 277 bp, 481 bp, 124 bp, 534 bp, 277 bp, 438 bp, 312 bp, 445 bp, and 337 bp in *E. fischeriana*, *E. lagascae*, *E. tirucalli*, *H. brasiliensis*, *J. curcas*, *M. esculenta*, *R. communis*, and *V. fordii*, respectively (Supplementary Table S2). In fact, this is higher in comparison to apple ESTs [45], i.e., 1 in every 706 bp and in ginger, i.e., 1 in every 619 bp [46]. 

SNP frequency was found to be higher, i.e., one SNP in every 3.61 kb, 2.08 kb, 8.09 kb, 1.87 kb, 3.62 kb, 2.28 kb, 3.21 kb, 2.24 kb, and 2.96 kb in *E. esula* L., *E. fischeriana* Steud., *E. lagascae* Spreng., *E. tirucalli* L., *H. brasiliensis* Müll.Arg., *J. curcas* L., *M. esculenta* Crantz., *R. communis* L., and *V. fordii* Hemsl., respectively (Supplementary Table S2). Similar results were also found in *Arabidopsis thaliana* ecotype: Landsberg erecta (1 SNP in every 3.3 kb) and in ecotype Columbia (1 SNP in every 6.1 kb) [47]. Pootakham et al. studied SNP identification in *H. brasiliensis* Müll.Arg. and found that the average SNP frequency was 1 SNP in every 1.5 kb [48], while the present study showed much lower frequency (1 SNP in every 3.62 kb). They observed the transition:transversion ratio to be 1.67, while, in this study, the ratio was found to be 1.30. In the current study, it was shown that all the selected members of the Euphorbiaceae family had an excess of transitions on average, which is in accordance with earlier studies on SNP discovery in maize [49], as well as ginger [46]. This can be attributed to the abundant hyper-mutable methylated di-nucleotide 5′-CpG-3′ [43]. One probable explanation of this would be the high spontaneous rate of the deamination of 5′-methylated cytosines (5mC) at CpG di-nucleotides to thymidine (C↔T) SNPs and (G↔A) on the complementary strand [50]. Higher A↔T mutation was observed among all the species under study, which is similar to the results in ginger [46], and the reason for this still remains unclear.

This study provides information about potential SNPs (25,432) and selects 14,351 high-quality SNPs, including 2276 indels from the EST datasets of nine agro-economically important species belonging to the family Euphorbiaceae. It also provides insight in the comparative distribution of high-quality SNPs in the exonic and intronic regions, as well as a comparison of nucleotide substitution among the members of Euphorbiaceae. The triple filtration strategy adopted in QualitySNP makes it a more stringent and efficient tool for SNP detection from EST. Although QualitySNP generated a reduced number of high-quality SNPs, the reliability of the results obtained is more than other software tools like PolyBayes, which needs quality files, or Pearl script AutoSNP. A study on SNP mining from EST in sea bass using different tools justifies the reliability of SNP discovery by QualitySNP over other tools [51]. Results obtained using QualitySNP in the case of ginger EST was more efficient compared to the results obtained from the same EST dataset in ginger by AutoSNP [46]. The reliability of the SNPs produced by QualitySNP was also confirmed in the potato EST dataset, and the results outperformed the results of AutoSNP [25]. In addition to predicting reliable SNPs, QualitySNP’s haplotype-based approach also identifies haplotypes and can be applied to EST-based genotyping. SNPs discovered in this study can further be validated and used for future research as a tool for genome mapping, map-based positional cloning, QTL detection, and the assessment of genetic relationships.

## 5. Conclusions

The mining of ESTs revealed 25,432 potential SNPs and 14,351 high-quality SNPs, which includes 2276 indels from a total of 260,479 EST sequences from the nine agro-economically important species of Euphorbiaceae under study. SNP frequency was found to be high among all of the members, with an average of 3.33 SNPs/1000 bp. A high frequency of transition was observed among all of the members. The comparative distribution of SNPs (transition, transversion, and indel) showed that both transition and transversion were present more in the exonic region than the intronic region, while indels were present more in the intronic region compared to the exonic region. C↔T (transition) is the most dominant nucleotide substitution among all the members, while in transversion, A↔T is the dominant nucleotide substitution, and in indel, A/− is dominant. The SNP markers detected may be useful for linkage mapping and marker-assisted breeding programs, as well as for studying genetic diversity among the members of Euphorbiaceae. They may also be used for mapping important phenotypic traits, such as adaptation, oil production, or disease resistance, by targeting and screening mutations in important genes.

## Figures and Tables

**Table 1:** Summary of single-nucleotide polymorphism (SNP) detection, indels, and other parameters identified from nine selected species belonging to the family Euphorbiaceae.

	*E. esula*	*E. fischeriana*	*E. lagascae*	*E. tirucalli*	*H. brasiliensis*	*J. curcas*	*M. esculenta*	*R. communis*	*V. fordii*
Total ESTs	42,751	1766	233	9284	34,212	39,175	74,596	55,636	2826
Total Contigs	8865	206	14	1144	3579	5060	10448	5209	211
Singletons	17,593	886	186	5474	7961	14,658	21,133	8442	1008
Total Available Contings	1719	38	2	260	1367	1489	4037	2413	99
Total ESTs in Available Contigs	8802	503	22	1825	16,751	15,656	38,618	35,977	1561
No. of Contigs with Potential SNP	935	15	2	117	434	462	1925	946	45
No. of ESTs in Contigs Containing Potential SNP	5274	377	22	1079	10,500	9368	26,819	26,616	1160
Total Consensus Size (bp)	906,024	14,897	865	94,059	452,159	456,724	1,706,808	1,146,581	47,218
Average Contig Length	969	993	432.5	804	1042	989	884	1029	1049
Average ESTs per Contig	5.2	24.1	9	7.6	20.14	17.25	12.09	24.41	22.76
Maximum Contig Size (bp)	2086	1728	434	1767	3107	2840	2751	3102	2052
Minimum Contig Size (bp)	301	500	431	385	183	192	167	185	484
Contings with 2 ESTs	5082	127	11	667	1548	2617	4388	1919	79
Contings with > 2 ESTs	3783	79	3	477	2031	2442	6060	3290	129
Potential SNPs (pSNP)	4377	53	18	421	2296	2620	8560	6449	638
True High-Quality SNPs (qSNP)	3270	31	7	176	1635	1043	5475	2574	140
Haplotypes	2309	36	11	364	1097	1459	5806	3427	215
Removed single haplotypes	386	5	4	111	102	373	1054	810	73
Ratio of qSNP to pSNP	0.75	0.58	0.39	0.42	0.71	0.4	0.64	0.4	0.22
No. of SNPs per kbp	3.61	2.08	8.09	1.87	3.62	2.28	3.21	2.24	2.96
No. of SNPs per Contig	3.5	2.1	3.5	1.5	3.8	2.3	2.8	2.7	3.1
No. of bp per SNP	277	481	124	534	277	438	312	445	337
Transitions (ts)	1728	18	0	77	833	592	2479	843	46
Transversions (tv)	1179	7	0	68	638	326	2236	927	78
Ratio of Transitions to Transversion	1.47	2.57	0	1.13	1.3	1.82	1.11	0.91	0.59
InDels	363	6	7	31	164	125	760	804	16
InDels per kbp	0.04	0.4	8	0.33	0.36	0.27	0.44	0.7	0.33
InDels per Contig	0.21	0.16	3.5	0.12	0.12	0.08	0.19	0.33	0.16
Base pairs per InDels	2496	2128	124	3034	2757	3654	2246	1426	2951

## Data Availability

Not applicable.

## Supplementary Materials

The following supporting information can be downloaded at: https://www.mdpi.com/article/10.3390/genes14020332/s1. Table S1. Distribution of high-quality exonic single-nucleotide polymorphisms (SNPs). Table S2. Distribution of high-quality intronic single-nucleotide polymorphisms (SNPs). Table S3. Distribution of nucleotide substitutions in nine selected species belonging to the family Euphorbiaceae. File S1. named “realsnpinfo” includes all SNPs in the clusters, excluding those from single haplotypes. File S2. named “SNPquality” provides all relevant information for identifying reliable SNPs, such as confidence scores and allele haplotype scores, as well as the location of SNPs. File S3. BLASTx results of contigs containing SNPs. File S4. named “allavailsnp” contains 25 nucleotides senses and a reverse string surrounding the SNP; the SNP is at the middle position (residue 13). These can be used for designing probes.

## References

[1] Cataluna P., Rates S.M.K., Martino V., Caffini N., Lappa A., Ferraro G., Schilder H. (1999). The traditional use of the latex from *Euphorbia tirucalli* Linnaeus (Euphorbiaceae) in the treatment of cancer in South Brazil. Proc. WOCMAP-2 Pharmacognosy, Pharmacology, Phytomedicines, Toxicology.

[2] Van Damme P.L.J., Schlissel A., Pasternak D. (2001). Euphorbia tirucalli for high biomass production. Combating Desertification with Plants.

[3] Duke J.A., Handbook of Energy Crops (1983). Purdue University Centre for New Crops and Plant Products. https://www.hort.purdue.edu/newcrop/duke_energy/dukeindex.html.

[4] De Oliveira J.S., Leite P.M., de Souza L.B., Mello V.M., Silva E.C., Rubim J.C., Meneghetti S.M.P., Suarez P.A.Z. (2009). Characteristics and composition of *Jatropha gossypiifolia* and *Jatropha curcas* L. oils and application for biodiesel production. Biomass Bioenergy.

[5] Meneghetti S.A.P., Meneghetti M.R., Serra T.A., Barbosa D.C., Wolf C.R. (2007). Biodiesel production from vegetable oil mixtures: Cottonseed, soybean, and castor oils. Energy Fuels.

[6] Bhuyan S., Sundararajan S., Andjelkovic D., Larock R. (2010). Effect of crosslinking on tribological behavior of tung oil-based polymers. Tribol. Int..

[7] Aloys N., Ming Z.H. (2006). Traditional cassava foods in Burundi–A review. Food Rev. Int..

[8] Lee C.H., Jeon J.H., Lee S.G., Lee H.S. (2010). Insecticidal Properties of Euphorbiaceae: *Sebastiania corniculata*-derived 8-Hydroxyquinoline and its Derivatives against Three Planthopper Species (Hemiptera: Delphacidae). J. Korean Soc. Appl. Biol. Chem..

[9] Kapoor L.D. (1989). Handbook of Ayurvedic Medicinal Plants: Herbal Reference Library.

[10] Johnson H.E., Banack S.A., Cox P.A. (2008). Variability in content of the anti-AIDS drug candidate prostratin in Samoan populations of *Homalanthus nutans*. J. Nat. Prod..

[11] Cordeiro G.M., Casu R., Mclntyre C.L., Manners J.M., Henry R.J. (2001). Microsatellite markers from sugarcane (*Saccharum* spp.) ESTs cross transferable to erianthus and sorghum. Plant Sci..

[12] Kantety R.V., La Rota M., Matthews D.E., Sorrells M.E. (2002). Data mining for simple sequence repeats in expressed sequence tags from barley, maize, rice, sorghum and wheat. Plant Mol. Biol..

[13] Thiel T., Michalek W., Varshney R.K., Graner A. (2003). Exploiting EST databases for the development and characterization of gene-derived SSR-markers in barley (*Hordeum vulgare* L.). Theor. Appl. Genet..

[14] Feng S.P., Li W.G., Huang H.S., Wang J.Y., Wu Y.T. (2009). Development, characterization and cross-species/genera transferability of EST-SSR markers for rubber tree (*Hevea brasiliensis*). Mol. Breed..

[15] Brookes A.J. (1999). The essence of SNPs. Gene.

[16] Rafalski A. (2002). Applications of single nucleotide polymorphisms in crop genetics. Curr. Opin. Plant Biol..

[17] Jalving R., van’t Slot R., van Oost B.A. (2004). Chicken single nucleotide polymorphism identification and selection for genetic mapping. Poult. Sci..

[18] Parkinson J., Blaxter M., Melville S.E. (2004). Expressed sequence tags: Analysis and annotation. Methods in Molecular Biology Parasite Genomics Protocols.

[19] Nickerson D.A., Tobe V.O., Taylor S.L. (1997). PolyPhred: Automating the detection and genotyping of single nucleotide substitutions using fluorescence-based resequencing. Nucleic Acids Res..

[20] Marth G.T., Korf I., Yandell M.D., Yeh R.T., Gu Z., Zakeri H., Stitziel N.O., Hillier L., Kwok P.Y., Gish W.R. (1999). A general approach to single-nucleotide polymorphism discovery. Nat. Genet..

[21] Huntley D., Baldo A., Johri S., Sergot M. (2006). SEAN: SNP prediction and display program utilizing EST sequence clusters. Bioinformatics.

[22] Bonfield J.K., Rada C., Staden R. (1998). Automated detection of point mutations using fluorescent sequence trace subtraction. Nucleic Acids Res..

[23] Close T.J., Wanamaker S., Roose M.L., Lyon M. (2007). HarvEST. Methods Mol. Biol..

[24] Barker G., Batley J., O’Sullivan H., Edwards K.J., Edwards D. (2003). Redundancy based detection of sequence polymorphisms in expressed sequence tag data using AutoSNP. Bioinformatics.

[25] Tang J., Vosman B., Voorrips R.E., van der Linden C.G., Leunissen J.A. (2006). QualitySNP: A pipeline for detecting single nucleotide polymorphisms and insertions/deletions in EST data from diploid and polyploid species. BMC Bioinform..

[26] Tang J., Leunissen J.A., Voorrips R.E., van der Linden C.G., Vosman B. (2008). HaploSNPer: A web-based allele and SNP detection tool. BMC Genet..

[27] Nijveen H., van Kaauwen M., Esselink D.G., Hoegen B., Vosman B. (2013). QualitySNPng: A user-friendly SNP detection and visualization tool. Nucleic Acids Res..

[28] Anithakumari A.M., Tang J., van Eck H.J., Visser R.G., Leunissen J.A., Vosman B., van der Linden C.G. (2010). A pipeline for high throughput detection and mapping of SNPs from EST databases. Mol. Breed..

[29] Stapley J., Birkhead T.R., Burke T., Slate J. (2008). A linkage map of the zebra finch *Taeniopygia guttata* provides new insights into avian genome evolution. Genetics.

[30] Orsini L., Jansen M., Souche E.L., Geldof S., De Meester L. (2011). Single nucleotide polymorphism discovery from expressed sequence tags in the waterflea *Daphnia magna*. BMC Genom..

[31] Cardoso K.C., Da Silva M.J., Costa G.G., Torres T.T., Del Bem L.E., Vidal R.O., Menossi M., Hyslop S. (2010). A transcriptomic analysis of gene expression in the venom gland of the snake *Bothrops alternatus* (urutu). BMC Genom..

[32] Hou R., Bao Z., Wang S., Su H., Li Y., Du H., Hu J., Wang S., Hu X. (2011). Transcriptome sequencing and *de novo* analysis for Yesso scallop (*Patinopecten yessoensis*) using 454 GS FLX. PLoS ONE.

[33] http://www.ncbi.nlm.nih.gov/dbEST/index.html.

[34] Masoudi-Nejad A., Tonomura K., Kawashima S., Moriya Y., Suzuki M., Itoh M., Kanehisa M., Endo T., Goto S. (2006). EGassembler: Online bioinformatics service for large-scale processing, clustering and assembling ESTs and genomic DNA fragments. Nucleic Acids Res..

[35] Lee Y., Tsai J., Sunkara S., Karamycheva S., Pertea G., Sultana R., Antonescu V., Chan A., Cheung F., Quackenbush J. (2005). The TIGR Gene Indices: Clustering and assembling EST and known genes and integration with eukaryotic genomes. Nucleic Acids Res..

[36] Huang X., Madan A. (1999). CAP3: A DNA sequence assembly program. Genome Res..

[37] Smit A.F.A., Hubley R., Green P. RepeatMasker Open-3.0. http://www.repeatmasker.org/.

[38] Ewing B., Hillier L., Wendl M.C., Green P. (1998). Base-calling of automated sequencer traces using Phred I: Accuracy assessment. Genome Res..

[39] Burge C., Karlin S. (1997). Prediction of complete gene structures in human genomic DNA. J. Mol. Biol..

[40] Allan G., Williams A., Rabinowicz P.D., Chan A.P., Ravel J., Keim P. (2008). Worldwide genotyping of castor bean germplasm (*Ricinus communis* L.) using AFLPs and SSRs. Genet. Resour. Crop Evol..

[41] Foster J.T., Allan G.J., Chan A.P., Rabinowicz P.D., Ravel J., Jackson P.J., Keim P. (2010). Single nucleotide polymorphisms for assessing genetic diversity in castor bean (*Ricinus communis*). BMC Plant Biol..

[42] Gupta P., Idris A., Mantri S., Asif M.H., Yadav H.K., Roy J.K., Tuli R., Mohanty C.S., Sawant S.V. (2012). Discovery and use of single nucleotide polymorphic (SNP) markers in *Jatropha curcas* L.. Mol. Breed..

[43] Siju S., Ismanizan I., Wickneswari R. (2015). Genetic homogeneity in *Jatropha curcas* L. individuals as revealed by microsatellite markers: Implication to breeding strategies. Braz. J. Bot..

[44] Jander G., Norris S.R., Rounsley S.D., Bush D.F., Levin I.M., Last R.L. (2002). Arabidopsis map-based cloning in the post-genome era. Plant Physiol..

[45] Newcomb R.D., Crowhurst R.N., Gleave A.P., Rikkerink E.H., Allan A.C., Beuning L.L., Bowen J.H., Gera E., Jamieson K.R., Janssen B.J. (2006). Analyses of expressed sequence tags from apple. Plant Physiol..

[46] Gaur M., Das A., Subudhi E. (2015). High quality SNPs/Indels mining and characterization in ginger from ESTs data base. Bioinformation.

[47] https://www.arabidopsis.org.

[48] Pootakham W., Chanprasert J., Jomchai N., Sangsrakru D., Yoocha T., Therawattanasuk K., Tangphatsornruang S. (2011). Single nucleotide polymorphism marker development in the rubber tree, *Hevea brasiliensis* (Euphorbiaceae). Am. J. Bot..

[49] Bhattramakki D., Dolan M., Hanafey M., Wineland R., Vaske D., Register J.C., Tingey S.V., Rafalski A. (2002). Insertion-deletion polymorphisms in 3’ regions of maize genes occur frequently and can be used as highly informative genetic markers. Plant Mol. Biol..

[50] Duncan B.K., Miller J.H. (1980). Mutagenic deamination of cytosine residues in DNA. Nature.

[51] Souche E.L., Hellemans B., Van Houdt J.K.J., Canario A., Klages S., Reinhardt R., Volckaert F.A.M. (2007). Mining for Single Nucleotide Polymorphisms in Expressed Sequence Tags of European Sea Bass. J. Integr. Bioinform..

