# Supplementary material for: In Silico Mining and Characterization of High-Quality SNP/Indels in Some Agro-Economically Important Species Belonging to the Family Euphorbiaceae
Source: Genes (Basel). 2023 Jan 27;14(2):332. doi: 10.3390/genes14020332 (PMC9956114; doi:10.3390/genes14020332)
Supplement: Supplementary file 1 [file genes-14-00332-s001.zip › Supplement Data File S3.docx]

**Supplement. 3: BLASTX results of EST-SSR Contigs**

***Euphorbia esula***

| **Sl. No.** | **ID** | **Function** |
| --- | --- | --- |
| 1 | Contig18 | Chlorophyll A-B binding protein |
| 2 | Contig36 | multiprotein-bridging factor 1a |
| 3 | Contig38 | probable protein phosphatase 2C 60 |
| 4 | Contig40 | protein draper-like |
| 5 | Contig41 | shaggy-related protein kinase eta |
| 6 | Contig46 | glycine-rich RNA-binding protein 2, mitochondrial |
| 7 | Contig48 | isopentenyl-diphosphate Delta-isomerase I |
| 8 | Contig52 | N-terminal acetyltransferase B complex catalytic subunit NAA20 |
| 9 | Contig59 | 40S ribosomal protein SA-like |
| 10 | Contig71 | aquaporin PIP1-3 |
| 11 | Contig76 | nuclear transport factor 2 |
| 12 | Contig78 | delta(12)-fatty-acid desaturase FAD2 |
| 13 | Contig81 | dolichyl-diphosphooligosaccharide--protein glycosyltransferase subunit STT3A |
| 14 | Contig85 | inactive beta-amylase 9 |
| 15 | Contig88 | 40S ribosomal protein S18 |
| 16 | Contig90 | ---NA--- |
| 17 | Contig91 | 60S ribosomal protein L23 |
| 18 | Contig93 | eukaryotic translation initiation factor 5A |
| 19 | Contig94 | NADP-dependent D-sorbitol-6-phosphate dehydrogenase-like |
| 20 | Contig97 | photosystem II reaction center W protein, chloroplastic |
| 21 | Contig99 | ubiquitin-conjugating enzyme E2 28 |
| 22 | Contig100 | calmodulin binding protein, putative |
| 23 | Contig114 | ---NA--- |
| 24 | Contig116 | 60S ribosomal protein L15-1 |
| 25 | Contig119 | alpha-1,4-glucan-protein synthase [UDP-forming] 2 |
| 26 | Contig124 | histone H1-like |
| 27 | Contig126 | cytochrome P450 83B1-like |
| 28 | Contig132 | 60S ribosomal protein L8 |
| 29 | Contig136 | protein BOBBER 1-like |
| 30 | Contig142 | 60S ribosomal protein L37-3-like |
| 31 | Contig146 | proteasome subunit alpha type-7 |
| 32 | Contig153 | dormancy-associated protein homolog 3 isoform X1 |
| 33 | Contig155 | UDP-glucose 6-dehydrogenase 5 |
| 34 | Contig156 | acetate/butyrate--CoA ligase AAE7, peroxisomal |
| 35 | Contig161 | protein SRC2 |
| 36 | Contig162 | ---NA--- |
| 37 | Contig163 | probable ADP-ribosylation factor GTPase-activating protein AGD5 |
| 38 | Contig172 | calreticulin-3 isoform X2 |
| 39 | Contig175 | hypothetical protein JCGZ_11403 |
| 40 | Contig177 | dnaJ homolog subfamily B member 1 |
| 41 | Contig178 | phosphoglycerate kinase, chloroplastic |
| 42 | Contig188 | malate dehydrogenase |
| 43 | Contig189 | S-adenosylmethionine synthase 1 |
| 44 | Contig191 | ---NA--- |
| 45 | Contig199 | 60S ribosomal protein L21-1 |
| 46 | Contig203 | predicted protein |
| 47 | Contig206 | plastocyanin A'/A'' |
| 48 | Contig213 | elongation factor 1-gamma-like |
| 49 | Contig214 | 60S ribosomal protein L17-2 |
| 50 | Contig216 | 60S ribosomal protein L32-1 |
| 51 | Contig217 | 60S ribosomal protein L18-2 |
| 52 | Contig219 | 60S ribosomal protein L12 |
| 53 | Contig220 | 60S ribosomal protein L10 |
| 54 | Contig226 | 40S ribosomal protein SA |
| 55 | Contig227 | 40S ribosomal protein S15a-1 |
| 56 | Contig232 | bark storage protein A |
| 57 | Contig243 | REF/SRPP-like protein At1g67360 |
| 58 | Contig263 | translocon-associated protein subunit beta |
| 59 | Contig265 | aquaporin TIP1-1 |
| 60 | Contig270 | S-adenosylmethionine synthase 1 |
| 61 | Contig281 | nascent polypeptide-associated complex subunit alpha-like protein 1 |
| 62 | Contig283 | bidirectional sugar transporter N3-like |
| 63 | Contig285 | isocitrate dehydrogenase [NAD] catalytic subunit 5, mitochondrial |
| 64 | Contig288 | histone H3.3 |
| 65 | Contig295 | Elongation factor 1-alpha |
| 66 | Contig308 | catalase isozyme 1 |
| 67 | Contig311 | B-cell receptor-associated protein 31-like |
| 68 | Contig314 | actin-depolymerizing factor 2-like |
| 69 | Contig315 | ABC transporter F family member 1 |
| 70 | Contig326 | zinc finger CCCH domain-containing protein 13-like isoform X1 |
| 71 | Contig332 | CBS domain-containing protein CBSX5-like |
| 72 | Contig334 | UDP-glucuronate 4-epimerase 1 |
| 73 | Contig337 | cyclin-d1-binding protein 1 like |
| 74 | Contig344 | probable carbohydrate esterase At4g34215 |
| 75 | Contig353 | putative vesicle-associated membrane protein 726 |
| 76 | Contig377 | hypothetical protein MANES_01G247200 |
| 77 | Contig385 | beta-glucosidase 12-like |
| 78 | Contig386 | NADPH-dependent aldo-keto reductase, chloroplastic-like |
| 79 | Contig391 | F-box protein CPR30 |
| 80 | Contig394 | lysm and putative peptidoglycan-binding domain-containing protein 2 |
| 81 | Contig395 | DUF1336 domain-containing protein |
| 82 | Contig403 | protein S-acyltransferase 10 |
| 83 | Contig410 | adenylate kinase 4 |
| 84 | Contig419 | caffeoylshikimate esterase |
| 85 | Contig430 | CHD3-type chromatin-remodeling factor PICKLE |
| 86 | Contig439 | fructose-1,6-bisphosphatase, chloroplastic |
| 87 | Contig456 | peptidyl-prolyl cis-trans isomerase CYP40 |
| 88 | Contig467 | NAC transcription factor 29-like |
| 89 | Contig473 | V-type proton ATPase subunit c''1 |
| 90 | Contig486 | probable WRKY transcription factor 48 |
| 91 | Contig487 | cytosolic sulfotransferase 15-like |
| 92 | Contig523 | chaperone protein DnaJ |
| 93 | Contig534 | NAC domain-containing protein 78 |
| 94 | Contig544 | nicotinamidase 1-like |
| 95 | Contig556 | uncharacterized protein LOC105631404 isoform X3 |
| 96 | Contig563 | annexin D2-like |
| 97 | Contig564 | zinc-finger homeodomain protein 9 |
| 98 | Contig566 | Epimerase domain-containing protein |
| 99 | Contig596 | endochitinase EP3 |
| 100 | Contig605 | iron-sulfur cluster co-chaperone protein HscB, mitochondrial |
| 101 | Contig634 | growth-regulating factor 1-like |
| 102 | Contig652 | CMP-sialic acid transporter 1 |
| 103 | Contig654 | Alpha/beta hydrolase fold |
| 104 | Contig658 | methionine aminopeptidase 2B |
| 105 | Contig664 | AT-hook motif nuclear-localized protein 20-like |
| 106 | Contig694 | probable xyloglucan endotransglucosylase/hydrolase protein 32 |
| 107 | Contig700 | peroxidase 42 |
| 108 | Contig705 | 60S ribosomal protein L15-1-like isoform X2 |
| 109 | Contig707 | catalase |
| 110 | Contig723 | homeobox protein ATH1-like |
| 111 | Contig726 | 3-oxoacyl-[acyl-carrier-protein] synthase I, chloroplastic |
| 112 | Contig730 | uncharacterized protein LOC110636409 |
| 113 | Contig731 | PHD finger protein ALFIN-LIKE 2-like |
| 114 | Contig750 | TBC1 domain family member 15 |
| 115 | Contig767 | ultraviolet-B receptor UVR8-like |
| 116 | Contig794 | lipid phosphate phosphatase epsilon 2, chloroplastic |
| 117 | Contig797 | polygalacturonase-inhibiting protein |
| 118 | Contig813 | probable protein phosphatase 2C 11 |
| 119 | Contig834 | heat stress transcription factor C-1-like |
| 120 | Contig836 | COP9 signalosome complex subunit 1 |
| 121 | Contig844 | TLC domain-containing protein At5g14285-like |
| 122 | Contig849 | U2 small nuclear ribonucleoprotein A' |
| 123 | Contig852 | nitrogen regulatory protein P-II homolog |
| 124 | Contig862 | NADH dehydrogenase [ubiquinone] flavoprotein 2, mitochondrial-like |
| 125 | Contig879 | GTP 3',8-cyclase, mitochondrial isoform X2 |
| 126 | Contig887 | protein IQ-DOMAIN 1 |
| 127 | Contig890 | tropinone reductase homolog At5g06060-like |
| 128 | Contig903 | AT3G11945-like protein |
| 129 | Contig904 | caffeoylshikimate esterase |
| 130 | Contig908 | O-acyltransferase WSD1-like |
| 131 | Contig917 | SPX domain-containing protein 1 |
| 132 | Contig922 | myb-related protein 308-like |
| 133 | Contig926 | Glutamate [NMDA] receptor subunit epsilon-1 |
| 134 | Contig927 | delta-aminolevulinic acid dehydratase 1, chloroplastic-like |
| 135 | Contig968 | uncharacterized protein LOC110603031 |
| 136 | Contig986 | nucleosome assembly protein 1;2 |
| 137 | Contig995 | collagen alpha-1(III) chain-like |
| 138 | Contig1000 | ER lumen protein-retaining receptor A |
| 139 | Contig1022 | 28 kDa ribonucleoprotein, chloroplastic-like |
| 140 | Contig1026 | N-glycosylase/DNA lyase OGG1 isoform X1 |
| 141 | Contig1032 | phosphatidylcholine transfer protein, putative |
| 142 | Contig1054 | probable inactive receptor kinase At5g67200 |
| 143 | Contig1055 | non-specific phospholipase C2 |
| 144 | Contig1097 | mitochondrial uncoupling protein 5 |
| 145 | Contig1098 | heat shock cognate protein 80 |
| 146 | Contig1101 | uncharacterized protein LOC105649755 |
| 147 | Contig1133 | Cytosolic beta-glucosidase |
| 148 | Contig1137 | 50S ribosomal protein L12, chloroplastic-like |
| 149 | Contig1151 | cysteine and histidine-rich domain-containing protein RAR1 |
| 150 | Contig1157 | uncharacterized protein LOC110634334 |
| 151 | Contig1188 | probable xyloglucan endotransglucosylase/hydrolase protein 8 |
| 152 | Contig1191 | transcription factor ILR3 |
| 153 | Contig1214 | thioredoxin H-type-like isoform X1 |
| 154 | Contig1216 | probable N-succinyldiaminopimelate aminotransferase DapC |
| 155 | Contig1231 | vegetative cell wall protein gp1 |
| 156 | Contig1242 | uncharacterized protein LOC110643816 isoform X1 |
| 157 | Contig1261 | probable hydroxyacylglutathione hydrolase 2, chloroplastic |
| 158 | Contig1264 | UPF0505 protein C16orf62 homolog isoform X2 |
| 159 | Contig1269 | guanine nucleotide-binding protein alpha-1 subunit |
| 160 | Contig1282 | histone H2A |
| 161 | Contig1341 | GRF1-interacting factor 1 |
| 162 | Contig1342 | uncharacterized membrane protein At1g16860-like |
| 163 | Contig1347 | KRR1 small subunit processome component homolog |
| 164 | Contig1350 | malate dehydrogenase, mitochondrial |
| 165 | Contig1359 | protein DEHYDRATION-INDUCED 19 homolog 4-like |
| 166 | Contig1365 | zinc finger protein 346-like isoform X4 |
| 167 | Contig1370 | chromatin remodeling protein EBS |
| 168 | Contig1379 | adenosylhomocysteinase |
| 169 | Contig1383 | probable S-adenosylmethionine-dependent methyltransferase At5g37970 |
| 170 | Contig1385 | universal stress protein A-like protein |
| 171 | Contig1387 | DUF538 domain-containing protein |
| 172 | Contig1388 | BAHD acyltransferase At5g47980-like |
| 173 | Contig1393 | L-type lectin-domain containing receptor kinase S.4-like |
| 174 | Contig1405 | ER lumen protein-retaining receptor |
| 175 | Contig1416 | protein cfxQ homolog isoform X2 |
| 176 | Contig1422 | small nuclear ribonucleoprotein Sm D2-like |
| 177 | Contig1437 | NF-kappa-B inhibitor-like protein 2 isoform 1 |
| 178 | Contig1452 | cathepsin B |
| 179 | Contig1473 | dual specificity tyrosine-phosphorylation-regulated kinase 1A-like isoform X1 |
| 180 | Contig1474 | UDP-glucuronate 4-epimerase 6-like |
| 181 | Contig1476 | annexin-like protein RJ4 isoform X2 |
| 182 | Contig1477 | alpha-glucan water dikinase, chloroplastic |
| 183 | Contig1478 | BAG family molecular chaperone regulator 1-like |
| 184 | Contig1488 | serine/threonine-protein kinase D6PKL1 |
| 185 | Contig1505 | nucleolar protein 58-like |
| 186 | Contig1528 | transmembrane protein 230-like |
| 187 | Contig1529 | uncharacterized protein At3g49720-like |
| 188 | Contig1530 | 50S ribosomal protein L27, chloroplastic |
| 189 | Contig1553 | BTB/POZ domain-containing protein At1g21780 |
| 190 | Contig1557 | Regulator of rDNA transcription protein 15 |
| 191 | Contig1561 | DUF724 domain-containing protein 6 |
| 192 | Contig1576 | BTB/POZ and TAZ domain-containing protein 3 |
| 193 | Contig1579 | protein ECERIFERUM 26-like |
| 194 | Contig1583 | probable WRKY transcription factor 70 |
| 195 | Contig1584 | putative laccase-9 |
| 196 | Contig1607 | Nucleus-like protein |
| 197 | Contig1619 | probable methyltransferase PMT13 |
| 198 | Contig1620 | ankyrin repeat-containing protein At5g02620-like |
| 199 | Contig1621 | pyruvate dehydrogenase E1 component subunit alpha-3, chloroplastic-like |
| 200 | Contig1624 | WD repeat-containing protein VIP3 |
| 201 | Contig1631 | putative phospholipid-transporting ATPase 9 |
| 202 | Contig1644 | cytidine deaminase 1-like |
| 203 | Contig1655 | (S)-coclaurine N-methyltransferase-like |
| 204 | Contig1657 | gamma-glutamyl peptidase 5-like |
| 205 | Contig1664 | uncharacterized protein LOC110654820 isoform X1 |
| 206 | Contig1668 | ethylene-responsive transcription factor 12 |
| 207 | Contig1673 | ubiquitin-conjugating enzyme E2 variant 1D |
| 208 | Contig1692 | DENN domain and WD repeat-containing protein SCD1 |
| 209 | Contig1696 | probable methyltransferase PMT28 |
| 210 | Contig1713 | mRNA-decapping enzyme subunit 2 |
| 211 | Contig1727 | 4-coumarate--CoA ligase 2-like |
| 212 | Contig1733 | auxin-responsive protein IAA18-like |
| 213 | Contig1755 | transcription factor bHLH137-like |
| 214 | Contig1761 | probable xyloglucan endotransglucosylase/hydrolase protein 30 |
| 215 | Contig1767 | 2-methylene-furan-3-one reductase-like |
| 216 | Contig1772 | zinc finger protein CONSTANS-LIKE 13 |
| 217 | Contig1779 | SPARC-like protein 1 |
| 218 | Contig1781 | stem-specific protein TSJT1-like |
| 219 | Contig1790 | leucine-rich repeat receptor-like protein |
| 220 | Contig1792 | protein FAR1-RELATED SEQUENCE 5 |
| 221 | Contig1799 | probable pyridoxal 5'-phosphate synthase subunit PDX1 |
| 222 | Contig1808 | probable complex I intermediate-associated protein 30 |
| 223 | Contig1815 | suppressor of RPS4-RLD 1 isoform X1 |
| 224 | Contig1822 | multiple organellar RNA editing factor 2, chloroplastic-like |
| 225 | Contig1826 | rhodanese-like domain-containing protein 4, chloroplastic |
| 226 | Contig1848 | cyclin-dependent protein kinase inhibitor SMR11-like |
| 227 | Contig1857 | TMV resistance protein N-like |
| 228 | Contig1858 | probable alpha-mannosidase At5g13980 |
| 229 | Contig1861 | F-box protein At5g50450-like |
| 230 | Contig1863 | 36.4 kDa proline-rich protein |
| 231 | Contig1878 | signal peptide peptidase-like 1 |
| 232 | Contig1885 | cytosolic sulfotransferase 15-like |
| 233 | Contig1895 | craniofacial development protein 1 |
| 234 | Contig1897 | benzyl alcohol O-benzoyltransferase-like |
| 235 | Contig1901 | pectinesterase inhibitor 3-like |
| 236 | Contig1904 | apyrase 2-like |
| 237 | Contig1905 | zinc ion-binding protein, putative |
| 238 | Contig1915 | ATP-dependent Clp protease proteolytic subunit-related protein 1, chloroplastic |
| 239 | Contig1932 | protein ETHYLENE INSENSITIVE 3-like |
| 240 | Contig1934 | protein ABA DEFICIENT 4, chloroplastic |
| 241 | Contig1961 | Sterile alpha motif, type 2 |
| 242 | Contig1964 | PRA1 family protein B3-like |
| 243 | Contig1994 | 26S proteasome non-ATPase regulatory subunit 14 homolog |
| 244 | Contig1995 | protein TRIGALACTOSYLDIACYLGLYCEROL 4, chloroplastic |
| 245 | Contig2003 | NADH dehydrogenase [ubiquinone] flavoprotein 2, mitochondrial-like |
| 246 | Contig2013 | 2-alkenal reductase (NADP(+)-dependent) |
| 247 | Contig2014 | uncharacterized protein LOC110639581 |
| 248 | Contig2015 | TVP38/TMEM64 family membrane protein slr0305 |
| 249 | Contig2018 | mitochondrial uncoupling protein 1-like |
| 250 | Contig2031 | V-type proton ATPase 16 kDa proteolipid subunit |
| 251 | Contig2039 | E3 ubiquitin-protein ligase SINAT2-like |
| 252 | Contig2041 | E3 ubiquitin-protein ligase At4g11680-like |
| 253 | Contig2058 | pre-mRNA-splicing factor ISY1 homolog |
| 254 | Contig2061 | syntaxin-22-like |
| 255 | Contig2071 | probable sugar phosphate/phosphate translocator At3g14410 |
| 256 | Contig2096 | universal stress protein A-like protein |
| 257 | Contig2125 | alcohol dehydrogenase |
| 258 | Contig2138 | plant UBX domain-containing protein 4 |
| 259 | Contig2146 | conserved hypothetical protein |
| 260 | Contig2159 | uncharacterized protein LOC105643541 isoform X1 |
| 261 | Contig2169 | uncharacterized protein LOC110622219 |
| 262 | Contig2184 | NAD_binding_10 domain-containing protein |
| 263 | Contig2188 | protein LURP-one-related 8-like |
| 264 | Contig2191 | immune-associated nucleotide-binding protein 9 |
| 265 | Contig2193 | probable serine/threonine-protein kinase At1g54610 |
| 266 | Contig2202 | probable dolichyl-diphosphooligosaccharide--protein glycosyltransferase subunit 3B |
| 267 | Contig2204 | probable voltage-gated potassium channel subunit beta |
| 268 | Contig2213 | GTP-binding nuclear protein Ran-3 |
| 269 | Contig2245 | glyoxylate/succinic semialdehyde reductase 1-like |
| 270 | Contig2248 | acyl-protein thioesterase 2 |
| 271 | Contig2264 | tRNA (guanine-N(7)-)-methyltransferase non-catalytic subunit wdr4 |
| 272 | Contig2274 | polygalacturonase At1g48100-like |
| 273 | Contig2290 | tRNA (cytosine(38)-C(5))-methyltransferase isoform X1 |
| 274 | Contig2299 | protein IQ-DOMAIN 14-like isoform X2 |
| 275 | Contig2325 | ubiquitin-conjugating enzyme E2 34-like |
| 276 | Contig2326 | DUF1223 domain-containing protein |
| 277 | Contig2329 | pyridoxal 5'-phosphate synthase-like subunit PDX1.2 |
| 278 | Contig2330 | ATP-dependent DNA helicase Q-like 4A |
| 279 | Contig2334 | protein ELC-like |
| 280 | Contig2337 | UPF0114 domain-containing protein |
| 281 | Contig2346 | mitogen-activated protein kinase kinase 3 |
| 282 | Contig2354 | SH3 domain-containing protein 1 |
| 283 | Contig2371 | probable folate-biopterin transporter 7 |
| 284 | Contig2384 | aspartyl protease family protein At5g10770 |
| 285 | Contig2394 | probable ADP-ribosylation factor GTPase-activating protein AGD14 isoform X1 |
| 286 | Contig2404 | transcription factor TCP14-like |
| 287 | Contig2417 | Leucine carboxyl methyltransferase |
| 288 | Contig2420 | ubiquitin-like domain-containing CTD phosphatase 1 isoform X1 |
| 289 | Contig2427 | Endonuclease or glycosyl hydrolase with C2H2-type zinc finger domain, putative isoform 1 |
| 290 | Contig2438 | biotin carboxyl carrier protein of acetyl-CoA carboxylase 2, chloroplastic-like |
| 291 | Contig2454 | putative BPI/LBP family protein At1g04970 isoform X2 |
| 292 | Contig2457 | early nodulin-like protein 1 |
| 293 | Contig2462 | squalene epoxidase |
| 294 | Contig2463 | sister chromatid cohesion protein PDS5 homolog A-like isoform X1 |
| 295 | Contig2476 | MOB kinase activator-like 1A |
| 296 | Contig2478 | mitochondrial inner membrane protein OXA1-like |
| 297 | Contig2496 | acetolactate synthase small subunit 2, chloroplastic-like |
| 298 | Contig2518 | probable LRR receptor-like serine/threonine-protein kinase At5g10290 |
| 299 | Contig2538 | NAC domain class transcription factor |
| 300 | Contig2550 | uncharacterized protein LOC110632777 |
| 301 | Contig2555 | peptide chain release factor PrfB1, chloroplastic isoform X1 |
| 302 | Contig2563 | uncharacterized protein LOC110636255 |
| 303 | Contig2568 | premnaspirodiene oxygenase-like |
| 304 | Contig2586 | serine/threonine-protein kinase ATM |
| 305 | Contig2624 | ras-related protein Rab11C |
| 306 | Contig2625 | sulfite reductase [ferredoxin], chloroplastic-like |
| 307 | Contig2633 | expansin-A6-like |
| 308 | Contig2635 | RING-H2 finger protein ATL54-like |
| 309 | Contig2638 | sterol 3-beta-glucosyltransferase UGT80B1 isoform X1 |
| 310 | Contig2657 | protein SHORT HYPOCOTYL IN WHITE LIGHT 1 |
| 311 | Contig2665 | probable 1-acylglycerol-3-phosphate O-acyltransferase |
| 312 | Contig2669 | putative capsid protein |
| 313 | Contig2693 | BAG family molecular chaperone regulator 4 |
| 314 | Contig2694 | WAT1-related protein At4g08300-like |
| 315 | Contig2703 | bidirectional sugar transporter SWEET10-like |
| 316 | Contig2704 | NAC domain-containing protein 100-like |
| 317 | Contig2712 | probable starch synthase 4, chloroplastic/amyloplastic isoform X2 |
| 318 | Contig2715 | coiled-coil domain-containing protein 93 |
| 319 | Contig2719 | PRA1 family protein D |
| 320 | Contig2729 | multiple organellar RNA editing factor 1, mitochondrial-like |
| 321 | Contig2739 | uncharacterized protein LOC105631234 isoform X2 |
| 322 | Contig2742 | cell wall protein RBR3-like |
| 323 | Contig2745 | caffeic acid 3-O-methyltransferase |
| 324 | Contig2784 | uncharacterized protein LOC105644756 |
| 325 | Contig2792 | N-alpha-acetyltransferase 35, NatC auxiliary subunit isoform X1 |
| 326 | Contig2801 | F-box protein SKIP23-like |
| 327 | Contig2802 | probable adenylate kinase 7, mitochondrial |
| 328 | Contig2815 | RHOMBOID-like protein 12, mitochondrial |
| 329 | Contig2827 | MFS transporter |
| 330 | Contig2852 | S-adenosylmethionine-dependent methyltransferase, putative |
| 331 | Contig2855 | ---NA--- |
| 332 | Contig2856 | probable protein phosphatase 2C 53 |
| 333 | Contig2857 | probable cinnamyl alcohol dehydrogenase 1 |
| 334 | Contig2860 | probable serine/threonine-protein kinase Cx32, chloroplastic |
| 335 | Contig2869 | mitochondrial substrate carrier family protein C-like |
| 336 | Contig2881 | ribosomal RNA processing protein 36 homolog |
| 337 | Contig2885 | ---NA--- |
| 338 | Contig2886 | DEAD-box ATP-dependent RNA helicase 1 isoform X1 |
| 339 | Contig2889 | 60S ribosomal protein L28-2 |
| 340 | Contig2898 | two-pore potassium channel 5 |
| 341 | Contig2903 | ribosome biogenesis protein WDR12 homolog |
| 342 | Contig2929 | B3 domain-containing transcription factor VRN1-like |
| 343 | Contig2942 | zinc finger protein CONSTANS-LIKE 4-like isoform X2 |
| 344 | Contig2983 | cysteine-rich repeat secretory protein 12 |
| 345 | Contig2995 | V-type proton ATPase subunit E |
| 346 | Contig2999 | calcium-dependent protein kinase 28-like |
| 347 | Contig3016 | AUGMIN subunit 1 |
| 348 | Contig3022 | protein cereblon |
| 349 | Contig3027 | probable serine/threonine-protein kinase PBL2 isoform X3 |
| 350 | Contig3028 | 60S ribosomal protein L7a-1 |
| 351 | Contig3035 | spastin isoform X1 |
| 352 | Contig3046 | uroporphyrinogen-III synthase, chloroplastic isoform X2 |
| 353 | Contig3051 | probably inactive leucine-rich repeat receptor-like protein kinase At3g28040 |
| 354 | Contig3053 | AT-hook motif nuclear-localized protein 15-like |
| 355 | Contig3069 | CBL-interacting serine/threonine-protein kinase 12-like |
| 356 | Contig3078 | Multicopper oxidase, type 1 |
| 357 | Contig3089 | 1-aminocyclopropane-1-carboxylate oxidase |
| 358 | Contig3102 | nicotinate phosphoribosyltransferase 2-like |
| 359 | Contig3104 | protein kinase PINOID |
| 360 | Contig3107 | catalytic, putative |
| 361 | Contig3122 | cellulose synthase A catalytic subunit 1 [UDP-forming] |
| 362 | Contig3156 | electron transporter, putative |
| 363 | Contig3189 | cycloartenol-C-24-methyltransferase |
| 364 | Contig3198 | catalytic, putative |
| 365 | Contig3201 | cytochrome P450 CYP82D47-like |
| 366 | Contig3221 | probable apyrase 7 |
| 367 | Contig3228 | protein PAT1 homolog 1-like |
| 368 | Contig3233 | 8-amino-7-oxononanoate synthase |
| 369 | Contig3252 | organ-specific protein S2-like |
| 370 | Contig3253 | O-glucosyltransferase rumi homolog |
| 371 | Contig3273 | Sec20 family protein |
| 372 | Contig3282 | equilibrative nucleotide transporter 8 |
| 373 | Contig3286 | NADH dehydrogenase [ubiquinone] 1 alpha subcomplex assembly factor 3 |
| 374 | Contig3293 | CBL-interacting serine/threonine-protein kinase 6-like |
| 375 | Contig3294 | IAA-alanine resistance protein 1 |
| 376 | Contig3295 | protein EIN4 |
| 377 | Contig3296 | mannosyl-oligosaccharide 1,2-alpha-mannosidase MNS1 |
| 378 | Contig3305 | probable E3 ubiquitin-protein ligase LOG2 |
| 379 | Contig3306 | DUF716 domain-containing protein |
| 380 | Contig3307 | eukaryotic translation initiation factor 2 subunit beta |
| 381 | Contig3311 | DNA-binding protein BIN4 |
| 382 | Contig3317 | coiled-coil domain-containing protein 18-like |
| 383 | Contig3325 | probable nucleoredoxin 2 isoform X1 |
| 384 | Contig3328 | U3 small nucleolar ribonucleoprotein protein IMP4 |
| 385 | Contig3338 | MADS-box protein SVP |
| 386 | Contig3366 | ethylene-responsive transcription factor 4 |
| 387 | Contig3380 | biotin carboxyl carrier protein of acetyl-CoA carboxylase 2, chloroplastic-like |
| 388 | Contig3382 | 3-ketodihydrosphingosine reductase |
| 389 | Contig3395 | 26S protease regulatory subunit S10B homolog B |
| 390 | Contig3406 | probable protein phosphatase 2C 55 isoform X1 |
| 391 | Contig3408 | protein ABHD11 |
| 392 | Contig3409 | Glycosyl transferase, family 14 |
| 393 | Contig3416 | peroxidase 72-like |
| 394 | Contig3417 | premnaspirodiene oxygenase |
| 395 | Contig3429 | hypothetical protein MANES_13G009200 |
| 396 | Contig3444 | 26S protease regulatory subunit 7 |
| 397 | Contig3451 | probable pyridoxal 5'-phosphate synthase subunit PDX2 |
| 398 | Contig3475 | serine/threonine-protein kinase SRK2A-like |
| 399 | Contig3476 | BSD domain-containing family protein |
| 400 | Contig3510 | feruloyl CoA ortho-hydroxylase 1-like |
| 401 | Contig3511 | 3-hydroxybutyryl-CoA dehydrogenase-like |
| 402 | Contig3519 | ring finger containing protein, putative |
| 403 | Contig3525 | ZZ-type zinc finger-containing 3 |
| 404 | Contig3528 | protein GDAP2 homolog |
| 405 | Contig3536 | sericin 1-like |
| 406 | Contig3582 | ABC transporter F family member 4-like |
| 407 | Contig3588 | protein SIEVE ELEMENT OCCLUSION B |
| 408 | Contig3599 | thioredoxin F-type, chloroplastic-like |
| 409 | Contig3634 | RNA polymerase II C-terminal domain phosphatase-like 4 |
| 410 | Contig3636 | C-type lectin receptor-like tyrosine-protein kinase At1g52310 |
| 411 | Contig3654 | auxin-binding protein ABP19a |
| 412 | Contig3656 | serine/threonine-protein kinase-like protein CCR4 |
| 413 | Contig3687 | autophagy-related protein 18a-like |
| 414 | Contig3692 | 50S ribosomal protein L25 |
| 415 | Contig3712 | 60S ribosomal protein L19 |
| 416 | Contig3715 | mannose-P-dolichol utilization defect 1 protein homolog 2 |
| 417 | Contig3716 | uncharacterized protein LOC110610918 isoform X2 |
| 418 | Contig3737 | F-box/FBD/LRR-repeat protein At1g13570-like |
| 419 | Contig3753 | UBN2_2 domain-containing protein |
| 420 | Contig3757 | nudix hydrolase 3 |
| 421 | Contig3761 | protein CHAPERONE-LIKE PROTEIN OF POR1, chloroplastic |
| 422 | Contig3779 | proline synthase co-transcribed bacterial homolog protein |
| 423 | Contig3780 | allantoinase isoform X2 |
| 424 | Contig3788 | monocopper oxidase-like protein sku5 |
| 425 | Contig3802 | developmentally-regulated G-protein 3 |
| 426 | Contig3807 | probable WRKY transcription factor 40 |
| 427 | Contig3834 | mediator of RNA polymerase II transcription subunit 25 isoform X2 |
| 428 | Contig3848 | probable BOI-related E3 ubiquitin-protein ligase 3 |
| 429 | Contig3855 | cytochrome P450 734A1-like |
| 430 | Contig3862 | nuclear transcription factor Y subunit B-10 |
| 431 | Contig3868 | non-specific phospholipase C3-like |
| 432 | Contig3872 | probable mediator of RNA polymerase II transcription subunit 26c |
| 433 | Contig3880 | premnaspirodiene oxygenase-like |
| 434 | Contig3883 | probable serine/threonine-protein kinase WNK4 isoform X1 |
| 435 | Contig3890 | aspartyl protease family protein 2 |
| 436 | Contig3894 | 12-oxophytodienoate reductase 3 |
| 437 | Contig3900 | glyceraldehyde-3-phosphate dehydrogenase, cytosolic |
| 438 | Contig3912 | 36.4 kDa proline-rich protein-like |
| 439 | Contig3923 | uncharacterized protein LOC110639064 |
| 440 | Contig3925 | GATA transcription factor 26 |
| 441 | Contig3934 | polyadenylate-binding protein-interacting protein 4-like isoform X1 |
| 442 | Contig3940 | probable LRR receptor-like serine/threonine-protein kinase IRK |
| 443 | Contig3948 | cyclic nucleotide-gated ion channel 1 |
| 444 | Contig3962 | pentatricopeptide repeat-containing protein At3g49240 |
| 445 | Contig3971 | lysM domain receptor-like kinase 3 |
| 446 | Contig3975 | expansin-A10-like isoform X1 |
| 447 | Contig3981 | Transmembrane protein |
| 448 | Contig3987 | uncharacterized membrane protein YjcL |
| 449 | Contig3998 | leucine--tRNA ligase, cytoplasmic-like |
| 450 | Contig4000 | F-box protein At5g39250-like |
| 451 | Contig4001 | 28 kDa ribonucleoprotein, chloroplastic |
| 452 | Contig4013 | exocyst complex component SEC15A |
| 453 | Contig4019 | elongation factor 1-alpha |
| 454 | Contig4030 | phosphoenolpyruvate carboxylase kinase 1-like |
| 455 | Contig4039 | basic 7S globulin-like |
| 456 | Contig4052 | ribosomal RNA small subunit methyltransferase nep-1-like |
| 457 | Contig4061 | GDSL esterase/lipase At3g27950 |
| 458 | Contig4074 | BI1-like protein |
| 459 | Contig4077 | vacuolar protein sorting-associated protein 8 homolog |
| 460 | Contig4080 | deSI-like protein At4g17486 isoform X1 |
| 461 | Contig4081 | transcription termination factor MTERF4, chloroplastic |
| 462 | Contig4120 | catalytic, putative |
| 463 | Contig4122 | probable protein phosphatase 2C 46 |
| 464 | Contig4125 | elongation of fatty acids protein 3-like |
| 465 | Contig4129 | oxygen-evolving enhancer protein 2, chloroplastic |
| 466 | Contig4135 | inorganic pyrophosphatase 1-like |
| 467 | Contig4141 | galacturonokinase |
| 468 | Contig4165 | short-chain dehydrogenase TIC 32, chloroplastic-like |
| 469 | Contig4190 | two-component response regulator ORR3-like |
| 470 | Contig4200 | ribosomal L1 domain-containing protein 1-like |
| 471 | Contig4201 | abscisate beta-glucosyltransferase-like |
| 472 | Contig4202 | BTB/POZ domain-containing protein FBL11 isoform X2 |
| 473 | Contig4206 | histone-lysine N-methyltransferase, H3 lysine-9 specific SUVH4-like |
| 474 | Contig4224 | RING/U-box protein, putative isoform 2 |
| 475 | Contig4227 | protein LATERAL ROOT PRIMORDIUM 1 isoform X1 |
| 476 | Contig4245 | uncharacterized protein At1g76660-like isoform X2 |
| 477 | Contig4276 | acetylornithine deacetylase |
| 478 | Contig4282 | UDP-glucuronic acid decarboxylase 1-like |
| 479 | Contig4287 | glutathione S-transferase DHAR3, chloroplastic |
| 480 | Contig4290 | probable caffeine synthase 4 |
| 481 | Contig4301 | prosaposin |
| 482 | Contig4317 | calcium uptake protein, mitochondrial |
| 483 | Contig4321 | E3 ubiquitin-protein ligase RING1-like |
| 484 | Contig4355 | dna oxidative demethylase alkbh2 |
| 485 | Contig4387 | vacuolar-sorting receptor 3-like |
| 486 | Contig4394 | AMP deaminase |
| 487 | Contig4423 | OTU domain-containing protein At3g57810-like |
| 488 | Contig4437 | protein CHLOROPLAST IMPORT APPARATUS 2-like |
| 489 | Contig4451 | monoacylglycerol lipase ABHD6-like |
| 490 | Contig4452 | V-type proton ATPase subunit C |
| 491 | Contig4468 | pumilio homolog 2-like isoform X1 |
| 492 | Contig4474 | polyadenylate-binding protein-interacting protein 3 isoform X1 |
| 493 | Contig4482 | chlorophyll a-b binding protein CP29.3, chloroplastic |
| 494 | Contig4486 | TOM1-like protein 2 isoform X1 |
| 495 | Contig4488 | methylsterol monooxygenase 1-1-like |
| 496 | Contig4500 | uncharacterized serine-rich protein C215.13 |
| 497 | Contig4501 | putative ubiquitin-conjugating enzyme E2 38 isoform X1 |
| 498 | Contig4508 | pentatricopeptide repeat-containing protein At1g06270 |
| 499 | Contig4509 | serine/threonine-protein kinase STN7, chloroplastic |
| 500 | Contig4513 | uncharacterized protein LOC110653988 |
| 501 | Contig4514 | putative ALA-interacting subunit 2 |
| 502 | Contig4521 | alcohol dehydrogenase-like 2 |
| 503 | Contig4522 | superoxide dismutase [Mn], mitochondrial |
| 504 | Contig4529 | F-box/kelch-repeat protein At1g16250 isoform X1 |
| 505 | Contig4534 | peroxisomal adenine nucleotide carrier 1 |
| 506 | Contig4551 | probable serine/threonine-protein kinase At5g41260 |
| 507 | Contig4575 | ras-related protein RABF1 |
| 508 | Contig4581 | DUF3754 domain-containing protein |
| 509 | Contig4584 | 50S ribosomal protein L25-like |
| 510 | Contig4597 | protein FMP32, mitochondrial-like |
| 511 | Contig4606 | transcription factor MYB3R-3-like |
| 512 | Contig4609 | zinc/iron-chelating domain protein |
| 513 | Contig4626 | 4-diphosphocytidyl-2-C-methyl-D-erythritol kinase, chloroplastic |
| 514 | Contig4647 | protein SRC2 homolog |
| 515 | Contig4652 | phosducin-like protein 3 |
| 516 | Contig4653 | COMPASS-like H3K4 histone methylase component WDR5A |
| 517 | Contig4671 | glutathione S-transferase zeta class-like |
| 518 | Contig4672 | delta(3,5)-Delta(2,4)-dienoyl-CoA isomerase, peroxisomal |
| 519 | Contig4681 | tRNA-dihydrouridine synthase B |
| 520 | Contig4683 | COP9 signalosome complex subunit 3 |
| 521 | Contig4696 | BES1/BZR1 homolog protein 2-like |
| 522 | Contig4698 | protein AUXIN SIGNALING F-BOX 2-like |
| 523 | Contig4700 | vesicle-associated protein 4-2-like |
| 524 | Contig4701 | 26S proteasome non-ATPase regulatory subunit 7 homolog A |
| 525 | Contig4707 | probable leucine-rich repeat receptor-like protein kinase At1g35710 |
| 526 | Contig4714 | universal stress protein A-like protein |
| 527 | Contig4715 | PREDICTED: uncharacterized protein LOC8287412 |
| 528 | Contig4731 | protein THYLAKOID FORMATION1, chloroplastic |
| 529 | Contig4734 | GTP-binding protein YPTM2 |
| 530 | Contig4740 | CBL-interacting serine/threonine-protein kinase 11 |
| 531 | Contig4742 | heat shock cognate protein 80 |
| 532 | Contig4752 | 60S ribosomal protein L9 |
| 533 | Contig4756 | elongation of fatty acids protein 3-like |
| 534 | Contig4759 | paraspeckle component 1 |
| 535 | Contig4763 | homocysteine S-methyltransferase 2-like isoform X1 |
| 536 | Contig4764 | aspartyl protease family protein At5g10770-like |
| 537 | Contig4783 | probable calcium-binding protein CML49 |
| 538 | Contig4792 | soluble inorganic pyrophosphatase 4-like |
| 539 | Contig4796 | bZIP transcription factor 11-like |
| 540 | Contig4812 | WD repeat-containing protein LWD1 |
| 541 | Contig4822 | 60S ribosomal protein L5 |
| 542 | Contig4828 | protein SPA1-RELATED 2 isoform X1 |
| 543 | Contig4836 | phosphoenolpyruvate/phosphate translocator 1, chloroplastic |
| 544 | Contig4845 | splicing factor U2af small subunit B-like |
| 545 | Contig4854 | ubiquitin-conjugating enzyme E2 32 |
| 546 | Contig4859 | protein NRT1/ PTR FAMILY 6.4 |
| 547 | Contig4867 | cell division control protein 2 homolog C |
| 548 | Contig4871 | chlorophyll a-b binding protein CP26, chloroplastic |
| 549 | Contig4881 | ethylene-responsive transcription factor RAP2-12-like |
| 550 | Contig4928 | thiosulfate sulfurtransferase 16, chloroplastic-like |
| 551 | Contig4954 | cytochrome c1-2, heme protein, mitochondrial |
| 552 | Contig4955 | lysophospholipid acyltransferase 1-like |
| 553 | Contig4963 | probable adenylate kinase 6, chloroplastic |
| 554 | Contig4968 | flag-tagged protein kinase domain of putative mitogen-activated protein kinase kinase kinase |
| 555 | Contig4974 | dnaJ homolog subfamily B member 6 |
| 556 | Contig4983 | B-box zinc finger protein 24-like |
| 557 | Contig4984 | glyceraldehyde-3-phosphate dehydrogenase GAPCP2, chloroplastic |
| 558 | Contig4986 | 50S ribosomal protein L13, chloroplastic |
| 559 | Contig4991 | 40S ribosomal protein S4-1 |
| 560 | Contig5000 | F-box protein SKIP14 |
| 561 | Contig5008 | caffeoylshikimate esterase-like |
| 562 | Contig5009 | DNA-directed RNA polymerase II subunit 1 |
| 563 | Contig50110 | pointed first leaf |
| 564 | Contig5012 | ubiquitin-like modifier-activating enzyme 5 |
| 565 | Contig5030 | auxin-responsive protein IAA4-like |
| 566 | Contig5040 | GTP-binding protein YPTM2 |
| 567 | Contig5045 | REF/SRPP-like protein At3g05500 |
| 568 | Contig5057 | PHD finger family protein |
| 569 | Contig5064 | alpha-amylase isoform X1 |
| 570 | Contig5069 | Glycosyl transferase |
| 571 | Contig5083 | serine/threonine-protein kinase 16 |
| 572 | Contig5095 | acyl-CoA-binding domain-containing protein 3-like |
| 573 | Contig5117 | sarcoplasmic reticulum histidine-rich calcium-binding protein-like |
| 574 | Contig5122 | ethylene-responsive transcription factor RAP2-7 isoform X1 |
| 575 | Contig5131 | pleiotropic drug resistance protein 1-like |
| 576 | Contig5138 | cell division cycle protein 48 homolog |
| 577 | Contig5142 | transcription initiation factor IIF subunit beta-like |
| 578 | Contig5158 | ubiquitin-conjugating enzyme E2 10 |
| 579 | Contig5172 | LRR receptor-like serine/threonine-protein kinase FLS2 |
| 580 | Contig5182 | polyadenylate-binding protein RBP45B-like |
| 581 | Contig5189 | T-complex protein 1 subunit eta |
| 582 | Contig5196 | L-idonate 5-dehydrogenase |
| 583 | Contig5199 | BSD domain-containing family protein |
| 584 | Contig5207 | aspartic proteinase-like |
| 585 | Contig5210 | protein TRANSPARENT TESTA GLABRA 1-like |
| 586 | Contig5212 | transcription factor MYB44-like |
| 587 | Contig5219 | probable aquaporin TIP3-2 |
| 588 | Contig5224 | enoyl-CoA hydratase 2, peroxisomal |
| 589 | Contig5233 | ubiquitin carboxyl-terminal hydrolase 4 isoform X1 |
| 590 | Contig5235 | leucoanthocyanidin dioxygenase |
| 591 | Contig5261 | GDSL esterase/lipase 1-like |
| 592 | Contig5275 | calcium permeable stress-gated cation channel 1-like |
| 593 | Contig5277 | E3 ubiquitin-protein ligase RDUF1-like |
| 594 | Contig5279 | 60S ribosomal protein L7a-1 |
| 595 | Contig5287 | hypersensitive-induced response protein 2 |
| 596 | Contig5292 | protein argonaute 4-like |
| 597 | Contig5303 | ATP synthase subunit gamma, mitochondrial |
| 598 | Contig5314 | eukaryotic translation initiation factor 3 subunit C-like |
| 599 | Contig5318 | spermidine synthase 1 |
| 600 | Contig5320 | dehydrin ERD14 |
| 601 | Contig5327 | ricin precursor |
| 602 | Contig5330 | DUF642 family protein |
| 603 | Contig5333 | vestitone reductase-like |
| 604 | Contig5338 |  |
| 605 | Contig5347 | fructose-bisphosphate aldolase 3, chloroplastic |
| 606 | Contig5363 | eukaryotic translation initiation factor 3 subunit F-like |
| 607 | Contig5383 | ferredoxin--NADP reductase, leaf isozyme, chloroplastic |
| 608 | Contig5387 | probable tyrosine-protein phosphatase At1g05000 |
| 609 | Contig5388 | protein LHY-like isoform X1 |
| 610 | Contig5395 | DELLA protein GAI-like |
| 611 | Contig5398 | probable aquaporin PIP2-2 |
| 612 | Contig5404 | oleoyl-acyl carrier protein thioesterase 1, chloroplastic-like |
| 613 | Contig5416 | ribosome biogenesis protein NSA2 homolog |
| 614 | Contig5429 | stem-specific protein TSJT1-like |
| 615 | Contig5430 | 40S ribosomal protein S3-3 |
| 616 | Contig5442 | catalase isozyme 3 |
| 617 | Contig5444 | lipid phosphate phosphatase gamma, chloroplastic |
| 618 | Contig5459 | putative lactoylglutathione lyase |
| 619 | Contig5465 | glutamate decarboxylase |
| 620 | Contig5467 | mitogen-activated protein kinase 3 |
| 621 | Contig5482 | oxygen-evolving enhancer protein 1, chloroplastic |
| 622 | Contig5510 | carbonic anhydrase, chloroplastic |
| 623 | Contig5512 | protein BPS1, chloroplastic-like |
| 624 | Contig5518 | ras-related protein RABA1d |
| 625 | Contig5528 | DNA-damage-repair/toleration protein DRT111, chloroplastic |
| 626 | Contig5531 | transketolase, chloroplastic |
| 627 | Contig5534 | AAA-type ATPase family protein |
| 628 | Contig5548 | la-related protein 1C-like |
| 629 | Contig5550 | xyloglucan endotransglucosylase/hydrolase protein 9 |
| 630 | Contig5554 | eukaryotic translation initiation factor 2 subunit alpha homolog |
| 631 | Contig5560 | cysteine proteinase COT44 |
| 632 | Contig5562 | triose phosphate/phosphate translocator, chloroplastic isoform X1 |
| 633 | Contig5569 | uncharacterized protein LOC110640062 |
| 634 | Contig5572 | uncharacterized protein LOC110672407 |
| 635 | Contig5581 | peroxisomal membrane protein 11C |
| 636 | Contig5593 | succinate dehydrogenase [ubiquinone] iron-sulfur subunit 2, mitochondrial |
| 637 | Contig5597 | ras-related protein RABA5d-like |
| 638 | Contig5604 | NEDD8-conjugating enzyme Ubc12 |
| 639 | Contig5609 | cinnamoyl-CoA reductase 1-like |
| 640 | Contig5612 | protein DETOXIFICATION 24 |
| 641 | Contig5623 | elongation factor 2 |
| 642 | Contig5630 | eukaryotic translation initiation factor 3 subunit I-like |
| 643 | Contig5634 | triosephosphate isomerase, cytosolic |
| 644 | Contig5642 | B2 protein-like |
| 645 | Contig5647 | zinc finger CCCH domain-containing protein 55-like isoform X1 |
| 646 | Contig5651 | proteasome subunit beta type-5 |
| 647 | Contig5655 | aquaporin TIP2-1 |
| 648 | Contig5669 | peroxidase 12-like |
| 649 | Contig5671 | dnajc14 protein, putative |
| 650 | Contig5677 | phosphoglucomutase, cytoplasmic |
| 651 | Contig5688 | protein DEHYDRATION-INDUCED 19 homolog 3-like |
| 652 | Contig5704 | dehydrodolichyl diphosphate synthase 2 |
| 653 | Contig5714 | proteasome subunit alpha type-3 |
| 654 | Contig5715 | PUTATIVE TYPE 1 MEMBRANE family protein |
| 655 | Contig5733 | A_thal_3526 domain-containing protein |
| 656 | Contig5734 | secretory carrier-associated membrane protein 4 |
| 657 | Contig5738 | mitochondrial uncoupling protein 5 |
| 658 | Contig5744 | Voltage-dependent L-type calcium channel subunit alpha-1F |
| 659 | Contig5753 | ---NA--- |
| 660 | Contig5756 | derlin-2.2 |
| 661 | Contig5762 | 40S ribosomal protein S2-4-like |
| 662 | Contig5772 | 60S ribosomal protein L4 |
| 663 | Contig5780 | UV-B-induced protein At3g17800, chloroplastic isoform X2 |
| 664 | Contig5784 | probable protein disulfide-isomerase A6 |
| 665 | Contig5786 | reticulon-like protein B5 |
| 666 | Contig5798 | aspartyl protease family protein At5g10770-like |
| 667 | Contig5802 | squamosa promoter-binding-like protein 14 |
| 668 | Contig5832 | bifunctional nuclease 1-like |
| 669 | Contig5839 | ribosome-inactivating protein gelonin |
| 670 | Contig5873 | vacuolar protein sorting-associated protein 26A |
| 671 | Contig5885 | eukaryotic translation initiation factor-like |
| 672 | Contig5888 | peroxisome biogenesis factor 10 |
| 673 | Contig5899 | mRNA for AR781 family protein |
| 674 | Contig5947 | probable galacturonosyltransferase-like 3 |
| 675 | Contig5960 | probable inactive purple acid phosphatase 29 |
| 676 | Contig5962 | transcription factor bHLH104-like isoform X1 |
| 677 | Contig5986 | probable aquaporin PIP2-8 |
| 678 | Contig5987 | putative ER lumen protein-retaining receptor C28H8.4 |
| 679 | Contig5988 | UDP-arabinopyranose mutase 1 |
| 680 | Contig5997 | monosaccharide-sensing protein 2-like |
| 681 | Contig6015 | 2-Cys peroxiredoxin BAS1, chloroplastic |
| 682 | Contig6032 | histidine biosynthesis bifunctional protein hisIE, chloroplastic |
| 683 | Contig6033 | zinc finger A20 and AN1 domain-containing stress-associated protein 1 |
| 684 | Contig6051 | F-box protein At1g61340-like |
| 685 | Contig6054 | PRA1 family protein B4 |
| 686 | Contig6060 | B2 protein |
| 687 | Contig6064 | PHD finger protein ALFIN-LIKE 1-like |
| 688 | Contig6072 | triosephosphate isomerase, chloroplastic-like |
| 689 | Contig6075 | chloroplast stem-loop binding protein of 41 kDa a, chloroplastic |
| 690 | Contig6083 | serine carboxypeptidase-like 45 |
| 691 | Contig6096 | U4/U6.U5 tri-snRNP-associated protein 2-like |
| 692 | Contig6108 | GDSL esterase/lipase EXL3-like |
| 693 | Contig6117 | serine/threonine-protein kinase ATM |
| 694 | Contig6120 | TIP41-like protein |
| 695 | Contig6124 | stearoyl-[acyl-carrier-protein] 9-desaturase 6, chloroplastic-like |
| 696 | Contig6130 | protodermal factor 1 |
| 697 | Contig6144 | ---NA--- |
| 698 | Contig6158 | proteasome subunit alpha type-1-B-like |
| 699 | Contig6170 | vacuolar protein sorting-associated protein 32 homolog 2 |
| 700 | Contig6203 | ras-related protein RABE1c |
| 701 | Contig6228 | taxadien-5-alpha-ol O-acetyltransferase-like |
| 702 | Contig6229 | scarecrow-like protein 6 |
| 703 | Contig6247 | glutamyl-tRNA reductase-binding protein, chloroplastic |
| 704 | Contig6248 | peroxidase 64-like |
| 705 | Contig6253 | DNA-damage-repair/toleration protein DRT100-like |
| 706 | Contig6277 | nucleoside diphosphate kinase 3-like |
| 707 | Contig6284 | Transmembrane protein |
| 708 | Contig6287 | proteasome subunit beta type-7-B |
| 709 | Contig6290 | basic 7S globulin-like |
| 710 | Contig6296 | proteasome subunit beta type-1 |
| 711 | Contig6302 | 30S ribosomal protein S1, chloroplastic |
| 712 | Contig6304 | 40S ribosomal protein S7 |
| 713 | Contig6313 | RING/FYVE/PHD zinc finger superfamily protein isoform 3 |
| 714 | Contig6322 | ATP synthase gamma chain, chloroplastic |
| 715 | Contig6343 | SAGA-associated factor 29-like isoform X1 |
| 716 | Contig6364 | mitogen-activated protein kinase kinase 5-like |
| 717 | Contig6373 | mpv17-like protein isoform X1 |
| 718 | Contig6391 | probable xyloglucan endotransglucosylase/hydrolase protein B |
| 719 | Contig6395 | ras-related protein RABB1c |
| 720 | Contig6400 | naringenin,2-oxoglutarate 3-dioxygenase |
| 721 | Contig6401 | probable peroxygenase 3 |
| 722 | Contig6411 | probable xyloglucan galactosyltransferase GT19 |
| 723 | Contig6433 | 2-oxoglutarate dehydrogenase, mitochondrial-like |
| 724 | Contig6435 | glyceraldehyde-3-phosphate dehydrogenase B, chloroplastic |
| 725 | Contig6439 | syntaxin-132 |
| 726 | Contig6449 | cysteine-rich receptor-like protein kinase 10 |
| 727 | Contig6461 | bZIP transcription factor 53 |
| 728 | Contig6481 | tetraspanin-8-like |
| 729 | Contig6489 | stress-related protein |
| 730 | Contig6500 | stress response protein NST1 |
| 731 | Contig6509 | PGR5-like protein 1A, chloroplastic |
| 732 | Contig6516 | haloacid dehalogenase-like hydrolase |
| 733 | Contig6518 | probable methyltransferase PMT14 |
| 734 | Contig6521 | ASC1-like protein |
| 735 | Contig6524 | probable aspartyl aminopeptidase |
| 736 | Contig6543 | hydrolase, putative |
| 737 | Contig6554 | sucrose transporter 1 |
| 738 | Contig6572 | protein DEHYDRATION-INDUCED 19 homolog 3-like isoform X1 |
| 739 | Contig6577 | 2-methyl-6-phytyl-1,4-hydroquinone methyltransferase, chloroplastic |
| 740 | Contig6583 | probable CCR4-associated factor 1 homolog 7 |
| 741 | Contig6603 | glucose-6-phosphate/phosphate translocator 2, chloroplastic |
| 742 | Contig6642 | pectinesterase-like |
| 743 | Contig6643 | elongator complex protein 3 |
| 744 | Contig6653 | CBS domain-containing protein CBSX5-like |
| 745 | Contig6654 | outer envelope pore protein 37, chloroplastic |
| 746 | Contig6662 | non-specific lipid transfer protein GPI-anchored 1-like |
| 747 | Contig6666 | GDSL esterase/lipase At3g26430-like |
| 748 | Contig6672 | GDSL esterase/lipase At1g29670-like |
| 749 | Contig6675 | 26S protease regulatory subunit 6A homolog |
| 750 | Contig6678 | chitinase-like protein 2 |
| 751 | Contig6682 | photosystem I reaction center subunit XI, chloroplastic |
| 752 | Contig6693 | 2S albumin |
| 753 | Contig6694 | uncharacterized protein LOC105648340 |
| 754 | Contig6701 | eukaryotic initiation factor 4A-8 |
| 755 | Contig6718 | 2S albumin |
| 756 | Contig6720 | legumin B |
| 757 | Contig6725 | heme-binding-like protein At3g10130, chloroplastic |
| 758 | Contig6745 | PREDICTED: uncharacterized protein LOC105140363 |
| 759 | Contig6751 | non-specific lipid-transfer protein A-like |
| 760 | Contig6765 | Ribosomal protein L30/L7 family protein |
| 761 | Contig6768 | non-specific lipid-transfer protein A-like |
| 762 | Contig6779 | photosystem II 10 kDa polypeptide, chloroplastic |
| 763 | Contig6785 | uncharacterized protein LOC110633589 |
| 764 | Contig6790 | non-specific lipid-transfer protein A-like |
| 765 | Contig6793 | probable aquaporin PIP-type 7a |
| 766 | Contig6802 | ---NA--- |
| 767 | Contig6833 | dehydrin HIRD11 |
| 768 | Contig6837 | hypothetical protein MANES_01G247900 |
| 769 | Contig6839 | sm-like protein LSM3B |
| 770 | Contig6842 | sorcin-like isoform X2 |
| 771 | Contig6847 | uncharacterized protein LOC105633102 |
| 772 | Contig6863 | MFP1 attachment factor 1-like |
| 773 | Contig6881 | chromatin-remodeling complex subunit ies6 |
| 774 | Contig6889 | succinate-semialdehyde dehydrogenase, mitochondrial |
| 775 | Contig6901 | 60S acidic ribosomal protein P3-like |
| 776 | Contig6911 | delta(12)-fatty-acid desaturase FAD2-like |
| 777 | Contig6912 | 14 kDa proline-rich protein DC2.15-like |
| 778 | Contig6914 | ---NA--- |
| 779 | Contig6962 | stress-response A/B barrel domain-containing protein HS1-like |
| 780 | Contig6963 | cyprosin-like isoform X1 |
| 781 | Contig6970 | 60s ribosomal protein l31 |
| 782 | Contig6972 | 60S ribosomal protein L39-1 |
| 783 | Contig6973 | ---NA--- |
| 784 | Contig6996 | triosephosphate isomerase, cytosolic |
| 785 | Contig6998 | small nuclear ribonucleoprotein SmD3b |
| 786 | Contig7004 | pathogenesis-related protein 1 |
| 787 | Contig7014 | 18 kDa seed maturation protein |
| 788 | Contig7020 | methyl-CpG-binding domain-containing protein 4-like |
| 789 | Contig7025 | 3-ketoacyl-CoA thiolase 2, peroxisomal |
| 790 | Contig7037 | cytochrome c |
| 791 | Contig7041 | stress-associated endoplasmic reticulum protein 2 |
| 792 | Contig7054 | 60S ribosome subunit biogenesis protein NIP7 homolog |
| 793 | Contig7094 | pyruvate kinase, cytosolic isozyme |
| 794 | Contig7101 | UPF0057 membrane protein At4g30660-like |
| 795 | Contig7104 | histone H4 |
| 796 | Contig7105 | 18 kDa seed maturation protein-like |
| 797 | Contig7107 | 60S acidic ribosomal protein P2A |
| 798 | Contig7115 | inositol-3-phosphate synthase |
| 799 | Contig7123 | ---NA--- |
| 800 | Contig7131 | uncharacterized protein LOC110660011 isoform X2 |
| 801 | Contig7133 | lipid transfer protein |
| 802 | Contig7142 | enhancer of mRNA-decapping protein 4 |
| 803 | Contig7149 | Wound-responsive family protein |
| 804 | Contig7167 | 40S ribosomal protein S10-1-like |
| 805 | Contig7190 | elongation factor 1-alpha |
| 806 | Contig7191 | vicilin-like antimicrobial peptides 2-2 |
| 807 | Contig7198 | ---NA--- |
| 808 | Contig7209 | Thymidylate synthase |
| 809 | Contig7214 | pleckstrin homology domain-containing protein 1-like |
| 810 | Contig7219 | peptidyl-prolyl cis-trans isomerase |
| 811 | Contig7256 | acyl carrier protein 2, mitochondrial-like |
| 812 | Contig7257 | cold-regulated 413 plasma membrane protein 1 |
| 813 | Contig7265 | iron-sulfur cluster assembly protein 1-like |
| 814 | Contig7268 | conserved hypothetical protein |
| 815 | Contig7282 | protein LIGHT-DEPENDENT SHORT HYPOCOTYLS 4-like |
| 816 | Contig7294 | ---NA--- |
| 817 | Contig7322 | probable prolyl 4-hydroxylase 9 |
| 818 | Contig7344 | V-type proton ATPase catalytic subunit A |
| 819 | Contig7376 | probable NAD(P)H dehydrogenase (quinone) FQR1-like 3 isoform X2 |
| 820 | Contig7381 | uncharacterized protein LOC110633375 |
| 821 | Contig7383 | MLP-like protein 423 |
| 822 | Contig7390 | basic leucine zipper and W2 domain-containing protein 2-like |
| 823 | Contig7409 | defensin Ec-AMP-D2-like |
| 824 | Contig7415 | ethylene-responsive transcription factor 4 |
| 825 | Contig7432 | PLASMODESMATA CALLOSE-BINDING PROTEIN 3-like |
| 826 | Contig7442 | uncharacterized protein LOC111007393 |
| 827 | Contig7472 | 40S ribosomal protein S8 |
| 828 | Contig7480 | Aspartic proteinase nepenthesin-1 |
| 829 | Contig7499 | ADP-ribosylation factor 2 |
| 830 | Contig7525 | signal peptidase complex subunit 3B |
| 831 | Contig7529 | putative lipid-transfer protein DIR1 |
| 832 | Contig7532 | citrate synthase, glyoxysomal-like |
| 833 | Contig7543 | 60S ribosomal protein L26-1 |
| 834 | Contig7571 | ---NA--- |
| 835 | Contig7576 | omega-3 fatty acid desaturase |
| 836 | Contig7586 | ---NA--- |
| 837 | Contig7608 | ---NA--- |
| 838 | Contig7616 | ribosomal RNA processing protein 1 homolog |
| 839 | Contig7648 | transcription factor, putative |
| 840 | Contig7652 | high mobility group B protein 3-like |
| 841 | Contig7655 | probable histone H2A.1 |
| 842 | Contig7664 | putative oligosaccharyltransferase complex subunit CG9662 |
| 843 | Contig7665 | auxin-repressed 12.5 kDa protein-like |
| 844 | Contig7687 | 60S ribosomal protein L37a |
| 845 | Contig7688 | ethylene-responsive transcription factor 9-like |
| 846 | Contig7695 | putative zinc finger A20 and AN1 domain-containing stress-associated protein 8 |
| 847 | Contig7708 | 40S ribosomal protein S30 |
| 848 | Contig7733 | metallothionein-like protein type 2 |
| 849 | Contig7742 | 30S ribosomal protein S5, chloroplastic-like |
| 850 | Contig7770 | photosystem II 5 kDa protein, chloroplastic-like |
| 851 | Contig7788 | ---NA--- |
| 852 | Contig7811 | PREDICTED: uncharacterized protein LOC104825545 |
| 853 | Contig7851 | ---NA--- |
| 854 | Contig7852 | Alpha-crystallin, subunit A |
| 855 | Contig7863 | photosystem I reaction center subunit II, chloroplastic-like |
| 856 | Contig7878 | BRISC and BRCA1-A complex member 2-like isoform X2 |
| 857 | Contig7918 | stem-specific protein TSJT1-like |
| 858 | Contig7944 | nucleoside diphosphate kinase 2, chloroplastic |
| 859 | Contig7945 | stem-specific protein TSJT1-like |
| 860 | Contig7955 | cytosolic glyceraldehyde-3-phosophodehydrogenase |
| 861 | Contig7971 | cold-regulated protein, putative |
| 862 | Contig7972 | protein SGT1 homolog B-like |
| 863 | Contig7992 | ---NA--- |
| 864 | Contig8010 | hypothetical protein JCGZ_21620 |
| 865 | Contig8015 | 40S ribosomal protein S17-like |
| 866 | Contig8016 | Ankyrin repeat family protein, putative isoform 1 |
| 867 | Contig8018 | 60S ribosomal protein L19-3 |
| 868 | Contig8022 | ribulose bisphosphate carboxylase small chain, chloroplastic-like |
| 869 | Contig8043 | ---NA--- |
| 870 | Contig8044 | 14-3-3-like protein A |
| 871 | Contig8049 | major allergen Pru ar 1 |
| 872 | Contig8069 | probable WRKY transcription factor 53 |
| 873 | Contig8102 | Histone H4 |
| 874 | Contig8123 | ---NA--- |
| 875 | Contig8127 | V-type proton ATPase subunit c1 |
| 876 | Contig8133 | ---NA--- |
| 877 | Contig8135 | ubiquitin-conjugating enzyme E2-23 kDa |
| 878 | Contig8139 | legumin B |
| 879 | Contig8170 | ---NA--- |
| 880 | Contig8173 | Chaperone DnaJ 2 |
| 881 | Contig8185 | tobamovirus multiplication protein 2A |
| 882 | Contig8188 | ubiquitin-40S ribosomal protein S27a |
| 883 | Contig8190 | transcription factor TCP11 |
| 884 | Contig8193 | Cysteine proteinase inhibitor 5 |
| 885 | Contig8245 | probable ribonuclease P/MRP protein subunit POP5 |
| 886 | Contig8246 | beta-amylase 3, chloroplastic |
| 887 | Contig8251 | BI1-like protein |
| 888 | Contig8256 | cyclin-dependent kinase E-1-like |
| 889 | Contig8259 | 60S acidic ribosomal protein P2A |
| 890 | Contig8260 | metallothionein-like protein type 3 |
| 891 | Contig8265 | putative cell wall protein |
| 892 | Contig8267 | protein LNK3-like |
| 893 | Contig8274 | Superoxide dismutase |
| 894 | Contig8284 | autophagy-related protein 8f |
| 895 | Contig8292 | cysteine proteinase inhibitor-like |
| 896 | Contig8293 | non-specific lipid-transfer protein 2-like |
| 897 | Contig8297 | legumin B-like precursor |
| 898 | Contig8298 | Photosystem II PsbX |
| 899 | Contig8308 | Thylakoid soluble phosphoprotein TSP9 |
| 900 | Contig8329 | 60S ribosomal protein L35 |
| 901 | Contig8336 | gibberellin-regulated protein 1-like |
| 902 | Contig8363 | ---NA--- |
| 903 | Contig8382 | Lipid transfer protein |
| 904 | Contig8395 | cytochrome P450 76A1-like |
| 905 | Contig8454 | citrate-binding protein-like |
| 906 | Contig8458 | predicted protein |
| 907 | Contig8462 | beta-adaptin-like protein B |
| 908 | Contig8471 | 2S albumin |
| 909 | Contig8477 | 60S ribosomal protein L35a-1 |
| 910 | Contig8491 | ---NA--- |
| 911 | Contig8502 | origin recognition complex subunit 1 isoform X1 |
| 912 | Contig8521 | 60S ribosomal protein L36-2-like |
| 913 | Contig8527 | 60S ribosomal protein L30 |
| 914 | Contig8535 | proteasome subunit alpha type-5 |
| 915 | Contig8551 | ubiquitin-60S ribosomal protein L40 |
| 916 | Contig8566 | probable ADP-ribosylation factor GTPase-activating protein AGD14 isoform X2 |
| 917 | Contig8576 | 2S albumin-like |
| 918 | Contig8586 | cytochrome b5 |
| 919 | Contig8599 | 60S ribosomal protein L27 |
| 920 | Contig8604 | probable calcium-binding protein CML27 |
| 921 | Contig8614 | ---NA--- |
| 922 | Contig8620 | vacuolar protein sorting-associated protein 9A-like |
| 923 | Contig8628 | 60S ribosomal protein L3-2 |
| 924 | Contig8655 | 60S ribosomal protein L30 |
| 925 | Contig8668 | nodulin-related protein 1-like |
| 926 | Contig8682 | ---NA--- |
| 927 | Contig8693 | malate synthase, glyoxysomal |
| 928 | Contig8742 | hypothetical protein MANES_01G247900 |
| 929 | Contig8745 | 14 kDa proline-rich protein DC2.15-like |
| 930 | Contig8759 | protein TRIGALACTOSYLDIACYLGLYCEROL 2, chloroplastic |
| 931 | Contig8782 | armadillo/beta-catenin repeat family protein |
| 932 | Contig8813 | syntaxin-52-like |
| 933 | Contig8824 | chlorophyll a-b binding protein 151, chloroplastic |
| 934 | Contig8838 | 60S ribosomal protein L24 |
| 935 | Contig8852 | putative cytochrome c oxidase subunit 5b-like |

***Euphorbia fisheriana***

| **Sl. No.** | **ID** | **Function** |
| --- | --- | --- |
| 1 | Contig3 | ---NA--- |
| 2 | Contig5 | Elongation factor 1-beta 1 |
| 3 | Contig8 | ubiquitin receptor RAD23c-like isoform X2 |
| 4 | Contig23 | elongation factor 1-alpha |
| 5 | Contig29 | dnaJ protein homolog |
| 6 | Contig33 | 60S ribosomal protein L3-2 |
| 7 | Contig68 | tubulin beta-4 chain |
| 8 | Contig74 | yth domain-containing protein, putative |
| 9 | Contig82 | protein NUCLEAR FUSION DEFECTIVE 4-like |
| 10 | Contig134 | phytanoyl-CoA dioxygenase |
| 11 | Contig149 | polyubiquitin 3 |
| 12 | Contig151 | FRIGIDA-like protein 4a |
| 13 | Contig155 | elongation factor 1-gamma-like |
| 14 | Contig165 | putative quinone-oxidoreductase homolog, chloroplastic |
| 15 | Contig169 | Polyadenylate-binding protein 2 |

***Hevea brasiliensis***

| **Sl. No.** | **ID** | **Function** |
| --- | --- | --- |
| 1 | Contig5 | probable aquaporin PIP2-8 |
| 2 | Contig13 | ribulose bisphosphate carboxylase small chain, chloroplastic-like |
| 3 | Contig19 | eukaryotic translation initiation factor 5A |
| 4 | Contig47 | ---NA--- |
| 5 | Contig60 | auxin-responsive protein SAUR71-like |
| 6 | Contig65 | reticulon-like protein B5 |
| 7 | Contig76 | leucine-rich repeat receptor-like protein kinase PEPR1 |
| 8 | Contig80 | eukaryotic translation initiation factor 5 |
| 9 | Contig82 | vesicle transport v-SNARE 12-like |
| 10 | Contig83 | acyl-coenzyme A oxidase 4, peroxisomal |
| 11 | Contig177 | chorismate synthase, chloroplastic-like |
| 12 | Contig179 | probable glycerol-3-phosphate acyltransferase 8 |
| 13 | Contig185 | profilin-2 |
| 14 | Contig200 | abscisic stress-ripening protein 3-like |
| 15 | Contig209 | rRNA-processing protein EFG1-like isoform X1 |
| 16 | Contig217 | 40S ribosomal protein S16-like |
| 17 | Contig239 | putative disease resistance protein RGA3 isoform X1 |
| 18 | Contig244 | fimbrin-5 |
| 19 | Contig253 | PEMT domain-containing protein |
| 20 | Contig262 | uncharacterized protein LOC110652228 isoform X1 |
| 21 | Contig278 | 60S ribosomal protein L19-2 |
| 22 | Contig298 | Transmembrane protein |
| 23 | Contig306 | bet1-like SNARE 1-1 |
| 24 | Contig310 | uncharacterized protein LOC110660226 |
| 25 | Contig311 | late embryogenesis abundant protein At5g17165-like |
| 26 | Contig316 | uncharacterized protein LOC110654440 isoform X2 |
| 27 | Contig326 | CASP-like protein 5A2 |
| 28 | Contig327 | nuclear transport factor 2 |
| 29 | Contig331 | calcineurin subunit B-like |
| 30 | Contig349 | GTP-binding nuclear protein Ran-3 |
| 31 | Contig360 | translationally-controlled tumor protein homolog |
| 32 | Contig362 | cell number regulator 6-like |
| 33 | Contig372 | polyubiquitin-like |
| 34 | Contig379 | UTP:RNA uridylyltransferase 1 |
| 35 | Contig381 | transcription factor ILR3 |
| 36 | Contig387 | chalcone synthase-like |
| 37 | Contig405 | 60S acidic ribosomal protein P2A-like |
| 38 | Contig409 | 60S ribosomal protein L5 |
| 39 | Contig418 | clathrin interactor EPSIN 3-like isoform X1 |
| 40 | Contig421 | anthocyanidin 3-O-glucosyltransferase 5-like |
| 41 | Contig435 | ubiquitin-conjugating enzyme E2 28 |
| 42 | Contig441 | transmembrane protein 205 |
| 43 | Contig445 | glycine-rich RNA-binding protein-like |
| 44 | Contig466 | probable RNA-binding protein ARP1 isoform X1 |
| 45 | Contig468 | lipase class 3 family protein |
| 46 | Contig469 | protein NBR1 homolog |
| 47 | Contig472 | glyceraldehyde-3-phosphate dehydrogenase, cytosolic-like |
| 48 | Contig476 | protein ALUMINUM SENSITIVE 3 |
| 49 | Contig498 | prefoldin subunit 1 |
| 50 | Contig501 | E3 ubiquitin-protein ligase RNF4 |
| 51 | Contig503 | actin-depolymerizing factor 2 |
| 52 | Contig507 | ADP-ribosylation factor |
| 53 | Contig526 | thioredoxin-like 1-1, chloroplastic |
| 54 | Contig529 | diacylglycerol O-acyltransferase 3, cytosolic |
| 55 | Contig536 | probable RNA helicase SDE3 |
| 56 | Contig550 | transaldolase family protein |
| 57 | Contig554 | dnaJ protein homolog |
| 58 | Contig563 | probable protein phosphatase 2C 34 |
| 59 | Contig576 | natterin-like protein |
| 60 | Contig577 | transcription factor MYB1R1 |
| 61 | Contig591 | cytochrome b-c1 complex subunit Rieske-4, mitochondrial-like |
| 62 | Contig599 | enhancer of rudimentary homolog |
| 63 | Contig600 | SKP1-like protein 1B |
| 64 | Contig602 | polyubiquitin |
| 65 | Contig605 | 40S ribosomal protein S26-3 |
| 66 | Contig612 | protein DEHYDRATION-INDUCED 19 homolog 3-like |
| 67 | Contig621 | T-complex protein 1 subunit gamma |
| 68 | Contig636 | calmodulin-7-like isoform X2 |
| 69 | Contig639 | serine/threonine-protein kinase HT1-like |
| 70 | Contig640 | rop guanine nucleotide exchange factor 1 |
| 71 | Contig653 | thioredoxin H-type |
| 72 | Contig654 | heterogeneous nuclear ribonucleoprotein 1 |
| 73 | Contig658 | SWI/SNF complex component SNF12 homolog |
| 74 | Contig666 | 60S ribosomal protein L3 |
| 75 | Contig668 | ---NA--- |
| 76 | Contig680 | transcription initiation factor TFIID subunit 10 |
| 77 | Contig683 | ferredoxin, root R-B2-like |
| 78 | Contig688 | probable E3 ubiquitin-protein ligase XERICO |
| 79 | Contig697 | serine/threonine-protein kinase AtPK2/AtPK19-like |
| 80 | Contig698 | outer envelope pore protein 16-2, chloroplastic |
| 81 | Contig708 | beta-1,3-glucanase |
| 82 | Contig716 | peptidyl-prolyl cis-trans isomerase FKBP12 |
| 83 | Contig723 | uncharacterized protein LOC110633195 |
| 84 | Contig732 | thioredoxin H-type |
| 85 | Contig733 | uncharacterized protein LOC110639359 isoform X1 |
| 86 | Contig744 | GDP-L-galactose phosphorylase 2-like |
| 87 | Contig746 | eukaryotic initiation factor 4A-8 |
| 88 | Contig780 | chalcone synthase-like |
| 89 | Contig782 | NHP2-like protein 1 |
| 90 | Contig783 | histone H3.3 isoform X1 |
| 91 | Contig784 | embryo defective |
| 92 | Contig812 | 3-ketoacyl-CoA synthase 1 |
| 93 | Contig814 | MFP1 attachment factor 1-like |
| 94 | Contig821 | pro-hevein-like |
| 95 | Contig824 | proteinase inhibitor |
| 96 | Contig833 | nucleolar protein 16 |
| 97 | Contig861 | hypothetical protein 28 |
| 98 | Contig889 | uncharacterized protein LOC110654385 |
| 99 | Contig894 | NADH:ubiquinone oxidoreductase, ESSS subunit |
| 100 | Contig896 | nucleosome assembly protein 1;2-like isoform X1 |
| 101 | Contig916 | protein argonaute 5-like |
| 102 | Contig922 | protein LURP-one-related 5-like |
| 103 | Contig933 | protein ETHYLENE INSENSITIVE 3-like |
| 104 | Contig938 | KH domain-containing protein At1g09660/At1g09670-like isoform X1 |
| 105 | Contig943 | BTB/POZ domain-containing protein At1g21780 |
| 106 | Contig958 | myosin-binding protein 7 |
| 107 | Contig968 | copper methylamine oxidase-like |
| 108 | Contig983 | superoxide dismutase [Mn], mitochondrial |
| 109 | Contig988 | 40S ribosomal protein S24-1 |
| 110 | Contig992 | protein NBR1 homolog isoform X1 |
| 111 | Contig995 | cytochrome P450 90A1 |
| 112 | Contig997 | NADH dehydrogenase [ubiquinone] 1 beta subcomplex subunit 7 |
| 113 | Contig1001 | ADP-ribosylation factor 1 |
| 114 | Contig1014 | 40S ribosomal protein S4-1 |
| 115 | Contig1021 | 17.3 kDa class I heat shock protein-like |
| 116 | Contig1031 | protein LIGHT-DEPENDENT SHORT HYPOCOTYLS 4-like |
| 117 | Contig1036 | peptidyl-prolyl cis-trans isomerase |
| 118 | Contig1037 | GLABROUS1 enhancer-binding protein-like |
| 119 | Contig1045 | copper-transporting ATPase PAA1, chloroplastic-like isoform X2 |
| 120 | Contig1046 | 40S ribosomal protein S23 |
| 121 | Contig1060 | protein eyes shut |
| 122 | Contig1071 | uncharacterized LOC8263958 |
| 123 | Contig1072 | uncharacterized protein LOC110645350 |
| 124 | Contig1074 | ubiquitin-conjugating enzyme E2 36 |
| 125 | Contig1095 | NAC domain-containing protein 73-like |
| 126 | Contig1096 | biogenesis of lysosome-related organelles complex 1 subunit 2 |
| 127 | Contig1101 | acyl-CoA-binding protein |
| 128 | Contig1106 | 60S ribosomal protein L10 |
| 129 | Contig1114 | 60S ribosomal protein L24 |
| 130 | Contig1137 | ubiquitin-40S ribosomal protein S27a |
| 131 | Contig1138 | B-box zinc finger protein 21-like |
| 132 | Contig1140 | cis-prenyltransferase |
| 133 | Contig1183 | 40S ribosomal protein S27-2 |
| 134 | Contig1198 | protein translation factor SUI1 homolog 2-like |
| 135 | Contig1199 | reticulon-like protein B2 |
| 136 | Contig1219 | NAC domain-containing protein 83-like |
| 137 | Contig1222 | ran-binding protein 1 homolog b-like |
| 138 | Contig1234 | acyl carrier protein 1, chloroplastic-like |
| 139 | Contig1237 | PHD finger protein ALFIN-LIKE 4-like |
| 140 | Contig1240 | chalcone synthase-like |
| 141 | Contig1241 | probable RNA-binding protein ARP1 isoform X2 |
| 142 | Contig1244 | F17A17.10 protein |
| 143 | Contig1256 | DNA-binding protein DDB_G0278111 isoform X1 |
| 144 | Contig1264 | protein argonaute 1 |
| 145 | Contig1267 | ras-related protein RABF2b |
| 146 | Contig1268 | protein IQ-DOMAIN 1 |
| 147 | Contig1278 | putative G3BP-like protein |
| 148 | Contig1285 | ADP-ribosylation factor |
| 149 | Contig1296 | elongation factor 1-alpha-like |
| 150 | Contig1311 | homeobox-leucine zipper protein HOX11-like |
| 151 | Contig1312 | ubiquitin-conjugating enzyme E2 7 |
| 152 | Contig1323 | 60S ribosomal protein L31 |
| 153 | Contig1326 | zinc finger A20 and AN1 domain-containing stress-associated protein 5-like |
| 154 | Contig1331 | nuclear transcription factor Y subunit B-1-like isoform X1 |
| 155 | Contig1336 | pyroglutamyl-peptidase 1-like |
| 156 | Contig1349 | hypothetical protein MANES_18G041800 |
| 157 | Contig1356 | elongation factor 1-gamma |
| 158 | Contig1374 | metacaspase-4 |
| 159 | Contig1376 | HEVE_HEVBRRecName: Full=Pro-hevein; AltName: Full=Major hevein; Contains: RecName: Full=Hevein; AltName: Allergen=Hev b 6; Contains: RecName: Full=Win-like protein; Flags: Precursor |
| 160 | Contig1381 | transcription factor MYC2-like |
| 161 | Contig1384 | DNA replication complex GINS protein PSF2 |
| 162 | Contig1397 | signal peptidase complex catalytic subunit SEC11A-like |
| 163 | Contig1398 | polyadenylate-binding protein 2-like |
| 164 | Contig1408 | protein LIGHT-DEPENDENT SHORT HYPOCOTYLS 10-like |
| 165 | Contig1410 | transcription factor MafB-like |
| 166 | Contig1414 | NDR1/HIN1-like protein 13 |
| 167 | Contig1418 | triphosphate tunel metalloenzyme 3-like |
| 168 | Contig1420 | nuclear transcription factor Y subunit C-9-like |
| 169 | Contig1421 | eukaryotic translation initiation factor 4 gamma-like |
| 170 | Contig1424 | kinesin-like protein KIN-14T |
| 171 | Contig1434 | F-box/WD repeat-containing protein 4 |
| 172 | Contig1441 | mannan endo-1,4-beta-mannosidase 7-like |
| 173 | Contig1443 | ubiquitin-conjugating enzyme e2 2 |
| 174 | Contig1447 | HEVE_HEVBRRecName: Full=Pro-hevein; AltName: Full=Major hevein; Contains: RecName: Full=Hevein; AltName: Allergen=Hev b 6; Contains: RecName: Full=Win-like protein; Flags: Precursor |
| 175 | Contig1449 | thiamine thiazole synthase, chloroplastic |
| 176 | Contig1451 | 60S acidic ribosomal protein P0 |
| 177 | Contig1468 | salt stress-induced hydrophobic peptide ESI3 |
| 178 | Contig1474 | serine protease inhibitor |
| 179 | Contig1486 | 50S ribosomal protein L7/L12 |
| 180 | Contig1491 | 3'-5' exoribonuclease 1-like |
| 181 | Contig1494 | ---NA--- |
| 182 | Contig1499 | rubber cis-polyprenyltransferase HRT2-like |
| 183 | Contig1507 | malate dehydrogenase |
| 184 | Contig1514 | uncharacterized protein LOC110668936 |
| 185 | Contig1515 | reticulon-like protein B2 |
| 186 | Contig1526 | fimbrin-5 |
| 187 | Contig1550 | ubiquitin-conjugating enzyme e2 2 |
| 188 | Contig1553 | 60S ribosomal protein L10a-2 |
| 189 | Contig1581 | 40S ribosomal protein S28-2 |
| 190 | Contig1608 | FRIGIDA-like protein 4a |
| 191 | Contig1612 | 50S ribosomal protein L20 |
| 192 | Contig1614 | putative E3 ubiquitin-protein ligase XBAT35 isoform X1 |
| 193 | Contig1636 | NC domain-containing family protein |
| 194 | Contig1637 | eukaryotic translation initiation factor 3 subunit J-like |
| 195 | Contig1640 | 60s acidic ribosomal protein p2b |
| 196 | Contig1661 | zinc finger CCCH domain-containing protein 20-like |
| 197 | Contig1662 | ubiquitin-60S ribosomal protein L40 |
| 198 | Contig1679 | uncharacterized protein LOC110648045 |
| 199 | Contig1687 | 60S ribosomal protein L26-1 |
| 200 | Contig1691 | homeobox-leucine zipper protein HAT22-like |
| 201 | Contig1696 | wound-induced basic protein |
| 202 | Contig1706 | acyl carrier protein 1, chloroplastic-like |
| 203 | Contig1707 | 60S ribosomal protein L13a-4 |
| 204 | Contig1711 | cytochrome b5-like |
| 205 | Contig1713 | dnaJ protein homolog |
| 206 | Contig1718 | LOB domain-containing protein 4-like |
| 207 | Contig1727 | putative e3 ubiquitin-protein ligase rbra |
| 208 | Contig1728 | E3 ubiquitin-protein ligase XBAT32 isoform X1 |
| 209 | Contig1747 | ---NA--- |
| 210 | Contig1753 | 18.5 kDa class IV heat shock protein |
| 211 | Contig1755 | uncharacterized protein LOC110669829 |
| 212 | Contig1760 | 3-ketoacyl-CoA synthase 11 |
| 213 | Contig1764 | nodulin-related protein 1-like |
| 214 | Contig1767 | glutamine synthetase |
| 215 | Contig1769 | 60S ribosomal protein L30 |
| 216 | Contig1772 | Werner Syndrome-like exonuclease |
| 217 | Contig1802 | O-acyltransferase WSD1-like |
| 218 | Contig1810 | nucleolar protein 10 |
| 219 | Contig1824 | 40S ribosomal protein S5 |
| 220 | Contig1826 | V-type proton ATPase subunit F |
| 221 | Contig1827 | copper methylamine oxidase |
| 222 | Contig1841 | 60S ribosomal protein L7a-1 |
| 223 | Contig1858 | probable polyamine oxidase 2 |
| 224 | Contig1866 | peptidyl-prolyl cis-trans isomerase FKBP15-1 |
| 225 | Contig1880 | guanine nucleotide-binding protein subunit beta-like protein |
| 226 | Contig1903 | CS domain |
| 227 | Contig1904 | heavy metal-associated isoprenylated plant protein 20-like |
| 228 | Contig1917 | protein phosphatase 2C 16-like |
| 229 | Contig1932 | 40S ribosomal protein S17-like |
| 230 | Contig1953 | polyubiquitin |
| 231 | Contig1968 | EG964 |
| 232 | Contig1971 | 3-hydroxy-3-methylglutaryl-coenzyme A reductase 1 |
| 233 | Contig1972 | heat shock factor protein HSF24 |
| 234 | Contig1977 | aspartic proteinase A1-like |
| 235 | Contig1978 | UDP-glycosyltransferase 74B1-like |
| 236 | Contig1979 | salt stress-induced hydrophobic peptide ESI3 |
| 237 | Contig1981 | dynamin-related protein 4C-like |
| 238 | Contig1982 | cAMP-binding protein 1-like |
| 239 | Contig1986 | glycine-rich RNA-binding protein 2, mitochondrial-like |
| 240 | Contig2013 | chalcone synthase-like |
| 241 | Contig2022 | homeobox-leucine zipper protein HAT22-like |
| 242 | Contig2032 | iron-sulfur cluster assembly protein 1-like |
| 243 | Contig2038 | calcyclin-binding protein-like |
| 244 | Contig2042 | phosphoinositide phospholipase C 2 |
| 245 | Contig2046 | lanC-like protein GCR2 |
| 246 | Contig2051 | putative Myb family transcription factor At1g14600 |
| 247 | Contig2074 | ubiquitin-conjugating enzyme E2-17 kDa |
| 248 | Contig2078 | lanC-like protein GCL1 |
| 249 | Contig2084 | transcription factor MYC2-like |
| 250 | Contig2094 | protein LIKE COV 2 |
| 251 | Contig2100 | acylphosphatase, putative |
| 252 | Contig2109 | probable alpha-amylase 2 |
| 253 | Contig2111 | serine/threonine-protein kinase HT1-like |
| 254 | Contig2119 | triacylglycerol lipase, putative |
| 255 | Contig2129 | protein BUD31 homolog 1 |
| 256 | Contig2142 | nascent polypeptide-associated complex subunit alpha-like protein 1 |
| 257 | Contig2154 | cell number regulator 8-like |
| 258 | Contig2155 | Nucleic acid-binding protein |
| 259 | Contig2171 | ATP synthase delta chain, chloroplastic |
| 260 | Contig2174 | Nicotiana HR lesion-inducing family protein |
| 261 | Contig2178 | bifunctional dihydroflavonol 4-reductase/flavanone 4-reductase-like |
| 262 | Contig2193 | hydrophobic protein RCI2B |
| 263 | Contig2194 | probable terpene synthase 8 |
| 264 | Contig2207 | diphosphomevalonate decarboxylase |
| 265 | Contig2215 | translationally-controlled tumor protein homolog |
| 266 | Contig2220 | transmembrane protein 230-like |
| 267 | Contig2234 | senescence-associated family protein |
| 268 | Contig2237 | probable E3 ubiquitin-protein ligase XERICO |
| 269 | Contig2239 | putative F-box protein PP2-B12 |
| 270 | Contig2243 | actin-depolymerizing factor |
| 271 | Contig2251 | dynamin-related protein 4C-like |
| 272 | Contig2256 | 60S ribosomal protein L7-2-like |
| 273 | Contig2272 | ethylene-responsive transcription factor RAP2-12-like |
| 274 | Contig2280 | protein YIF1B |
| 275 | Contig2282 | ribosomal RNA small subunit methyltransferase nep-1 |
| 276 | Contig2283 | 60S ribosomal protein L7a-1-like |
| 277 | Contig2297 | transmembrane protein, putative |
| 278 | Contig2304 | protein translation factor SUI1 homolog 2-like |
| 279 | Contig2313 | protein indeterminate-domain 2-like |
| 280 | Contig2326 | probable glycerol-3-phosphate acyltransferase 8 |
| 281 | Contig2328 | glutaredoxin-C6-like |
| 282 | Contig2331 | uncharacterized protein LOC110647971 |
| 283 | Contig2339 | DUF789 domain-containing protein |
| 284 | Contig2343 | ---NA--- |
| 285 | Contig2345 | nudC domain-containing protein 2 |
| 286 | Contig2355 | putative ubiquitin |
| 287 | Contig2364 | vacuolar-sorting receptor 1-like |
| 288 | Contig2368 | ras-related protein RABA1f |
| 289 | Contig2374 | pyrophosphatase |
| 290 | Contig2384 | glutamic acid-rich protein-like isoform X2 |
| 291 | Contig2394 | uncharacterized protein LOC110631735 |
| 292 | Contig2396 | cucumber peeling cupredoxin-like |
| 293 | Contig2398 | hypothetical protein MANES_08G126300 |
| 294 | Contig2404 | 60S ribosomal protein L19-2 |
| 295 | Contig2411 | 40S ribosomal protein S19-3 |
| 296 | Contig2420 | protein transport protein Sec61 subunit beta |
| 297 | Contig2423 | ubiquitin-60S ribosomal protein L40 |
| 298 | Contig2428 | probable (S)-N-methylcoclaurine 3'-hydroxylase isozyme 2 |
| 299 | Contig2432 | ricin B-like lectin R40G3 |
| 300 | Contig2434 | uncharacterized protein LOC110663049 |
| 301 | Contig2440 | cytochrome P450 89A2-like |
| 302 | Contig2463 | 60S ribosomal protein L6 |
| 303 | Contig2474 | putative G3BP-like protein |
| 304 | Contig2477 | LYR motif protein |
| 305 | Contig2478 | actin T1-like protein |
| 306 | Contig2481 | trehalose-phosphate phosphatase A |
| 307 | Contig2483 | cell division topological specificity factor homolog, chloroplastic |
| 308 | Contig2494 | histone H3.3 isoform X1 |
| 309 | Contig2506 | 60S ribosomal protein L18-2 |
| 310 | Contig2508 | molybdenum cofactor sulfurase |
| 311 | Contig2529 | 60S ribosomal protein L21-1-like |
| 312 | Contig2544 | MADS-box protein SOC1 isoform X1 |
| 313 | Contig2546 | histone H1-like |
| 314 | Contig2552 | FRIGIDA-like protein 4a |
| 315 | Contig2560 | uncharacterized protein LOC110660811 |
| 316 | Contig2570 | wound-induced protein 1-like |
| 317 | Contig2610 | UDP-glycosyltransferase 74B1-like |
| 318 | Contig2612 | latex abundant protein 1 |
| 319 | Contig2616 | enhancer of rudimentary homolog |
| 320 | Contig2634 | probable calcium-binding protein CML27 |
| 321 | Contig2635 | transcription factor IBH1-like |
| 322 | Contig2643 | fructose-bisphosphate aldolase 6, cytosolic |
| 323 | Contig2657 | dnaJ protein homolog |
| 324 | Contig2658 | repetitive proline-rich cell wall protein 2-like |
| 325 | Contig2663 | probable phospholipid hydroperoxide glutathione peroxidase |
| 326 | Contig2678 | high mobility group B protein 1 |
| 327 | Contig2680 | ---NA--- |
| 328 | Contig2689 | defensin-like protein |
| 329 | Contig2695 | probable aquaporin PIP1-2 |
| 330 | Contig2698 | 60S ribosomal protein L17-2 |
| 331 | Contig2700 | DNA-binding protein S1FA-like |
| 332 | Contig2701 | abscisic stress-ripening protein 1-like |
| 333 | Contig2704 | ---NA--- |
| 334 | Contig2730 | glutathione S-transferase F9-like |
| 335 | Contig2731 | TOM1-like protein 2 isoform X1 |
| 336 | Contig2733 | 40S ribosomal protein S6 |
| 337 | Contig2750 | homeobox-DDT domain protein RLT1-like isoform X5 |
| 338 | Contig2754 | ---NA--- |
| 339 | Contig2760 | thioredoxin H-type |
| 340 | Contig2766 | protein TIFY 10A |
| 341 | Contig2775 | caffeic acid 3-O-methyltransferase-like |
| 342 | Contig2776 | uncharacterized protein At5g39865-like |
| 343 | Contig2777 | UDP-arabinopyranose mutase 1-like isoform X2 |
| 344 | Contig2779 | UDP-D-apiose/UDP-D-xylose synthase 2 |
| 345 | Contig2783 | polyadenylate-binding protein RBP47-like isoform X2 |
| 346 | Contig2794 | cullin-3A |
| 347 | Contig2817 | hypothetical protein JCGZ_10888 |
| 348 | Contig2826 | profilin |
| 349 | Contig2837 | deSI-like protein At4g17486 isoform X1 |
| 350 | Contig2844 | uncharacterized protein LOC110631595 |
| 351 | Contig2857 | uncharacterized protein LOC110647619 |
| 352 | Contig2866 | actin-depolymerizing factor 2 |
| 353 | Contig2878 | 60S ribosomal protein L11 |
| 354 | Contig2895 | ras-related protein RABB1c |
| 355 | Contig2903 | GDSL esterase/lipase-like |
| 356 | Contig2909 | diphthamide biosynthesis protein 3-like |
| 357 | Contig2916 | S-adenosylmethionine decarboxylase proenzyme |
| 358 | Contig2922 | hypothetical protein 21 |
| 359 | Contig2944 | probable RNA-binding protein ARP1 isoform X2 |
| 360 | Contig2954 | 18.2 kda class i heat shock protein |
| 361 | Contig2956 | hevamine-A |
| 362 | Contig2959 | ethylene-responsive transcription factor ERF016-like |
| 363 | Contig2963 | histone H4 |
| 364 | Contig2980 | 60S ribosomal protein L23a-like |
| 365 | Contig2981 | transcription elongation factor B polypeptide 1 |
| 366 | Contig2988 | voltage-dependent L-type calcium channel subunit |
| 367 | Contig2994 | adenylosuccinate synthetase 2, chloroplastic |
| 368 | Contig3002 | glutaredoxin |
| 369 | Contig3023 | protein EARLY-RESPONSIVE TO DEHYDRATION 7, chloroplastic-like |
| 370 | Contig3034 |  |
| 371 | Contig3037 | actin-depolymerizing factor 2-like |
| 372 | Contig3044 | ubiquitin-conjugating enzyme E2 variant 1D |
| 373 | Contig3047 | eukaryotic initiation factor 4A-8 |
| 374 | Contig3051 | zinc finger protein ZAT4-like |
| 375 | Contig3064 | sphingolipid delta(4)-desaturase DES1-like |
| 376 | Contig3084 | 40S ribosomal protein S25-2 |
| 377 | Contig3086 | ubiquitin-conjugating enzyme E2 28 |
| 378 | Contig3093 | NADPH-dependent aldehyde reductase 1, chloroplastic-like |
| 379 | Contig3094 | uncharacterized protein LOC110653263 |
| 380 | Contig3103 | stress enhanced protein 2, chloroplastic |
| 381 | Contig3105 | protein indeterminate-domain 12-like |
| 382 | Contig3109 | probable RNA helicase SDE3 isoform X3 |
| 383 | Contig3110 | peroxiredoxin family protein |
| 384 | Contig3127 | arginase 1, mitochondrial |
| 385 | Contig3130 | elongation factor 1-alpha-like |
| 386 | Contig3153 | membrane steroid-binding protein 2-like |
| 387 | Contig3185 | NADH dehydrogenase [ubiquinone] 1 beta subcomplex subunit 3-B-like |
| 388 | Contig3191 | high mobility group B protein 2-like |
| 389 | Contig3195 | histone H1 |
| 390 | Contig3198 | polyol transporter 5 |
| 391 | Contig3202 | putative potassium transporter 12 |
| 392 | Contig3207 | protein yippee-like |
| 393 | Contig3231 | uncharacterized protein LOC110666235 |
| 394 | Contig3232 | lesion inducing family protein |
| 395 | Contig3245 | abscisic acid receptor PYL4-like |
| 396 | Contig3249 | patatin-like protein 2 |
| 397 | Contig3274 | transcription factor MYC2-like |
| 398 | Contig3275 | Aspartate/glutamate/uridylate kinase |
| 399 | Contig3276 | 40S ribosomal protein S17-3-like |
| 400 | Contig3281 | NEDD8-conjugating enzyme Ubc12 |
| 401 | Contig3301 | binding partner of ACD11 1-like |
| 402 | Contig3310 | HEVE_HEVBRRecName: Full=Pro-hevein; AltName: Full=Major hevein; Contains: RecName: Full=Hevein; AltName: Allergen=Hev b 6; Contains: RecName: Full=Win-like protein; Flags: Precursor |
| 403 | Contig3336 | ATP synthase subunit epsilon, mitochondrial |
| 404 | Contig3347 | 40S ribosomal protein S21-2 |
| 405 | Contig3369 | uncharacterized protein LOC110653161 |
| 406 | Contig3370 | protein transport protein YOS1-like |
| 407 | Contig3390 | isoflavone reductase-like protein |
| 408 | Contig3397 | protein LIGHT-DEPENDENT SHORT HYPOCOTYLS 7-like |
| 409 | Contig3420 | uncharacterized protein LOC110646426 isoform X2 |
| 410 | Contig3430 | putative ubiquitin |
| 411 | Contig3435 | protein YIPF1 homolog |
| 412 | Contig3436 | copper transport protein ATX1-like |
| 413 | Contig3439 | eukaryotic translation initiation factor 3 subunit I-like |
| 414 | Contig3457 | ---NA--- |
| 415 | Contig3461 | triosephosphate isomerase, cytosolic |
| 416 | Contig3471 | solanesyl diphosphate synthase 3, chloroplastic/mitochondrial-like isoform X1 |
| 417 | Contig3472 | REF/SRPP-like protein At3g05500 |
| 418 | Contig3483 | myb family transcription factor PHL7-like isoform X1 |
| 419 | Contig3494 | vacuolar protein sorting-associated protein 32 homolog 2 |
| 420 | Contig3502 | ---NA--- |
| 421 | Contig3503 | dolichyl-phosphate beta-glucosyltransferase |
| 422 | Contig3514 | conserved hypothetical protein 12 |
| 423 | Contig3523 | eukaryotic translation initiation factor 3 subunit F-like |
| 424 | Contig3548 | copper-transporting ATPase PAA1, chloroplastic-like isoform X2 |
| 425 | Contig3556 | ASR-like protein 2 |
| 426 | Contig3557 | rubber elongation factor |
| 427 | Contig3558 | probable E3 ubiquitin-protein ligase XERICO |
| 428 | Contig3561 | abscisic stress-ripening protein 3-like |
| 429 | Contig3566 | REF/SRPP-like protein At3g05500 |
| 430 | Contig3567 | ---NA--- |
| 431 | Contig3569 | cyclin-dependent protein kinase inhibitor SMR2-like |
| 432 | Contig3571 | ---NA--- |
| 433 | Contig3572 | REF/SRPP-like protein At3g05500 |
| 434 | Contig3574 | REF/SRPP-like protein At3g05500 |

***Jatropha curcus***

| **Sl. No.** | **ID** | **Function** |
| --- | --- | --- |
| 1 | Contig3 | Regulator of rDNA transcription protein 15 |
| 2 | Contig9 | bidirectional sugar transporter SWEET1-like |
| 3 | Contig21 | enolase |
| 4 | Contig25 | polyadenylate-binding protein-interacting protein 7 |
| 5 | Contig36 | superoxide dismutase [Cu-Zn]-like |
| 6 | Contig50 | probable metal-nicotianamine transporter YSL6 |
| 7 | Contig58 | probable nucleoredoxin 2 |
| 8 | Contig74 | uncharacterized protein LOC105650678 |
| 9 | Contig79 | hypothetical protein JCGZ_16091 |
| 10 | Contig84 | 10 kDa chaperonin, mitochondrial-like |
| 11 | Contig94 | probable protein phosphatase 2C 28 isoform X1 |
| 12 | Contig124 | Regulator of rDNA transcription protein 15 |
| 13 | Contig146 | vicilin-like antimicrobial peptides 2-2 |
| 14 | Contig151 | nucleosome assembly protein 1;2-like isoform X1 |
| 15 | Contig158 | formimidoyltransferase-cyclodeaminase isoform X3 |
| 16 | Contig203 | cAMP-binding protein 1-like |
| 17 | Contig208 | N-terminal acetyltransferase A, auxiliary subunit |
| 18 | Contig228 | sucrose synthase 2 |
| 19 | Contig301 | glyceraldehyde-3-phosphate dehydrogenase, cytosolic |
| 20 | Contig308 | probable aquaporin PIP2-8 |
| 21 | Contig313 | ankyrin repeat-containing protein At5g02620-like |
| 22 | Contig319 | aldehyde dehydrogenase family 3 member F1 |
| 23 | Contig332 | basic leucine zipper and W2 domain-containing protein 2 |
| 24 | Contig353 | acyl-coenzyme A oxidase 4, peroxisomal |
| 25 | Contig369 | 40S ribosomal protein S17-like |
| 26 | Contig373 | dehydrin ERD14-like |
| 27 | Contig375 | ---NA--- |
| 28 | Contig381 | protein usf |
| 29 | Contig435 | Glycoside hydrolase |
| 30 | Contig443 | thaumatin-like protein |
| 31 | Contig474 | probable beta-D-xylosidase 5 |
| 32 | Contig490 | probable nucleoredoxin 2 |
| 33 | Contig499 | glutathione S-transferase T1-like |
| 34 | Contig500 | CUE domain-containing protein |
| 35 | Contig509 | ubiquitin-conjugating enzyme E2 28 |
| 36 | Contig512 | ---NA--- |
| 37 | Contig513 | bark storage protein A |
| 38 | Contig546 | delta(24)-sterol reductase |
| 39 | Contig564 | bark storage protein A-like |
| 40 | Contig584 | 3-ketoacyl-CoA thiolase 2, peroxisomal |
| 41 | Contig652 | early nodulin-75 |
| 42 | Contig693 | fructose-1,6-bisphosphatase, cytosolic |
| 43 | Contig699 | BURP domain-containing protein 17 isoform X1 |
| 44 | Contig716 | exopolygalacturonase clone GBGE184 |
| 45 | Contig728 | 2,3-bisphosphoglycerate-independent phosphoglycerate mutase |
| 46 | Contig768 | mannose-1-phosphate guanylyltransferase 1 |
| 47 | Contig779 | 60S ribosomal protein L19-3 |
| 48 | Contig801 | uncharacterized protein LOC105646703 |
| 49 | Contig811 | chlorophyll a-b binding protein of LHCII type 1 |
| 50 | Contig826 | 40S ribosomal protein SA-like |
| 51 | Contig839 | bifunctional epoxide hydrolase 2-like |
| 52 | Contig865 | probable low-specificity L-threonine aldolase 1 |
| 53 | Contig876 | proteinase inhibitor |
| 54 | Contig901 | polyadenylate-binding protein 2-like |
| 55 | Contig906 | actin-7 |
| 56 | Contig920 | non-specific lipid-transfer protein 1-like |
| 57 | Contig926 | inhibitor of trypsin and hageman factor |
| 58 | Contig939 | polyubiquitin |
| 59 | Contig945 | bark storage protein A |
| 60 | Contig947 | exopolygalacturonase-like |
| 61 | Contig961 | proteinase inhibitor |
| 62 | Contig964 | 60S ribosomal protein L8 |
| 63 | Contig972 | probable galactinol--sucrose galactosyltransferase 2 |
| 64 | Contig980 | ankyrin repeat domain-containing protein 2-like |
| 65 | Contig987 | ankyrin repeat domain-containing protein 2B-like |
| 66 | Contig988 | specific tissue protein |
| 67 | Contig1019 | probable enoyl-CoA hydratase 2, mitochondrial |
| 68 | Contig1020 | protein EXORDIUM-like 2 |
| 69 | Contig1024 | abscisic stress-ripening protein 2-like |
| 70 | Contig1042 | germin-like protein subfamily 1 member 13 |
| 71 | Contig1069 | dehydrodolichyl diphosphate synthase 6 |
| 72 | Contig1081 | L-ascorbate peroxidase, cytosolic |
| 73 | Contig1090 | elongation factor 2 |
| 74 | Contig1096 | ubiquitin carboxyl-terminal hydrolase 23 |
| 75 | Contig1099 | Ubiquitin-40S ribosomal protein S27a |
| 76 | Contig1107 | CCR4-NOT transcription complex subunit 1 isoform X2 |
| 77 | Contig1114 | 40S ribosomal protein S4-3 |
| 78 | Contig1117 | catalase |
| 79 | Contig1140 | mitochondrial outer membrane protein porin of 36 kDa |
| 80 | Contig1196 | polyubiquitin-like |
| 81 | Contig1199 | mitochondrial pyruvate carrier 4 |
| 82 | Contig1207 | polyubiquitin |
| 83 | Contig1209 | probable inactive receptor kinase At2g26730 |
| 84 | Contig1213 | eukaryotic initiation factor 4A-8 |
| 85 | Contig1255 | aquaporin PIP2-4-like |
| 86 | Contig1269 | ER membrane protein complex subunit 10 |
| 87 | Contig1279 | cysteine proteinase inhibitor 12 |
| 88 | Contig1329 | mitochondrial amidoxime reducing component 2 |
| 89 | Contig1341 | 26S proteasome non-ATPase regulatory subunit 7 homolog A |
| 90 | Contig1343 | proteasome subunit alpha type-5 |
| 91 | Contig1353 | glycylpeptide N-tetradecanoyltransferase 1 |
| 92 | Contig1379 | protein TRIGALACTOSYLDIACYLGLYCEROL 4, chloroplastic |
| 93 | Contig1390 | zinc finger A20 and AN1 domain-containing stress-associated protein 8-like |
| 94 | Contig1408 | vignain |
| 95 | Contig1413 | probable 26S proteasome non-ATPase regulatory subunit 3 |
| 96 | Contig1414 | histone H1 |
| 97 | Contig1437 | xyloglucan endotransglucosylase/hydrolase protein 9 |
| 98 | Contig1450 | MLP-like protein 423 |
| 99 | Contig1455 | fructose-bisphosphate aldolase 3, chloroplastic |
| 100 | Contig1460 | spermidine synthase 1 |
| 101 | Contig1461 | catalase isozyme 2 |
| 102 | Contig1464 | fructose-bisphosphate aldolase 6, cytosolic |
| 103 | Contig1470 | calcium-dependent protein kinase SK5 |
| 104 | Contig1517 | aspartate aminotransferase, cytoplasmic |
| 105 | Contig1525 | probable galactinol--sucrose galactosyltransferase 2 |
| 106 | Contig1546 | transcription initiation factor IIA large subunit |
| 107 | Contig1549 | purple acid phosphatase 3-like |
| 108 | Contig1560 | protein SRG1-like |
| 109 | Contig1590 | 3-ketoacyl-CoA synthase 6 |
| 110 | Contig1602 | Transmembrane protein |
| 111 | Contig1624 | putative cytochrome c oxidase subunit 5b-like |
| 112 | Contig1629 | gibberellin-regulated protein 4-like |
| 113 | Contig1653 | uncharacterized protein LOC105631864 |
| 114 | Contig1656 | putative septum site-determining protein minD homolog, chloroplastic |
| 115 | Contig1665 | gamma carbonic anhydrase 1, mitochondrial |
| 116 | Contig1668 | hypothetical protein AQUCO_02300079v1 |
| 117 | Contig1678 | 60S ribosomal protein L15-1 |
| 118 | Contig1698 | katanin p60 ATPase-containing subunit A1 |
| 119 | Contig1713 | superoxide dismutase [Cu-Zn], chloroplastic |
| 120 | Contig1745 | signal recognition particle receptor subunit beta-like |
| 121 | Contig1756 | protein TIFY 10A-like |
| 122 | Contig1763 | histone deacetylase HDT1-like |
| 123 | Contig1775 | thiamine thiazole synthase, chloroplastic |
| 124 | Contig1776 | allene oxide synthase |
| 125 | Contig1794 | zinc finger CCCH domain-containing protein 49-like |
| 126 | Contig1802 | ---NA--- |
| 127 | Contig1803 | ras-related protein Rab7 |
| 128 | Contig1805 | patellin-3-like |
| 129 | Contig1826 | Calcium-dependent phosphotriesterase superfamily protein |
| 130 | Contig1846 | probable phospholipid hydroperoxide glutathione peroxidase |
| 131 | Contig1865 | NAC domain-containing protein 72-like |
| 132 | Contig1884 | cytochrome b-c1 complex subunit Rieske-4, mitochondrial-like |
| 133 | Contig1887 | 40S ribosomal protein S8-like |
| 134 | Contig1910 | zeaxanthin epoxidase, chloroplastic-like isoform X1 |
| 135 | Contig1929 | gibberellin 20-oxidase |
| 136 | Contig1936 | pyruvate kinase isozyme A, chloroplastic |
| 137 | Contig1937 | V-type proton ATPase subunit E |
| 138 | Contig1944 | ATPase, alpha/beta subunit, N-terminal |
| 139 | Contig1947 | E3 ubiquitin-protein ligase RING1-like |
| 140 | Contig1973 | aquaporin TIP2-1 |
| 141 | Contig1994 | casein kinase II subunit alpha-2 |
| 142 | Contig1998 | 40S ribosomal protein S7 |
| 143 | Contig2018 | aldehyde oxidase GLOX |
| 144 | Contig2027 | malate dehydrogenase |
| 145 | Contig2029 | zinc finger CCCH domain-containing protein 20-like |
| 146 | Contig2062 | probable xyloglucan endotransglucosylase/hydrolase protein 23 |
| 147 | Contig2094 | 3-oxoacyl-[acyl-carrier-protein] reductase 4-like |
| 148 | Contig2107 | probable ATP synthase 24 kDa subunit, mitochondrial |
| 149 | Contig2120 | inositol-3-phosphate synthase |
| 150 | Contig2156 | lipid transfer protein EARLI 1-like |
| 151 | Contig2160 | 20 kDa chaperonin, chloroplastic-like |
| 152 | Contig2213 | B-box zinc finger protein 22 |
| 153 | Contig2238 | protein NCA1 |
| 154 | Contig2307 | protein transport protein Sec61 subunit beta |
| 155 | Contig2337 | NAD(P)-binding domain containing protein |
| 156 | Contig2374 | 36.4 kda proline-rich protein |
| 157 | Contig2382 | transmembrane 9 superfamily member 1 |
| 158 | Contig2418 | probable flavin-containing monooxygenase 1 |
| 159 | Contig2433 | MLP-like protein 423 |
| 160 | Contig2470 | serine/threonine-protein phosphatase 6 regulatory ankyrin repeat subunit B-like |
| 161 | Contig2479 | RHOMBOID-like protein 2 |
| 162 | Contig2488 | protein MARD1-like |
| 163 | Contig2506 | uncharacterized protein LOC105641009 |
| 164 | Contig2547 | stem-specific protein TSJT1-like |
| 165 | Contig2549 | auxin-repressed 12.5 kDa protein-like |
| 166 | Contig2558 | PLASMODESMATA CALLOSE-BINDING PROTEIN 3-like |
| 167 | Contig2561 | metallothionein-like protein type 2 |
| 168 | Contig2570 | putative pectinesterase/pectinesterase inhibitor 28 |
| 169 | Contig2584 | thiol protease aleurain-like |
| 170 | Contig2586 | lipoamide acyltransferase component of branched-chain alpha-keto acid dehydrogenase complex, mitochondrial |
| 171 | Contig2604 | UDP-rhamnose/UDP-galactose transporter 2-like |
| 172 | Contig2609 | translocon-associated protein subunit alpha |
| 173 | Contig2624 | ethylene-responsive transcription factor RAP2-12-like |
| 174 | Contig2632 | hypothetical protein JCGZ_02798 |
| 175 | Contig2635 | major allergen Pru ar 1-like |
| 176 | Contig2636 | shaggy-related protein kinase eta |
| 177 | Contig2637 | protein canopy-1 isoform X1 |
| 178 | Contig2641 | aquaporin PIP1-3 |
| 179 | Contig2642 | ---NA--- |
| 180 | Contig2650 | Pro-Pol polyprotein |
| 181 | Contig2653 | legumin B |
| 182 | Contig2654 | uncharacterized protein LOC105634316 |
| 183 | Contig2656 | legumin A |
| 184 | Contig2658 | protein PHR1-LIKE 2-like |
| 185 | Contig2667 | orf49 (mitochondrion) |
| 186 | Contig2678 | coatomer subunit epsilon-1 |
| 187 | Contig2679 | thiol protease aleurain-like |
| 188 | Contig2688 | non-specific lipid-transfer protein A-like |
| 189 | Contig2692 | citrate synthase, glyoxysomal |
| 190 | Contig2694 | peroxisomal membrane protein 11C |
| 191 | Contig2705 | tropinone reductase-like 3 |
| 192 | Contig2707 | 40S ribosomal protein S10-1-like |
| 193 | Contig2710 | inosine triphosphate pyrophosphatase |
| 194 | Contig2713 | 40S ribosomal protein S8 |
| 195 | Contig2729 | glutamine synthetase cytosolic isozyme |
| 196 | Contig2739 | annexin-like protein RJ4 isoform X1 |
| 197 | Contig2746 | malate synthase, glyoxysomal |
| 198 | Contig2748 | uncharacterized protein LOC105631865 |
| 199 | Contig2757 | profilin-1 |
| 200 | Contig2759 | xylose isomerase |
| 201 | Contig2761 | probable aquaporin PIP-type 7a |
| 202 | Contig2765 | legumin B-like |
| 203 | Contig2769 | probable isoaspartyl peptidase/L-asparaginase 2 |
| 204 | Contig2770 | lipid transfer-like protein VAS |
| 205 | Contig2771 | indole-3-acetic acid-induced protein ARG2 |
| 206 | Contig2772 | phosphatidylcholine:diacylglycerol cholinephosphotransferase 1-like |
| 207 | Contig2779 | ADP-ribosylation factor |
| 208 | Contig2781 | cytochrome c1-2, heme protein, mitochondrial |
| 209 | Contig2782 | peptidyl-prolyl cis-trans isomerase |
| 210 | Contig2791 | protein CREG1 |
| 211 | Contig2795 | probable aquaporin PIP2-5 |
| 212 | Contig2797 | aspartic proteinase-like |
| 213 | Contig2806 | WD repeat-containing protein VIP3 |
| 214 | Contig2809 | polyubiquitin 10 |
| 215 | Contig2811 | vignain-like |
| 216 | Contig2812 | Octicosapeptide/Phox/Bem1p family protein isoform 1 |
| 217 | Contig2827 | oxalate--CoA ligase-like |
| 218 | Contig2832 | hypothetical protein JCGZ_07827 |
| 219 | Contig2844 | probable cytosolic oligopeptidase A |
| 220 | Contig2848 | non-specific lipid-transfer protein A |
| 221 | Contig2850 | uncharacterized protein LOC105636756 |
| 222 | Contig2863 | S-adenosylmethionine decarboxylase proenzyme-like |
| 223 | Contig2866 | 60S ribosomal protein L15 |
| 224 | Contig2867 | ATP synthase subunit delta', mitochondrial-like |
| 225 | Contig2892 | CUE domain-containing protein |
| 226 | Contig2895 | probable NADH dehydrogenase [ubiquinone] 1 alpha subcomplex subunit 5, mitochondrial |
| 227 | Contig2906 | eukaryotic translation initiation factor 4G |
| 228 | Contig2907 | Copper centre Cu(A) |
| 229 | Contig2908 | ATP synthase subunit gamma, mitochondrial |
| 230 | Contig2914 | reverse transcriptase |
| 231 | Contig2917 | clathrin light chain 1-like |
| 232 | Contig2921 | zinc finger A20 and AN1 domain-containing stress-associated protein 8-like |
| 233 | Contig2925 | putative E3 ubiquitin-protein ligase XBAT31 |
| 234 | Contig2935 | dnaJ protein homolog |
| 235 | Contig2937 | Retrotransposable element Tf2 |
| 236 | Contig2957 | delta(12)-fatty-acid desaturase FAD2 |
| 237 | Contig2958 | JHL23C09.1, partial |
| 238 | Contig2966 | protein FREE1 isoform X1 |
| 239 | Contig2980 | hypothetical protein JCGZ_05373 |
| 240 | Contig2989 | GDSL esterase/lipase At1g09390 |
| 241 | Contig2990 | protein FREE1 isoform X2 |
| 242 | Contig2996 | acyl-ACP thioesterase |
| 243 | Contig2997 | malate dehydrogenase, mitochondrial |
| 244 | Contig3017 | aminopeptidase M1 |
| 245 | Contig3031 | aspartyl protease AED3 |
| 246 | Contig3055 | protein LIKE COV 2 |
| 247 | Contig3066 | clathrin light chain 2-like |
| 248 | Contig3074 | uncharacterized protein LOC105648026 |
| 249 | Contig3077 | protein NYNRIN-like |
| 250 | Contig3088 | protein TIFY 3B-like isoform X2 |
| 251 | Contig3095 | protein translation factor SUI1 homolog 2-like |
| 252 | Contig3100 | serine/threonine-protein kinase TOR |
| 253 | Contig3130 | non-specific lipid-transfer protein 1-like |
| 254 | Contig3134 | hypothetical protein CISIN_1g020872mg |
| 255 | Contig3150 | Integrase, catalytic region; Ribonuclease H |
| 256 | Contig3153 | probable aldo-keto reductase 2 |
| 257 | Contig3157 | uncharacterized protein LOC105636756 |
| 258 | Contig3163 | 60S ribosomal protein L3 |
| 259 | Contig3167 | glyceraldehyde-3-phosphate dehydrogenase GAPCP2, chloroplastic |
| 260 | Contig3173 | succinate--CoA ligase [ADP-forming] subunit alpha, mitochondrial |
| 261 | Contig3179 | pyruvate kinase, cytosolic isozyme |
| 262 | Contig3182 | Aspartic peptidase |
| 263 | Contig3188 | JHL23C09.1, partial |
| 264 | Contig3190 | phosphoglycerate kinase, cytosolic |
| 265 | Contig3195 | hypothetical protein VITISV_029446 |
| 266 | Contig3199 | protein disulfide-isomerase-like |
| 267 | Contig3201 | legumin B-like |
| 268 | Contig3206 | aquaporin TIP1-1 |
| 269 | Contig3216 | 40S ribosomal protein S11-like |
| 270 | Contig3218 | fructose-bisphosphate aldolase 1, chloroplastic |
| 271 | Contig3228 | 40S ribosomal protein S19-3 |
| 272 | Contig3230 | 40S ribosomal protein S6 |
| 273 | Contig3247 | 2-alkenal reductase (NADP(+)-dependent) |
| 274 | Contig3248 | orf101d (mitochondrion) |
| 275 | Contig3252 | malate dehydrogenase |
| 276 | Contig3263 | FRIGIDA-like protein 4a |
| 277 | Contig3269 | V-type proton ATPase 16 kDa proteolipid subunit |
| 278 | Contig3279 | proteasome subunit alpha type-7 |
| 279 | Contig3284 | apocytochrome b (mitochondrion) |
| 280 | Contig3289 | thioredoxin f5 |
| 281 | Contig3296 | low-temperature-induced cysteine proteinase-like |
| 282 | Contig3300 | cyclin-B1-2-like |
| 283 | Contig3304 | G-patch domain |
| 284 | Contig3309 | aspartic proteinase-like |
| 285 | Contig3318 | F-box only protein 6 |
| 286 | Contig3320 | classical arabinogalactan protein 4 |
| 287 | Contig3337 | folate synthesis bifunctional protein, mitochondrial-like |
| 288 | Contig3339 | legumin A |
| 289 | Contig3342 | Ureidoglycolate hydrolases |
| 290 | Contig3357 | folate synthesis bifunctional protein, mitochondrial-like |
| 291 | Contig3377 | uncharacterized protein LOC105631058 |
| 292 | Contig3380 | phosphatidylinositol 4-kinase gamma 5-like |
| 293 | Contig3384 | elongation factor 1-alpha |
| 294 | Contig3387 | GTP-binding nuclear protein Ran-3-like |
| 295 | Contig3388 | endoglucanase 11-like |
| 296 | Contig3400 | protein CREG1 |
| 297 | Contig3405 | hypothetical protein JCGZ_14891 |
| 298 | Contig3426 | hypothetical protein JCGZ_02872 |
| 299 | Contig3435 | hypothetical protein JCGZ_21429 |
| 300 | Contig3443 | NADH dehydrogenase [ubiquinone] flavoprotein 2, mitochondrial-like |
| 301 | Contig3446 | 60S ribosomal protein L13-1 |
| 302 | Contig3447 | uncharacterized protein LOC105634316 |
| 303 | Contig3448 | defensin Ec-AMP-D2-like |
| 304 | Contig3481 | probable polygalacturonase |
| 305 | Contig3483 | LRAT domain-containing protein |
| 306 | Contig3494 | peroxidase 42 |
| 307 | Contig3511 | ethylene-responsive transcription factor 2-like |
| 308 | Contig3519 | serine/arginine-rich splicing factor SC35 |
| 309 | Contig3521 | retrotransposon-related protein |
| 310 | Contig3534 | heat shock cognate 70 kDa protein 2 |
| 311 | Contig3539 | phosphoenolpyruvate carboxykinase (ATP) |
| 312 | Contig3556 | reticulon-like protein b5 |
| 313 | Contig3558 | isocitrate lyase |
| 314 | Contig3559 | glutathione S-transferase T1-like |
| 315 | Contig3562 | ATPase subunit 8 (mitochondrion) |
| 316 | Contig3564 | uncharacterized protein At4g13200, chloroplastic |
| 317 | Contig3567 | histone H3.3 |
| 318 | Contig3573 | G-patch domain |
| 319 | Contig3575 | sucrose synthase 2 isoform X1 |
| 320 | Contig3595 | senescence-associated protein |
| 321 | Contig3609 | thioredoxin H-type |
| 322 | Contig3628 | glutathione S-transferase L3-like isoform X1 |
| 323 | Contig3645 | uncharacterized protein LOC105649409 |
| 324 | Contig3657 | heat shock cognate 70 kDa protein 2 |
| 325 | Contig3674 | hypothetical protein JCGZ_09754 |
| 326 | Contig3691 | 60S ribosomal protein L13a-4 |
| 327 | Contig3704 | hypothetical protein JCGZ_14891 |
| 328 | Contig3720 | triosephosphate isomerase, cytosolic |
| 329 | Contig3723 | alpha tubulin 1 |
| 330 | Contig3745 | tubulin alpha-3 chain |
| 331 | Contig3750 | cinnamoyl-CoA reductase 1 |
| 332 | Contig3751 | hypothetical protein JCGZ_14891 |
| 333 | Contig3784 | hypothetical protein JCGZ_07867 |
| 334 | Contig3785 | uncharacterized protein LOC105648192 |
| 335 | Contig3787 | probable polygalacturonase |
| 336 | Contig3789 | hypothetical protein JCGZ_02872 |
| 337 | Contig3795 | alpha tubulin 1 |
| 338 | Contig3799 | tubulin beta-2 chain |
| 339 | Contig3837 | pyrophosphate--fructose 6-phosphate 1-phosphotransferase subunit alpha |
| 340 | Contig3844 | RNA-binding protein 39 |
| 341 | Contig3849 | 5-methyltetrahydropteroyltriglutamate--homocysteine methyltransferase |
| 342 | Contig3850 | probable protein disulfide-isomerase A6 |
| 343 | Contig3878 | hypothetical protein JCGZ_15335 |
| 344 | Contig3880 | ---NA--- |
| 345 | Contig3896 | late embryogenesis abundant protein, group 6 |
| 346 | Contig3908 | hypothetical protein JCGZ_26700 |
| 347 | Contig3922 | hemiasterlin resistant protein 1-like |
| 348 | Contig3949 | cysteine protease RD19A-like |
| 349 | Contig3970 | profilin-1 |
| 350 | Contig3973 | splicing factor 3B subunit 1 |
| 351 | Contig3985 | 2-Cys peroxiredoxin BAS1, chloroplastic |
| 352 | Contig4002 | probable serine/threonine-protein phosphatase 2A regulatory subunit B'' subunit TON2 |
| 353 | Contig4012 | embryonic protein DC-8 |
| 354 | Contig4025 | L-type lectin-domain containing receptor kinase S.6 |
| 355 | Contig4029 | GTP cyclohydrolase 1 |
| 356 | Contig4030 | uncharacterized protein LOC105649868 |
| 357 | Contig4031 | biotin carboxyl carrier protein of acetyl-CoA carboxylase, chloroplastic isoform X2 |
| 358 | Contig4050 | 60S ribosomal protein L11 |
| 359 | Contig4056 | transmembrane protein, putative |
| 360 | Contig4064 | 2S albumin |
| 361 | Contig4072 | protein MOTHER of FT and TFL1-like |
| 362 | Contig4073 | peptide methionine sulfoxide reductase B5-like |
| 363 | Contig4089 | nuclear transport factor 2 |
| 364 | Contig4112 | 60S ribosomal protein L10a |
| 365 | Contig4126 | translationally-controlled tumor protein homolog |
| 366 | Contig4134 | senescence-associated protein, putative |
| 367 | Contig4137 | 40S ribosomal protein S16 |
| 368 | Contig4142 | oleosin 18.2 kDa-like |
| 369 | Contig4154 | vicilin-like seed storage protein At2g28490 |
| 370 | Contig4158 | uncharacterized protein LOC111475501 |
| 371 | Contig4161 | cytochrome P450 like_TBP |
| 372 | Contig4194 | ADP,ATP carrier protein 1, mitochondrial |
| 373 | Contig4211 | 40S ribosomal protein S2-4-like |
| 374 | Contig4218 | plant/MUD21-2 protein |
| 375 | Contig4225 | protein SLE1 |
| 376 | Contig4227 | ran-binding protein 1 homolog b-like |
| 377 | Contig4229 | Em-like protein GEA6 |
| 378 | Contig4238 | Nucleic acid-binding, OB-fold containing protein |
| 379 | Contig4239 | 40S ribosomal protein S10-1-like |
| 380 | Contig4241 | 11S globulin seed storage protein 2-like |
| 381 | Contig4245 | hypothetical protein JCGZ_05719 |
| 382 | Contig4249 | 17.3 kDa class I heat shock protein-like |
| 383 | Contig4254 | 18.1 kDa class I heat shock protein |
| 384 | Contig4266 | elongation factor 1-alpha-like |
| 385 | Contig4267 | ABC transporter F family member 1 |
| 386 | Contig4274 | legumin B-like |
| 387 | Contig4276 | GTP-binding nuclear protein Ran-3 |
| 388 | Contig4277 | oleosin 1-like |
| 389 | Contig4287 | senescence-associated protein |
| 390 | Contig4314 | 60S ribosomal protein L31 |
| 391 | Contig4316 | 40S ribosomal protein S19-3 |
| 392 | Contig4329 | oleosin 18.2 kDa-like |
| 393 | Contig4347 | seed maturation protein |
| 394 | Contig4350 | dehydrin Xero 1 |
| 395 | Contig4355 | ---NA--- |
| 396 | Contig4358 | 17.3 kDa class I heat shock protein |
| 397 | Contig4366 | high mobility group B protein 2-like |
| 398 | Contig4368 | 2S albumin |
| 399 | Contig4393 | 40S ribosomal protein S15-4 |
| 400 | Contig4410 | MATH domain-containing protein At5g43560 isoform X1 |
| 401 | Contig4429 | guanine nucleotide-binding protein-like NSN1 |
| 402 | Contig4444 | glycine-rich RNA-binding protein 2, mitochondrial-like |
| 403 | Contig4454 | oil body-associated protein 1A |
| 404 | Contig4464 | probable aquaporin TIP3-2 |
| 405 | Contig4486 | ---NA--- |
| 406 | Contig4487 | ankyrin repeat domain-containing protein 2A-like |
| 407 | Contig4488 | oil body-associated protein 2A |
| 408 | Contig4512 | Nad2 (mitochondrion) |
| 409 | Contig4525 | ---NA--- |
| 410 | Contig4614 | ubiquitin-activating enzyme E1 1 |
| 411 | Contig4619 | subtilisin-like protease SBT2.5 |
| 412 | Contig4687 | probable histone H2A.1 |
| 413 | Contig4688 | legumin A |
| 414 | Contig4691 | late embryogenesis abundant protein D-7 |
| 415 | Contig4704 | AWPM-19-like membrane family protein |
| 416 | Contig4710 | oleosin 18.2 kDa-like |
| 417 | Contig4727 | hypothetical protein MTR_0055s0030 |
| 418 | Contig4745 | glutathione S-transferase F9-like |
| 419 | Contig4753 | pyruvate kinase isozyme A, chloroplastic |
| 420 | Contig4757 | 60S acidic ribosomal protein P2B-like |
| 421 | Contig4758 | 17.3 kDa class I heat shock protein |
| 422 | Contig4772 | nascent polypeptide-associated complex subunit alpha-like protein 2 |
| 423 | Contig4784 | Serine/arginine repetitive matrix protein 2, putative isoform 2 |
| 424 | Contig4802 | elongation factor 1-beta 2 |
| 425 | Contig4808 | 60S ribosomal protein L17-2 |
| 426 | Contig4823 | uncharacterized oxidoreductase At4g09670-like |
| 427 | Contig4829 | elongation factor 1-alpha |
| 428 | Contig4830 | uncharacterized transmembrane protein DDB_G0289901 |
| 429 | Contig4831 | hypothetical protein JCGZ_25663 |
| 430 | Contig4836 | casein kinase 1-like protein 6 isoform X1 |
| 431 | Contig4846 | protein CURVATURE THYLAKOID 1C, chloroplastic |
| 432 | Contig4847 | ---NA--- |
| 433 | Contig4850 | major allergen Pru ar 1-like |
| 434 | Contig4853 | heparan-alpha-glucosaminide N-acetyltransferase |
| 435 | Contig4867 | ribulose bisphosphate carboxylase small chain, chloroplastic |
| 436 | Contig4871 | 14 kDa proline-rich protein DC2.15-like |
| 437 | Contig4878 | ---NA--- |
| 438 | Contig4884 | glycine-rich cell wall structural protein 2 |
| 439 | Contig4888 | late embryogenesis abundant protein Lea5-D |
| 440 | Contig4894 | chlorophyll a-b binding protein 151, chloroplastic |
| 441 | Contig4899 | photosystem II 10 kDa polypeptide, chloroplastic |
| 442 | Contig4906 | putative lipid-transfer protein DIR1 |
| 443 | Contig4909 | chlorophyll a-b binding protein of LHCII type 1 |
| 444 | Contig4919 | VAMP-like protein YKT61 |
| 445 | Contig4925 | 14 kDa proline-rich protein DC2.15-like |
| 446 | Contig4927 | putative lipid-transfer protein DIR1 |
| 447 | Contig4935 | metallothionein-like protein type 2 |
| 448 | Contig4936 | metallothionein-like protein type 2 |
| 449 | Contig4937 | metallothionein-like protein type 2 |
| 450 | Contig4938 | hydrophobic protein RCI2B |
| 451 | Contig4941 | polyubiquitin-like |
| 452 | Contig4943 | 60S ribosomal protein L23 |
| 453 | Contig4944 | 40S ribosomal protein S14-3 |
| 454 | Contig4955 | uncharacterized transmembrane protein DDB_G0289901 |
| 455 | Contig4961 | ubiquitin-conjugating enzyme E2-17 kDa |
| 456 | Contig4987 | ---NA--- |
| 457 | Contig5005 | ---NA--- |
| 458 | Contig5011 | choline/ethanolaminephosphotransferase 1 |
| 459 | Contig5012 | 40S ribosomal protein S28-2 |
| 460 | Contig5020 | ferredoxin-like |
| 461 | Contig5029 | histone H4 |
| 462 | Contig5057 | protein BPS1, chloroplastic |

***Euphorbia lagascae***

| **Sl. No.** | **ID** | **Function** |
| --- | --- | --- |
| 1 | Contig9 | agmatine deiminase |
| 2 | Contig13 | agmatine deiminase |

***Manihot esculenta***

| **Sl. No.** | **ID** | **Function** |
| --- | --- | --- |
| 1 | Contig12 | 40S ribosomal protein S7-like |
| 2 | Contig13 | proactivator polypeptide-like 1 |
| 3 | Contig19 | zinc finger CCCH domain-containing protein 29-like |
| 4 | Contig21 | glycine-rich RNA-binding protein 1-like |
| 5 | Contig22 | 60S ribosomal protein L27a-3-like |
| 6 | Contig23 | glycine-rich domain-containing protein 1-like |
| 7 | Contig24 | ---NA--- |
| 8 | Contig29 | 60S ribosomal protein L10 |
| 9 | Contig30 | lysine-specific histone demethylase 1 homolog 3 |
| 10 | Contig31 | hypothetical protein GLYMA_13G021700, partial |
| 11 | Contig32 | probable fructokinase-4 |
| 12 | Contig35 | E3 ubiquitin-protein ligase BOI isoform X2 |
| 13 | Contig36 | probable glucuronoxylan glucuronosyltransferase F8H isoform X2 |
| 14 | Contig38 | 60S ribosomal protein L18a |
| 15 | Contig39 | succinate dehydrogenase assembly factor 2, mitochondrial |
| 16 | Contig43 | hypothetical protein KK1_020634 |
| 17 | Contig45 | dual specificity phosphatase Cdc25 |
| 18 | Contig46 | hypothetical protein KK1_020634 |
| 19 | Contig48 | translocase of chloroplast 120, chloroplastic-like |
| 20 | Contig49 | uncharacterized protein LOC110606542 |
| 21 | Contig50 | cytochrome c oxidase subunit 5c |
| 22 | Contig51 | Calcium and calcium/calmodulin-dependent serine/threonine-protein kinase isoform 1 |
| 23 | Contig52 | sucrose synthase |
| 24 | Contig53 | Wound-induced protein 1 |
| 25 | Contig57 | cell division cycle 5-like protein |
| 26 | Contig58 | Copper centre Cu(A) |
| 27 | Contig64 | Sodium/hydrogen exchanger 7 |
| 28 | Contig67 | signal peptide peptidase-like 2 |
| 29 | Contig71 | hypothetical protein MANES_15G002800 |
| 30 | Contig73 | secretory carrier-associated membrane protein 3-like |
| 31 | Contig76 | protein-L-isoaspartate O-methyltransferase 1 isoform X1 |
| 32 | Contig77 | sucrose synthase |
| 33 | Contig84 | guanine nucleotide-binding protein subunit beta-like protein |
| 34 | Contig85 | RING-H2 finger protein ATL56-like |
| 35 | Contig88 | cysteine-rich PDZ-binding protein |
| 36 | Contig108 | eukaryotic translation initiation factor 4B3 |
| 37 | Contig112 | ATP-dependent zinc metalloprotease FTSH 4, mitochondrial |
| 38 | Contig123 | hypothetical protein MANES_12G025700 |
| 39 | Contig126 | premnaspirodiene oxygenase-like |
| 40 | Contig128 | ---NA--- |
| 41 | Contig143 | elongation factor 1-alpha |
| 42 | Contig158 | cysteine protease RD19A |
| 43 | Contig167 | 60S ribosomal protein L15-1 |
| 44 | Contig195 | ribulose-1,5-bisphosphate carboxylase/oxygenase small subunit (chloroplast) |
| 45 | Contig215 | photosystem I reaction center subunit V, chloroplastic |
| 46 | Contig218 | KH domain-containing protein At1g09660/At1g09670-like isoform X1 |
| 47 | Contig221 | dnaJ protein homolog |
| 48 | Contig236 | kinesin-like protein KIN-7E |
| 49 | Contig245 | hypothetical protein MANES_06G003500 |
| 50 | Contig246 | Na+/H+ antiporter family protein 2 |
| 51 | Contig247 | 60S ribosomal protein L12 |
| 52 | Contig259 | casein kinase 1-like protein 1 |
| 53 | Contig267 | photosystem II 22 kDa protein, chloroplastic-like |
| 54 | Contig268 | RNA/RNP complex-1-interacting phosphatase, putative |
| 55 | Contig275 | UV-B-induced protein At3g17800, chloroplastic-like |
| 56 | Contig278 | vacuolar cation/proton exchanger 3 |
| 57 | Contig281 | calcium homeostasis endoplasmic reticulum protein |
| 58 | Contig292 | peptidyl-prolyl cis-trans isomerase FKBP13, chloroplastic |
| 59 | Contig293 | E3 ubiquitin protein ligase RIE1-like |
| 60 | Contig304 | 40S ribosomal protein S21-2 |
| 61 | Contig305 | probable galactinol--sucrose galactosyltransferase 2 |
| 62 | Contig309 | aspartate aminotransferase, chloroplastic |
| 63 | Contig312 | zinc finger A20 and AN1 domain-containing stress-associated protein 5-like |
| 64 | Contig324 | probable E3 ubiquitin-protein ligase RHC2A |
| 65 | Contig341 | hypothetical protein MANES_05G160400 |
| 66 | Contig346 | 5-methyltetrahydropteroyltriglutamate--homocysteine methyltransferase |
| 67 | Contig350 | cytochrome c oxidase subunit 6a, mitochondrial-like |
| 68 | Contig351 | hypothetical protein MANES_06G041600, partial |
| 69 | Contig360 | enhancer of mRNA-decapping protein 4-like |
| 70 | Contig364 | translation initiation factor IF3-1, mitochondrial |
| 71 | Contig371 | EIN3-binding F-box protein 1-like |
| 72 | Contig376 | inner membrane protein PPF-1, chloroplastic |
| 73 | Contig379 | Ribosomal protein L36, putative |
| 74 | Contig400 | uncharacterized protein LOC110614483 |
| 75 | Contig407 | 60S ribosomal protein L19-3 |
| 76 | Contig414 | CASP-like protein 2B1 |
| 77 | Contig415 | universal stress protein A-like protein |
| 78 | Contig417 | 50S ribosomal protein L24, chloroplastic |
| 79 | Contig432 | chaperonin 60 subunit beta 2, chloroplastic |
| 80 | Contig443 | elongation factor 1-delta 1 |
| 81 | Contig445 | polyol transporter 5 |
| 82 | Contig447 | uncharacterized protein LOC110612173 |
| 83 | Contig455 | Rubredoxin, putative |
| 84 | Contig461 | stem-specific protein TSJT1-like |
| 85 | Contig475 | THO complex subunit 4D isoform X2 |
| 86 | Contig485 | protein Dr1 homolog isoform X1 |
| 87 | Contig488 | protein CURVATURE THYLAKOID 1A, chloroplastic |
| 88 | Contig502 | zinc finger A20 and AN1 domain-containing stress-associated protein 4-like |
| 89 | Contig511 | glutathione peroxidase |
| 90 | Contig518 | protein SRC2 homolog |
| 91 | Contig528 | geranylgeranyl diphosphate reductase, chloroplastic |
| 92 | Contig541 | ABSCISIC ACID-INSENSITIVE 5-like protein 7 |
| 93 | Contig544 | hypothetical protein MANES_07G005200 |
| 94 | Contig554 | cell division control protein 2 homolog A |
| 95 | Contig555 | 29 kDa ribonucleoprotein A, chloroplastic-like |
| 96 | Contig559 | aldehyde dehydrogenase family 3 member H1 |
| 97 | Contig562 | eukaryotic translation initiation factor 3 subunit F-like |
| 98 | Contig577 | luminal binding protein (BiP) |
| 99 | Contig584 | Cytochrome-c oxidases,electron carriers |
| 100 | Contig609 | ADP-ribosylation factor 1-like |
| 101 | Contig611 | cytochrome b5 |
| 102 | Contig613 | ferric reduction oxidase 6-like |
| 103 | Contig618 | late embryogenesis abundant protein Lea5-D |
| 104 | Contig619 | thioredoxin-like protein YLS8 |
| 105 | Contig622 | hypothetical protein MANES_14G128000 |
| 106 | Contig632 | ---NA--- |
| 107 | Contig637 | OTU domain-containing protein At3g57810-like |
| 108 | Contig651 | EID1-like F-box protein 2 |
| 109 | Contig657 | receptor-like protein kinase FERONIA |
| 110 | Contig661 | 4-hydroxy-3-methylbut-2-enyl diphosphate reductase |
| 111 | Contig669 | vacuolar protein sorting-associated protein 32 homolog 2 |
| 112 | Contig677 | Ribosomal protein S21 family protein isoform 1 |
| 113 | Contig684 | 60S ribosomal protein L38 |
| 114 | Contig687 | thiol protease aleurain-like |
| 115 | Contig690 | phosphatidylcholine transfer protein, putative |
| 116 | Contig693 | ubiquitin-like protein 5 |
| 117 | Contig696 | protein Iojap, chloroplastic |
| 118 | Contig697 | eukaryotic translation initiation factor 3 subunit C-like |
| 119 | Contig707 | histone H2B |
| 120 | Contig713 | pentatricopeptide repeat-containing protein At5g46580, chloroplastic |
| 121 | Contig715 | protein PELOTA 1 |
| 122 | Contig717 | transcription factor MYB44 |
| 123 | Contig724 | chaperone protein ClpC, chloroplastic |
| 124 | Contig772 | 60S ribosomal protein L26-1 |
| 125 | Contig773 | ferredoxin-like |
| 126 | Contig775 | phosphoserine aminotransferase 2, chloroplastic-like |
| 127 | Contig781 | ---NA--- |
| 128 | Contig812 | NADH-ubiquinone reductase complex 1 MLRQ subunit |
| 129 | Contig816 | ribonuclease TUDOR 1-like |
| 130 | Contig822 | vacuolar-processing enzyme |
| 131 | Contig827 | uncharacterized protein LOC110615711 |
| 132 | Contig828 | late embryogenesis abundant protein At5g17165-like |
| 133 | Contig829 | endoplasmin homolog |
| 134 | Contig830 | 5'-adenylylsulfate reductase-like 5 |
| 135 | Contig846 | annexin D2-like |
| 136 | Contig851 | ras-interacting protein RIP3 |
| 137 | Contig855 | elongation factor 2 |
| 138 | Contig858 | asparagine--tRNA ligase, cytoplasmic 1-like |
| 139 | Contig859 | 10 kDa chaperonin 1, chloroplastic-like |
| 140 | Contig873 | non-specific lipid-transfer protein 8 |
| 141 | Contig876 | probable E3 ubiquitin-protein ligase RHC2A |
| 142 | Contig879 | glyceraldehyde-3-phosphate dehydrogenase A, chloroplastic isoform X2 |
| 143 | Contig890 | triosephosphate isomerase, cytosolic |
| 144 | Contig891 | rhodanese-like domain-containing protein 14, chloroplastic |
| 145 | Contig893 | methionine gamma-lyase |
| 146 | Contig917 | putative RING-type E3 ubiquitin transferase C3H69 isoform X1 |
| 147 | Contig934 | cytochrome b-c1 complex subunit 9 |
| 148 | Contig955 | chlorophyll a-b binding protein, chloroplastic |
| 149 | Contig958 | protein CURVATURE THYLAKOID 1C, chloroplastic |
| 150 | Contig983 | heavy metal-associated isoprenylated plant protein 39-like |
| 151 | Contig985 | chaperone protein dnaJ 11, chloroplastic-like |
| 152 | Contig996 | dnaJ protein homolog |
| 153 | Contig1004 | long chain base biosynthesis protein 2a-like |
| 154 | Contig1008 | transcription elongation factor 1 homolog |
| 155 | Contig1009 | protein CURVATURE THYLAKOID 1A, chloroplastic-like |
| 156 | Contig1026 | peptidyl-prolyl cis-trans isomerase |
| 157 | Contig1029 | BEL1-like homeodomain protein 1 |
| 158 | Contig1037 | auxin response factor 2A-like |
| 159 | Contig1038 | lisH domain-containing protein C1711.05 |
| 160 | Contig1040 | vacuolar-sorting receptor 3 |
| 161 | Contig1052 | homeobox-leucine zipper protein ATHB-12-like |
| 162 | Contig1054 | photosystem I reaction center subunit psaK, chloroplastic |
| 163 | Contig1070 | ubiquitin-conjugating enzyme E2 10 |
| 164 | Contig1071 | XIAP-associated factor 1-like |
| 165 | Contig1089 | acid phosphatase 1 |
| 166 | Contig1094 | elongation factor 1-alpha |
| 167 | Contig1099 | probable ribosome-binding factor A, chloroplastic |
| 168 | Contig1114 | protein TIC 20-I, chloroplastic-like |
| 169 | Contig1116 | histone H2B-like |
| 170 | Contig1121 | translationally-controlled tumor protein homolog |
| 171 | Contig1122 | transcription elongation factor 1 homolog |
| 172 | Contig1123 | non-functional NADPH-dependent codeinone reductase 2-like |
| 173 | Contig1125 | stress enhanced protein 1, chloroplastic |
| 174 | Contig1130 | tubby-like F-box protein 5 |
| 175 | Contig1142 | nudix hydrolase 17, mitochondrial-like |
| 176 | Contig1157 | putative LOV domain-containing protein |
| 177 | Contig1185 | transcription factor bHLH48-like |
| 178 | Contig1197 | metalloendoproteinase 1-like |
| 179 | Contig1202 | NAD(P)-binding domain containing protein |
| 180 | Contig1207 | non-specific lipid-transfer protein-like protein At5g64080 |
| 181 | Contig1221 | alpha/beta-gliadin A-III |
| 182 | Contig1223 | 60S ribosomal protein L14-1 |
| 183 | Contig1224 | nascent polypeptide-associated complex subunit alpha-like protein 1 |
| 184 | Contig1234 | mediator of RNA polymerase II transcription subunit 15a-like |
| 185 | Contig1244 | caffeoyl-CoA O-methyltransferase |
| 186 | Contig1252 | ribulose bisphosphate carboxylase small chain, chloroplastic-like |
| 187 | Contig1254 | prostamide/prostaglandin F synthase |
| 188 | Contig1255 | uncharacterized protein LOC110620109 isoform X1 |
| 189 | Contig1257 | nucleotide binding protein, putative |
| 190 | Contig1269 | protein kinase APK1A, chloroplastic-like isoform X1 |
| 191 | Contig1273 | annexin D2-like |
| 192 | Contig1279 | triose phosphate/phosphate translocator, non-green plastid, chloroplastic-like |
| 193 | Contig1282 | malate dehydrogenase |
| 194 | Contig1288 | 40S ribosomal protein S20-2 |
| 195 | Contig1362 | eukaryotic translation initiation factor NCBP |
| 196 | Contig1364 | TBC1 domain family member 15 |
| 197 | Contig1367 | peptide methionine sulfoxide reductase A1-like |
| 198 | Contig1377 | abscisic acid receptor PYR1 |
| 199 | Contig1417 | protein Asterix |
| 200 | Contig1418 | 3-ketoacyl-CoA thiolase 2, peroxisomal |
| 201 | Contig1439 | ferredoxin-like |
| 202 | Contig1440 | nascent polypeptide-associated complex subunit beta-like |
| 203 | Contig1441 | dr1-associated corepressor homolog |
| 204 | Contig1455 | transmembrane protein 258-like |
| 205 | Contig1456 | coatomer subunit zeta-1-like |
| 206 | Contig1458 | uncharacterized protein LOC110610326 isoform X1 |
| 207 | Contig1460 | SEC12-like protein 2 |
| 208 | Contig1475 | probable calcium-binding protein CML13 |
| 209 | Contig1484 | auxin-repressed 12.5 kDa protein-like |
| 210 | Contig1487 | mitochondrial import receptor subunit TOM7-1-like |
| 211 | Contig1492 | translation machinery-associated protein 7-like |
| 212 | Contig1493 | Collagen alpha-4(VI) chain |
| 213 | Contig1504 | carboxymethylenebutenolidase homolog |
| 214 | Contig1506 | cationic amino acid transporter 2, vacuolar-like |
| 215 | Contig1507 | prohibitin-1, mitochondrial |
| 216 | Contig1517 | 40S ribosomal protein S14-3 |
| 217 | Contig1545 | heat shock factor protein HSF24 |
| 218 | Contig1547 | ---NA--- |
| 219 | Contig1549 | eukaryotic translation initiation factor 2D |
| 220 | Contig1553 | auxin-responsive protein IAA9-like |
| 221 | Contig1556 | R3H domain-containing protein 2 |
| 222 | Contig1560 | 26S proteasome non-ATPase regulatory subunit 2 homolog A |
| 223 | Contig1574 | 7-deoxyloganetin glucosyltransferase-like |
| 224 | Contig1586 | glutamate decarboxylase 1 |
| 225 | Contig1589 | chloroplast stem-loop binding protein of 41 kDa b, chloroplastic |
| 226 | Contig1596 | dynein light chain 2, cytoplasmic |
| 227 | Contig1600 | 40S ribosomal protein S14-3 |
| 228 | Contig1601 | lysophospholipid acyltransferase LPEAT1 isoform X1 |
| 229 | Contig1608 | shaggy-related protein kinase alpha |
| 230 | Contig1610 | monothiol glutaredoxin-S7, chloroplastic |
| 231 | Contig1614 | oligouridylate-binding protein 1-like |
| 232 | Contig1619 | 50S ribosomal protein L34, chloroplastic-like |
| 233 | Contig1632 | ATP phosphoribosyltransferase 2, chloroplastic-like |
| 234 | Contig1651 | probable protein phosphatase 2C 25 |
| 235 | Contig1677 | probable serine incorporator |
| 236 | Contig1686 | probable 26S proteasome non-ATPase regulatory subunit 3 |
| 237 | Contig1723 | ubiquitin-conjugating enzyme E2 34-like |
| 238 | Contig1743 | small nuclear ribonucleoprotein SmD3b-like |
| 239 | Contig1756 | histone H2A |
| 240 | Contig1761 | ankyrin repeat domain-containing protein 2-like |
| 241 | Contig1765 | la-related protein 6B |
| 242 | Contig1766 | protein BOLA4, chloroplastic/mitochondrial |
| 243 | Contig1776 | protein PEROXIN-4 |
| 244 | Contig1789 | cytochrome P450 72A15-like |
| 245 | Contig1792 | tetraspanin-3-like |
| 246 | Contig1799 | SKP1-like protein 1B |
| 247 | Contig1800 | hypothetical protein POPTR_005G107600v3 |
| 248 | Contig1804 | probable CCR4-associated factor 1 homolog 9 |
| 249 | Contig1811 | homeobox-leucine zipper protein ATHB-6 |
| 250 | Contig1833 | MLP-like protein 423 |
| 251 | Contig1844 | glucose-6-phosphate isomerase 1, chloroplastic |
| 252 | Contig1853 | endoribonuclease Dicer homolog 2-like isoform X1 |
| 253 | Contig1856 | T-complex protein 1 subunit alpha |
| 254 | Contig1876 | uncharacterized protein LOC110599682 |
| 255 | Contig1884 | NADPH--cytochrome P450 reductase 2 |
| 256 | Contig1889 | cyclin-B1-2-like |
| 257 | Contig1894 | sucrose transport protein SUC4-like |
| 258 | Contig1903 | catalase isozyme 2 |
| 259 | Contig1907 | aconitate hydratase, cytoplasmic |
| 260 | Contig1921 | formate dehydrogenase, mitochondrial |
| 261 | Contig1924 | hypothetical protein MANES_06G031800 |
| 262 | Contig1929 | CAX-interacting protein 4 |
| 263 | Contig1941 | cellulose synthase-like protein E1 |
| 264 | Contig1947 | DCC family protein At1g52590, chloroplastic |
| 265 | Contig1957 | DUF3067 domain-containing protein |
| 266 | Contig1970 | NADH dehydrogenase [ubiquinone] 1 beta subcomplex subunit 3-B-like |
| 267 | Contig1983 | photosystem II core complex proteins psbY, chloroplastic-like |
| 268 | Contig1988 | protein ETHYLENE INSENSITIVE 3-like |
| 269 | Contig1999 | transformation/transcription domain-associated protein-like |
| 270 | Contig2005 | uncharacterized protein LOC110627706 |
| 271 | Contig2013 | membrane-associated 30 kDa protein, chloroplastic-like |
| 272 | Contig2017 | protein-lysine N-methyltransferase Mettl10 |
| 273 | Contig2018 | 2-methylbutanal oxime monooxygenase |
| 274 | Contig2026 | ribulose bisphosphate carboxylase small chain clone 512-like |
| 275 | Contig2031 | ATP-dependent Clp protease proteolytic subunit-related protein 1, chloroplastic |
| 276 | Contig2034 | protein LNK1 isoform X2 |
| 277 | Contig2040 | dnaJ protein homolog |
| 278 | Contig2043 | protein BOLA2 |
| 279 | Contig2049 | hypothetical protein MANES_18G027500 |
| 280 | Contig2053 | RING finger protein 10 |
| 281 | Contig2073 | ubiquitin-protein ligase, putative |
| 282 | Contig2088 | 17.9 kDa class II heat shock protein-like |
| 283 | Contig2091 | Echinoderm microtubule-associated protein-like 6 |
| 284 | Contig2092 | long chain acyl-CoA synthetase 6, peroxisomal |
| 285 | Contig2093 | signal recognition particle receptor subunit alpha-like |
| 286 | Contig2135 | H/ACA ribonucleoprotein complex subunit 3-like protein |
| 287 | Contig2141 | cytochrome b5-like |
| 288 | Contig2145 | peptide methionine sulfoxide reductase B5-like |
| 289 | Contig2168 | aspartic proteinase PCS1 |
| 290 | Contig2176 | uncharacterized protein LOC110618729 |
| 291 | Contig2180 | 40S ribosomal protein S27-2 |
| 292 | Contig2181 | 18.1 kDa class I heat shock protein-like |
| 293 | Contig2183 | 40S ribosomal protein S15-4 |
| 294 | Contig2184 | pre-mRNA splicing factor SR-like 1 isoform X1 |
| 295 | Contig2185 | protein transport protein Sec61 subunit gamma-like |
| 296 | Contig2189 | ubiquitin-40S ribosomal protein S27a |
| 297 | Contig2194 | allene oxide cyclase, chloroplastic-like |
| 298 | Contig2200 | putative lipid-transfer protein DIR1 |
| 299 | Contig2201 | ras-related protein RABF1 |
| 300 | Contig2203 | Protein LRP16, putative |
| 301 | Contig2217 | 40S ribosomal protein S26-1-like |
| 302 | Contig2219 | putative lactoylglutathione lyase |
| 303 | Contig2220 | 60S ribosomal protein L24 |
| 304 | Contig2223 | calmodulin-like protein 1 |
| 305 | Contig2225 | 1-aminocyclopropane-1-carboxylate oxidase homolog 4-like |
| 306 | Contig2226 | aspartic proteinase A1-like |
| 307 | Contig2229 | 40S ribosomal protein S21-2 |
| 308 | Contig2239 | transmembrane emp24 domain-containing protein p24beta3 |
| 309 | Contig2240 | acetate/butyrate--CoA ligase AAE7, peroxisomal |
| 310 | Contig2241 | ATP sulfurylase 1, chloroplastic-like |
| 311 | Contig2242 | 60S ribosomal protein L13-1 |
| 312 | Contig2244 | alpha-soluble NSF attachment protein 2-like |
| 313 | Contig2246 | dolichyl-phosphate beta-glucosyltransferase |
| 314 | Contig2247 | mitochondrial import receptor subunit TOM6 homolog |
| 315 | Contig2252 | S-adenosylmethionine synthase 1 |
| 316 | Contig2260 | AP-1 complex subunit mu-2 |
| 317 | Contig2265 | superoxide dismutase [Cu-Zn] |
| 318 | Contig2272 | protein LIGHT-DEPENDENT SHORT HYPOCOTYLS 10-like |
| 319 | Contig2274 | programmed cell death protein 4 |
| 320 | Contig2275 | hypoia-responsive family protein 2 |
| 321 | Contig2278 | histone H4 |
| 322 | Contig2282 | 7-deoxyloganetin glucosyltransferase-like |
| 323 | Contig2285 | superoxide dismutase [Cu-Zn], chloroplastic |
| 324 | Contig2289 | accelerated cell death 11 |
| 325 | Contig2301 | glutathione S-transferase-like |
| 326 | Contig2308 | endochitinase-like |
| 327 | Contig2309 | 60S ribosomal protein L37a |
| 328 | Contig2310 | TMV resistance protein N-like |
| 329 | Contig2316 | lachrymatory-factor synthase-like |
| 330 | Contig2317 | elongation factor 1-alpha |
| 331 | Contig2318 | 40S ribosomal protein S27-2 |
| 332 | Contig2323 | 40S ribosomal protein S15a-1 |
| 333 | Contig2324 | ribosome maturation protein SBDS |
| 334 | Contig2328 | eukaryotic translation initiation factor 1A-like |
| 335 | Contig2336 | 40S ribosomal protein S11 |
| 336 | Contig2338 | ---NA--- |
| 337 | Contig2339 | cytochrome b5 |
| 338 | Contig2341 | E3 ubiquitin-protein ligase RDUF1-like |
| 339 | Contig2344 | stress-response A/B barrel domain-containing protein At5g22580-like |
| 340 | Contig2350 | ATP synthase subunit d, mitochondrial |
| 341 | Contig2354 | S-norcoclaurine synthase 2-like |
| 342 | Contig2360 | mitochondrial import receptor subunit TOM6 homolog |
| 343 | Contig2369 | stem-specific protein TSJT1-like |
| 344 | Contig2377 | ethylene-responsive transcription factor 4 |
| 345 | Contig2382 | signal peptidase complex catalytic subunit SEC11A-like |
| 346 | Contig2383 | nascent polypeptide-associated complex subunit alpha-like protein 2 |
| 347 | Contig2384 | RAB6-interacting golgin |
| 348 | Contig2385 | 60S ribosomal protein L35 |
| 349 | Contig2386 | cytochrome P450 81D11-like |
| 350 | Contig2388 | isopentenyl-diphosphate Delta-isomerase I |
| 351 | Contig2391 | B12D protein |
| 352 | Contig2392 | universal stress protein PHOS32-like |
| 353 | Contig2396 | mitochondrial import receptor subunit TOM20-like |
| 354 | Contig2401 | 40S ribosomal protein S9-2-like |
| 355 | Contig2406 | patellin-3 isoform X1 |
| 356 | Contig2409 | cytochrome b561 and DOMON domain-containing protein At5g35735-like |
| 357 | Contig2416 | cysteine proteinase inhibitor 5-like |
| 358 | Contig2425 | B-cell receptor-associated protein |
| 359 | Contig2426 | uncharacterized protein LOC110611937 |
| 360 | Contig2427 | peptide methionine sulfoxide reductase B5-like |
| 361 | Contig2431 | calmodulin-7-like isoform X2 |
| 362 | Contig2432 | Actin cross-linking protein, putative |
| 363 | Contig2439 | major allergen Pru ar 1 |
| 364 | Contig2447 | tobamovirus multiplication protein 2B |
| 365 | Contig2457 | polyubiquitin 9 |
| 366 | Contig2461 | blue copper protein-like |
| 367 | Contig2469 | ---NA--- |
| 368 | Contig2474 | calcium-binding protein PBP1-like |
| 369 | Contig2480 | 60S ribosomal protein L18a |
| 370 | Contig2484 | NADH dehydrogenase [ubiquinone] 1 beta subcomplex subunit 7-like |
| 371 | Contig2490 | tetratricopeptide repeat protein 1-like |
| 372 | Contig2492 | sister chromatid cohesion 1 protein 4-like isoform X1 |
| 373 | Contig2498 | 14 kDa zinc-binding protein |
| 374 | Contig2501 | actin T1-like protein |
| 375 | Contig2502 | eukaryotic translation initiation factor 4e |
| 376 | Contig2506 | aconitate hydratase 1 |
| 377 | Contig2508 | DUF538 domain-containing protein |
| 378 | Contig2517 | calmodulin-like protein 5 |
| 379 | Contig2519 | autophagy-related protein 8C-like isoform X1 |
| 380 | Contig2521 | uncharacterized protein At4g22758-like |
| 381 | Contig2525 | protein DEHYDRATION-INDUCED 19 homolog 4-like |
| 382 | Contig2532 | SEC14 cytosolic factor |
| 383 | Contig2534 | 40S ribosomal protein S23 |
| 384 | Contig2539 | protein mago nashi homolog |
| 385 | Contig2540 | ADP-ribosylation factor 2 |
| 386 | Contig2549 | uncharacterized protein LOC110638161 |
| 387 | Contig2553 | 40S ribosomal protein S3-3-like |
| 388 | Contig2558 | ras-related protein RABF2b |
| 389 | Contig2561 | probable metal-nicotianamine transporter YSL5 |
| 390 | Contig2565 | non-specific lipid-transfer protein 2-like |
| 391 | Contig2566 | 60S ribosomal protein L32-1 |
| 392 | Contig2569 | salicylic acid-binding protein 2-like |
| 393 | Contig2580 | cold-regulated 413 plasma membrane protein 1 |
| 394 | Contig2581 | basic blue protein-like |
| 395 | Contig2584 | dnaJ homolog subfamily B member 6 |
| 396 | Contig2591 | protein NUCLEAR FUSION DEFECTIVE 6, chloroplastic/mitochondrial-like |
| 397 | Contig2602 | eukaryotic translation initiation factor 5A-2 |
| 398 | Contig2607 | eukaryotic translation initiation factor 5A-like |
| 399 | Contig2611 | BAG family molecular chaperone regulator 3-like |
| 400 | Contig2613 | 60S ribosomal protein L37-3 |
| 401 | Contig2614 | metallothionein-like protein type 3 |
| 402 | Contig2620 | elongation factor 1-delta-like |
| 403 | Contig2621 | protein ripply1 |
| 404 | Contig2626 | GATA transcription factor 5-like |
| 405 | Contig2630 | thioredoxin Y2, chloroplastic-like |
| 406 | Contig2632 | cytochrome c |
| 407 | Contig2637 | ---NA--- |
| 408 | Contig2638 | Small nuclear ribonucleoprotein Sm D1 |
| 409 | Contig2639 | indole-3-acetic acid-induced protein ARG2 |
| 410 | Contig2643 | hypothetical protein MANES_15G133700 |
| 411 | Contig2644 | inosine triphosphate pyrophosphatase |
| 412 | Contig2649 | thioredoxin H-type-like |
| 413 | Contig2661 | 40S ribosomal protein S4-1-like |
| 414 | Contig2662 | 30S ribosomal protein S21, chloroplastic-like |
| 415 | Contig2664 | ubiquitin-conjugating enzyme E2 20 |
| 416 | Contig2665 | 40S ribosomal protein S5 |
| 417 | Contig2666 | phosphoinositide phospholipase C 2-like |
| 418 | Contig2673 | pollen-specific protein C13-like |
| 419 | Contig2679 | probable calcium-binding protein CML13 |
| 420 | Contig2683 | light-regulated protein |
| 421 | Contig2686 | B-box zinc finger protein 24-like |
| 422 | Contig2690 | ADP-ribosylation factor-like protein 5 |
| 423 | Contig2693 | v-type proton atpase subunit e1 |
| 424 | Contig2697 | pectin acetylesterase 12 |
| 425 | Contig2703 | 40S ribosomal protein S25-2 |
| 426 | Contig2705 | beta-glucosidase BoGH3B |
| 427 | Contig2712 | 50S ribosomal protein 5 alpha, chloroplastic-like |
| 428 | Contig2714 | zinc finger A20 and AN1 domain-containing stress-associated protein 1 |
| 429 | Contig2716 | CBS domain-containing protein CBSX5-like |
| 430 | Contig2720 | 14-3-3-like protein |
| 431 | Contig2723 | metallothiol transferase FosB-like |
| 432 | Contig2727 | protein DETOXIFICATION 27-like |
| 433 | Contig2739 | thioredoxin H2 |
| 434 | Contig2740 | DNA-binding protein DDB_G0278111 |
| 435 | Contig2743 | mitochondrial outer membrane protein porin of 34 kDa-like |
| 436 | Contig2744 | protein BUD31 homolog 2 |
| 437 | Contig2749 | cucumber peeling cupredoxin-like |
| 438 | Contig2760 | HVA22-like protein k |
| 439 | Contig2764 | CASP-like protein 2C1 |
| 440 | Contig2766 | V-type proton ATPase subunit G 1-like |
| 441 | Contig2768 | protein SDE2 homolog |
| 442 | Contig2784 | thioredoxin H7-like |
| 443 | Contig2785 | 50S ribosomal protein L21, chloroplastic |
| 444 | Contig2810 | 21 kDa protein-like |
| 445 | Contig2811 | probable mannitol dehydrogenase |
| 446 | Contig2816 | glycine cleavage system H protein, mitochondrial |
| 447 | Contig2818 | MADS-box protein SVP isoform X1 |
| 448 | Contig2821 | V-type proton ATPase subunit B 2 |
| 449 | Contig2826 | magnesium-chelatase subunit ChlI, chloroplastic |
| 450 | Contig2827 | DnaJ/Hsp40 cysteine-rich domain superfamily protein isoform 1 |
| 451 | Contig2829 | proteasome subunit beta type-3-A |
| 452 | Contig2831 | protein HGV2 |
| 453 | Contig2833 | uncharacterized protein LOC110623063 |
| 454 | Contig2836 | probable steroid-binding protein 3 |
| 455 | Contig2844 | uncharacterized protein LOC110606755 |
| 456 | Contig2847 | annexin D1 |
| 457 | Contig2849 | probable ribose-5-phosphate isomerase 2 |
| 458 | Contig2853 | copper transporter 5.1 |
| 459 | Contig2857 | nicotinamidase 1-like |
| 460 | Contig2859 | 40S ribosomal protein S13 |
| 461 | Contig2861 | 60S ribosomal protein L13a-4 |
| 462 | Contig2866 | 60S ribosomal protein L36-2-like |
| 463 | Contig2871 | 17.3 kDa class I heat shock protein-like |
| 464 | Contig2874 | repetitive proline-rich cell wall protein 1-like |
| 465 | Contig2877 | phosphoglycerate kinase, chloroplastic |
| 466 | Contig2879 | glucose-6-phosphate/phosphate translocator 2, chloroplastic |
| 467 | Contig2894 | ATP-dependent zinc metalloprotease FTSH 2, chloroplastic |
| 468 | Contig2898 | 60S ribosomal protein L19-3 |
| 469 | Contig2901 | calmodulin-7-like isoform X2 |
| 470 | Contig2910 | E3 ubiquitin-protein ligase RHA1B-like |
| 471 | Contig2914 | histone H3.3 |
| 472 | Contig2916 | glutathione S-transferase T1-like |
| 473 | Contig2917 | 40S ribosomal protein S8 |
| 474 | Contig2920 | S-adenosylmethionine decarboxylase proenzyme |
| 475 | Contig2922 | lipid transfer protein |
| 476 | Contig2924 | 60S ribosomal protein L6 |
| 477 | Contig2934 | light-harvesting complex-like protein OHP2, chloroplastic |
| 478 | Contig2935 | transcription factor IBH1-like |
| 479 | Contig2937 | Localized to the inner membrane of the chloroplast |
| 480 | Contig2943 | cytochrome c oxidase subunit 6a, mitochondrial-like |
| 481 | Contig2945 | ABC transporter C family member 3-like |
| 482 | Contig2946 | 60S ribosomal protein L17-2 |
| 483 | Contig2960 | non-specific lipid-transfer protein 2 |
| 484 | Contig2962 | leucine-rich repeat protein 2-like |
| 485 | Contig2972 | expansin-like B1 |
| 486 | Contig2973 | zinc finger A20 and AN1 domain-containing stress-associated protein 5-like |
| 487 | Contig2975 | fructose-bisphosphate aldolase 3, chloroplastic |
| 488 | Contig2982 | 2,3-bisphosphoglycerate-independent phosphoglycerate mutase |
| 489 | Contig2990 | translational elongation factor Tu (chloroplast) |
| 490 | Contig2995 | S-adenosylmethionine decarboxylase proenzyme |
| 491 | Contig2998 | universal stress protein PHOS34-like |
| 492 | Contig3009 | palmitoyl-protein thioesterase 1-like |
| 493 | Contig3012 | respiratory burst oxidase homolog protein C-like |
| 494 | Contig3016 | zinc finger A20 and AN1 domain-containing stress-associated protein 8-like |
| 495 | Contig3018 | eukaryotic translation initiation factor 3 subunit G-like |
| 496 | Contig3021 | molybdopterin synthase sulfur carrier subunit |
| 497 | Contig3022 | chloroplast photosystem II PsbW protein |
| 498 | Contig3025 | RING-H2 finger protein ATL2-like |
| 499 | Contig3030 | thioredoxin-like protein Clot |
| 500 | Contig3031 | B-box zinc finger protein 24-like |
| 501 | Contig3034 | early nodulin-75-like |
| 502 | Contig3037 | cyprosin-like isoform X1 |
| 503 | Contig3042 | aspartate aminotransferase, cytoplasmic |
| 504 | Contig3048 | cytochrome c oxidase subunit 5C |
| 505 | Contig3049 | gibberellin-regulated protein 3-like |
| 506 | Contig3055 | tetraspanin-8-like |
| 507 | Contig3063 | 2-C-methyl-D-erythritol 2,4-cyclodiphosphate synthase, chloroplastic-like |
| 508 | Contig3069 | 30S ribosomal protein S13, chloroplastic-like |
| 509 | Contig3071 | probable small nuclear ribonucleoprotein G |
| 510 | Contig3082 | GTP-binding protein YPTM2 |
| 511 | Contig3094 | ras-related protein Rab11A |
| 512 | Contig3101 | temperature-induced lipocalin-1 |
| 513 | Contig3103 | ATP-dependent Clp protease proteolytic subunit 5, chloroplastic |
| 514 | Contig3105 | 60S ribosomal protein L15-1-like |
| 515 | Contig3107 | serine/threonine-protein kinase HT1 |
| 516 | Contig3111 | 2,3-bisphosphoglycerate-dependent phosphoglycerate mutase |
| 517 | Contig3120 | Splicing factor 3B subunit 5/RDS3 complex subunit 10 |
| 518 | Contig3122 | pentatricopeptide repeat-containing protein At3g59040 |
| 519 | Contig3124 | protein IWS1 homolog 1 |
| 520 | Contig3133 | UDP-glucuronate 4-epimerase 1 |
| 521 | Contig3136 | low-temperature-induced cysteine proteinase-like |
| 522 | Contig3137 | tonoplast dicarboxylate transporter |
| 523 | Contig3141 | Post-illumination chlorophyll fluorescence increase isoform 1 |
| 524 | Contig3142 | probable protein Pop3 |
| 525 | Contig3144 | phenylalanine ammonia-lyase |
| 526 | Contig3146 | vacuolar-sorting receptor 3-like |
| 527 | Contig3150 | non-specific lipid-transfer protein 1-like |
| 528 | Contig3151 | glycine-rich RNA-binding protein-like |
| 529 | Contig3152 | thioredoxin-like protein CDSP32, chloroplastic |
| 530 | Contig3153 | Elongation factor 1-alpha |
| 531 | Contig3154 | peroxidase 12-like |
| 532 | Contig3156 | 60S ribosomal protein L38 |
| 533 | Contig3158 | Octicosapeptide/Phox/Bem1p family protein isoform 1 |
| 534 | Contig3165 | 60S ribosomal protein L10a-2 |
| 535 | Contig3170 | p21-activated protein kinase-interacting protein 1-like |
| 536 | Contig3177 | squamous cell carcinoma antigen recognized by T-cells 3 |
| 537 | Contig3179 | isocitrate dehydrogenase [NAD] catalytic subunit 5, mitochondrial |
| 538 | Contig3180 | glutaredoxin |
| 539 | Contig3185 | mediator of RNA polymerase II transcription subunit 10b-like isoform X1 |
| 540 | Contig3187 | hypothetical protein MANES_13G084800 |
| 541 | Contig3189 | granule-bound starch synthase 1, chloroplastic/amyloplastic |
| 542 | Contig3193 | WD repeat-containing protein LWD1 |
| 543 | Contig3194 | digalactosyldiacylglycerol synthase 2, chloroplastic |
| 544 | Contig3196 | chlorophyll a-b binding protein, chloroplastic |
| 545 | Contig3197 | sm-like protein LSM36B |
| 546 | Contig3199 | 60S ribosomal protein L24 |
| 547 | Contig3204 | pollen-specific protein C13-like |
| 548 | Contig3208 | mitochondrial import receptor subunit TOM9-2-like |
| 549 | Contig3212 | peroxidase 42 |
| 550 | Contig3219 | ubiquitin-conjugating enzyme E2 36 |
| 551 | Contig3235 | serine carboxypeptidase-like |
| 552 | Contig3236 | polyadenylate-binding protein RBP47-like |
| 553 | Contig3248 | syntaxin-71-like |
| 554 | Contig3251 | phospho-2-dehydro-3-deoxyheptonate aldolase 1, chloroplastic-like |
| 555 | Contig3259 | chlorophyll a-b binding protein P4, chloroplastic |
| 556 | Contig3263 | probable inactive purple acid phosphatase 29 |
| 557 | Contig3267 | ubiquitin carboxyl-terminal hydrolase 24 isoform X1 |
| 558 | Contig3270 | alpha-galactosidase 3 |
| 559 | Contig3278 | phosphatidylinositol 4-kinase gamma 5-like |
| 560 | Contig3281 | UDP-glucose 6-dehydrogenase 1 |
| 561 | Contig3282 | multiple organellar RNA editing factor 8, chloroplastic/mitochondrial-like |
| 562 | Contig3290 | ---NA--- |
| 563 | Contig3292 | hypothetical protein MANES_01G135100 |
| 564 | Contig3295 | rve domain-containing protein/RVT_3 domain-containing protein |
| 565 | Contig3298 | plastocyanin |
| 566 | Contig3306 | 1,2-dihydroxy-3-keto-5-methylthiopentene dioxygenase 2 |
| 567 | Contig3312 | cinnamoyl-CoA reductase 1-like |
| 568 | Contig3313 | THO complex subunit 4D-like isoform X1 |
| 569 | Contig3324 | hypothetical protein MANES_S069800 |
| 570 | Contig3330 | fructose-bisphosphate aldolase 1, chloroplastic |
| 571 | Contig3334 | immune-associated nucleotide-binding protein 9 |
| 572 | Contig3335 | 5-methyltetrahydropteroyltriglutamate--homocysteine methyltransferase |
| 573 | Contig3336 | hypothetical protein MANES_13G027400 |
| 574 | Contig3341 | chlorophyll a-b binding protein 13, chloroplastic |
| 575 | Contig3343 | protochlorophyllide reductase, chloroplastic |
| 576 | Contig3344 | galactinol synthase 1 |
| 577 | Contig3352 | low-temperature-induced cysteine proteinase-like |
| 578 | Contig3355 | 40S ribosomal protein S23 |
| 579 | Contig3359 | Tetratricopeptide-like helical domain containing protein |
| 580 | Contig3361 | glycine-rich RNA-binding protein |
| 581 | Contig3363 | actin-depolymerizing factor 2 |
| 582 | Contig3366 | chalcone synthase |
| 583 | Contig3367 | photosystem I chlorophyll a/b-binding protein 6, chloroplastic |
| 584 | Contig3370 | UDP-N-acetylglucosamine diphosphorylase 2-like |
| 585 | Contig3372 | 5'-adenylylsulfate reductase 3, chloroplastic-like |
| 586 | Contig3373 | uncharacterized protein LOC110617626 |
| 587 | Contig3384 | bifunctional protein FolD 2 |
| 588 | Contig3390 | ribonuclease P protein subunit p25-like protein |
| 589 | Contig3392 | 60S ribosomal protein L31 |
| 590 | Contig3393 | MAPK kinase substrate protein At1g80180 |
| 591 | Contig3397 | uncharacterized protein LOC110617791 |
| 592 | Contig3406 | persulfide dioxygenase ETHE1 homolog, mitochondrial |
| 593 | Contig3407 | calreticulin-3-like |
| 594 | Contig3412 | MADS-box protein SVP isoform X1 |
| 595 | Contig3414 | non-specific phospholipase C1 |
| 596 | Contig3416 | membrane steroid-binding protein 2-like |
| 597 | Contig3422 | O-fucosyltransferase family protein isoform 1 |
| 598 | Contig3425 | acyl-CoA-binding domain-containing protein 1 |
| 599 | Contig3427 | enolase 2 |
| 600 | Contig3433 | probable protein disulfide-isomerase A6 |
| 601 | Contig3435 | NDR1/HIN1-like protein 1 |
| 602 | Contig3436 | protein DELETION OF SUV3 SUPPRESSOR 1(I)-like |
| 603 | Contig3443 | Leucine-rich repeat structural protein ORF147 isoform 1 |
| 604 | Contig3453 | 3'-5' exoribonuclease 1-like |
| 605 | Contig3458 | dihydroflavonol 4-reductase-like |
| 606 | Contig3464 | elongation factor 1-beta 2-like |
| 607 | Contig3469 | Transmembrane protein |
| 608 | Contig3472 | photosystem I reaction center subunit II, chloroplastic-like |
| 609 | Contig3473 | peroxidase 42 |
| 610 | Contig3477 | S-adenosyl-L-homocysteine hydrolase |
| 611 | Contig3480 | transmembrane ascorbate ferrireductase 1 |
| 612 | Contig3482 | eukaryotic initiation factor 4A-8 |
| 613 | Contig3483 | ---NA--- |
| 614 | Contig3488 | ADP,ATP carrier protein 1, mitochondrial-like |
| 615 | Contig3489 | chalcone synthase 1-like |
| 616 | Contig3490 | long-chain-alcohol oxidase FAO2 |
| 617 | Contig3494 | very-long-chain enoyl-CoA reductase |
| 618 | Contig3517 | adenosylhomocysteinase |
| 619 | Contig3518 | phospho-2-dehydro-3-deoxyheptonate aldolase 2, chloroplastic |
| 620 | Contig3523 | Interferon-induced with tetratricopeptide repeats 2 |
| 621 | Contig3528 | Ubiquitin system component Cue protein, putative isoform 2 |
| 622 | Contig3531 | glycine-rich RNA-binding protein-like |
| 623 | Contig3537 | probable glutathione S-transferase |
| 624 | Contig3541 | NDR1/HIN1-like protein 1 |
| 625 | Contig3546 | MICOS complex subunit mic60 |
| 626 | Contig3548 | zinc finger CCCH domain-containing protein 37 isoform X1 |
| 627 | Contig3553 | S-adenosylmethionine synthase 1 |
| 628 | Contig3560 | probable serine incorporator |
| 629 | Contig3561 | actin-7 |
| 630 | Contig3562 | hevamine-A |
| 631 | Contig3565 | iron-sulfur cluster assembly protein 1-like |
| 632 | Contig3572 | dihydrolipoyl dehydrogenase 1, mitochondrial |
| 633 | Contig3577 | probable E3 ubiquitin-protein ligase XERICO |
| 634 | Contig3585 | 5-methyltetrahydropteroyltriglutamate--homocysteine methyltransferase |
| 635 | Contig3593 | multiprotein-bridging factor 1c |
| 636 | Contig3597 | adenosylhomocysteinase 1-like |
| 637 | Contig3604 | metalloendoproteinase 3-MMP-like |
| 638 | Contig3609 | soluble inorganic pyrophosphatase-like |
| 639 | Contig3613 | 60S ribosomal protein L36-2-like |
| 640 | Contig3615 | uncharacterized protein At5g64816-like |
| 641 | Contig3618 | L-ascorbate peroxidase, cytosolic |
| 642 | Contig3620 | UV-B-induced protein At3g17800, chloroplastic-like |
| 643 | Contig3621 | WAT1-related protein At4g08300-like |
| 644 | Contig3623 | probable magnesium transporter NIPA6 |
| 645 | Contig3627 | uncharacterized protein LOC110609887 |
| 646 | Contig3629 | Malectin/receptor protein kinase family protein |
| 647 | Contig3630 | glutathione S-transferase U17-like |
| 648 | Contig3632 | dormancy-associated protein homolog 3 isoform X1 |
| 649 | Contig3637 | ADP,ATP carrier protein 1, mitochondrial |
| 650 | Contig3642 | cysteine proteinase 15A-like |
| 651 | Contig3655 | mRNA turnover protein 4 homolog |
| 652 | Contig3656 | cysteine proteinase inhibitor-like |
| 653 | Contig3660 | BURP domain protein RD22 |
| 654 | Contig3661 | chaperonin 60 subunit beta 2, chloroplastic |
| 655 | Contig3662 | probable glutathione S-transferase |
| 656 | Contig3665 | pectin acetylesterase 7 |
| 657 | Contig3666 | DNA-damage-repair/toleration protein DRT100-like |
| 658 | Contig3669 | 40S ribosomal protein S12-like |
| 659 | Contig3676 | 60S ribosomal protein L10 |
| 660 | Contig3677 | transaldolase family protein |
| 661 | Contig3678 | light-regulated protein-like |
| 662 | Contig3681 | galactinol synthase 1 |
| 663 | Contig3686 | 40S ribosomal protein S5 |
| 664 | Contig3687 | protein C2-DOMAIN ABA-RELATED 4-like |
| 665 | Contig3689 | CBL-interacting serine/threonine-protein kinase 4-like |
| 666 | Contig3690 | vesicle-associated protein 4-1-like |
| 667 | Contig3715 | F-box protein At5g46170-like |
| 668 | Contig3717 | protein decapping 5 |
| 669 | Contig3720 | probable E3 ubiquitin-protein ligase BAH1-like 1 |
| 670 | Contig3723 | alpha-1,4-glucan-protein synthase [UDP-forming] 2 |
| 671 | Contig3731 | Cyclic nucleotide-gated ion channel 4 |
| 672 | Contig3742 | protein TIFY 3B-like isoform X1 |
| 673 | Contig3743 | oxygen-evolving enhancer protein 3-2, chloroplastic-like |
| 674 | Contig3744 | hypothetical protein MANES_06G170800 |
| 675 | Contig3746 | probable nucleoredoxin 2 |
| 676 | Contig3749 | photosystem II 5 kDa protein, chloroplastic-like |
| 677 | Contig3755 | 60S ribosomal protein L6 |
| 678 | Contig3757 | catalase isozyme 1 |
| 679 | Contig3760 | uncharacterized protein LOC110619953 |
| 680 | Contig3761 | cysteine synthase-like isoform X1 |
| 681 | Contig3770 | senescence-associated family protein |
| 682 | Contig3771 | nucleolin 1-like isoform X1 |
| 683 | Contig3787 | 60S ribosomal protein L10 |
| 684 | Contig3806 | DNA-directed RNA polymerases II, IV and V subunit 11 |
| 685 | Contig3818 | EIN3-binding F-box protein 1-like |
| 686 | Contig3820 | vacuolar protein sorting-associated protein 18 homolog |
| 687 | Contig3821 | fructose-1,6-bisphosphatase, chloroplastic |
| 688 | Contig3839 | DUF4228 domain-containing protein |
| 689 | Contig3841 | 5'-adenylylsulfate reductase 3, chloroplastic-like |
| 690 | Contig3842 | thioredoxin-like 2, chloroplastic |
| 691 | Contig3845 | Transmembrane protein |
| 692 | Contig3854 | polyubiquitin |
| 693 | Contig3856 | protein ASPARTIC PROTEASE IN GUARD CELL 1-like |
| 694 | Contig3857 | putative LOV domain-containing protein |
| 695 | Contig3859 | uncharacterized protein LOC110620763 |
| 696 | Contig3863 | zinc finger protein 593 |
| 697 | Contig3868 | vacuolar cation/proton exchanger 3 |
| 698 | Contig3869 | small nuclear ribonucleoprotein E-like |
| 699 | Contig3873 | fructose-bisphosphate aldolase 1, chloroplastic |
| 700 | Contig3876 | cellulose synthase-like protein E1 |
| 701 | Contig3878 | ethylene-responsive transcription factor 3-like |
| 702 | Contig3880 | chlorophyll a-b binding protein P4, chloroplastic |
| 703 | Contig3881 | DNA-damage-repair/toleration protein DRT102 |
| 704 | Contig3884 | 60S ribosomal protein L26-1 |
| 705 | Contig3885 | peroxisomal membrane protein 11C |
| 706 | Contig3891 | calmodulin-like protein 5 |
| 707 | Contig3892 | S-adenosyl-l-homocysteine hydrolase A |
| 708 | Contig3898 | kynurenine formamidase |
| 709 | Contig3901 | 60s ribosomal protein l39-1 |
| 710 | Contig3903 | DUF1517 domain-containing protein |
| 711 | Contig3909 | cytochrome b-c1 complex subunit Rieske-4, mitochondrial-like |
| 712 | Contig3911 | probable NADH dehydrogenase [ubiquinone] 1 alpha subcomplex subunit 5, mitochondrial |
| 713 | Contig3912 | 60S ribosomal protein L44 |
| 714 | Contig3921 | plastidic ATP/ADP-transporter-like |
| 715 | Contig3927 | protochlorophyllide reductase, chloroplastic |
| 716 | Contig3944 | calcium-dependent protein kinase 26 isoform X2 |
| 717 | Contig3945 | metallothionein-like protein type 2 |
| 718 | Contig3948 | ---NA--- |
| 719 | Contig3959 | carbonic anhydrase, chloroplastic-like isoform X2 |
| 720 | Contig3962 | putative lipid-transfer protein DIR1 |
| 721 | Contig3963 | 60S ribosomal protein L3-2 |
| 722 | Contig3966 | 23.6 kDa heat shock protein, mitochondrial-like isoform X2 |
| 723 | Contig3967 | 60S ribosomal protein L23 |
| 724 | Contig3970 | (S)-hydroxynitrile lyase-like |
| 725 | Contig3971 | proteasome subunit alpha type-7 |
| 726 | Contig3973 | NAC domain-containing protein 2 |
| 727 | Contig3991 | UDP-sulfoquinovose synthase |
| 728 | Contig3996 | 60S ribosomal protein L11 |
| 729 | Contig3998 | phospholipid hydroperoxide glutathione peroxidase 1, chloroplastic |
| 730 | Contig4000 | BI1-like protein |
| 731 | Contig4001 | probable serine/threonine-protein kinase ndrD |
| 732 | Contig4003 | 60S ribosomal protein L31 |
| 733 | Contig4004 | serine/threonine-protein kinase SRK2I |
| 734 | Contig4008 | serine/threonine-protein kinase D6PK-like |
| 735 | Contig4011 | high mobility group B protein 3-like |
| 736 | Contig4015 | heptahelical transmembrane protein 4-like |
| 737 | Contig4019 | (S)-hydroxynitrile lyase-like |
| 738 | Contig4046 | tubulin beta-5 chain |
| 739 | Contig4057 | glyceraldehyde-3-phosphate dehydrogenase, cytosolic |
| 740 | Contig4059 | 60S ribosomal protein L4 |
| 741 | Contig4063 | flowering time control protein FCA-like isoform X3 |
| 742 | Contig4065 | translationally-controlled tumor protein homolog |
| 743 | Contig4070 | glycine-rich RNA-binding protein RZ1A |
| 744 | Contig4071 | cyclic AMP-responsive element-binding protein |
| 745 | Contig4082 | chlorophyll a-b binding protein of LHCII type 1 |
| 746 | Contig4083 | polyadenylate-binding protein RBP45C-like isoform X1 |
| 747 | Contig4087 | proteasome subunit alpha type-2-A |
| 748 | Contig4089 | profilin-1 |
| 749 | Contig4092 | probable signal peptidase complex subunit 2 |
| 750 | Contig4096 | 3-isopropylmalate dehydratase small subunit 3-like |
| 751 | Contig4102 | protein ETHYLENE INSENSITIVE 3-like |
| 752 | Contig4110 | glycine-rich RNA-binding protein 2, mitochondrial-like |
| 753 | Contig4112 | auxin-repressed 12.5 kDa protein-like isoform X2 |
| 754 | Contig4114 | peroxisomal membrane protein PMP22 |
| 755 | Contig4119 | calvin cycle protein CP12-2, chloroplastic |
| 756 | Contig4120 | thioredoxin H-type |
| 757 | Contig4125 | L-ascorbate peroxidase, cytosolic |
| 758 | Contig4126 | 40s ribosomal protein s16 |
| 759 | Contig4129 | inner membrane protein |
| 760 | Contig4131 | 40S ribosomal protein SA-like isoform X1 |
| 761 | Contig4132 | 60S ribosomal protein L9 |
| 762 | Contig4133 | peptidyl-prolyl cis-trans isomerase CYP20-1-like |
| 763 | Contig4138 | arginine decarboxylase-like |
| 764 | Contig4144 | 60S acidic ribosomal protein P3-like |
| 765 | Contig4154 | peptidyl-prolyl cis-trans isomerase FKBP12 |
| 766 | Contig4155 | NADH dehydrogenase [ubiquinone] 1 alpha subcomplex subunit 13-B |
| 767 | Contig4157 | Regulator of rDNA transcription protein 15 |
| 768 | Contig4158 | vacuolar protein sorting-associated protein 25 |
| 769 | Contig4162 | Protein TAR1 |
| 770 | Contig4165 | 17.3 kDa class I heat shock protein-like |
| 771 | Contig4167 | nucleobase-ascorbate transporter 6 |
| 772 | Contig4173 | V-type proton ATPase 16 kDa proteolipid subunit |
| 773 | Contig4175 | light-harvesting complex-like protein 3 isotype 1, chloroplastic |
| 774 | Contig4177 | FRIGIDA-like protein 3 |
| 775 | Contig4179 | 26S proteasome non-ATPase regulatory subunit 14 homolog |
| 776 | Contig4181 | transcription factor TCP9 |
| 777 | Contig4182 | malignant T-cell-amplified sequence 1 homolog |
| 778 | Contig4185 | molybdopterin synthase catalytic subunit |
| 779 | Contig4188 | photosystem I reaction center subunit XI, chloroplastic |
| 780 | Contig4189 | NADH-ubiquinone oxidoreductase 20.9 kDa subunit |
| 781 | Contig4190 | ATP synthase subunit beta, mitochondrial |
| 782 | Contig4202 | 60S ribosomal protein L30 |
| 783 | Contig4213 | ethylene-responsive transcription factor 4 |
| 784 | Contig4219 | phospho-2-dehydro-3-deoxyheptonate aldolase 2, chloroplastic |
| 785 | Contig4223 | arginine decarboxylase |
| 786 | Contig4227 | metal-nicotianamine transporter YSL3 isoform X1 |
| 787 | Contig4230 | heat shock 70 kDa protein |
| 788 | Contig4241 | probable histone H2A.1 |
| 789 | Contig4243 | cytochrome oxidase assembly protein |
| 790 | Contig4244 | Transferring glycosyl groups, putative |
| 791 | Contig4245 | 2-methylbutanal oxime monooxygenase-like |
| 792 | Contig4248 | glyceraldehyde-3-phosphate dehydrogenase, cytosolic |
| 793 | Contig4255 | pyrrolidone-carboxylate peptidase 1 |
| 794 | Contig4264 | ethylene-responsive transcription factor RAP2-4 |
| 795 | Contig4265 | pectin acetylesterase 7 |
| 796 | Contig4266 | stress response protein NST1 |
| 797 | Contig4274 | ---NA--- |
| 798 | Contig4275 | secoisolariciresinol dehydrogenase-like |
| 799 | Contig4277 | photosystem I reaction center subunit VI, chloroplastic |
| 800 | Contig4279 | cytochrome c1-2, heme protein, mitochondrial |
| 801 | Contig4283 | glutathione S-transferase U7-like |
| 802 | Contig4290 | cysteine proteinase RD19a-like |
| 803 | Contig4306 | peptidyl-prolyl cis-trans isomerase CYP18-2 |
| 804 | Contig4314 | pto-interacting protein 1 |
| 805 | Contig4316 | aquaporin TIP1-1 |
| 806 | Contig4319 | 30S ribosomal protein S10, chloroplastic-like |
| 807 | Contig4321 | MFP1 attachment factor 1-like |
| 808 | Contig4326 | BTB/POZ and TAZ domain-containing protein 1-like |
| 809 | Contig4329 | bark storage protein A |
| 810 | Contig4332 | probable WRKY transcription factor 12 |
| 811 | Contig4334 | aquaporin TIP1-1 |
| 812 | Contig4340 | CBL-interacting protein kinase 2-like |
| 813 | Contig4350 | ribulose bisphosphate carboxylase small chain, chloroplastic-like |
| 814 | Contig4357 | ferredoxin-thioredoxin reductase, variable chain, chloroplastic-like |
| 815 | Contig4359 | uncharacterized protein LOC110621397 |
| 816 | Contig4363 | protein transport protein Sec61 subunit alpha-like |
| 817 | Contig4364 | 60S ribosomal protein L27-3-like |
| 818 | Contig4368 | 40S ribosomal protein S9-2-like |
| 819 | Contig4370 | hypothetical protein MANES_01G231900 |
| 820 | Contig4377 | uncharacterized protein LOC110628626 |
| 821 | Contig4386 | GTP-binding nuclear protein Ran-3-like |
| 822 | Contig4387 | cysteine protease RD19A-like |
| 823 | Contig4388 | serine/threonine-protein kinase HT1 |
| 824 | Contig4390 | uncharacterized protein LOC110605779 |
| 825 | Contig4405 | mitogen-activated protein kinase homolog NTF3-like |
| 826 | Contig4407 | uncharacterized protein OsI_027940-like |
| 827 | Contig4410 | synaptotagmin-4-like isoform X2 |
| 828 | Contig4411 | serine/threonine-protein kinase HT1-like |
| 829 | Contig4416 | arginine decarboxylase-like |
| 830 | Contig4433 | Lactoylglutathione lyase / glyoxalase I family protein |
| 831 | Contig4440 | NAD(P)-binding Rossmann-fold superfamily protein isoform 1 |
| 832 | Contig4442 | hypothetical protein MANES_10G034100 |
| 833 | Contig4447 | tRNA-splicing ligase RtcB |
| 834 | Contig4448 | chlorophyll a-b binding protein 151, chloroplastic-like |
| 835 | Contig4455 | ribonuclease P protein subunit p25-like protein |
| 836 | Contig4463 | enolase |
| 837 | Contig4473 | G-type lectin S-receptor-like serine/threonine-protein kinase At1g34300 |
| 838 | Contig4474 | cytochrome P450 CYP82D47-like |
| 839 | Contig4480 | DNA damage-repair/toleration protein DRT102 |
| 840 | Contig4481 | ---NA--- |
| 841 | Contig4497 | oxygen-evolving enhancer protein 2, chloroplastic |
| 842 | Contig4507 | actin-depolymerizing factor |
| 843 | Contig4510 | UDP-glucose 6-dehydrogenase 1 |
| 844 | Contig4514 | heat shock cognate 70 kDa protein 2-like |
| 845 | Contig4523 | sm-like protein LSM7 |
| 846 | Contig4533 | auxin transporter-like protein 2 |
| 847 | Contig4544 | dual specificity phosphatase Cdc25 |
| 848 | Contig4553 | GDP-L-galactose phosphorylase 2-like |
| 849 | Contig4556 | early light-induced protein, chloroplastic |
| 850 | Contig4560 | uncharacterized protein LOC110618503 |
| 851 | Contig4562 | 60S ribosomal protein L17-2 |
| 852 | Contig4564 | 50S ribosomal protein L19-1, chloroplastic |
| 853 | Contig4572 | ring finger protein 141 |
| 854 | Contig4576 | 60S ribosomal protein L13a-4 |
| 855 | Contig4580 | photosystem II 22 kDa protein, chloroplastic-like |
| 856 | Contig4581 | triose phosphate/phosphate translocator, chloroplastic isoform X1 |
| 857 | Contig4582 | plant cysteine oxidase 2-like |
| 858 | Contig4583 | cystinosin homolog |
| 859 | Contig4584 | classical arabinogalactan protein 4-like |
| 860 | Contig4586 | chitinase-like protein 1 |
| 861 | Contig4598 | Zim17-type zinc finger protein |
| 862 | Contig4603 | chalcone synthase |
| 863 | Contig4604 | chloroplast envelope quinone oxidoreductase homolog |
| 864 | Contig4619 | upstream activation factor subunit UAF30 |
| 865 | Contig4620 | cysteine synthase |
| 866 | Contig4622 | phospho-2-dehydro-3-deoxyheptonate aldolase 1, chloroplastic-like |
| 867 | Contig4624 | probable sodium/metabolite cotransporter BASS3, chloroplastic |
| 868 | Contig4625 | hypothetical protein POPTR_001G141500v3 |
| 869 | Contig4626 | 1-aminocyclopropane-1-carboxylate oxidase |
| 870 | Contig4630 | proline-rich receptor-like protein kinase PERK15 |
| 871 | Contig4644 | non-specific phospholipase C1 |
| 872 | Contig4658 | ---NA--- |
| 873 | Contig4666 | 26S proteasome non-ATPase regulatory subunit 13 homolog B |
| 874 | Contig4671 | 40S ribosomal protein S29 |
| 875 | Contig4677 | ER membrane protein complex subunit 3-like |
| 876 | Contig4680 | probable methyltransferase PMT24 |
| 877 | Contig4686 | 40S ribosomal protein S15 |
| 878 | Contig4692 | heat shock cognate 70 kDa protein 2 |
| 879 | Contig4696 | stem-specific protein TSJT1-like |
| 880 | Contig4699 | Wound-responsive family protein |
| 881 | Contig4701 | 10 kDa chaperonin-like |
| 882 | Contig4704 | Trigger_N domain-containing protein |
| 883 | Contig4708 | OTU domain-containing protein At3g57810-like |
| 884 | Contig4717 | CBL-interacting serine/threonine-protein kinase 6-like |
| 885 | Contig4720 | 60S ribosomal protein L11 |
| 886 | Contig4721 | alpha tubulin 1 |
| 887 | Contig4723 | long-chain-alcohol oxidase FAO2 |
| 888 | Contig4742 | calcium-binding EF hand family protein |
| 889 | Contig4750 | geranylgeranyl diphosphate reductase, chloroplastic |
| 890 | Contig4755 | gamma aminobutyrate transaminase 3, chloroplastic-like |
| 891 | Contig4770 | photosystem II core complex proteins psbY, chloroplastic-like |
| 892 | Contig4775 | calnexin homolog |
| 893 | Contig4782 | dolichol-phosphate mannosyltransferase subunit 3-like |
| 894 | Contig4784 | zinc finger protein 706 |
| 895 | Contig4787 | ADP-ribosylation factor |
| 896 | Contig4789 | galactan beta-1,4-galactosyltransferase GALS3-like |
| 897 | Contig4794 | serine-threonine kinase receptor-associated protein-like |
| 898 | Contig4803 | 60S acidic ribosomal protein P1-like |
| 899 | Contig4804 | probable histone H2B.3 |
| 900 | Contig4805 | nucleobase-ascorbate transporter 6 |
| 901 | Contig4806 | mitotic-spindle organizing protein 1A-like isoform X3 |
| 902 | Contig4808 | oxygen-evolving enhancer protein 1, chloroplastic |
| 903 | Contig4812 | esterase-like |
| 904 | Contig4813 | galactinol synthase 1 |
| 905 | Contig4819 | peroxisomal membrane protein 13 |
| 906 | Contig4871 | Homeobox protein knotted-1-like 3 |
| 907 | Contig4874 | sorbitol dehydrogenase |
| 908 | Contig4895 | stem-specific protein TSJT1-like |
| 909 | Contig4898 | dormancy-associated protein homolog 3 isoform X1 |
| 910 | Contig4899 | soluble inorganic pyrophosphatase 4 |
| 911 | Contig4901 | regulatory protein NPR3-like |
| 912 | Contig4907 | Polyketide cyclase/dehydrase and lipid transport superfamily protein |
| 913 | Contig4919 | 60S ribosomal protein L6 |
| 914 | Contig4924 | protein SLOW GREEN 1, chloroplastic-like |
| 915 | Contig4930 | monoglyceride lipase-like |
| 916 | Contig4936 | 60S ribosomal protein L18a-like protein isoform X1 |
| 917 | Contig4942 | ATP synthase subunit O, mitochondrial |
| 918 | Contig4949 | 60S ribosomal protein L7-2-like |
| 919 | Contig4952 | Glycine-rich protein, putative isoform 1 |
| 920 | Contig4955 | thiamine thiazole synthase, chloroplastic |
| 921 | Contig4963 | REF/SRPP-like protein At3g05500 |
| 922 | Contig4969 | copper transport protein ATX1-like isoform X2 |
| 923 | Contig4971 | Fiber protein Fb15 |
| 924 | Contig4973 | scarecrow-like protein 13 |
| 925 | Contig4975 | chlorophyll synthase, chloroplastic |
| 926 | Contig4987 | histidine-containing phosphotransfer protein 1-like |
| 927 | Contig4988 | germin-like protein subfamily 3 member 1 |
| 928 | Contig4993 | 40S ribosomal protein S13 |
| 929 | Contig4998 | 60S acidic ribosomal protein P2B-like |
| 930 | Contig5007 | fumarate hydratase 1, mitochondrial |
| 931 | Contig5014 | 40S ribosomal protein S10-1-like |
| 932 | Contig5015 | thiamine thiazole synthase, chloroplastic-like |
| 933 | Contig5016 | vestitone reductase-like |
| 934 | Contig5021 | 40S ribosomal protein S20-2 |
| 935 | Contig5022 | eukaryotic initiation factor 4A-15 |
| 936 | Contig5026 | methyl-CpG-binding domain-containing protein 11-like |
| 937 | Contig5027 | hypothetical protein MANES_09G100500 |
| 938 | Contig5028 | 60S ribosomal protein L13-1-like |
| 939 | Contig5036 | B2 protein |
| 940 | Contig5039 | chlorophyll a-b binding protein of LHCII type 1 |
| 941 | Contig5050 | probable xyloglucan endotransglucosylase/hydrolase protein 28 |
| 942 | Contig5084 | protein DCL homolog, chloroplastic |
| 943 | Contig5089 | pentatricopeptide repeat-containing protein At3g06430, chloroplastic |
| 944 | Contig5108 | cytochrome b-c1 complex subunit 7-2 |
| 945 | Contig5111 | 40S ribosomal protein S4-1 |
| 946 | Contig5113 | uncharacterized protein LOC110616094 |
| 947 | Contig5125 | nascent polypeptide-associated complex subunit beta-like |
| 948 | Contig5134 | GDP-L-galactose phosphorylase 2 |
| 949 | Contig5139 | 1-aminocyclopropane-1-carboxylate oxidase homolog 4-like |
| 950 | Contig5148 | CBL-interacting protein kinase 2-like |
| 951 | Contig5150 | programmed cell death protein 4-like |
| 952 | Contig5166 | Hypoxia induced protein, domain containing protein |
| 953 | Contig5181 | outer envelope membrane protein 7-like |
| 954 | Contig5193 | fructose-bisphosphate aldolase 1, chloroplastic |
| 955 | Contig5196 | heat shock cognate 70 kDa protein 2 |
| 956 | Contig5197 | glycine-rich RNA-binding protein-like |
| 957 | Contig5198 | remorin-like |
| 958 | Contig5201 | 36.4 kDa proline-rich protein-like |
| 959 | Contig5206 | probable nucleoredoxin 1 |
| 960 | Contig5207 | glyceraldehyde-3-phosphate dehydrogenase B, chloroplastic |
| 961 | Contig5208 | thioredoxin-like protein HCF164, chloroplastic |
| 962 | Contig5210 | 4-coumarate--CoA ligase 1-like |
| 963 | Contig5216 | nematode resistance protein-like HSPRO2 |
| 964 | Contig5217 | ATP-dependent Clp protease proteolytic subunit-related protein 2, chloroplastic |
| 965 | Contig5218 | sedoheptulose-1,7-bisphosphatase, chloroplastic-like |
| 966 | Contig5220 | putative RING-type E3 ubiquitin transferase C3H69 isoform X1 |
| 967 | Contig5221 | nucleoid-associated protein At4g30620, chloroplastic-like |
| 968 | Contig5222 | putative methyltransferase DDB_G0268948 |
| 969 | Contig5227 | uricase-2 isozyme 2 |
| 970 | Contig5229 | ATP synthase subunit epsilon, mitochondrial |
| 971 | Contig5231 | protein EARLY RESPONSIVE TO DEHYDRATION 15-like |
| 972 | Contig5232 | 60S ribosomal protein L36-2 |
| 973 | Contig5237 | cysteine and histidine-rich domain-containing protein RAR1 |
| 974 | Contig5240 | probable arabinosyltransferase ARAD1 |
| 975 | Contig5242 | pyrophosphatase |
| 976 | Contig5244 | ---NA--- |
| 977 | Contig5251 | 60S ribosomal protein L7a-1-like |
| 978 | Contig5256 | 60S ribosomal protein L21-1 |
| 979 | Contig5258 | katanin p60 ATPase-containing subunit A1 |
| 980 | Contig5268 | polyubiquitin |
| 981 | Contig5269 | nitric oxide synthase-interacting protein-like |
| 982 | Contig5275 | syntaxin-71-like |
| 983 | Contig5277 | CAX-interacting protein 4 |
| 984 | Contig5278 | ER membrane protein complex subunit 4-like |
| 985 | Contig5281 | photosystem I reaction center subunit III, chloroplastic |
| 986 | Contig5286 | dof zinc finger protein DOF5.4-like |
| 987 | Contig5289 | UBP1-associated protein 2C-like |
| 988 | Contig5295 | zinc finger CCCH domain-containing protein 20-like |
| 989 | Contig5300 | oligouridylate-binding protein 1B-like isoform X1 |
| 990 | Contig5302 | non-symbiotic hemoglobin 1 |
| 991 | Contig5303 | probable CCR4-associated factor 1 homolog 6 |
| 992 | Contig5307 | 60S ribosomal protein L12 |
| 993 | Contig5323 | cyclin-B1-2-like |
| 994 | Contig5331 | gamma carbonic anhydrase-like 2, mitochondrial |
| 995 | Contig5333 | 40S ribosomal protein S6 |
| 996 | Contig5337 | magnesium-dependent phosphatase 1 |
| 997 | Contig5341 | phosphoenolpyruvate carboxykinase [ATP]-like |
| 998 | Contig5346 | potassium channel AKT1-like |
| 999 | Contig5350 | UPF0051 protein ABCI8, chloroplastic |
| 1000 | Contig5353 | protein MAINTENANCE OF PSII UNDER HIGH LIGHT 1 |
| 1001 | Contig5365 | vesicle-associated membrane protein 714 |
| 1002 | Contig5368 | heat shock protein 90-5, chloroplastic |
| 1003 | Contig5371 | sucrose synthase 2 |
| 1004 | Contig5377 | phosphatidylinositol/phosphatidylcholine transfer protein SFH9-like |
| 1005 | Contig5394 | AT-rich interactive domain protein |
| 1006 | Contig5399 | PP2A regulatory subunit TAP46 |
| 1007 | Contig5408 | transcription factor DIVARICATA |
| 1008 | Contig5416 | heme-binding protein 2-like |
| 1009 | Contig5436 | uncharacterized protein LOC110626720 |
| 1010 | Contig5440 | uncharacterized protein LOC110626720 |
| 1011 | Contig5443 | triosephosphate isomerase, cytosolic |
| 1012 | Contig5454 | glutathione S-transferase F9-like |
| 1013 | Contig5455 | ER membrane protein complex subunit 3 |
| 1014 | Contig5458 | glycine-rich RNA-binding protein 2, mitochondrial-like |
| 1015 | Contig5470 | metallothionein-like protein type 2 |
| 1016 | Contig5474 | probable E3 ubiquitin-protein ligase RNF217 |
| 1017 | Contig5481 | malate dehydrogenase, glyoxysomal |
| 1018 | Contig5483 | 40S ribosomal protein S12-like |
| 1019 | Contig5488 | 5'-methylthioadenosine/S-adenosylhomocysteine nucleosidase 1-like |
| 1020 | Contig5489 | protein TRIGALACTOSYLDIACYLGLYCEROL 5, chloroplastic |
| 1021 | Contig5491 | prostatic spermine-binding protein-like |
| 1022 | Contig5495 | uncharacterized calcium-binding protein At1g02270-like isoform X1 |
| 1023 | Contig5498 | NDR1/HIN1-like protein 1 |
| 1024 | Contig5502 | ---NA--- |
| 1025 | Contig5504 | ATP binding protein, putative |
| 1026 | Contig5509 | protein E6-like |
| 1027 | Contig5522 | serine hydroxymethyltransferase 3, chloroplastic-like |
| 1028 | Contig5524 | putative E3 ubiquitin-protein ligase XBAT35 isoform X1 |
| 1029 | Contig5526 | methylenetetrahydrofolate reductase 2-like |
| 1030 | Contig5534 | protein DOWNY MILDEW RESISTANCE 6-like |
| 1031 | Contig5536 | photosystem I reaction center subunit IV A, chloroplastic |
| 1032 | Contig5539 | probable calcium-binding protein CML36 |
| 1033 | Contig5545 | bifunctional epoxide hydrolase 2-like |
| 1034 | Contig5546 | translationally-controlled tumor protein homolog |
| 1035 | Contig5554 | galactinol synthase 1 |
| 1036 | Contig5555 | reticulon-like protein B2 |
| 1037 | Contig5556 | elongation factor 1-alpha |
| 1038 | Contig5559 | RGG repeats nuclear RNA binding protein A-like isoform X1 |
| 1039 | Contig5571 | abscisic acid receptor PYL8 |
| 1040 | Contig5578 | 3-oxo-Delta(4,5)-steroid 5-beta-reductase-like |
| 1041 | Contig5582 | 14-3-3-like protein A |
| 1042 | Contig5584 | 60S ribosomal protein L21-1 |
| 1043 | Contig5603 | linamarin synthase 2-like |
| 1044 | Contig5604 | fructose-1,6-bisphosphatase, chloroplastic |
| 1045 | Contig5606 | 40S ribosomal protein S4-1 |
| 1046 | Contig5632 | probable methyltransferase PMT24 |
| 1047 | Contig5633 | myb-related protein 306-like |
| 1048 | Contig5637 | plasma membrane intrinsic protein |
| 1049 | Contig5638 | chlorophyll a-b binding protein 6, chloroplastic |
| 1050 | Contig5639 | peroxiredoxin family protein |
| 1051 | Contig5641 | 3-oxoacyl-[acyl-carrier-protein] reductase, chloroplastic-like |
| 1052 | Contig5642 | hydrophobic protein RCI2B |
| 1053 | Contig5648 | signal recognition particle 14 kDa protein |
| 1054 | Contig5649 | REF/SRPP-like protein At1g67360 |
| 1055 | Contig5653 | rhodanese-like domain-containing protein 9, chloroplastic |
| 1056 | Contig5654 | cytochrome b-c1 complex subunit Rieske-4, mitochondrial-like |
| 1057 | Contig5657 | chlorophyllase-1, chloroplastic-like |
| 1058 | Contig5660 | 2-Cys peroxiredoxin BAS1, chloroplastic |
| 1059 | Contig5663 | vacuolar protein sorting-associated protein 32 homolog 2 |
| 1060 | Contig5666 | autophagy-related protein 8C |
| 1061 | Contig5668 | ---NA--- |
| 1062 | Contig5669 | zinc finger protein CONSTANS-LIKE 5 |
| 1063 | Contig5674 | ---NA--- |
| 1064 | Contig5678 | transmembrane 9 superfamily member 1 |
| 1065 | Contig5687 | adenine phosphoribosyltransferase 1-like |
| 1066 | Contig5688 | histone H2B |
| 1067 | Contig5698 | Transmembrane 9 superfamily member 9 |
| 1068 | Contig5700 | polyubiquitin 11 |
| 1069 | Contig5704 | cysteine proteinase inhibitor |
| 1070 | Contig5706 | splicing factor U2af small subunit B-like |
| 1071 | Contig5710 | 17.3 kDa class I heat shock protein-like |
| 1072 | Contig5711 | NADH dehydrogenase [ubiquinone] iron-sulfur protein 7, mitochondrial |
| 1073 | Contig5713 | 1,2-dihydroxy-3-keto-5-methylthiopentene dioxygenase 1 |
| 1074 | Contig5723 | malate dehydrogenase |
| 1075 | Contig5728 | calnexin homolog |
| 1076 | Contig5738 | photosystem I reaction center subunit II, chloroplastic-like |
| 1077 | Contig5747 | purple acid phosphatase 17-like isoform X1 |
| 1078 | Contig5752 | serine--glyoxylate aminotransferase |
| 1079 | Contig5764 | uncharacterized protein LOC110642785 isoform X2 |
| 1080 | Contig5768 | 8-amino-7-oxononanoate synthase |
| 1081 | Contig5783 | eukaryotic initiation factor 4A-3 |
| 1082 | Contig5788 | peroxidase 16 |
| 1083 | Contig5794 | arginine decarboxylase |
| 1084 | Contig5803 | phosphoglycolate phosphatase 1B, chloroplastic-like |
| 1085 | Contig5806 | Transmembrane protein |
| 1086 | Contig5824 | endoplasmin homolog |
| 1087 | Contig5825 | 60S ribosomal protein L5 |
| 1088 | Contig5831 | thiol protease aleurain-like |
| 1089 | Contig5833 | nodulin-related protein 1-like |
| 1090 | Contig5834 | oligouridylate-binding protein 1B |
| 1091 | Contig5857 | metallothionein-like protein type 2 |
| 1092 | Contig5860 | protein argonaute 1 |
| 1093 | Contig5870 | 60S ribosomal protein L35a-1 |
| 1094 | Contig5877 | 40S ribosomal protein S24-1 |
| 1095 | Contig5885 | ribulose bisphosphate carboxylase small chain, chloroplastic-like |
| 1096 | Contig5889 | actin-depolymerizing factor 2 |
| 1097 | Contig5895 | post-GPI attachment to proteins factor 3 |
| 1098 | Contig5897 | zinc transporter 11-like |
| 1099 | Contig5903 | delta(12)-fatty-acid desaturase FAD2 |
| 1100 | Contig5904 | 40S ribosomal protein S8 |
| 1101 | Contig5907 | HR-like lesion-inducer |
| 1102 | Contig5908 | ycf3-interacting protein 1, chloroplastic isoform X1 |
| 1103 | Contig5923 | phosphoglycerate kinase, cytosolic |
| 1104 | Contig5926 | probable ribose-5-phosphate isomerase 3, chloroplastic |
| 1105 | Contig5941 | malate dehydrogenase, chloroplastic-like |
| 1106 | Contig5963 | probable UDP-arabinopyranose mutase 5 isoform X1 |
| 1107 | Contig5977 | probable inactive leucine-rich repeat receptor-like protein kinase At3g03770 |
| 1108 | Contig5982 | microtubule-associated protein 70-2-like |
| 1109 | Contig5986 | chlorophyll a-b binding protein of LHCII type 1 |
| 1110 | Contig5987 | hypothetical protein MANES_17G096800 |
| 1111 | Contig5991 | ADP-ribosylation factor |
| 1112 | Contig5995 | mitochondrial uncoupling protein 5-like |
| 1113 | Contig5996 | uncharacterized protein LOC110604426 |
| 1114 | Contig5998 | glutamine synthetase leaf isozyme, chloroplastic |
| 1115 | Contig6001 | delta(12)-fatty-acid desaturase FAD2 |
| 1116 | Contig6002 | ALA-interacting subunit 3-like |
| 1117 | Contig6008 | universal stress protein PHOS32-like |
| 1118 | Contig6009 | subtilisin-like protease SBT1.7 |
| 1119 | Contig6012 | protein PAT1 homolog 1 isoform X2 |
| 1120 | Contig6024 | heat shock factor protein HSF24 |
| 1121 | Contig6025 | proteasome subunit alpha type-6 |
| 1122 | Contig6031 | membrane steroid-binding protein 2-like |
| 1123 | Contig6032 | catalase isozyme 2 |
| 1124 | Contig6036 | 21 kDa protein-like |
| 1125 | Contig6039 | 40S ribosomal protein S6-like |
| 1126 | Contig6047 | probable nucleoredoxin 2 |
| 1127 | Contig6055 | 60S ribosomal protein L9 |
| 1128 | Contig6061 | UDP-glucuronate 4-epimerase 6-like |
| 1129 | Contig6063 | hypothetical protein MANES_12G019900 |
| 1130 | Contig6064 | 14-3-3 protein 6-like |
| 1131 | Contig6069 | profilin-1 |
| 1132 | Contig6071 | abscisic stress-ripening protein 2-like |
| 1133 | Contig6074 | 40S ribosomal protein S27-2 |
| 1134 | Contig6080 | 14 kDa proline-rich protein DC2.15-like |
| 1135 | Contig6084 | remorin isoform X1 |
| 1136 | Contig6086 | probable GTP diphosphokinase CRSH, chloroplastic |
| 1137 | Contig6096 | dihydropyrimidinase [Tarenaya hassleriana] |
| 1138 | Contig6142 | phosphoprotein ECPP44-like |
| 1139 | Contig6146 | ADP-ribosylation factor 2 |
| 1140 | Contig6147 | CBS domain-containing protein CBSX3, mitochondrial-like |
| 1141 | Contig6149 | mitochondrial-processing peptidase subunit alpha-like |
| 1142 | Contig6152 | metalloendoproteinase 1-like |
| 1143 | Contig6155 | probable transmembrane GTPase FZO-like, chloroplastic |
| 1144 | Contig6167 | 60S ribosomal protein L7a-1-like |
| 1145 | Contig6170 | coiled-coil-helix-coiled-coil-helix domain-containing protein 10, mitochondrial-like |
| 1146 | Contig6171 | chlorophyll a-b binding protein 151, chloroplastic |
| 1147 | Contig6172 | malate dehydrogenase |
| 1148 | Contig6175 | 30S ribosomal protein 3-1, chloroplastic-like |
| 1149 | Contig6176 | pectin acetylesterase 7 |
| 1150 | Contig6182 | histone deacetylase HDT1-like |
| 1151 | Contig6186 | metallothionein-like protein type 2 |
| 1152 | Contig6187 | putative cytochrome c oxidase subunit 5b-like |
| 1153 | Contig6188 | adenine phosphoribosyltransferase 5 isoform X2 |
| 1154 | Contig6197 | Ubiquitin system component Cue protein, putative isoform 1 |
| 1155 | Contig6210 | CCG-binding protein 1 |
| 1156 | Contig6219 | ethphon-induced protein |
| 1157 | Contig6221 | 60S ribosomal protein L34-like |
| 1158 | Contig6227 | mitogen-activated protein kinase kinase kinase 18-like |
| 1159 | Contig6235 | thylakoid membrane protein slr0575-like |
| 1160 | Contig6238 | uncharacterized protein LOC110604139 |
| 1161 | Contig6239 | probable calcium-binding protein CML36 |
| 1162 | Contig6243 | 21 kDa protein-like |
| 1163 | Contig6247 | 60S acidic ribosomal protein P1-like |
| 1164 | Contig6249 | putative cytochrome c oxidase subunit 5b-like |
| 1165 | Contig6255 | photosystem I subunit O |
| 1166 | Contig6262 | 40S ribosomal protein S3a |
| 1167 | Contig6269 | probable strigolactone esterase DAD2 |
| 1168 | Contig6270 | ubiquitin fusion degradation protein 1 homolog |
| 1169 | Contig6275 | D-3-phosphoglycerate dehydrogenase 1, chloroplastic-like |
| 1170 | Contig6278 | 14-3-3-like protein |
| 1171 | Contig6300 | ATP-dependent zinc metalloprotease FTSH, chloroplastic |
| 1172 | Contig6303 | ribulose bisphosphate carboxylase/oxygenase activase, chloroplastic isoform X1 |
| 1173 | Contig6304 | probable LRR receptor-like serine/threonine-protein kinase At5g10290 |
| 1174 | Contig6306 | probable methyltransferase PMT2 |
| 1175 | Contig6307 | low-temperature-induced cysteine proteinase-like |
| 1176 | Contig6320 | cinnamoyl CoA reductase |
| 1177 | Contig6322 | vacuolar-sorting receptor 1-like |
| 1178 | Contig6329 | probable sodium-coupled neutral amino acid transporter 6 |
| 1179 | Contig6337 | Protein pelota |
| 1180 | Contig6339 | 28 kDa heat- and acid-stable phosphoprotein-like |
| 1181 | Contig6343 | F-box protein SKIP8-like |
| 1182 | Contig6357 | hypersensitive-induced response protein 2 |
| 1183 | Contig6358 | DNA/RNA-binding protein Alba-like protein |
| 1184 | Contig6365 | Nucleosome assembly |
| 1185 | Contig6372 | homeobox-leucine zipper protein HAT5-like |
| 1186 | Contig6376 | 60S ribosomal protein L18-2-like |
| 1187 | Contig6378 | SEC12-like protein 2 |
| 1188 | Contig6391 | glutamate-1-semialdehyde 2,1-aminomutase 2, chloroplastic |
| 1189 | Contig6396 | 50S ribosomal protein L13, chloroplastic |
| 1190 | Contig6410 | catalase isozyme 1 |
| 1191 | Contig6413 | 2-alkenal reductase (NADP(+)-dependent) |
| 1192 | Contig6416 | ras-related protein RABB1c |
| 1193 | Contig6418 | methyltransferase-like protein |
| 1194 | Contig6424 | protein CURVATURE THYLAKOID 1B, chloroplastic-like |
| 1195 | Contig6427 | carboxymethylenebutenolidase homolog |
| 1196 | Contig6430 | protein MARD1-like |
| 1197 | Contig6434 | phosphoribosylamine--glycine ligase |
| 1198 | Contig6435 | ATP synthase subunit gamma, mitochondrial |
| 1199 | Contig6447 | pathogen-related protein-like |
| 1200 | Contig6450 | magnesium-protoporphyrin IX monomethyl ester [oxidative] cyclase, chloroplastic |
| 1201 | Contig6459 | haloalkane dehalogenase |
| 1202 | Contig6462 | exosome complex component CSL4 |
| 1203 | Contig6473 | actin-depolymerizing factor |
| 1204 | Contig6475 | probable protein phosphatase 2C 60 |
| 1205 | Contig6478 | zinc finger A20 and AN1 domain-containing stress-associated protein 4-like |
| 1206 | Contig6480 | glycerate dehydrogenase |
| 1207 | Contig6503 | polyubiquitin |
| 1208 | Contig6508 | proteinaceous RNase P 1, chloroplastic/mitochondrial-like |
| 1209 | Contig6515 | enolase |
| 1210 | Contig6517 | GRF1-interacting factor 3 |
| 1211 | Contig6522 | glyceraldehyde-3-phosphate dehydrogenase B, chloroplastic |
| 1212 | Contig6536 | uncharacterized protein LOC110609807 |
| 1213 | Contig6538 | probable aldo-keto reductase 2 |
| 1214 | Contig6539 | GTP-binding nuclear protein Ran-3-like |
| 1215 | Contig6542 | eukaryotic translation initiation factor 3 subunit K-like |
| 1216 | Contig6549 | uncharacterized protein LOC110610527 |
| 1217 | Contig6550 | putative lipid-transfer protein DIR1 |
| 1218 | Contig6563 | CAX-interacting protein 4 |
| 1219 | Contig6566 | 26S proteasome non-ATPase regulatory subunit 14 homolog |
| 1220 | Contig6569 | metallothionein-like protein type 3 |
| 1221 | Contig6576 | GATA transcription factor 25-like |
| 1222 | Contig6579 | Golgi apparatus membrane protein-like protein ECHIDNA |
| 1223 | Contig6581 | SUMO-conjugating enzyme SCE1 |
| 1224 | Contig6583 | BTB/POZ domain-containing protein POB1 |
| 1225 | Contig6584 | photosystem I reaction center subunit psaK, chloroplastic |
| 1226 | Contig6586 | thioredoxin F-type, chloroplastic-like |
| 1227 | Contig6590 | chlorophyll a-b binding protein CP26, chloroplastic |
| 1228 | Contig6594 | Negative regulator of sporulation MDS3 |
| 1229 | Contig6601 | thioredoxin M-type, chloroplastic-like |
| 1230 | Contig6603 | serine/threonine-protein kinase PBS1 |
| 1231 | Contig6605 | S-adenosylmethionine synthase 1 |
| 1232 | Contig6625 | proteasome subunit beta type-1 |
| 1233 | Contig6629 | calmodulin-7-like isoform X2 |
| 1234 | Contig6630 | N-acetyltransferase 9-like protein isoform X1 |
| 1235 | Contig6640 | pheophorbide a oxygenase, chloroplastic |
| 1236 | Contig6647 | methylesterase 3-like |
| 1237 | Contig6650 | peroxisomal fatty acid beta-oxidation multifunctional protein AIM1 |
| 1238 | Contig6665 | 60S ribosomal protein L31 |
| 1239 | Contig6669 | probable E3 ubiquitin ligase SUD1 isoform X2 |
| 1240 | Contig6697 | fructose-bisphosphate aldolase 1, chloroplastic |
| 1241 | Contig6718 | peroxisomal (S)-2-hydroxy-acid oxidase |
| 1242 | Contig6722 | nucleobase-ascorbate transporter 6 |
| 1243 | Contig6727 | 60S ribosomal protein L32-1 |
| 1244 | Contig6739 | glyceraldehyde-3-phosphate dehydrogenase GAPCP2, chloroplastic-like isoform X2 |
| 1245 | Contig6748 | 50S ribosomal protein L12-3, chloroplastic-like |
| 1246 | Contig6759 | probable WRKY transcription factor 75 |
| 1247 | Contig6774 | homeobox-leucine zipper protein ATHB-12-like |
| 1248 | Contig6775 | bifunctional protein FolD 2 |
| 1249 | Contig6778 | RGG repeats nuclear RNA binding protein A-like isoform X2 |
| 1250 | Contig6779 | homocysteine S-methyltransferase 3 |
| 1251 | Contig6782 | ferredoxin, root R-B2 |
| 1252 | Contig6785 | remorin-like |
| 1253 | Contig6788 | uncharacterized protein LOC110625201 isoform X2 |
| 1254 | Contig6790 | ---NA--- |
| 1255 | Contig6791 | uncharacterized protein LOC110618303 |
| 1256 | Contig6795 | homeobox-leucine zipper protein HAT5-like |
| 1257 | Contig6798 | ras-related protein RABD2a |
| 1258 | Contig6802 | putative membrane peptidase YdiL |
| 1259 | Contig6806 | NADH dehydrogenase [ubiquinone] 1 beta subcomplex subunit 3-B-like |
| 1260 | Contig6808 | naringenin,2-oxoglutarate 3-dioxygenase |
| 1261 | Contig6809 | mitochondrial import inner membrane translocase subunit TIM17-2-like |
| 1262 | Contig6825 | L-aspartate oxidase, chloroplastic |
| 1263 | Contig6828 | rhomboid-like protein 14, mitochondrial |
| 1264 | Contig6831 | 15.7 kDa heat shock protein, peroxisomal |
| 1265 | Contig6853 | auxin-responsive protein IAA9-like |
| 1266 | Contig6857 | mitochondrial inner membrane protease subunit 2-like |
| 1267 | Contig6864 | heat shock factor protein HSF24 |
| 1268 | Contig6867 | 60S ribosomal protein L22-2 |
| 1269 | Contig6874 | methylesterase 3-like |
| 1270 | Contig6883 | protein translation factor SUI1 homolog 2-like |
| 1271 | Contig6884 | peptidyl-prolyl cis-trans isomerase CYP19-4 |
| 1272 | Contig6895 | F-box protein SKP2A |
| 1273 | Contig6900 | GDSL esterase/lipase APG |
| 1274 | Contig6905 | 40S ribosomal protein S23 |
| 1275 | Contig6910 | probable fructokinase-7 |
| 1276 | Contig6916 | putative membrane protein |
| 1277 | Contig6917 | delta-aminolevulinic acid dehydratase 1, chloroplastic-like |
| 1278 | Contig6919 | NAC domain-containing protein 83-like |
| 1279 | Contig6924 | Eukaryotic translation initiation factor-related, putative isoform 3 |
| 1280 | Contig6928 | DUF538 domain-containing protein |
| 1281 | Contig6930 | 20 kDa chaperonin, chloroplastic-like |
| 1282 | Contig6946 | protein NRT1/ PTR FAMILY 4.6-like |
| 1283 | Contig6968 | Ribosomal protein L39e |
| 1284 | Contig6977 | CBL-interacting serine/threonine-protein kinase 6 |
| 1285 | Contig6989 | ribulose-phosphate 3-epimerase, chloroplastic |
| 1286 | Contig6993 | pyruvate kinase 1, cytosolic |
| 1287 | Contig6995 | nascent polypeptide-associated complex subunit beta-like |
| 1288 | Contig7000 | NADH dehydrogenase [ubiquinone] 1 alpha subcomplex subunit 9, mitochondrial |
| 1289 | Contig7009 | 60s ribosomal protein l44 |
| 1290 | Contig7010 | ATP-dependent Clp protease proteolytic subunit 3, chloroplastic |
| 1291 | Contig7016 | ribulose bisphosphate carboxylase/oxygenase activase, chloroplastic isoform X1 |
| 1292 | Contig7017 | Maternal effect embryo arrest 59 |
| 1293 | Contig7021 | uncharacterized protein LOC110615805 isoform X4 |
| 1294 | Contig7024 | Protein translocase subunit SecA |
| 1295 | Contig7026 | plastid-lipid-associated protein, chloroplastic |
| 1296 | Contig7030 | gamma-soluble NSF attachment protein |
| 1297 | Contig7032 | hypothetical protein MANES_04G157600 |
| 1298 | Contig7040 | cyprosin-like isoform X1 |
| 1299 | Contig7043 | 40S ribosomal protein S18 |
| 1300 | Contig7046 | cytochrome b6-f complex iron-sulfur subunit, chloroplastic-like |
| 1301 | Contig7051 | uncharacterized protein LOC110613481 |
| 1302 | Contig7056 | clathrin light chain 1-like |
| 1303 | Contig7057 | hypothetical protein MANES_14G011400 |
| 1304 | Contig7083 | senescence-associated family protein |
| 1305 | Contig7085 | 40S ribosomal protein S28-2 |
| 1306 | Contig7086 | non-classical arabinogalactan protein 31-like |
| 1307 | Contig7088 | aquaporin TIP2-1 |
| 1308 | Contig7095 | 40S ribosomal protein SA-like |
| 1309 | Contig7096 | GDSL esterase/lipase At5g33370-like |
| 1310 | Contig7097 | abscisic acid receptor PYL8-like |
| 1311 | Contig7100 | 40S ribosomal protein S2-4-like |
| 1312 | Contig7106 | UPF0496 protein 4-like |
| 1313 | Contig7107 | heavy metal-associated isoprenylated plant protein 26-like |
| 1314 | Contig7110 | late embryogenesis abundant protein At5g17165-like |
| 1315 | Contig7117 | fructose-bisphosphate aldolase, cytoplasmic isozyme 1 |
| 1316 | Contig7124 | CBL-interacting serine/threonine-protein kinase 6-like |
| 1317 | Contig7125 | elongation factor 2 |
| 1318 | Contig7127 | peroxiredoxin-2F, mitochondrial |
| 1319 | Contig7137 | probable E3 ubiquitin-protein ligase RHY1A |
| 1320 | Contig7148 | ras-related protein RABH1b |
| 1321 | Contig7154 | 40S ribosomal protein S4-3 |
| 1322 | Contig7157 | homeobox-leucine zipper protein ATHB-6-like |
| 1323 | Contig7159 | 40S ribosomal protein S18 |
| 1324 | Contig7161 | small ubiquitin-related modifier 1 |
| 1325 | Contig7163 | ADP-ribosylation factor 1 |
| 1326 | Contig7167 | 60S ribosomal protein L34-like |
| 1327 | Contig7168 | uncharacterized protein LOC110628874 |
| 1328 | Contig7170 | 4-hydroxy-3-methylbut-2-enyl diphosphate reductase, chloroplastic |
| 1329 | Contig7185 | polcalcin Phl p 7-like |
| 1330 | Contig7186 | eukaryotic translation initiation factor 5A |
| 1331 | Contig7190 | 40S ribosomal protein S9-2-like |
| 1332 | Contig7194 | FRIGIDA-like protein 4a |
| 1333 | Contig7200 | ABC transporter F family member 5 |
| 1334 | Contig7202 | asparagine--tRNA ligase, cytoplasmic 1-like |
| 1335 | Contig7203 | 40S ribosomal protein S29 |
| 1336 | Contig7209 | protein pleiotropic regulatory locus 1 |
| 1337 | Contig7216 | la-related protein 6A |
| 1338 | Contig7220 | E3 ubiquitin-protein ligase RING1-like |
| 1339 | Contig7225 | uncharacterized protein At5g49945-like |
| 1340 | Contig7229 | 60S ribosomal protein L8 |
| 1341 | Contig7230 | photosystem II 10 kDa polypeptide, chloroplastic |
| 1342 | Contig7231 | cytochrome b561 and DOMON domain-containing protein At5g47530-like |
| 1343 | Contig7234 | protein FAM32A-like |
| 1344 | Contig7239 | universal stress protein A-like protein |
| 1345 | Contig7245 | uncharacterized protein LOC110629495 |
| 1346 | Contig7246 | protein NRT1/ PTR FAMILY 5.10-like |
| 1347 | Contig7251 | phosphoenolpyruvate carboxykinase [ATP] |
| 1348 | Contig7254 | hypothetical protein MANES_S056300 |
| 1349 | Contig7258 | serine hydroxymethyltransferase 4 |
| 1350 | Contig7259 | ---NA--- |
| 1351 | Contig7261 | NADH dehydrogenase [ubiquinone] 1 alpha subcomplex subunit 13-B |
| 1352 | Contig7265 | ubiquitin-like protein 5 |
| 1353 | Contig7272 |  |
| 1354 | Contig7281 | phosphatidylcholine transfer protein, putative |
| 1355 | Contig7289 | chlorophyll a-b binding protein CP26, chloroplastic |
| 1356 | Contig7297 | 40S ribosomal protein S15a-1 |
| 1357 | Contig7306 | protein DETOXIFICATION 40-like |
| 1358 | Contig7309 | dolichyl-diphosphooligosaccharide--protein glycosyltransferase subunit 1B-like |
| 1359 | Contig7314 | multiple organellar RNA editing factor 8, chloroplastic/mitochondrial-like |
| 1360 | Contig7315 | alpha-tubulin |
| 1361 | Contig7352 | thiamine-repressible mitochondrial transport protein THI74 |
| 1362 | Contig7354 | aminomethyltransferase, mitochondrial |
| 1363 | Contig7356 | sucrose synthase 2 |
| 1364 | Contig7357 | classical arabinogalactan protein 7-like |
| 1365 | Contig7359 | protein EXORDIUM |
| 1366 | Contig7368 | wound-induced basic protein |
| 1367 | Contig7382 | uncharacterized protein LOC110599682 |
| 1368 | Contig7387 | probable sodium-coupled neutral amino acid transporter 6 |
| 1369 | Contig7393 | NADP-dependent D-sorbitol-6-phosphate dehydrogenase-like |
| 1370 | Contig7395 | ATP-dependent Clp protease adapter protein CLPS1, chloroplastic |
| 1371 | Contig7397 | photosystem II reaction center W protein, chloroplastic-like |
| 1372 | Contig7401 | 40S ribosomal protein S14-3 |
| 1373 | Contig7403 | bet1-like protein At4g14600 |
| 1374 | Contig7404 | oxygen-evolving enhancer protein 2, chloroplastic |
| 1375 | Contig7411 | Transmembrane protein |
| 1376 | Contig7412 | succinate dehydrogenase subunit 6, mitochondrial |
| 1377 | Contig7418 | sm-like protein LSM2 |
| 1378 | Contig7425 | 60S ribosomal protein L30 |
| 1379 | Contig7429 | 60S ribosomal protein L10a-2 |
| 1380 | Contig7448 | 40S ribosomal protein S3-3 |
| 1381 | Contig7452 | peptidyl-prolyl cis-trans isomerase NIMA-interacting 4 |
| 1382 | Contig7463 | ubiquitin-related modifier 1 homolog 2 |
| 1383 | Contig7465 | binding partner of ACD11 1-like |
| 1384 | Contig7467 | SPX domain-containing protein 3-like |
| 1385 | Contig7471 | peroxisomal membrane protein 13 |
| 1386 | Contig7497 | plastidic ATP/ADP-transporter |
| 1387 | Contig7505 | hypothetical protein MANES_04G082300 |
| 1388 | Contig7508 | protochlorophyllide reductase, chloroplastic |
| 1389 | Contig7518 | auxin-induced in root cultures protein 12-like |
| 1390 | Contig7519 | aquaporin TIP1-3-like |
| 1391 | Contig7551 | protein TIC110, chloroplastic |
| 1392 | Contig7553 | Transport protein SEC31 |
| 1393 | Contig7555 | homeobox-leucine zipper protein HAT5-like |
| 1394 | Contig7557 | squalene synthase |
| 1395 | Contig7578 | pentatricopeptide repeat-containing protein At2g15690-like |
| 1396 | Contig7584 | 21 kDa protein-like |
| 1397 | Contig7588 | 17.3 kDa class I heat shock protein-like |
| 1398 | Contig7589 | uncharacterized protein LOC110626509 isoform X1 |
| 1399 | Contig7591 | probable small nuclear ribonucleoprotein F |
| 1400 | Contig7592 | catalase isozyme 2 |
| 1401 | Contig7599 | phospholipase D alpha 1 |
| 1402 | Contig7604 | uncharacterized protein At4g13200, chloroplastic-like |
| 1403 | Contig7608 | protein PLANT CADMIUM RESISTANCE 8-like |
| 1404 | Contig7610 | heavy metal-associated isoprenylated plant protein 39-like |
| 1405 | Contig7612 | 40S ribosomal protein S10-1-like |
| 1406 | Contig7619 | 1-aminocyclopropane-1-carboxylate oxidase |
| 1407 | Contig7620 | hypothetical protein MANES_04G017600 |
| 1408 | Contig7625 | uncharacterized protein LOC110608525 |
| 1409 | Contig7627 | RING-H2 finger protein ATL48-like |
| 1410 | Contig7637 | multiprotein-bridging factor 1b |
| 1411 | Contig7640 | heparan-alpha-glucosaminide N-acetyltransferase-like isoform X1 |
| 1412 | Contig7652 | ---NA--- |
| 1413 | Contig7658 | elongation factor 1-gamma |
| 1414 | Contig7663 | protein disulfide isomerase-like 2-3 |
| 1415 | Contig7669 | 4F5 domain-containing protein/zf-met2 domain-containing protein |
| 1416 | Contig7677 | glucose-6-phosphate 1-dehydrogenase, cytoplasmic isoform |
| 1417 | Contig7688 | protein CutA, chloroplastic |
| 1418 | Contig7691 | gamma carbonic anhydrase 1, mitochondrial |
| 1419 | Contig7693 | zinc finger A20 and AN1 domain-containing stress-associated protein 3 |
| 1420 | Contig7703 | glutamine synthetase nodule isozyme-like |
| 1421 | Contig7721 | phosphoribosylamine--glycine ligase |
| 1422 | Contig7725 | alpha-L-arabinofuranosidase 1-like |
| 1423 | Contig7736 | Tetratricopeptide-like helical domain containing protein |
| 1424 | Contig7749 | inorganic phosphate transporter 1-4 |
| 1425 | Contig7762 | 60S ribosomal protein L27 |
| 1426 | Contig7764 | gamma-interferon-inducible lysosomal thiol reductase |
| 1427 | Contig7771 | secoisolariciresinol dehydrogenase-like |
| 1428 | Contig7778 | caffeic acid 3-O-methyltransferase |
| 1429 | Contig7779 | putative vesicle-associated membrane protein 726 |
| 1430 | Contig7788 | ferredoxin, root R-B2-like |
| 1431 | Contig7795 | uncharacterized protein LOC110630128 |
| 1432 | Contig7797 | synaptotagmin-4-like isoform X2 |
| 1433 | Contig7806 | cytochrome b561 and DOMON domain-containing protein At5g47530-like |
| 1434 | Contig7808 | KH domain-containing protein At1g09660/At1g09670-like isoform X1 |
| 1435 | Contig7809 | tubulin alpha-3 chain |
| 1436 | Contig7810 | alcohol dehydrogenase 1B |
| 1437 | Contig7811 | translation initiation factor IF-3 |
| 1438 | Contig7815 | xyloglucan endotransglucosylase/hydrolase protein 9 |
| 1439 | Contig7823 | aspartic proteinase A1-like |
| 1440 | Contig7825 | TOM1-like protein 2 |
| 1441 | Contig7837 | GTP-binding protein SAR1A-like |
| 1442 | Contig7843 | Pollen Ole e 1 allergen and extensin family protein |
| 1443 | Contig7849 | protein DETOXIFICATION 33 |
| 1444 | Contig7858 | universal stress protein PHOS34-like |
| 1445 | Contig7863 | aquaporin PIP1-3 |
| 1446 | Contig7864 | ankyrin repeat domain-containing protein 2-like |
| 1447 | Contig7874 | hypothetical protein MANES_09G109200 |
| 1448 | Contig7879 | protein BPS1, chloroplastic-like |
| 1449 | Contig7882 | 60S ribosomal protein L27-3-like |
| 1450 | Contig7886 | biotin synthase |
| 1451 | Contig7888 | ATP-dependent zinc metalloprotease FTSH, chloroplastic |
| 1452 | Contig7891 | SKP1-like protein 1B |
| 1453 | Contig7892 | 40S ribosomal protein S7 |
| 1454 | Contig7896 | 60S ribosomal protein L15 |
| 1455 | Contig7901 | nucleobase-ascorbate transporter 6 |
| 1456 | Contig7904 | eukaryotic translation initiation factor 2 subunit beta |
| 1457 | Contig7918 | glyceraldehyde-3-phosphate dehydrogenase A, chloroplastic |
| 1458 | Contig7920 | vesicle-associated membrane protein 722 |
| 1459 | Contig7924 | D-amino-acid transaminase, chloroplastic-like |
| 1460 | Contig7929 | Serine-rich protein-related |
| 1461 | Contig7932 | protein translation factor SUI1 homolog 2-like |
| 1462 | Contig7933 | protein MET1, chloroplastic-like |
| 1463 | Contig7937 | probable aquaporin PIP-type 7a |
| 1464 | Contig7938 | ---NA--- |
| 1465 | Contig7939 | ubiquitin-conjugating enzyme E2 14 |
| 1466 | Contig7941 | tropinone reductase-like 3 |
| 1467 | Contig7942 | HR-like lesion-inducer |
| 1468 | Contig7944 | proteasome subunit beta type-7-A |
| 1469 | Contig7947 | 60S ribosomal protein L19-2 |
| 1470 | Contig7951 | isocitrate dehydrogenase [NAD] catalytic subunit 5, mitochondrial |
| 1471 | Contig7952 | putative calcium-binding protein CML19 |
| 1472 | Contig7960 | uncharacterized protein LOC110600818 |
| 1473 | Contig7961 | 60S ribosomal protein L35 |
| 1474 | Contig7965 | uncharacterized protein LOC110601245 |
| 1475 | Contig7974 | phenazine biosynthesis protein, putative |
| 1476 | Contig7979 | 60S ribosomal protein L24 |
| 1477 | Contig7980 | nicotinamidase 1 |
| 1478 | Contig7984 | CBL-interacting protein kinase 32 |
| 1479 | Contig7992 | 40S ribosomal protein S26-3 |
| 1480 | Contig7994 | thioredoxin O2, mitochondrial-like isoform X1 |
| 1481 | Contig7997 | protein CURVATURE THYLAKOID 1D, chloroplastic |
| 1482 | Contig7998 | pyrrolidone-carboxylate peptidase |
| 1483 | Contig8000 | subtilisin-like protease SBT3.17 |
| 1484 | Contig8003 | 20 kDa chaperonin, chloroplastic-like |
| 1485 | Contig8007 | small ubiquitin-related modifier 1 |
| 1486 | Contig8009 | probable calcium-binding protein CML23 |
| 1487 | Contig8010 | chlorophyll a-b binding protein CP26, chloroplastic |
| 1488 | Contig8067 | HSP20-like chaperone |
| 1489 | Contig8068 | stress induced protein |
| 1490 | Contig8069 | translocase of chloroplast 33, chloroplastic |
| 1491 | Contig8076 | malate dehydrogenase, glyoxysomal |
| 1492 | Contig8078 | ATP synthase subunit delta', mitochondrial-like |
| 1493 | Contig8089 | 50S ribosomal protein L9, chloroplastic |
| 1494 | Contig8095 | alpha-galactosidase 3 |
| 1495 | Contig8099 | hypothetical protein MANES_02G056900 |
| 1496 | Contig8100 | ---NA--- |
| 1497 | Contig8108 | protein TIFY 6b-like isoform X1 |
| 1498 | Contig8109 | actin-depolymerizing factor |
| 1499 | Contig8110 | persulfide dioxygenase ETHE1 homolog, mitochondrial |
| 1500 | Contig8112 | LIM domain-containing protein WLIM2b |
| 1501 | Contig8115 | NADH dehydrogenase subunit 4 (mitochondrion) |
| 1502 | Contig8117 | 60S ribosomal protein L23 |
| 1503 | Contig8124 | oleosin 1-like |
| 1504 | Contig8127 | O-fucosyltransferase family protein isoform 1 |
| 1505 | Contig8128 | 40S ribosomal protein S11 |
| 1506 | Contig8130 | Serine-rich protein-related |
| 1507 | Contig8131 | zinc finger protein ZAT10-like |
| 1508 | Contig8136 | peroxisomal membrane protein 13-like |
| 1509 | Contig8140 | zinc finger A20 and AN1 domain-containing stress-associated protein 8-like |
| 1510 | Contig8153 | elongation factor 1-alpha-like |
| 1511 | Contig8160 | Peptidyl-prolyl cis-trans isomerase G, putative |
| 1512 | Contig8161 | bax inhibitor 1-like |
| 1513 | Contig8162 | anthranilate phosphoribosyltransferase, chloroplastic |
| 1514 | Contig8165 | protein SRG1-like isoform X2 |
| 1515 | Contig8171 | elongation factor 1-gamma |
| 1516 | Contig8174 | F-box/kelch-repeat protein At1g80440 |
| 1517 | Contig8175 | ethylene-responsive transcription factor RAP2-4-like |
| 1518 | Contig8179 | GDSL esterase/lipase At2g04570-like |
| 1519 | Contig8184 | GTP-binding protein SAR1A-like |
| 1520 | Contig8189 | Uncharacterized protein TCM_007931 |
| 1521 | Contig8191 | uncharacterized membrane protein At4g09580 |
| 1522 | Contig8205 | dnaJ homolog subfamily C member 28-like |
| 1523 | Contig8211 | NF-kappa-B-activating protein-like |
| 1524 | Contig8214 | ---NA--- |
| 1525 | Contig8222 | probable NAD(P)H dehydrogenase (quinone) FQR1-like 1 |
| 1526 | Contig8231 | aldehyde dehydrogenase family 2 member B4, mitochondrial-like |
| 1527 | Contig8249 | dehydrin |
| 1528 | Contig8250 | 40S ribosomal protein S16 |
| 1529 | Contig8251 | oxygen-evolving enhancer protein 2, chloroplastic |
| 1530 | Contig8254 | ABC transporter I family member 10, chloroplastic |
| 1531 | Contig8259 | ---NA--- |
| 1532 | Contig8260 | fructose-1,6-bisphosphatase, chloroplastic |
| 1533 | Contig8267 | 40S ribosomal protein S3a-1 |
| 1534 | Contig8274 | 17.3 kDa class I heat shock protein |
| 1535 | Contig8279 | probable mannitol dehydrogenase |
| 1536 | Contig8290 | 60S ribosomal protein L35a-1 |
| 1537 | Contig8291 | proteasome subunit alpha type-4 |
| 1538 | Contig8299 | photosystem I reaction center subunit VI, chloroplastic |
| 1539 | Contig8300 | photosystem I reaction center subunit II, chloroplastic-like |
| 1540 | Contig8301 | ferredoxin, root R-B2 |
| 1541 | Contig8302 | hypothetical protein MANES_03G099300 |
| 1542 | Contig8314 | mediator of RNA polymerase II transcription subunit 19a-like isoform X1 |
| 1543 | Contig8317 | 26S protease regulatory subunit S10B homolog B |
| 1544 | Contig8318 | peptidyl-prolyl cis-trans isomerase |
| 1545 | Contig8321 | probable inactive purple acid phosphatase 29 |
| 1546 | Contig8332 | UPF0051 protein ABCI8, chloroplastic |
| 1547 | Contig8341 | pre-mRNA cleavage factor Im 25 kDa subunit 2 |
| 1548 | Contig8348 | peptidyl-prolyl cis-trans isomerase |
| 1549 | Contig8357 | Tetratricopeptide-like helical domain containing protein |
| 1550 | Contig8358 | glucose-6-phosphate/phosphate translocator 1, chloroplastic-like |
| 1551 | Contig8361 | Transcription factor bHLH14 |
| 1552 | Contig8370 | plastidic ATP/ADP-transporter-like |
| 1553 | Contig8375 | LIM domain-containing protein WLIM1-like |
| 1554 | Contig8385 | ribonuclease 2 |
| 1555 | Contig8396 | calmodulin-binding protein 60 B-like isoform X1 |
| 1556 | Contig8411 | ---NA--- |
| 1557 | Contig8413 | probable CCR4-associated factor 1 homolog 6 |
| 1558 | Contig8416 | 60S ribosomal protein L35 |
| 1559 | Contig8418 | probable glutathione S-transferase |
| 1560 | Contig8422 | amino acid transporter AVT3B-like |
| 1561 | Contig8428 | 5-methyltetrahydropteroyltriglutamate--homocysteine methyltransferase |
| 1562 | Contig8429 | probable serine/threonine protein phosphatase 2A regulatory subunit B''delta isoform X2 |
| 1563 | Contig8443 | putative lipid-binding protein At4g00165 |
| 1564 | Contig8447 | calmodulin-7-like isoform X2 |
| 1565 | Contig8450 | serine/threonine protein phosphatase 2A 55 kDa regulatory subunit B beta isoform isoform X1 |
| 1566 | Contig8454 | chlorophyll a-b binding protein 8, chloroplastic |
| 1567 | Contig8457 | FRIGIDA-like protein 4a |
| 1568 | Contig8458 | superoxide dismutase [Mn], mitochondrial |
| 1569 | Contig8461 | auxin-responsive protein IAA9 |
| 1570 | Contig8462 | chlorophyll a-b binding protein CP29.1, chloroplastic |
| 1571 | Contig8468 | 40S ribosomal protein S10-1-like |
| 1572 | Contig8475 | probably inactive leucine-rich repeat receptor-like protein kinase At5g48380 |
| 1573 | Contig8480 | ORM1-like protein 2 |
| 1574 | Contig8488 | transketolase, chloroplastic |
| 1575 | Contig8494 | protein DEHYDRATION-INDUCED 19 homolog 4-like |
| 1576 | Contig8495 | calcineurin subunit B-like |
| 1577 | Contig8502 | polyubiquitin 11-like |
| 1578 | Contig8508 | gamma-secretase subunit APH1-like |
| 1579 | Contig8512 | UTP--glucose-1-phosphate uridylyltransferase |
| 1580 | Contig8523 | 40S ribosomal protein S3-3-like |
| 1581 | Contig8527 | chlorophyll a-b binding protein CP24 10A, chloroplastic |
| 1582 | Contig8535 | pyrophosphate-energized vacuolar membrane proton pump-like |
| 1583 | Contig8538 | ATP synthase 6 kDa subunit, mitochondrial |
| 1584 | Contig8543 | guanine nucleotide-binding protein subunit beta-like protein |
| 1585 | Contig8546 | D-amino-acid transaminase, chloroplastic |
| 1586 | Contig8550 | phosphoserine aminotransferase 2, chloroplastic-like |
| 1587 | Contig8553 | NADP-dependent malic enzyme |
| 1588 | Contig8554 | transmembrane protein 205 |
| 1589 | Contig8557 | 50S ribosomal protein L3, chloroplastic |
| 1590 | Contig8562 | 40S ribosomal protein S28-2 |
| 1591 | Contig8569 | biotin synthase |
| 1592 | Contig8577 | histone deacetylase 6 |
| 1593 | Contig8579 | ubiquitin-conjugating enzyme E2-17 kDa |
| 1594 | Contig8582 | Sanguinarine reductase |
| 1595 | Contig8585 | protein ABIL2-like isoform X1 |
| 1596 | Contig8586 | zinc finger CCCH domain-containing protein 66-like |
| 1597 | Contig8595 | transmembrane protein adipocyte-associated 1-like |
| 1598 | Contig8601 | 60S ribosomal protein L18-2-like |
| 1599 | Contig8606 | ran-binding protein 1 homolog a-like |
| 1600 | Contig8614 | abscisic acid receptor PYL9 |
| 1601 | Contig8615 | Suppressor of G2 allele of skp1 |
| 1602 | Contig8630 | photosystem II 10 kDa polypeptide, chloroplastic |
| 1603 | Contig8631 | la-related protein 6A |
| 1604 | Contig8635 | ras GTPase-activating protein-binding protein 1-like isoform X2 |
| 1605 | Contig8636 | two-component response regulator ORR3-like |
| 1606 | Contig8639 | NAD(P)H-quinone oxidoreductase subunit S, chloroplastic |
| 1607 | Contig8642 | Lactoylglutathione lyase / glyoxalase I family protein |
| 1608 | Contig8643 | ---NA--- |
| 1609 | Contig8647 | phenazine biosynthesis protein, putative |
| 1610 | Contig8659 | glutamate--glyoxylate aminotransferase 2 |
| 1611 | Contig8663 | chlorophyllase-1, chloroplastic-like |
| 1612 | Contig8665 | two-component response regulator ORR3-like |
| 1613 | Contig8667 | uncharacterized protein At3g27210 |
| 1614 | Contig8672 | alcohol dehydrogenase |
| 1615 | Contig8676 | serine/threonine-protein kinase STN7, chloroplastic |
| 1616 | Contig8685 | uncharacterized protein LOC110618026 isoform X1 |
| 1617 | Contig8686 | NEDD8-conjugating enzyme Ubc12 |
| 1618 | Contig8687 | Stress up-regulated Nod 19 |
| 1619 | Contig8708 | actin-depolymerizing factor 2 |
| 1620 | Contig8713 | 50S ribosomal protein L15, chloroplastic |
| 1621 | Contig8718 | ---NA--- |
| 1622 | Contig8729 | 60S ribosomal protein L3-2 |
| 1623 | Contig8736 | eukaryotic translation initiation factor 4B2-like |
| 1624 | Contig8737 | polyubiquitin-like |
| 1625 | Contig8738 | scarecrow-like protein 13 |
| 1626 | Contig8739 | chaperone protein dnaJ 20, chloroplastic-like |
| 1627 | Contig8754 | protein trichome birefringence-like 25 |
| 1628 | Contig8763 | calcium-transporting ATPase 1, endoplasmic reticulum-type |
| 1629 | Contig8764 | NAC domain-containing protein 41-like |
| 1630 | Contig8769 | UDP-glycosyltransferase 73B5-like |
| 1631 | Contig8773 | Copper centre Cu(A) |
| 1632 | Contig8775 | 18.1 kDa class I heat shock protein |
| 1633 | Contig8782 | Copper centre Cu(A) |
| 1634 | Contig8788 | GTP-binding nuclear protein Ran-3 |
| 1635 | Contig8789 | wound-induced basic protein |
| 1636 | Contig8790 | ADP,ATP carrier protein 3, mitochondrial |
| 1637 | Contig8792 | uncharacterized protein LOC110600510 |
| 1638 | Contig8796 | plant UBX domain-containing protein 1-like isoform X2 |
| 1639 | Contig8802 | hypothetical protein CRG98_046318 |
| 1640 | Contig8806 | probable E3 ubiquitin-protein ligase RHB1A |
| 1641 | Contig8814 | Polyubiquitin 3 |
| 1642 | Contig8818 | putative mitochondrial protein |
| 1643 | Contig8820 | NADH dehydrogenase subunit 1 (mitochondrion) |
| 1644 | Contig8824 | defensin Ec-AMP-D2-like |
| 1645 | Contig8826 | probable 1-acylglycerol-3-phosphate O-acyltransferase |
| 1646 | Contig8831 | UvrABC system protein C |
| 1647 | Contig8833 | actin-related protein 2/3 complex subunit 2A isoform X2 |
| 1648 | Contig8837 | uncharacterized protein LOC110660778 |
| 1649 | Contig8842 | Copper centre Cu(A) |
| 1650 | Contig8845 | hypothetical protein MANES_14G095600 |
| 1651 | Contig8848 | proline-rich receptor-like protein kinase PERK15 isoform X1 |
| 1652 | Contig8851 | non-histone chromosomal protein 6 |
| 1653 | Contig8857 | probable serine/threonine-protein kinase PBL7 |
| 1654 | Contig8862 | uncharacterized protein At5g39570-like |
| 1655 | Contig8871 | cytochrome b-c1 complex subunit Rieske-4, mitochondrial-like |
| 1656 | Contig8872 | AP-1 complex subunit mu-2 |
| 1657 | Contig8874 | glutathione S-transferase DHAR2-like |
| 1658 | Contig8875 | beta-amylase 1, chloroplastic |
| 1659 | Contig8878 | aspartyl protease AED3-like |
| 1660 | Contig8887 | Heat shock protein 70 family |
| 1661 | Contig8888 | DNA (cytosine-5)-methyltransferase 3A-like isoform X2 |
| 1662 | Contig8889 | plant/T7A14-6 protein |
| 1663 | Contig8903 | probable calcium-binding protein CML13 |
| 1664 | Contig8907 | putative mitochondrial protein |
| 1665 | Contig8915 | Transmembrane protein |
| 1666 | Contig8923 | ---NA--- |
| 1667 | Contig8924 | putative lignin regulation |
| 1668 | Contig8925 | ---NA--- |
| 1669 | Contig8926 | ---NA--- |
| 1670 | Contig8930 | ESCRT-related protein CHMP1B |
| 1671 | Contig8938 | CBL-interacting serine/threonine-protein kinase 25-like |
| 1672 | Contig8945 | stem-specific protein TSJT1-like |
| 1673 | Contig8948 | protein HGH1 homolog |
| 1674 | Contig8953 | S-formylglutathione hydrolase |
| 1675 | Contig8960 | heat shock cognate protein 80 |
| 1676 | Contig8970 | acetyltransferase At1g77540 |
| 1677 | Contig8977 | histone H3.3 |
| 1678 | Contig8978 | outer envelope pore protein 16, chloroplastic |
| 1679 | Contig8983 | proteasome subunit beta type-6 |
| 1680 | Contig8984 | uncharacterized protein LOC110610908 |
| 1681 | Contig8987 | ---NA--- |
| 1682 | Contig8990 | cysteine-rich receptor-like protein kinase 10 |
| 1683 | Contig8995 | ---NA--- |
| 1684 | Contig8999 | ---NA--- |
| 1685 | Contig9003 | transcription factor IBH1-like |
| 1686 | Contig9004 | neuronal acetylcholine receptor subunit alpha |
| 1687 | Contig9007 | hypothetical protein MANES_14G050000 |
| 1688 | Contig9008 | F-box protein At1g47056-like |
| 1689 | Contig9022 | sacsin isoform X1 |
| 1690 | Contig9028 | hypothetical protein MANES_03G042700, partial |
| 1691 | Contig9034 | ras-related protein RABA1d |
| 1692 | Contig9035 | copper methylamine oxidase-like isoform X1 |
| 1693 | Contig9040 | ---NA--- |
| 1694 | Contig9045 | ubiquitin-conjugating enzyme E2-17 kDa |
| 1695 | Contig9046 | hypothetical protein TSUD_291690 |
| 1696 | Contig9049 | RING-H2 finger protein ATL8 |
| 1697 | Contig9050 | altered inheritance of mitochondria protein 32-like |
| 1698 | Contig9055 | AAA-ATPase At2g46620-like |
| 1699 | Contig9060 | cyclin-dependent kinases regulatory subunit 1 |
| 1700 | Contig9063 | Copper centre Cu(A) |
| 1701 | Contig9066 | ubiquitin-conjugating enzyme E2 22-like |
| 1702 | Contig9068 | protein LATERAL ROOT PRIMORDIUM 1-like |
| 1703 | Contig9069 | latex abundant protein 1 |
| 1704 | Contig9070 | cyclin-B1-2-like |
| 1705 | Contig9073 | Copper centre Cu(A) |
| 1706 | Contig9080 | Demethylmenaquinone methyltransferase |
| 1707 | Contig9084 | phosphoenolpyruvate carboxylase kinase 2-like |
| 1708 | Contig9091 | N-terminal acetyltransferase A complex catalytic subunit NAA10-like |
| 1709 | Contig9092 | hepatocyte growth factor-regulated tyrosine kinase substrate-like |
| 1710 | Contig9095 | hypothetical protein MANES_06G100800 |
| 1711 | Contig9103 | peptide methionine sulfoxide reductase-like |
| 1712 | Contig9107 | ribosomal protein S3 (mitochondrion) |
| 1713 | Contig9108 | ---NA--- |
| 1714 | Contig9111 | histone H1 |
| 1715 | Contig9127 | polyubiquitin |
| 1716 | Contig9136 | 60S ribosomal protein L29-1 |
| 1717 | Contig9168 | pyrophosphate-energized vacuolar membrane proton pump |
| 1718 | Contig9173 | death domain associated protein |
| 1719 | Contig9175 | probable inactive patatin-like protein 9 |
| 1720 | Contig9184 | ethylene-responsive transcription factor RAP2-12-like |
| 1721 | Contig9185 | Senescence associated gene 20, putative |
| 1722 | Contig9186 | serine--tRNA ligase |
| 1723 | Contig9187 | 60S ribosomal protein L37a |
| 1724 | Contig9188 | 40S ribosomal protein S2-4-like |
| 1725 | Contig9202 | heat shock 70 kDa protein 15-like |
| 1726 | Contig9203 | ubiquitin-conjugating enzyme E2 14 |
| 1727 | Contig9208 | 14 kDa proline-rich protein DC2.15-like |
| 1728 | Contig9216 | probable mannitol dehydrogenase |
| 1729 | Contig9227 | ---NA--- |
| 1730 | Contig9230 | putative zinc finger protein CONSTANS-LIKE 11 isoform X1 |
| 1731 | Contig9249 | 40S ribosomal protein S16 |
| 1732 | Contig9250 | 22.0 kDa class IV heat shock protein |
| 1733 | Contig9255 | binding partner of ACD11 1 |
| 1734 | Contig9260 | GDP-L-galactose phosphorylase 1-like |
| 1735 | Contig9266 | glutathione peroxidase |
| 1736 | Contig9267 | protein gast1 |
| 1737 | Contig9270 | 60S ribosomal protein L7a-1 |
| 1738 | Contig9275 | fructose-bisphosphate aldolase 6, cytosolic |
| 1739 | Contig9278 | protein EARLY RESPONSIVE TO DEHYDRATION 15-like |
| 1740 | Contig9285 | ribosome biogenesis protein NOP53 isoform X1 |
| 1741 | Contig9289 | tubulin beta-5 chain |
| 1742 | Contig9305 | serine/threonine-protein kinase SRK2A |
| 1743 | Contig9312 | methylthioribose kinase-like |
| 1744 | Contig9319 | putative 4-hydroxy-4-methyl-2-oxoglutarate aldolase 2 |
| 1745 | Contig9321 | polyprotein |
| 1746 | Contig9323 | C-repeat/dehydration-responsive element-binding factor 4 |
| 1747 | Contig9331 | superoxide dismutase [Cu-Zn]-like |
| 1748 | Contig9333 | 30S ribosomal protein S31, mitochondrial-like |
| 1749 | Contig9336 | 60S ribosomal protein L23 |
| 1750 | Contig9339 | arginine decarboxylase |
| 1751 | Contig9360 | tetraspanin-8-like |
| 1752 | Contig9380 | 60S ribosomal protein L18-2-like |
| 1753 | Contig9384 | NADH dehydrogenase [ubiquinone] 1 beta subcomplex subunit 7-like |
| 1754 | Contig9386 | vacuolar-processing enzyme |
| 1755 | Contig9389 | 60S ribosomal protein L7a-1 |
| 1756 | Contig9393 | nucleoside diphosphate kinase B |
| 1757 | Contig9397 | glyoxylate/hydroxypyruvate reductase HPR3-like |
| 1758 | Contig9398 | polyubiquitin 11 |
| 1759 | Contig9404 | protein GIGANTEA |
| 1760 | Contig9422 | polyubiquitin 11 |
| 1761 | Contig9428 | 60S ribosomal protein L32-1 |
| 1762 | Contig9433 | probable WRKY transcription factor 53 |
| 1763 | Contig9438 | chaperone protein DnaJ |
| 1764 | Contig9441 | probable xyloglucan endotransglucosylase/hydrolase protein 23 |
| 1765 | Contig9444 | heavy metal-associated isoprenylated plant protein 46 |
| 1766 | Contig9447 | 40S ribosomal protein S29 |
| 1767 | Contig9452 | cell wall integrity and stress response component 1 |
| 1768 | Contig9456 | PHD finger protein ALFIN-LIKE 7-like isoform X1 |
| 1769 | Contig9476 | small EDRK-rich factor 2-like |
| 1770 | Contig9501 | coat protein |
| 1771 | Contig9504 | ubiquinone biosynthesis protein COQ9-B, mitochondrial |
| 1772 | Contig9511 | ubiquitin-60S ribosomal protein L40 |
| 1773 | Contig9515 | protein DEHYDRATION-INDUCED 19 homolog 3-like |
| 1774 | Contig9525 | homeobox protein knotted-1-like LET12 isoform X1 |
| 1775 | Contig9544 | ketol-acid reductoisomerase, chloroplastic |
| 1776 | Contig9553 | putative disease resistance protein At4g11170 |
| 1777 | Contig9565 | pyruvate dehydrogenase E1 component subunit alpha-3, chloroplastic |
| 1778 | Contig9570 | ---NA--- |
| 1779 | Contig9573 | GPN-loop GTPase 3 |
| 1780 | Contig9574 | nuclear transcription factor Y subunit B-1-like isoform X1 |
| 1781 | Contig9594 | RNA polymerase II second largest subunit |
| 1782 | Contig9597 | phosphotriesterase-related protein |
| 1783 | Contig9599 | cytochrome c oxidase subunit 6b-1 |
| 1784 | Contig9611 | UDP-D-apiose/UDP-D-xylose synthase 2 |
| 1785 | Contig9618 | peroxisome biogenesis protein 22 |
| 1786 | Contig9624 | protein SRC2 |
| 1787 | Contig9628 | allergenic-related protein Pt2L4 |
| 1788 | Contig9639 | 36.4 kDa proline-rich protein-like |
| 1789 | Contig9643 | shaggy-related protein kinase eta |
| 1790 | Contig9654 | 40S ribosomal protein S25-like |
| 1791 | Contig9657 | FMN-linked oxidoreductases superfamily protein |
| 1792 | Contig9660 | succinate-semialdehyde dehydrogenase, mitochondrial |
| 1793 | Contig9667 | golgin subfamily B member 1 |
| 1794 | Contig9675 | phosphatidylinositol 3-kinase, root isoform |
| 1795 | Contig9680 | Glycosyl transferase |
| 1796 | Contig9682 | methylmalonate-semialdehyde dehydrogenase [acylating], mitochondrial-like |
| 1797 | Contig9694 |  |
| 1798 | Contig9701 | ---NA--- |
| 1799 | Contig9713 | uncharacterized protein At5g39570 |
| 1800 | Contig9728 | transaldolase family protein |
| 1801 | Contig9730 | eukaryotic translation initiation factor 2 subunit beta-like |
| 1802 | Contig9738 | CBS domain-containing protein CBSX3, mitochondrial |
| 1803 | Contig9743 | presequence protease 1, chloroplastic/mitochondrial-like |
| 1804 | Contig9752 | 60S ribosomal protein L8 |
| 1805 | Contig9754 | 2-methyl-6-phytyl-1,4-hydroquinone methyltransferase, chloroplastic |
| 1806 | Contig9755 | transcription factor ILR3 |
| 1807 | Contig9759 | ATP-dependent Clp protease proteolytic subunit-related protein 3, chloroplastic |
| 1808 | Contig9760 | probable pyridoxal 5'-phosphate synthase subunit PDX1 |
| 1809 | Contig9767 | PREDICTED: uncharacterized protein LOC8285295 |
| 1810 | Contig9768 | RNA polymerase II transcriptional coactivator KELP |
| 1811 | Contig9771 | ethylene-responsive transcription factor RAP2-3 |
| 1812 | Contig9778 | LIM domain-containing protein WLIM1-like |
| 1813 | Contig9779 | ubiquitin-conjugating enzyme E2 variant 1D |
| 1814 | Contig9780 | uncharacterized protein LOC110624412 isoform X1 |
| 1815 | Contig9784 | profilin-1 |
| 1816 | Contig9811 | monothiol glutaredoxin-S10 isoform X1 |
| 1817 | Contig9813 | hypothetical protein MANES_07G004700 |
| 1818 | Contig9815 | oligouridylate-binding protein 1B |
| 1819 | Contig9816 | hypothetical protein MANES_02G177700 |
| 1820 | Contig9819 | ---NA--- |
| 1821 | Contig9831 | ubiquitin-conjugating enzyme E2 28 |
| 1822 | Contig9835 | 2-oxoglutarate dehydrogenase, mitochondrial-like |
| 1823 | Contig9847 | metalloendoproteinase 1-like |
| 1824 | Contig9857 | auxin-responsive protein IAA27 |
| 1825 | Contig9859 | TATA element modulatory factor isoform X1 |
| 1826 | Contig9868 | D-amino-acid transaminase, chloroplastic |
| 1827 | Contig9869 | ubiquitin-conjugating enzyme E2-17 kDa |
| 1828 | Contig9874 | germin-like protein subfamily 2 member 4 |
| 1829 | Contig9876 | hypothetical protein MANES_14G095700 |
| 1830 | Contig9877 | DNA polymerase epsilon catalytic subunit A, putative |
| 1831 | Contig9881 | diaminopimelate decarboxylase 2, chloroplastic-like |
| 1832 | Contig9886 | protein transport protein Sec61 subunit alpha-like |
| 1833 | Contig9892 | actin-7 |
| 1834 | Contig9899 | probable methyltransferase PMT2 |
| 1835 | Contig9902 | subtilisin-like protease SBT3.3 isoform X2 |
| 1836 | Contig9909 | ERBB-3 BINDING PROTEIN 1 |
| 1837 | Contig9928 | phenylalanine ammonia-lyase |
| 1838 | Contig9929 | uncharacterized protein LOC110627882 |
| 1839 | Contig9939 | Late embryogenesis abundant protein, LEA-14 |
| 1840 | Contig9944 | 60S ribosomal protein L34-like |
| 1841 | Contig9956 | EG2771 |
| 1842 | Contig9957 | probable aquaporin PIP2-8 |
| 1843 | Contig9959 | probable histone H2A.1 |
| 1844 | Contig9960 | bZIP transcription factor 11-like |
| 1845 | Contig9964 | zinc finger A20 and AN1 domain-containing stress-associated protein 8-like |
| 1846 | Contig9970 | ATP synthase subunit beta, mitochondrial |
| 1847 | Contig9972 | histone H3.3 |
| 1848 | Contig9973 | adenosine kinase 2 |
| 1849 | Contig9974 | 2,3-bisphosphoglycerate-independent phosphoglycerate mutase |
| 1850 | Contig9988 | sulfite reductase [ferredoxin], chloroplastic-like |
| 1851 | Contig10004 | pre-mRNA-processing factor 19 homolog 1-like |
| 1852 | Contig10010 | hypothetical protein MANES_06G041600, partial |
| 1853 | Contig10013 | UDP-glucuronic acid decarboxylase 6-like |
| 1854 | Contig10024 | ATOZI1, putative |
| 1855 | Contig10032 | microsomal glutathione S-transferase 3 |
| 1856 | Contig10035 | xylose isomerase |
| 1857 | Contig10039 | endoglucanase 9 |
| 1858 | Contig10041 | senescence-associated family protein |
| 1859 | Contig10049 | 40S ribosomal protein S17-like |
| 1860 | Contig10050 | transketolase, chloroplastic |
| 1861 | Contig10052 | dynamin-like protein ARC5 |
| 1862 | Contig10063 | tubulin beta chain-like |
| 1863 | Contig10064 | ubiquitin-conjugating enzyme E2 10 |
| 1864 | Contig10067 | putative clathrin assembly protein At2g25430 |
| 1865 | Contig10077 | 60S ribosomal protein L26-1 |
| 1866 | Contig10082 | mediator of RNA polymerase II transcription subunit 31 |
| 1867 | Contig10087 | ---NA--- |
| 1868 | Contig10090 | LRR domain containing protein |
| 1869 | Contig10100 | ethylene-responsive transcription factor RAP2-12-like |
| 1870 | Contig10105 | 60S ribosomal protein L13-1-like |
| 1871 | Contig10125 | protein usf |
| 1872 | Contig10133 | uncharacterized protein LOC110620271 |
| 1873 | Contig10135 | membrane family protein |
| 1874 | Contig10138 | F-box protein GID2-like |
| 1875 | Contig10149 | ---NA--- |
| 1876 | Contig10152 | ubiquitin-conjugating enzyme E2 variant 1D |
| 1877 | Contig10159 | basic secretory protease |
| 1878 | Contig10163 | nuclear transport factor 2 |
| 1879 | Contig10168 | UTP--glucose-1-phosphate uridylyltransferase |
| 1880 | Contig10187 | calreticulin |
| 1881 | Contig10189 | high mobility group B protein 2-like |
| 1882 | Contig10207 | calmodulin-like protein 5 |
| 1883 | Contig10208 | early nodulin-93-like |
| 1884 | Contig10215 | coat protein |
| 1885 | Contig10222 | V-type proton ATPase 16 kDa proteolipid subunit |
| 1886 | Contig10226 | eukaryotic initiation factor 4A-8 |
| 1887 | Contig10227 | protein FLX-like 1 |
| 1888 | Contig10237 | trans-cinnamate 4-monooxygenase |
| 1889 | Contig10263 | GID1b |
| 1890 | Contig10270 | heat shock 70 kDa protein 15-like |
| 1891 | Contig10273 | protein transport protein Sec61 subunit gamma-like |
| 1892 | Contig10275 | alpha tubulin 1 |
| 1893 | Contig10277 | ethylene receptor |
| 1894 | Contig10285 | Acidic leucine-rich nuclear phosphoprotein 32-related protein 2 |
| 1895 | Contig10286 | abscisic acid receptor PYL9 |
| 1896 | Contig10287 | ---NA--- |
| 1897 | Contig10288 | ---NA--- |
| 1898 | Contig10289 | predicted protein |
| 1899 | Contig10294 | histone H2A |
| 1900 | Contig10297 | mitochondrial import receptor subunit TOM5 homolog |
| 1901 | Contig10299 | sucrose transporter 1 |
| 1902 | Contig10310 | hypothetical protein MANES_04G067700 |
| 1903 | Contig10331 | ruBisCO large subunit-binding protein subunit alpha |
| 1904 | Contig10337 | ---NA--- |
| 1905 | Contig10352 | cytochrome c-type biogenesis ccda-like chloroplastic protein 2 |
| 1906 | Contig10362 | fructose-bisphosphate aldolase 6, cytosolic |
| 1907 | Contig10363 | 28 kDa ribonucleoprotein, chloroplastic-like |
| 1908 | Contig10365 | FT-interacting protein 1-like |
| 1909 | Contig10369 | protein SPIRAL1-like 1 |
| 1910 | Contig10371 | BAHD acyltransferase DCR |
| 1911 | Contig10374 | probable protein disulfide-isomerase A6 |
| 1912 | Contig10385 | serine/threonine-protein phosphatase PP2A-3 catalytic subunit |
| 1913 | Contig10387 | RGG repeats nuclear RNA binding protein A-like |
| 1914 | Contig10410 | elongation factor 1-alpha |
| 1915 | Contig10412 | elongation factor 1-alpha |
| 1916 | Contig10414 | elongation factor 1-alpha |
| 1917 | Contig10421 | fructose-bisphosphate aldolase 1, chloroplastic |
| 1918 | Contig10422 | probable aldo-keto reductase 2 |
| 1919 | Contig10424 | auxin-repressed 12.5 kDa protein-like isoform X2 |
| 1920 | Contig10425 | probable aldo-keto reductase 2 |
| 1921 | Contig10433 | glycerophosphodiester phosphodiesterase GDPD6 |
| 1922 | Contig10434 | phenylalanine ammonia-lyase |
| 1923 | Contig10438 | probable aldo-keto reductase 2 |
| 1924 | Contig10440 | auxin-repressed 12.5 kDa protein-like isoform X2 |
| 1925 | Contig10443 | probable aldo-keto reductase 2 |
| 1926 | Contig10444 | elongation factor 1-alpha |
| 1927 | Contig10445 | auxin-repressed 12.5 kDa protein-like isoform X2 |
| 1928 | Contig10446 | probable aldo-keto reductase 2 |
| 1929 | Contig10447 | probable aldo-keto reductase 2 |

***Ricinus communis***

| **Sl No.** | **ID** | **Function** |
| --- | --- | --- |
| 1 | Contig5 | 40S ribosomal protein S27-like |
| 2 | Contig6 | trans-cinnamate 4-monooxygenase |
| 3 | Contig7 | probable pectate lyase 5 |
| 4 | Contig13 | fructose-bisphosphate aldolase 6, cytosolic |
| 5 | Contig14 | phosphoglycolate phosphatase 1B, chloroplastic-like |
| 6 | Contig15 | protein TRANSPARENT TESTA 16 |
| 7 | Contig16 | hypothetical protein BOX15_Mlig031762g1 |
| 8 | Contig18 | Actin, cytoplasmic 2 |
| 9 | Contig22 | 60S ribosomal protein L7a-1 |
| 10 | Contig31 | Dynein light chain 1, cytoplasmic |
| 11 | Contig37 | 60S ribosomal protein L8 |
| 12 | Contig47 | biotin carboxyl carrier protein of acetyl-CoA carboxylase |
| 13 | Contig65 | hypothetical protein BOX15_Mlig006345g3, partial |
| 14 | Contig74 | CRE-LBP-5 protein |
| 15 | Contig81 | 60S ribosomal protein L5-like |
| 16 | Contig84 | 40S ribosomal protein S4-1 |
| 17 | Contig97 | protein FATTY ACID EXPORT 2, chloroplastic |
| 18 | Contig98 | oxygen-dependent coproporphyrinogen-III oxidase, chloroplastic |
| 19 | Contig99 | non-classical arabinogalactan protein 31-like |
| 20 | Contig103 | venom protease-like |
| 21 | Contig119 | 40S ribosomal protein S5 |
| 22 | Contig125 | probable proteasome inhibitor isoform X1 |
| 23 | Contig129 | ---NA--- |
| 24 | Contig135 | hypothetical protein BOX15_Mlig017551g1 |
| 25 | Contig149 | probable cysteine protease RD19D |
| 26 | Contig160 | ubiquitin-40S ribosomal protein S27a |
| 27 | Contig171 | superoxide dismutase [Mn], mitochondrial |
| 28 | Contig181 | hypothetical protein BOX15_Mlig021841g1, partial |
| 29 | Contig189 | 60S ribosomal protein L5 |
| 30 | Contig200 | 36.4 kDa proline-rich protein |
| 31 | Contig201 | 11-beta-hydroxysteroid dehydrogenase 1B-like |
| 32 | Contig203 | tetraspanin-3-like |
| 33 | Contig213 | proteasome subunit alpha type-1-B-like |
| 34 | Contig233 | ATP synthase subunit beta, mitochondrial |
| 35 | Contig236 | 2S albumin-like |
| 36 | Contig253 | mitochondrial succinate-fumarate transporter 1 |
| 37 | Contig254 | zinc finger A20 and AN1 domain-containing stress-associated protein 4-like |
| 38 | Contig255 | UDP-glucuronic acid decarboxylase 1 |
| 39 | Contig266 | pyruvate decarboxylase 2 |
| 40 | Contig270 | serine/arginine-rich splicing factor RSZ21 isoform X1 |
| 41 | Contig291 | eukaryotic translation initiation factor 4B3 |
| 42 | Contig293 | hypersensitive-induced response protein 2 |
| 43 | Contig300 | chymotrypsin-like protease CTRL-1 |
| 44 | Contig310 | glutathione S-transferase DHAR2-like |
| 45 | Contig321 | protein TIFY 10A |
| 46 | Contig342 | hypothetical protein BOX15_Mlig025766g2 |
| 47 | Contig348 | hypothetical protein BOX15_Mlig001779g6, partial |
| 48 | Contig349 | probable galactinol--sucrose galactosyltransferase 2 |
| 49 | Contig351 | FHA domain-containing protein FHA2-like isoform X1 |
| 50 | Contig352 | polyubiquitin 10 |
| 51 | Contig369 | 60S ribosomal protein L36-like |
| 52 | Contig373 | hypothetical protein BOX15_Mlig019489g1 |
| 53 | Contig375 | protein ENHANCED DISEASE RESISTANCE 2-like |
| 54 | Contig378 | hypothetical protein BOX15_Mlig019594g3 |
| 55 | Contig380 | NADH dehydrogenase [ubiquinone] 1 alpha subcomplex subunit 13-B |
| 56 | Contig392 | probable WRKY transcription factor 17 |
| 57 | Contig396 | ABC transporter I family member 6, chloroplastic |
| 58 | Contig412 | hypothetical protein BOX15_Mlig003912g2 |
| 59 | Contig437 | PREDICTED: uncharacterized protein LOC8271821 isoform X1 |
| 60 | Contig438 | probable pyridoxal 5'-phosphate synthase subunit PDX1 |
| 61 | Contig441 | annexin-like protein RJ4 isoform X2 |
| 62 | Contig453 | caveolin-1-like |
| 63 | Contig456 | ribosomal protein L37a |
| 64 | Contig459 | late embryogenesis abundant protein, group 3 |
| 65 | Contig460 | hypothetical protein BOX15_Mlig017017g6 |
| 66 | Contig471 | hypothetical protein BOX15_Mlig026993g1 |
| 67 | Contig476 | lysine-rich arabinogalactan protein 18 |
| 68 | Contig485 | 60S ribosomal protein L12-like |
| 69 | Contig491 | GTP-binding nuclear protein Ran-3 |
| 70 | Contig496 | ribonuclease P protein subunit p25-like protein |
| 71 | Contig502 | low choriolytic enzyme |
| 72 | Contig515 | E3 ubiquitin-protein ligase SH3RF1 |
| 73 | Contig521 | small heat shock protein, chloroplastic-like isoform X2 |
| 74 | Contig529 | AGAP000573-PB-like protein |
| 75 | Contig533 | 40S ribosomal protein S6 |
| 76 | Contig539 | triosephosphate isomerase, cytosolic |
| 77 | Contig540 | eukaryotic initiation factor 4A-11 |
| 78 | Contig542 | aspartyl protease AED3-like |
| 79 | Contig546 | multiple organellar RNA editing factor 3, mitochondrial |
| 80 | Contig549 | hypothetical protein BOX15_Mlig030183g1 |
| 81 | Contig558 | 2-hydroxyisoflavanone dehydratase-like |
| 82 | Contig563 | CRE-LBP-5 protein |
| 83 | Contig564 | 40S ribosomal protein S4-3 |
| 84 | Contig572 | vesicle-associated membrane protein 714 |
| 85 | Contig593 | putative cytochrome c oxidase subunit 5b-like |
| 86 | Contig602 | 36.4 kDa proline-rich protein-like |
| 87 | Contig610 | ADP,ATP carrier protein 1, mitochondrial |
| 88 | Contig631 | 60S ribosomal protein L18 |
| 89 | Contig681 | legumin B-like |
| 90 | Contig682 | nuclear transcription factor Y subunit A-3-like isoform X1 |
| 91 | Contig683 | inositol-3-phosphate synthase |
| 92 | Contig693 | preproricin |
| 93 | Contig698 | enolase |
| 94 | Contig702 | aminomethyltransferase, mitochondrial |
| 95 | Contig706 | nucleosome assembly protein 1;2 |
| 96 | Contig707 | probable 26S proteasome non-ATPase regulatory subunit 3 |
| 97 | Contig710 | sorting nexin 1 isoform X2 |
| 98 | Contig712 | serine/arginine-rich splicing factor SR45a-like |
| 99 | Contig713 | subtilisin-like protease SBT1.8 |
| 100 | Contig715 | vacuolar-processing enzyme-like |
| 101 | Contig718 | serine carboxypeptidase-like |
| 102 | Contig719 | probable trafficking protein particle complex subunit 13 homolog isoform X1 |
| 103 | Contig720 | DUF2359 domain-containing protein |
| 104 | Contig722 | calnexin homolog |
| 105 | Contig726 | cytochrome P450 98A2 |
| 106 | Contig730 | phosphomethylpyrimidine synthase, chloroplastic isoform X1 |
| 107 | Contig732 | enoyl-[acyl-carrier-protein] reductase [NADH], chloroplastic-like |
| 108 | Contig735 | putative amidase C869.01 |
| 109 | Contig740 | 40S ribosomal protein S3a |
| 110 | Contig741 | 60S ribosomal protein L17-2 |
| 111 | Contig743 | obg-like ATPase 1 |
| 112 | Contig745 | ER membrane protein complex subunit 10 |
| 113 | Contig752 | 3-oxoacyl-[acyl-carrier-protein] synthase I, chloroplastic |
| 114 | Contig756 | metal transporter Nramp5-like |
| 115 | Contig759 | plastidic ATP/ADP-transporter-like |
| 116 | Contig761 | ricin precursor |
| 117 | Contig767 | eukaryotic translation initiation factor 3 subunit C-like |
| 118 | Contig770 | PREDICTED: uncharacterized protein LOC8268650 |
| 119 | Contig774 | ---NA--- |
| 120 | Contig777 | thiamine thiazole synthase, chloroplastic |
| 121 | Contig780 | ascorbate transporter, chloroplastic isoform X1 |
| 122 | Contig782 | stearoyl-[acyl-carrier-protein] 9-desaturase, chloroplastic |
| 123 | Contig784 | 60S ribosomal protein L18-2 |
| 124 | Contig787 |  |
| 125 | Contig791 | 60S ribosomal protein L10a-2 |
| 126 | Contig796 | ubiquitin-conjugating enzyme E2 10 |
| 127 | Contig797 | elongation factor 1-alpha-like |
| 128 | Contig798 | triacylglycerol lipase, putative |
| 129 | Contig799 | ruBisCO large subunit-binding protein subunit alpha |
| 130 | Contig801 | tubulin alpha-3 chain |
| 131 | Contig802 | cell differentiation protein RCD1 homolog isoform X1 |
| 132 | Contig806 | ketol-acid reductoisomerase, chloroplastic |
| 133 | Contig820 | UDP-glucose 6-dehydrogenase 5 |
| 134 | Contig821 | probable serine incorporator |
| 135 | Contig825 | aspartic proteinase A1-like |
| 136 | Contig827 | 14-3-3-like protein |
| 137 | Contig832 | triacylglycerol lipase, putative |
| 138 | Contig838 | 40S ribosomal protein S6 |
| 139 | Contig841 | protein MOTHER of FT and TFL1-like |
| 140 | Contig848 | proteasome subunit beta type-4-like |
| 141 | Contig852 | stearoyl-[acyl-carrier-protein] 9-desaturase 6, chloroplastic |
| 142 | Contig855 | oligouridylate-binding protein 1 |
| 143 | Contig856 | myb-like protein X isoform X1 |
| 144 | Contig857 | alanine aminotransferase 2-like |
| 145 | Contig864 | protein disulfide-isomerase-like |
| 146 | Contig871 | iron-sulfur cluster assembly protein 1-like |
| 147 | Contig877 | outer envelope pore protein 24B, chloroplastic |
| 148 | Contig881 | osmotin-like protein |
| 149 | Contig890 | 40S ribosomal protein S9-2-like |
| 150 | Contig901 | vicilin-like seed storage protein At2g18540 |
| 151 | Contig906 | translationally-controlled tumor protein homolog |
| 152 | Contig915 | 2S albumin-like |
| 153 | Contig920 | oleosin 1-like |
| 154 | Contig922 | heavy metal-associated isoprenylated plant protein 7-like |
| 155 | Contig927 | probable aminotransferase TAT2 |
| 156 | Contig943 | delta(12)-acyl-lipid-desaturase-like |
| 157 | Contig945 | enolase |
| 158 | Contig946 | cysteine synthase |
| 159 | Contig950 | RNA-binding KH domain-containing protein PEPPER-like |
| 160 | Contig952 | 1-Cys peroxiredoxin |
| 161 | Contig955 | glycine-rich RNA-binding protein GRP1A-like |
| 162 | Contig958 | conserved hypothetical protein |
| 163 | Contig961 | 40S ribosomal protein S12-like |
| 164 | Contig962 | 60S ribosomal protein L24 |
| 165 | Contig966 | 40S ribosomal protein S18 |
| 166 | Contig968 | metallothionein-like protein type 2 |
| 167 | Contig969 | pyrophosphate--fructose 6-phosphate 1-phosphotransferase subunit beta |
| 168 | Contig978 | 2S albumin-like |
| 169 | Contig983 | probable phospholipid hydroperoxide glutathione peroxidase |
| 170 | Contig985 | 60S ribosomal protein L34-like |
| 171 | Contig989 | PREDICTED: uncharacterized protein LOC8268539 |
| 172 | Contig995 | 60S ribosomal protein L7-4 |
| 173 | Contig998 | legumin B-like |
| 174 | Contig1015 | 40S ribosomal protein S20-2 |
| 175 | Contig1021 | 2,3-bisphosphoglycerate-independent phosphoglycerate mutase |
| 176 | Contig1025 | tRNA 2'-phosphotransferase 1 |
| 177 | Contig1040 | transmembrane emp24 domain-containing protein p24beta3 |
| 178 | Contig1042 | non-specific lipid-transfer protein 2-like |
| 179 | Contig1044 | subtilisin-like protease SBT1.7 |
| 180 | Contig1045 | 60S acidic ribosomal protein P1 |
| 181 | Contig1046 | 40S ribosomal protein S8-like |
| 182 | Contig1047 | ubiquitin-60S ribosomal protein L40 |
| 183 | Contig1057 | NADP-specific glutamate dehydrogenase |
| 184 | Contig1083 | proteasome subunit alpha type-3 |
| 185 | Contig1096 | REF/SRPP-like protein At1g67360 |
| 186 | Contig1097 | sucrose-binding protein-like |
| 187 | Contig1098 | 18 kDa seed maturation protein |
| 188 | Contig1100 | cell number regulator 8-like |
| 189 | Contig1103 | legumin A |
| 190 | Contig1104 | histone H1-like |
| 191 | Contig1111 | subtilisin-like protease SBT1.5 |
| 192 | Contig1112 | zinc finger CCCH domain-containing protein 20-like |
| 193 | Contig1115 | PREDICTED: uncharacterized protein LOC8262535 |
| 194 | Contig1128 | oil body-associated protein 2c |
| 195 | Contig1131 | histone H1 |
| 196 | Contig1135 | histidine-containing phosphotransfer protein 1-like |
| 197 | Contig1137 | subtilisin-like protease SBT1.8 |
| 198 | Contig1138 | legumin B |
| 199 | Contig1147 | succinate--CoA ligase [ADP-forming] subunit alpha-2, mitochondrial |
| 200 | Contig1148 | seed biotin-containing protein SBP65 isoform X1 |
| 201 | Contig1150 | oleosin 18.2 kDa-like |
| 202 | Contig1151 | cysteine proteinase inhibitor 12 |
| 203 | Contig1154 | probable calcium-binding protein CML49 |
| 204 | Contig1157 | 26S protease regulatory subunit S10B homolog B |
| 205 | Contig1166 | protein phosphatase 2C 37-like |
| 206 | Contig1167 | legumin A |
| 207 | Contig1177 | cytochrome P450 like_TBP |
| 208 | Contig1180 | 14-3-3-like protein GF14 kappa isoform X1 |
| 209 | Contig1183 | ethylene-responsive transcription factor 3-like |
| 210 | Contig1190 | protein PYRICULARIA ORYZAE RESISTANCE 21-like |
| 211 | Contig1192 | PREDICTED: uncharacterized protein LOC8263722 |
| 212 | Contig1201 | proline synthase co-transcribed bacterial homolog protein |
| 213 | Contig1202 | HSP20-like chaperones superfamily protein isoform 1 |
| 214 | Contig1205 | 40S ribosomal protein S2-4-like |
| 215 | Contig1208 | benzyl alcohol O-benzoyltransferase-like |
| 216 | Contig1219 | sorting and assembly machinery component 50 homolog |
| 217 | Contig1220 | NADH--cytochrome b5 reductase 1-like |
| 218 | Contig1224 | alpha-1,4-glucan-protein synthase [UDP-forming] 2 |
| 219 | Contig1228 | protein IRX15-LIKE |
| 220 | Contig1230 | 60S ribosomal protein L15 |
| 221 | Contig1241 | ABC transporter I family member 11, chloroplastic isoform X1 |
| 222 | Contig1244 | cAMP-binding protein 1-like |
| 223 | Contig1246 | 60S acidic ribosomal protein P0 |
| 224 | Contig1247 | prohibitin-3, mitochondrial |
| 225 | Contig1249 | BTB/POZ domain-containing protein POB1-like |
| 226 | Contig1250 | isocitrate dehydrogenase [NAD] catalytic subunit 5, mitochondrial |
| 227 | Contig1257 | peroxisomal membrane protein PEX14-like isoform X1 |
| 228 | Contig1259 | embryogenic cell protein 40-like |
| 229 | Contig1263 | 50S ribosomal protein L27, chloroplastic |
| 230 | Contig1268 | glycine-rich family protein |
| 231 | Contig1276 | multiple organellar RNA editing factor 2, chloroplastic-like |
| 232 | Contig1278 | single-stranded DNA-binding protein WHY1, chloroplastic-like isoform X1 |
| 233 | Contig1284 | probable pectinesterase/pectinesterase inhibitor 51 |
| 234 | Contig1285 | probable calcium-binding protein CML36 |
| 235 | Contig1286 | serine hydroxymethyltransferase 4 |
| 236 | Contig1289 | conserved hypothetical protein |
| 237 | Contig1290 | 40S ribosomal protein S10-1-like |
| 238 | Contig1296 | reactive Intermediate Deaminase A, chloroplastic |
| 239 | Contig1298 | syntaxin-22-like |
| 240 | Contig1299 | probable calcium-binding protein CML13 |
| 241 | Contig1303 | acetolactate synthase 3, chloroplastic |
| 242 | Contig1317 | 60S ribosomal protein L11 |
| 243 | Contig1340 | 36.4 kDa proline-rich protein |
| 244 | Contig1342 | oil body-associated protein 1A |
| 245 | Contig1345 | mitochondrial import inner membrane translocase subunit TIM17-2-like |
| 246 | Contig1350 | malate dehydrogenase, mitochondrial |
| 247 | Contig1354 | fasciclin-like arabinogalactan protein 2 |
| 248 | Contig1355 | 3-ketoacyl-CoA thiolase 2, peroxisomal |
| 249 | Contig1356 | ADP,ATP carrier protein 3, mitochondrial |
| 250 | Contig1364 | hexokinase-2 |
| 251 | Contig1372 | probable acetyltransferase NATA1-like |
| 252 | Contig1374 | heat shock cognate 70 kDa protein 2-like |
| 253 | Contig1376 | eukaryotic translation initiation factor 5A |
| 254 | Contig1384 | aspartate-semialdehyde dehydrogenase |
| 255 | Contig1385 | fasciclin-like arabinogalactan protein 17 |
| 256 | Contig1389 | dna oxidative demethylase alkbh2 |
| 257 | Contig1397 | 60S ribosomal protein L3 |
| 258 | Contig1398 | pollen-specific protein C13-like |
| 259 | Contig1401 | DEAD-box ATP-dependent RNA helicase 8-like |
| 260 | Contig1409 | mitogen-activated protein kinase homolog MMK1 |
| 261 | Contig1414 | 40S ribosomal protein S2-4-like |
| 262 | Contig1429 | 40S ribosomal protein S5-like |
| 263 | Contig1431 | eukaryotic translation initiation factor |
| 264 | Contig1436 | succinate dehydrogenase subunit 5, mitochondrial |
| 265 | Contig1441 | ABC transporter F family member 1-like |
| 266 | Contig1446 | trihelix transcription factor ASR3 |
| 267 | Contig1456 | legumin A |
| 268 | Contig1457 | allene oxide cyclase, chloroplastic-like |
| 269 | Contig1460 | protein TRIGALACTOSYLDIACYLGLYCEROL 4, chloroplastic |
| 270 | Contig1463 | 60S ribosomal protein L8 |
| 271 | Contig1469 | protein TIFY 6B-like isoform X1 |
| 272 | Contig1470 | PREDICTED: uncharacterized protein LOC8287412 |
| 273 | Contig1474 | REF/SRPP-like protein At3g05500 |
| 274 | Contig1475 | pyruvate dehydrogenase E1 component subunit beta-3, chloroplastic |
| 275 | Contig1476 | methylesterase 3-like |
| 276 | Contig1478 | splicing factor 3A subunit 2 |
| 277 | Contig1480 | fructose-bisphosphate aldolase 3, chloroplastic |
| 278 | Contig1486 | cytidine deaminase 1-like |
| 279 | Contig1488 | FAM10 family protein At4g22670 |
| 280 | Contig1491 | PREDICTED: uncharacterized protein LOC8267322 |
| 281 | Contig1494 | 60S ribosomal protein L8 |
| 282 | Contig1499 | mitochondrial dicarboxylate/tricarboxylate transporter DTC |
| 283 | Contig1503 | polyadenylate-binding protein 2 |
| 284 | Contig1504 | lysine-rich arabinogalactan protein 19 |
| 285 | Contig1508 | serine/arginine-rich splicing factor SR45a isoform X2 |
| 286 | Contig1513 | NADH dehydrogenase [ubiquinone] flavoprotein 1, mitochondrial |
| 287 | Contig1518 | 5-methyltetrahydropteroyltriglutamate--homocysteine methyltransferase |
| 288 | Contig1523 | UMP-CMP kinase 3-like isoform X1 |
| 289 | Contig1524 | ring finger containing protein, putative |
| 290 | Contig1527 | guanine nucleotide-binding protein subunit beta-like protein |
| 291 | Contig1529 | D-aminoacyl-tRNA deacylase |
| 292 | Contig1532 | extradiol ring-cleavage dioxygenase |
| 293 | Contig1537 | Coproporphyrinogen-III oxidase, aerobic |
| 294 | Contig1538 | probable 3-hydroxyisobutyrate dehydrogenase-like 1, mitochondrial |
| 295 | Contig1539 | thiamine thiazole synthase, chloroplastic |
| 296 | Contig1546 | translation initiation factor eIF-2B subunit alpha-like |
| 297 | Contig1547 | protein EXORDIUM-like 5 |
| 298 | Contig1548 | methionine gamma-lyase |
| 299 | Contig1550 | clathrin light chain 1-like |
| 300 | Contig1559 | tubulin beta-5 chain |
| 301 | Contig1562 | 29 kDa ribonucleoprotein A, chloroplastic-like |
| 302 | Contig1566 | proline-rich extensin-like protein EPR1 |
| 303 | Contig1574 | S-adenosylmethionine synthase 1 |
| 304 | Contig1580 | PREDICTED: uncharacterized protein LOC8286853 |
| 305 | Contig1584 | 3'-5' exoribonuclease 1-like |
| 306 | Contig1586 | PREDICTED: uncharacterized protein At5g39570 |
| 307 | Contig1592 | ---NA--- |
| 308 | Contig1598 | homoserine kinase |
| 309 | Contig1602 | peroxidase 17-like |
| 310 | Contig1608 | BOI-related E3 ubiquitin-protein ligase 1-like |
| 311 | Contig1609 | ras-interacting protein RIP3 |
| 312 | Contig1612 | delta(3,5)-Delta(2,4)-dienoyl-CoA isomerase, peroxisomal |
| 313 | Contig1616 | polygalacturonase 1 beta-like protein 3 |
| 314 | Contig1617 | 3-oxo-Delta(4,5)-steroid 5-beta-reductase-like |
| 315 | Contig1619 | non-classical arabinogalactan protein 31-like |
| 316 | Contig1621 | ethylene-responsive transcription factor RAP2-3-like |
| 317 | Contig1622 | legumin A |
| 318 | Contig1628 | clavaminate synthase-like protein At3g21360 |
| 319 | Contig1629 | translocase subunit seca |
| 320 | Contig1630 | proline-rich extensin-like protein EPR1 |
| 321 | Contig1636 | asparagine--tRNA ligase, cytoplasmic 1-like |
| 322 | Contig1639 | 26S proteasome non-ATPase regulatory subunit 1 homolog A-like |
| 323 | Contig1644 | probable pectinesterase/pectinesterase inhibitor 34 |
| 324 | Contig1645 | tryptophan--tRNA ligase, cytoplasmic |
| 325 | Contig1654 | aspartyl protease family protein 2 |
| 326 | Contig1657 | autophagy-related protein 18a-like |
| 327 | Contig1659 | nucleolin 1-like isoform X1 |
| 328 | Contig1662 | conserved oligomeric Golgi complex subunit 8-like isoform X1 |
| 329 | Contig1663 | embryonic protein DC-8 |
| 330 | Contig1680 | ---NA--- |
| 331 | Contig1682 | subtilisin-like protease SBT1.5 |
| 332 | Contig1685 | probable fructokinase-4 |
| 333 | Contig1694 | S-adenosylmethionine synthase 3 |
| 334 | Contig1696 | expansin-like A2 |
| 335 | Contig1702 | V-type proton ATPase 16 kDa proteolipid subunit |
| 336 | Contig1707 | probable aquaporin TIP3-2 |
| 337 | Contig1709 | KH domain-containing protein At1g09660/At1g09670-like isoform X1 |
| 338 | Contig1714 | mitochondrial import inner membrane translocase subunit TIM23-2-like |
| 339 | Contig1715 | protein ALP1-like |
| 340 | Contig1728 | phenylalanine ammonia-lyase |
| 341 | Contig1730 | mitochondrial import inner membrane translocase subunit TIM22-2-like |
| 342 | Contig1734 | protein eyes shut |
| 343 | Contig1736 | ranBP2-type zinc finger protein At1g67325 isoform X2 |
| 344 | Contig1739 | PREDICTED: uncharacterized protein LOC8281317 |
| 345 | Contig1745 | 60S ribosomal protein L19-3 |
| 346 | Contig1748 | Heat shock protein 70 |
| 347 | Contig1749 | zinc finger protein ZAT10-like |
| 348 | Contig1754 | transcription factor TGA9-like isoform X1 |
| 349 | Contig1757 | zinc finger A20 and AN1 domain-containing stress-associated protein 3 |
| 350 | Contig1762 | Patellin-3, putative |
| 351 | Contig1770 | F-box protein At3g07870-like |
| 352 | Contig1781 | ethylene-responsive transcription factor ERF109-like |
| 353 | Contig1782 | PsbD mRNA maturation factor Nac2, chloroplastic |
| 354 | Contig1784 | Replicase polyprotein 1ab |
| 355 | Contig1786 | protein SLOW GREEN 1, chloroplastic-like |
| 356 | Contig1796 | DUF4228 domain-containing protein |
| 357 | Contig1797 | probable E3 ubiquitin-protein ligase LOG2 |
| 358 | Contig1799 | WAT1-related protein At4g08290 |
| 359 | Contig1804 | aquaporin TIP2-1 |
| 360 | Contig1805 | DELLA protein SLR1-like |
| 361 | Contig1808 | telomere repeat-binding protein 5 isoform X1 |
| 362 | Contig1812 | aspartyl protease AED3-like |
| 363 | Contig1813 | aquaporin TIP1-1 |
| 364 | Contig1821 | 3-oxoacyl-[acyl-carrier-protein] reductase FabG |
| 365 | Contig1824 | 40S ribosomal protein SA-like |
| 366 | Contig1825 | probable calcium-binding protein CML43 |
| 367 | Contig1838 | Plastocyanin-like protein |
| 368 | Contig1843 | putative beta-D-xylosidase |
| 369 | Contig1847 | probable aquaporin NIP5-1 |
| 370 | Contig1849 | terpene synthase 10-like |
| 371 | Contig1850 | ethylene-responsive transcription factor 2-like |
| 372 | Contig1868 | probable xyloglucan endotransglucosylase/hydrolase protein 28 |
| 373 | Contig1869 | protein SRC2 |
| 374 | Contig1872 | polyadenylate-binding protein 2-like |
| 375 | Contig1874 | sarcoplasmic reticulum histidine-rich calcium-binding protein-like |
| 376 | Contig1875 | transcription factor VIP1-like |
| 377 | Contig1883 | aldo-keto reductase family 4 member C9-like |
| 378 | Contig1885 | equilibrative nucleotide transporter 1 |
| 379 | Contig1886 | putative late blight resistance protein homolog R1B-19 |
| 380 | Contig1893 | phosphoenolpyruvate carboxykinase [ATP]-like |
| 381 | Contig1899 | protein DCL, chloroplastic |
| 382 | Contig1901 | lipoamide acyltransferase component of branched-chain alpha-keto acid dehydrogenase complex, mitochondrial |
| 383 | Contig1907 | F-box protein At5g50450-like |
| 384 | Contig1908 | glycine-rich RNA-binding protein-like |
| 385 | Contig1911 | cleft lip and palate transmembrane protein 1 homolog |
| 386 | Contig1919 | probable WRKY transcription factor 25 |
| 387 | Contig1922 | transcription factor MYB44-like |
| 388 | Contig1930 | remorin 4.1-like |
| 389 | Contig1933 | mitochondrial uncoupling protein 5 |
| 390 | Contig1937 | 24-methylenesterol C-methyltransferase 2 |
| 391 | Contig1940 | tubby-like F-box protein 8 |
| 392 | Contig1942 | glycine-rich protein A3 |
| 393 | Contig1947 | ATP synthase subunit beta, mitochondrial |
| 394 | Contig1958 | probable aquaporin TIP1-2 |
| 395 | Contig1960 | zinc finger protein ZAT10-like |
| 396 | Contig1963 | B-box zinc finger protein 21-like |
| 397 | Contig1965 | ethylene-responsive transcription factor 2-like |
| 398 | Contig1966 | transketolase, chloroplastic |
| 399 | Contig1972 | serine/arginine-rich splicing factor RS40 isoform X1 |
| 400 | Contig1981 | WD repeat-containing protein LWD1 |
| 401 | Contig1988 | heterogeneous nuclear ribonucleoprotein 1-like |
| 402 | Contig1989 | heat stress transcription factor C-1-like |
| 403 | Contig1996 | conserved hypothetical protein |
| 404 | Contig2001 | peptidyl-prolyl cis-trans isomerase CYP63 isoform X1 |
| 405 | Contig2002 | cyclin-dependent kinase inhibitor 5 |
| 406 | Contig2005 | terpene synthase 10-like |
| 407 | Contig2006 | chromatin remodeling protein SHL |
| 408 | Contig2017 | lysine--tRNA ligase, cytoplasmic-like |
| 409 | Contig2019 | ethylene-responsive transcription factor 5-like |
| 410 | Contig2021 | ATP synthase subunit b', chloroplastic |
| 411 | Contig2031 | zinc finger CCCH domain-containing protein 49-like |
| 412 | Contig2038 | Zinc finger protein, putative isoform 1 |
| 413 | Contig2040 | FRIGIDA-like protein 4a |
| 414 | Contig2041 | dormancy-associated protein homolog 3 isoform X1 |
| 415 | Contig2046 | nematode resistance protein-like HSPRO2 |
| 416 | Contig2049 | ATP-dependent zinc metalloprotease FTSH, chloroplastic |
| 417 | Contig2063 | putative ER lumen protein-retaining receptor C28H8.4 |
| 418 | Contig2066 | Serine-rich protein-related |
| 419 | Contig2069 | probable E3 ubiquitin-protein ligase RHC1A |
| 420 | Contig2071 | dr1-associated corepressor homolog |
| 421 | Contig2074 | protein MODIFIER OF SNC1 11-like |
| 422 | Contig2077 | peroxidase 2-like |
| 423 | Contig2084 | 2-isopropylmalate synthase 2, chloroplastic-like |
| 424 | Contig2086 | Avr9/Cf-9 rapidly elicited protein |
| 425 | Contig2090 | S-adenosylmethionine decarboxylase proenzyme-like |
| 426 | Contig2101 | histone H3.v1 |
| 427 | Contig2109 | probable E3 ubiquitin-protein ligase LUL4 |
| 428 | Contig2113 | reticulon-like protein B2 |
| 429 | Contig2120 | CASP-like protein 1D1 |
| 430 | Contig2123 | E3 ubiquitin-protein ligase RING1 |
| 431 | Contig2125 | PREDICTED: uncharacterized protein LOC107261088 |
| 432 | Contig2126 | monothiol glutaredoxin-S17 |
| 433 | Contig2131 | serine carboxypeptidase-like 13 |
| 434 | Contig2132 | blue copper protein-like |
| 435 | Contig2143 | chromatin remodeling protein EBS |
| 436 | Contig2146 | S-adenosyl-L-homocysteine hydrolase |
| 437 | Contig2150 | U-box domain-containing protein 28-like |
| 438 | Contig2151 | gamma carbonic anhydrase 1, mitochondrial |
| 439 | Contig2154 | ethylene-responsive transcription factor 4-like |
| 440 | Contig2186 | sucrose transport protein SUC4-like |
| 441 | Contig2191 | transcription factor MYB1R1 |
| 442 | Contig2192 | UPF0496 protein 4-like |
| 443 | Contig2202 | probable WRKY transcription factor 15 isoform X2 |
| 444 | Contig2203 | protein RETICULATA-RELATED 1, chloroplastic |
| 445 | Contig2205 | probable aquaporin PIP2-2 |
| 446 | Contig2209 | PREDICTED: uncharacterized protein LOC8280088 |
| 447 | Contig2213 | synaptotagmin-5-like |
| 448 | Contig2219 | L-ascorbate peroxidase, cytosolic |
| 449 | Contig2222 | xyloglucan endotransglucosylase/hydrolase protein 9 |
| 450 | Contig2228 | THO complex subunit 4D isoform X1 |
| 451 | Contig2230 | quinone oxidoreductase PIG3 |
| 452 | Contig2233 | heat shock factor protein HSF24 |
| 453 | Contig2234 | glycine-rich protein |
| 454 | Contig2240 | cysteine and histidine-rich domain-containing protein RAR1 |
| 455 | Contig2241 | ribonuclease P protein subunit p25-like protein isoform X1 |
| 456 | Contig2247 | glycine-rich RNA-binding protein RZ1A |
| 457 | Contig2248 | putative MO25-like protein At5g47540 |
| 458 | Contig2251 | nuclear transcription factor Y subunit A-3-like isoform X1 |
| 459 | Contig2272 | ethylene-responsive transcription factor ERF106-like |
| 460 | Contig2277 | delta-aminolevulinic acid dehydratase 1, chloroplastic |
| 461 | Contig2280 | casein kinase II subunit alpha |
| 462 | Contig2284 | transcription factor bHLH149-like |
| 463 | Contig2285 | zinc finger A20 and AN1 domain-containing stress-associated protein 3 |
| 464 | Contig2288 | GEM-like protein 5 |
| 465 | Contig2292 | branchpoint-bridging protein |
| 466 | Contig2293 | hypothetical protein L484_002552 |
| 467 | Contig2298 | protein EXORDIUM-like |
| 468 | Contig2299 | mitogen-activated protein kinase kinase 5 |
| 469 | Contig2303 | probable mitochondrial-processing peptidase subunit beta, mitochondrial |
| 470 | Contig2306 | 60S ribosomal protein L19-3 |
| 471 | Contig2307 | CBL-interacting serine/threonine-protein kinase 11 |
| 472 | Contig2316 | DELLA protein GAI |
| 473 | Contig2318 | VQ motif-containing protein 4-like |
| 474 | Contig2319 | GATA transcription factor 1-like |
| 475 | Contig2321 | abscisic acid receptor PYL4-like |
| 476 | Contig2336 | nuclear transcription factor Y subunit C-2 |
| 477 | Contig2341 | syntaxin-121-like |
| 478 | Contig2348 | heavy metal-associated isoprenylated plant protein 5-like |
| 479 | Contig2358 | terpene synthase 10-like |
| 480 | Contig2372 | putative quinone-oxidoreductase homolog, chloroplastic |
| 481 | Contig2373 | mitochondrial import inner membrane translocase subunit TIM22-like |
| 482 | Contig2384 | superoxide dismutase [Mn], mitochondrial |
| 483 | Contig2385 | ubiquitin-conjugating enzyme E2-17 kDa |
| 484 | Contig2386 | cinnamoyl-CoA reductase 1-like |
| 485 | Contig2394 | eukaryotic translation initiation factor 3 subunit F-like |
| 486 | Contig2396 | pentatricopeptide repeat-containing protein At2g31400, chloroplastic |
| 487 | Contig2403 | protein LIGHT-DEPENDENT SHORT HYPOCOTYLS 4-like |
| 488 | Contig2404 | 3-hydroxybutyryl-CoA dehydrogenase |
| 489 | Contig2409 | polyadenylate-binding protein 8-like |
| 490 | Contig2410 | polyadenylate-binding protein 8-like |
| 491 | Contig2415 | plasma membrane-associated cation-binding protein 1 |
| 492 | Contig2421 | histone H1 |
| 493 | Contig2429 | mediator of RNA polymerase II transcription subunit 36a-like |
| 494 | Contig2437 | splicing factor U2af small subunit B-like |
| 495 | Contig2441 | protein-tyrosine-phosphatase MKP1-like |
| 496 | Contig2443 | transcription factor bHLH66-like |
| 497 | Contig2452 | Elongation factor 1-beta 1 |
| 498 | Contig2456 | scarecrow-like protein 21 |
| 499 | Contig2458 | glycine-rich RNA-binding protein 2, mitochondrial |
| 500 | Contig2463 | putative Polycomb group protein ASXL3 |
| 501 | Contig2466 | PREDICTED: uncharacterized protein LOC8267007 |
| 502 | Contig2475 | LOB domain-containing protein 41-like |
| 503 | Contig2503 | putative late blight resistance protein homolog R1B-19 |
| 504 | Contig2510 | galactan beta-1,4-galactosyltransferase GALS3-like |
| 505 | Contig2514 | NAC domain-containing protein 2 |
| 506 | Contig2521 | zinc finger protein ZAT10-like |
| 507 | Contig2522 | aspartyl protease family protein At5g10770-like |
| 508 | Contig2524 | transcription initiation factor TFIID subunit 15b |
| 509 | Contig2529 | classical arabinogalactan protein 5 |
| 510 | Contig2533 | aspartate aminotransferase, cytoplasmic |
| 511 | Contig2534 | Zinc finger, RING-type |
| 512 | Contig2535 | CASP-like protein 4A3 |
| 513 | Contig2537 | tetraspanin-8-like |
| 514 | Contig2539 | polyubiquitin 10 |
| 515 | Contig2543 | His_Phos_1 domain-containing protein |
| 516 | Contig2545 | rac-like GTP-binding protein 5 |
| 517 | Contig2548 | nuclear/nucleolar GTPase 2 |
| 518 | Contig2553 | C2 calcium-dependent membrane targeting |
| 519 | Contig2556 | polyadenylate-binding protein RBP45C-like isoform X1 |
| 520 | Contig2577 | casein kinase II subunit alpha-2-like |
| 521 | Contig2584 | tubulin beta-5 chain |
| 522 | Contig2586 | probable receptor-like protein kinase At5g24010 |
| 523 | Contig2587 | probable WRKY transcription factor 25 |
| 524 | Contig2590 | protein SUPPRESSOR OF K(+) TRANSPORT GROWTH DEFECT 1 |
| 525 | Contig2592 | sorbitol dehydrogenase |
| 526 | Contig2596 | PREDICTED: uncharacterized protein LOC8287714 |
| 527 | Contig2598 | stromal cell-derived factor 2-like protein |
| 528 | Contig2599 | THO complex subunit 4D isoform X1 |
| 529 | Contig2604 | protein NRT1/ PTR FAMILY 7.3 |
| 530 | Contig2605 | PREDICTED: uncharacterized protein LOC8274216 |
| 531 | Contig2609 | HMG-Y-related protein B-like |
| 532 | Contig2610 | purine permease 3-like |
| 533 | Contig2611 | transcription factor UNE12-like |
| 534 | Contig2616 | linoleate 13S-lipoxygenase 3-1, chloroplastic-like |
| 535 | Contig2618 | 40S ribosomal protein S17-like |
| 536 | Contig2626 | conserved hypothetical protein |
| 537 | Contig2630 | ABC transporter F family member 1-like |
| 538 | Contig2633 | expansin-like B1 |
| 539 | Contig2636 | protein LHY-like isoform X1 |
| 540 | Contig2651 | WD repeat-containing protein VIP3 |
| 541 | Contig2661 | probable carboxylesterase 15 |
| 542 | Contig2665 | F-box/kelch-repeat protein At1g80440 |
| 543 | Contig2675 | late embryogenesis abundant protein At5g17165-like |
| 544 | Contig2679 | magnesium transporter MRS2-4 |
| 545 | Contig2682 | F-box/kelch-repeat protein SKIP11-like |
| 546 | Contig2686 | AT-hook motif nuclear-localized protein 19 |
| 547 | Contig2691 | la-related protein 6A |
| 548 | Contig2702 | protein JINGUBANG |
| 549 | Contig2707 | glycine-rich protein A3 |
| 550 | Contig2711 | ethylene-responsive transcription factor ERF105-like |
| 551 | Contig2713 | tubby-like F-box protein 7 |
| 552 | Contig2719 | terpene synthase 10-like |
| 553 | Contig2721 | ethylene-responsive transcription factor 9 |
| 554 | Contig2722 | chitin-inducible gibberellin-responsive protein 1-like |
| 555 | Contig2726 | HMG-Y-related protein A-like |
| 556 | Contig2735 | oxalate--CoA ligase-like |
| 557 | Contig2737 | cyclin-dependent kinase 12 isoform X1 |
| 558 | Contig2741 | probable carboxylesterase 15 |
| 559 | Contig2742 | 60S ribosomal protein L4 |
| 560 | Contig2746 | calcineurin B-like protein-interacting protein kinase |
| 561 | Contig2752 | aquaporin transporter, putative |
| 562 | Contig2757 | ---NA--- |
| 563 | Contig2761 | hsp70-Hsp90 organizing protein 3 |
| 564 | Contig2765 | protein RTF2 homolog |
| 565 | Contig2771 | UBP1-associated protein 2A |
| 566 | Contig2775 | oligouridylate-binding protein 1B |
| 567 | Contig2778 | PREDICTED: uncharacterized protein LOC8267007 |
| 568 | Contig2786 | splicing factor 3A subunit 2 |
| 569 | Contig2789 | serine/arginine-rich splicing factor SR30 isoform X1 |
| 570 | Contig2800 | kynurenine formamidase |
| 571 | Contig2801 | putative laccase-9 |
| 572 | Contig2813 | 2-isopropylmalate synthase 2, chloroplastic-like |
| 573 | Contig2814 | pre-mRNA-splicing factor 18 |
| 574 | Contig2816 | mitochondrial uncoupling protein 5 |
| 575 | Contig2823 | far upstream element-binding protein 1-like |
| 576 | Contig2824 | far upstream element-binding protein 1-like |
| 577 | Contig2830 | PREDICTED: uncharacterized protein LOC107262380 |
| 578 | Contig2831 | late embryogenesis abundant protein D-34 |
| 579 | Contig2835 | pyrophosphate-energized vacuolar membrane proton pump-like |
| 580 | Contig2841 | terpene synthase 10-like |
| 581 | Contig2842 | terpene synthase 10-like |
| 582 | Contig2843 | acetolactate synthase 3, chloroplastic |
| 583 | Contig2857 | aconitate hydratase, cytoplasmic |
| 584 | Contig2867 | hypothetical protein GLYMA_13G020100 |
| 585 | Contig2872 | heat stress transcription factor B-2a |
| 586 | Contig2874 | protein BASIC PENTACYSTEINE6 |
| 587 | Contig2882 | probable CCR4-associated factor 1 homolog 9 |
| 588 | Contig2888 | plasminogen activator inhibitor 1 RNA-binding protein |
| 589 | Contig2897 | probable GTP diphosphokinase RSH2, chloroplastic isoform X4 |
| 590 | Contig2900 | calmodulin-binding protein 25-like |
| 591 | Contig2908 | phospholipase A1-Ibeta2, chloroplastic |
| 592 | Contig2912 | DUF1644 domain-containing protein |
| 593 | Contig2913 | telomere repeat-binding protein 3-like |
| 594 | Contig2914 | classical arabinogalactan protein 9-like |
| 595 | Contig2918 | protein KINESIN LIGHT CHAIN-RELATED 1-like |
| 596 | Contig2925 | primary amine oxidase-like |
| 597 | Contig2937 | 50S ribosomal protein L18 |
| 598 | Contig2938 | probable E3 ubiquitin-protein ligase RHC2A |
| 599 | Contig2942 | terpene synthase 10-like |
| 600 | Contig2946 | zinc finger CCCH domain-containing protein 66-like |
| 601 | Contig2950 | UBP1-associated protein 2A-like |
| 602 | Contig2956 | 4-diphosphocytidyl-2-C-methyl-D-erythritol kinase, chloroplastic |
| 603 | Contig2972 | TOM1-like protein 4 isoform X1 |
| 604 | Contig2979 | serine acetyltransferase 5 |
| 605 | Contig2980 | multiple organellar RNA editing factor 8, chloroplastic/mitochondrial |
| 606 | Contig2981 | DDT domain-containing protein DDB_G0282237-like isoform X2 |
| 607 | Contig2985 | C-repeat/dehydration-responsive element-binding factor 4 |
| 608 | Contig2987 | serine/arginine-rich splicing factor RSZ22A-like |
| 609 | Contig2993 | homeobox-leucine zipper protein HAT5 |
| 610 | Contig2999 | terpene synthase 10-like |
| 611 | Contig3004 | terpene synthase 10-like |
| 612 | Contig3007 | 3-oxo-Delta(4,5)-steroid 5-beta-reductase-like |
| 613 | Contig3015 | protein SRC2 homolog |
| 614 | Contig3019 | protein LURP-one-related 8 |
| 615 | Contig3023 | terpene synthase 10-like |
| 616 | Contig3025 | transcription factor TCP23 |
| 617 | Contig3027 | G-box-binding factor 4-like |
| 618 | Contig3028 | eukaryotic initiation factor 4A-3 |
| 619 | Contig3029 | indole-3-acetic acid-induced protein ARG2-like |
| 620 | Contig3032 | splicing factor 3A subunit 2 |
| 621 | Contig3035 | tobamovirus multiplication protein 2A-like |
| 622 | Contig3037 | dormancy-associated protein homolog 4 isoform X2 |
| 623 | Contig3039 | N-lysine methyltransferase |
| 624 | Contig3040 | E3 ubiquitin-protein ligase ATL6-like |
| 625 | Contig3044 | basic salivary proline-rich protein 3 |
| 626 | Contig3045 | haloacid dehalogenase-like hydrolase domain-containing protein 3 |
| 627 | Contig3049 | terpene synthase 10-like |
| 628 | Contig3050 | endoglucanase 25-like |
| 629 | Contig3053 | stem-specific protein TSJT1-like |
| 630 | Contig3055 | serine/threonine-protein kinase AFC2 isoform X3 |
| 631 | Contig3056 | probable glutamyl endopeptidase, chloroplastic |
| 632 | Contig3057 | diaminopimelate epimerase, chloroplastic |
| 633 | Contig3070 | vacuolar protein sorting-associated protein 28 homolog 1 |
| 634 | Contig3084 | caffeoylshikimate esterase |
| 635 | Contig3093 | polyubiquitin |
| 636 | Contig3108 | THO complex subunit 4A-like |
| 637 | Contig3113 | NADH dehydrogenase [ubiquinone] iron-sulfur protein 7, mitochondrial |
| 638 | Contig3115 | ---NA--- |
| 639 | Contig3117 | 14-3-3-like protein |
| 640 | Contig3120 | terpene synthase 10-like |
| 641 | Contig3138 | eukaryotic initiation factor 4A-3 |
| 642 | Contig3147 | tubby-like F-box protein 5 |
| 643 | Contig3150 | terpene synthase 10-like |
| 644 | Contig3160 | ethylene-responsive transcription factor RAP2-12-like |
| 645 | Contig3164 | nitrile-specifier protein 5 |
| 646 | Contig3168 | protein GLUTAMINE DUMPER 2-like |
| 647 | Contig3181 | heat stress transcription factor B-3-like |
| 648 | Contig3183 | transcription factor MYB44-like |
| 649 | Contig3195 | U-box domain-containing protein 26-like |
| 650 | Contig3197 | coiled-coil domain-containing protein 124 |
| 651 | Contig3199 | trihelix transcription factor ASR3 |
| 652 | Contig3203 | 14-3-3-like protein D isoform X2 |
| 653 | Contig3205 | probable aquaporin TIP-type |
| 654 | Contig3220 | zinc finger protein CONSTANS-LIKE 5 |
| 655 | Contig3222 | monodehydroascorbate reductase |
| 656 | Contig3225 | zinc finger CCCH domain-containing protein 20 |
| 657 | Contig3237 | KH domain-containing protein At1g09660/At1g09670-like isoform X1 |
| 658 | Contig3247 | U1 small nuclear ribonucleoprotein 70 kDa |
| 659 | Contig3258 | heat shock cognate 70 kDa protein 2 |
| 660 | Contig3259 | BOI-related E3 ubiquitin-protein ligase 1-like |
| 661 | Contig3262 | small nuclear ribonucleoprotein-associated protein B'-like |
| 662 | Contig3267 | probable ribose-5-phosphate isomerase 2 |
| 663 | Contig3270 | tonoplast dicarboxylate transporter |
| 664 | Contig3284 | zinc finger protein ZAT10-like |
| 665 | Contig3285 | serpin-ZX |
| 666 | Contig3289 | protein EXORDIUM-like 2 |
| 667 | Contig3302 | glycerol-3-phosphate dehydrogenase [NAD(+)] 2, chloroplastic |
| 668 | Contig3305 | alcohol dehydrogenase 1 |
| 669 | Contig3306 | patellin-3 |
| 670 | Contig3308 | S-adenosyl-L-methionine-dependent methyltransferase |
| 671 | Contig3315 | AT-hook motif nuclear-localized protein 22-like |
| 672 | Contig3327 | peptide methionine sulfoxide reductase A1-like |
| 673 | Contig3333 | Late embryogenesis abundant protein, LEA-14 |
| 674 | Contig3334 | senescence-associated family protein |
| 675 | Contig3338 | transcriptional corepressor SEUSS |
| 676 | Contig3343 | VQ motif-containing protein 9 |
| 677 | Contig3349 | protein-lysine N-methyltransferase Mettl10 |
| 678 | Contig3351 | ubiquinol oxidase, mitochondrial-like |
| 679 | Contig3365 | abscisic acid receptor PYL4-like |
| 680 | Contig3367 | probable xyloglucan endotransglucosylase/hydrolase protein 30 |
| 681 | Contig3380 | ATP synthase mitochondrial F1 complex assembly factor 2 |
| 682 | Contig3382 | pfkB-type carbohydrate kinase family protein |
| 683 | Contig3384 | peroxidase 12-like |
| 684 | Contig3387 | 60S ribosomal protein L13a-4 |
| 685 | Contig3393 | probable adenylate kinase 7, mitochondrial |
| 686 | Contig3394 | dormancy-associated protein homolog 4 isoform X1 |
| 687 | Contig3400 | ubiquitin receptor RAD23c-like |
| 688 | Contig3404 | E3 ubiquitin-protein ligase CIP8 |
| 689 | Contig3409 | protein SRC2 homolog |
| 690 | Contig3416 | UPF0496 protein 1-like |
| 691 | Contig3424 | NDR1/HIN1-like protein 13 |
| 692 | Contig3431 | 40S ribosomal protein SA-like |
| 693 | Contig3432 | arogenate dehydratase/prephenate dehydratase 6, chloroplastic-like |
| 694 | Contig3436 | 26S proteasome non-ATPase regulatory subunit 4 homolog |
| 695 | Contig3438 | Rgp1 domain-containing protein |
| 696 | Contig3445 | 50S ribosomal protein L7/L12 isoform X1 |
| 697 | Contig3454 | endochitinase EP3 |
| 698 | Contig3456 | peptidyl-tRNA hydrolase ICT1, mitochondrial isoform X1 |
| 699 | Contig3463 | RING-H2 finger protein ATL2 |
| 700 | Contig3467 | PREDICTED: uncharacterized protein LOC8268543 |
| 701 | Contig3471 | RNA polymerase II degradation factor 1 |
| 702 | Contig3472 | CBL-interacting serine/threonine-protein kinase 6-like |
| 703 | Contig3486 | GDP-L-galactose phosphorylase 2 |
| 704 | Contig3487 | putative nucleic acid binding protein |
| 705 | Contig3488 | transcription factor MYBS3-like |
| 706 | Contig3491 | ubiquitin receptor RAD23d isoform X1 |
| 707 | Contig3501 | glyceraldehyde-3-phosphate dehydrogenase B, chloroplastic |
| 708 | Contig3520 | ribulose bisphosphate carboxylase small chain, chloroplastic-like |
| 709 | Contig3522 | nuclear transcription factor Y subunit C-9-like |
| 710 | Contig3527 | PRA1 family protein H-like isoform X1 |
| 711 | Contig3533 | oxygen-evolving enhancer protein 3-2, chloroplastic |
| 712 | Contig3539 | 28 kDa ribonucleoprotein, chloroplastic-like |
| 713 | Contig3540 | photosystem II 22 kDa protein, chloroplastic |
| 714 | Contig3549 | protochlorophyllide reductase-like |
| 715 | Contig3557 | serine--glyoxylate aminotransferase |
| 716 | Contig3563 | carbonic anhydrase 2 isoform X1 |
| 717 | Contig3576 | prenyl-dependent CAAX protease, putative |
| 718 | Contig3578 | proline-rich protein 4 |
| 719 | Contig3582 | glyoxylate/succinic semialdehyde reductase 1-like |
| 720 | Contig3585 | salicylic acid-binding protein 2-like |
| 721 | Contig3593 | alpha tubulin 1 |
| 722 | Contig3599 | probable aquaporin PIP2-8 |
| 723 | Contig3613 | pyruvate dehydrogenase E1 component subunit alpha-3, chloroplastic |
| 724 | Contig3620 | triphosphate tunel metalloenzyme 3-like |
| 725 | Contig3621 | glucose-6-phosphate/phosphate translocator 1, chloroplastic-like |
| 726 | Contig3627 | photosystem I reaction center subunit III, chloroplastic |
| 727 | Contig3636 | chlorophyll a-b binding protein 13, chloroplastic |
| 728 | Contig3646 | dnaJ homolog subfamily B member 6 |
| 729 | Contig3666 | chlorophyll a-b binding protein CP26, chloroplastic |
| 730 | Contig3669 | zinc finger A20 and AN1 domain-containing stress-associated protein 8-like |
| 731 | Contig3680 | PREDICTED: uncharacterized protein LOC8263555 |
| 732 | Contig3688 | transcription factor ILR3 |
| 733 | Contig3695 | protein SRC2 homolog |
| 734 | Contig3700 | oxygen-evolving enhancer protein 2, chloroplastic |
| 735 | Contig3702 | ribulose bisphosphate carboxylase/oxygenase activase, chloroplastic isoform X1 |
| 736 | Contig3706 | conserved hypothetical protein |
| 737 | Contig3709 | fructose-bisphosphate aldolase 1, chloroplastic |
| 738 | Contig3718 | diacylglycerol kinase 5-like |
| 739 | Contig3726 | RGG repeats nuclear RNA binding protein A-like |
| 740 | Contig3733 | vacuolar protein sorting-associated protein 32 homolog 2 |
| 741 | Contig3734 | chlorophyll a-b binding protein of LHCII type 1 |
| 742 | Contig3736 | sec-independent protein translocase protein TATC, chloroplastic |
| 743 | Contig3740 | chlorophyll a-b binding protein of LHCII type 1-like |
| 744 | Contig3741 | R3H domain-containing protein 1 |
| 745 | Contig3749 | phosphoglycerate kinase, cytosolic |
| 746 | Contig3750 | glycine-rich RNA-binding protein 2, mitochondrial |
| 747 | Contig3755 | sucrose synthase 2 |
| 748 | Contig3760 | homeobox-leucine zipper protein HAT4-like |
| 749 | Contig3761 | aquaporin AQPAn.G-like |
| 750 | Contig3763 | ribulose bisphosphate carboxylase small chain, chloroplastic-like |
| 751 | Contig3770 | 3-isopropylmalate dehydratase small subunit 3-like |
| 752 | Contig3774 | fructose-bisphosphate aldolase 1, chloroplastic |
| 753 | Contig3775 | probable aquaporin PIP-type 7a |
| 754 | Contig3778 | probable transaldolase |
| 755 | Contig3781 | triose phosphate/phosphate translocator TPT, chloroplastic isoform X1 |
| 756 | Contig3785 | fasciclin-like arabinogalactan protein 4 |
| 757 | Contig3786 | PLASMODESMATA CALLOSE-BINDING PROTEIN 3-like |
| 758 | Contig3792 | 60S ribosomal protein L10 |
| 759 | Contig3806 | glyceraldehyde-3-phosphate dehydrogenase, cytosolic |
| 760 | Contig3811 | mitochondrial carnitine/acylcarnitine carrier-like protein |
| 761 | Contig3820 | betaine aldehyde dehydrogenase 1, chloroplastic |
| 762 | Contig3841 | zinc finger A20 and AN1 domain-containing stress-associated protein 5 |
| 763 | Contig3844 | ATP synthase subunit delta, chloroplastic |
| 764 | Contig3852 | catalase isozyme 2 |
| 765 | Contig3862 | GDT1-like protein 1, chloroplastic isoform X1 |
| 766 | Contig3866 | uncharacterized LOC8278573 precursor |
| 767 | Contig3888 | B-box zinc finger protein 24-like |
| 768 | Contig3891 | auxin-responsive protein IAA9-like |
| 769 | Contig3895 | mitochondrial adenine nucleotide transporter ADNT1 |
| 770 | Contig3904 | leucine-rich repeat receptor-like protein |
| 771 | Contig3910 | nuclear transcription factor Y subunit C-9-like |
| 772 | Contig3924 | chlorophyll a-b binding protein 151, chloroplastic |
| 773 | Contig3925 | 2-Cys peroxiredoxin BAS1, chloroplastic |
| 774 | Contig3929 | BTB/POZ domain-containing protein At1g63850-like |
| 775 | Contig3930 | phosphoglycerate kinase, chloroplastic |
| 776 | Contig3931 | chlorophyll a-b binding protein P4, chloroplastic |
| 777 | Contig3935 | transcription factor MYB1R1 |
| 778 | Contig3936 | histone deacetylase HDT1-like |
| 779 | Contig3941 | ADP-ribosylation factor 2 |
| 780 | Contig3945 | glutamine synthetase leaf isozyme, chloroplastic |
| 781 | Contig3955 | protein MARD1-like |
| 782 | Contig3958 | GTP-binding protein SAR1A |
| 783 | Contig3959 | low-temperature-induced cysteine proteinase |
| 784 | Contig3961 | 60S ribosomal protein L7a |
| 785 | Contig3967 | aquaporin PIP1-3 |
| 786 | Contig3976 | UDP-glucuronate 4-epimerase 1 |
| 787 | Contig3981 | 1-acyl-sn-glycerol-3-phosphate acyltransferase PLS1 |
| 788 | Contig3989 | 60S ribosomal protein L8 |
| 789 | Contig3994 | PREDICTED: uncharacterized protein LOC8273045 |
| 790 | Contig4012 | prostatic spermine-binding protein-like |
| 791 | Contig4021 | thaumatin-like protein |
| 792 | Contig4032 | arginine decarboxylase |
| 793 | Contig4036 |  |
| 794 | Contig4038 | solute carrier family 25 member 44 |
| 795 | Contig4040 | chlorophyll a-b binding protein CP29.1, chloroplastic |
| 796 | Contig4046 | serine/arginine-rich splicing factor SR45a isoform X2 |
| 797 | Contig4053 | RNA-binding protein 1-like isoform X1 |
| 798 | Contig4061 | uncharacterized GPI-anchored protein At4g28100 |
| 799 | Contig4067 | NDR1/HIN1-like protein 10 |
| 800 | Contig4072 | alpha tubulin 1 |
| 801 | Contig4077 | DPP6 N-terminal domain-like protein |
| 802 | Contig4094 | acylpyruvase FAHD1, mitochondrial |
| 803 | Contig4103 | phospho-2-dehydro-3-deoxyheptonate aldolase 1, chloroplastic-like |
| 804 | Contig4107 | peptidyl-tRNA hydrolase ICT1, mitochondrial isoform X1 |
| 805 | Contig4110 | GDSL esterase/lipase At2g04570-like |
| 806 | Contig4113 | GDSL esterase/lipase At5g33370-like |
| 807 | Contig4114 | serine/arginine-rich splicing factor SR45 isoform X1 |
| 808 | Contig4120 | 40S ribosomal protein S6 |
| 809 | Contig4129 | polyubiquitin-like |
| 810 | Contig4134 | chlorophyll a-b binding protein CP24 10A, chloroplastic |
| 811 | Contig4139 | chitotriosidase-1-like isoform X2 |
| 812 | Contig4140 | ribonuclease 2 |
| 813 | Contig4153 | endo-1,3;1,4-beta-D-glucanase-like |
| 814 | Contig4162 | cellulose synthase A catalytic subunit 2 [UDP-forming] |
| 815 | Contig4164 | elongation factor 1-alpha-like |
| 816 | Contig4167 | voltage-dependent L-type calcium channel subunit |
| 817 | Contig4169 | alpha carbonic anhydrase 4-like |
| 818 | Contig4173 | cytochrome b-c1 complex subunit Rieske-4, mitochondrial-like |
| 819 | Contig4180 | phospholipase A1-IIdelta-like |
| 820 | Contig4181 | RNA binding protein, putative |
| 821 | Contig4184 | cysteine proteinase 15A-like |
| 822 | Contig4202 | chlorophyll a-b binding protein, chloroplastic |
| 823 | Contig4209 | serine/arginine-rich SC35-like splicing factor SCL33 |
| 824 | Contig4211 | annexin D1 |
| 825 | Contig4216 | nuclear transcription factor Y subunit C-1 |
| 826 | Contig4220 | 3-isopropylmalate dehydratase large subunit-like |
| 827 | Contig4239 | elongation factor 1-alpha |
| 828 | Contig4250 | carbonic anhydrase 2 |
| 829 | Contig4256 | uridine 5'-monophosphate synthase-like |
| 830 | Contig4266 | 40S ribosomal protein S9-2 |
| 831 | Contig4281 | basic leucine zipper and W2 domain-containing protein 2 |
| 832 | Contig4286 | nucleoside diphosphate kinase 2, chloroplastic |
| 833 | Contig4299 | peroxisomal (S)-2-hydroxy-acid oxidase |
| 834 | Contig4303 | malate dehydrogenase, glyoxysomal |
| 835 | Contig4315 | alcohol dehydrogenase class-3 |
| 836 | Contig4316 | malonyl CoA-acyl carrier protein transacylase |
| 837 | Contig4318 | probable serine/threonine-protein kinase At4g35230 |
| 838 | Contig4321 | S-adenosylmethionine synthase 1 |
| 839 | Contig4327 | universal stress protein PHOS34-like |
| 840 | Contig4339 | shaggy-related protein kinase eta |
| 841 | Contig4340 | RING-H2 finger protein ATL48-like |
| 842 | Contig4343 | v-type proton atpase subunit b2 |
| 843 | Contig4349 | MAR-binding filament-like protein 1 isoform 1 |
| 844 | Contig4358 | non-specific lipid-transfer protein 1-like |
| 845 | Contig4363 | phospholipid transfer protein 1 |
| 846 | Contig4380 | GPI-anchored protein LLG1-like |
| 847 | Contig4384 | PTI1-like tyrosine-protein kinase 1 isoform X1 |
| 848 | Contig4385 | BTB/POZ domain-containing protein POB1-like isoform X1 |
| 849 | Contig4389 | 40S ribosomal protein S23 |
| 850 | Contig4397 | auxin-responsive protein IAA9 |
| 851 | Contig4398 | photosystem I reaction center subunit XI, chloroplastic |
| 852 | Contig4400 | glyceraldehyde-3-phosphate dehydrogenase A, chloroplastic |
| 853 | Contig4407 | F-box protein At2g32560-like |
| 854 | Contig4409 | acyl carrier protein 1, chloroplastic-like |
| 855 | Contig4419 | soluble diacylglycerol acyltransferase |
| 856 | Contig4420 | auxin-responsive protein SAUR32 isoform X1 |
| 857 | Contig4426 | Pollen_allerg_1 domain-containing protein/DPBB_1 domain-containing protein |
| 858 | Contig4428 | PREDICTED: LOW QUALITY PROTEIN: uncharacterized protein LOC8259331 |
| 859 | Contig4435 | putative pectinesterase/pectinesterase inhibitor 26 |
| 860 | Contig4436 | pectinesterase PPME1-like |
| 861 | Contig4441 | Zinc finger, RING/FYVE/PHD-type |
| 862 | Contig4451 | bZIP transcription factor 11-like |
| 863 | Contig4452 | fructose-bisphosphate aldolase 6, cytosolic |
| 864 | Contig4453 | thioredoxin H-type |
| 865 | Contig4461 | mitochondrial fission 1 protein A |
| 866 | Contig4491 | protein transport protein Sec61 subunit alpha-like |
| 867 | Contig4493 | 60S ribosomal protein L11 |
| 868 | Contig4494 | PREDICTED: uncharacterized protein LOC8276123 |
| 869 | Contig4511 | phytochrome A-associated F-box protein |
| 870 | Contig4523 | dehydrin |
| 871 | Contig4529 | auxin-binding protein ABP19a-like |
| 872 | Contig4536 | peroxiredoxin family protein |
| 873 | Contig4547 | 14-3-3-like protein A |
| 874 | Contig4551 | ubiquitin carboxyl-terminal hydrolase 18-like |
| 875 | Contig4552 | KH domain-containing protein At5g56140 |
| 876 | Contig4563 | pentatricopeptide repeat-containing protein At1g01970 |
| 877 | Contig4565 | protein EXORDIUM-like 2 |
| 878 | Contig4566 | GLABROUS1 enhancer-binding protein-like |
| 879 | Contig4576 | GPN-loop GTPase 3 |
| 880 | Contig4578 | actin-depolymerizing factor 2 |
| 881 | Contig4581 | histone H3.3 |
| 882 | Contig4584 | AT-hook motif nuclear-localized protein 10-like isoform X1 |
| 883 | Contig4591 | exopolygalacturonase-like |
| 884 | Contig4606 | 2-alkenal reductase (NADP(+)-dependent) |
| 885 | Contig4616 | abscisic acid receptor PYL8 |
| 886 | Contig4618 | rac-like GTP-binding protein RHO1 |
| 887 | Contig4636 | endochitinase 2-like |
| 888 | Contig4648 | GATA transcription factor 11 isoform X2 |
| 889 | Contig4654 | chlorophyll a-b binding protein of LHCII type 1 |
| 890 | Contig4686 | 60S ribosomal protein L10 |
| 891 | Contig4691 | vacuolar-sorting receptor 1-like |
| 892 | Contig4701 | late embryogenesis abundant protein Lea5 |
| 893 | Contig4709 | acylamino-acid-releasing enzyme, putative |
| 894 | Contig4715 | thaumatin-like protein 1 |
| 895 | Contig4729 | biotin carboxyl carrier protein of acetyl-CoA carboxylase 2, chloroplastic |
| 896 | Contig4736 | protein TIFY 3B-like |
| 897 | Contig4743 | START domain containing protein |
| 898 | Contig4751 | 36.4 kDa proline-rich protein-like |
| 899 | Contig4752 | cytochrome b-c1 complex subunit Rieske-4, mitochondrial-like |
| 900 | Contig4758 | Self-incomp_S1 domain-containing protein |
| 901 | Contig4764 | 60S ribosomal protein L35a-1 |
| 902 | Contig4767 | vacuolar cation/proton exchanger 3 |
| 903 | Contig4778 | beta-carotene hydroxylase 2, chloroplastic-like |
| 904 | Contig4791 | protein ELC-like |
| 905 | Contig4792 | BTB/POZ and TAZ domain-containing protein 4 |
| 906 | Contig4808 | L-ascorbate peroxidase, cytosolic |
| 907 | Contig4819 | S-adenosylmethionine decarboxylase proenzyme-like |
| 908 | Contig4820 | NAC domain-containing protein 72 |
| 909 | Contig4821 | protein trichome birefringence-like 38 |
| 910 | Contig4827 | PREDICTED: uncharacterized protein LOC8259965 |
| 911 | Contig4855 | 40S ribosomal protein S19-3 |
| 912 | Contig4867 | ABC transporter F family member 1 |
| 913 | Contig4904 | auxin-responsive protein IAA16 |
| 914 | Contig4905 | fasciclin-like arabinogalactan protein 13 |
| 915 | Contig4915 | serine carboxypeptidase-like 18 |
| 916 | Contig4929 | ATP synthase subunit gamma, mitochondrial |
| 917 | Contig4930 | calmodulin-7 |
| 918 | Contig4948 | selenoprotein K-like |
| 919 | Contig4951 | RNA-binding protein with multiple splicing |
| 920 | Contig4957 | dihydrolipoyl dehydrogenase 1, mitochondrial |
| 921 | Contig4962 | PREDICTED: uncharacterized protein LOC8288304 isoform X1 |
| 922 | Contig4970 | putative pectinesterase 63 |
| 923 | Contig4971 | high mobility group B protein 2-like |
| 924 | Contig4976 | ubiquitin receptor RAD23d isoform X1 |
| 925 | Contig4995 | glycine cleavage system H protein 2, mitochondrial-like |
| 926 | Contig5000 | 60S ribosomal protein L35 |
| 927 | Contig5027 | classical arabinogalactan protein 6 |
| 928 | Contig5028 | actin |
| 929 | Contig5034 | actin-depolymerizing factor 2-like |
| 930 | Contig5035 | phosphatidylcholine transfer protein, putative |
| 931 | Contig5036 | bidirectional sugar transporter SWEET6b |
| 932 | Contig5037 | protein C2-DOMAIN ABA-RELATED 4 |
| 933 | Contig5040 | glucan endo-1,3-beta-glucosidase 13 |
| 934 | Contig5049 | nucleobase-ascorbate transporter 6 |
| 935 | Contig5050 | ubiquitin-conjugating enzyme E2 variant 1D |
| 936 | Contig5053 | ---NA--- |
| 937 | Contig5066 | ---NA--- |
| 938 | Contig5095 | chlorophyll a-b binding protein 8, chloroplastic |
| 939 | Contig5096 | cystathionine gamma-synthase 1, chloroplastic-like |
| 940 | Contig5102 | PHD finger protein Alfin1 |
| 941 | Contig5124 | shikimate O-hydroxycinnamoyltransferase-like |
| 942 | Contig5135 | homeobox-leucine zipper protein HDG5 |
| 943 | Contig5141 | ---NA--- |
| 944 | Contig5143 | AMME syndrome candidateprotein 1 protein, putative |
| 945 | Contig5175 | serine-threonine protein kinase, plant-type, putative |
| 946 | Contig5179 | ---NA--- |
| 947 | Contig5186 | oleosin 18.2 kDa-like |
| 948 | Contig5188 | 2S albumin-like |
| 949 | Contig5191 | probable acetyltransferase NATA1-like |

***Euphorbia tirucalli***

| **Sl No.** | **ID** | **Function** |
| --- | --- | --- |
| 1 | Contig11 | histone H2A |
| 2 | Contig17 | tryptophan--tRNA ligase, cytoplasmic |
| 3 | Contig21 | high mobility group B protein 3-like |
| 4 | Contig82 | glyceraldehyde-3-phosphate dehydrogenase, cytosolic-like |
| 5 | Contig130 | very-long-chain (3R)-3-hydroxyacyl-CoA dehydratase PASTICCINO 2 |
| 6 | Contig138 | glycine-rich RNA-binding protein-like |
| 7 | Contig155 | non-specific lipid-transfer protein-like protein At5g64080 |
| 8 | Contig162 | iron-sulfur cluster assembly protein 1-like |
| 9 | Contig179 | cytochrome b-c1 complex subunit 8-like |
| 10 | Contig189 | glucan endo-1,3-beta-glucosidase-like |
| 11 | Contig198 | ---NA--- |
| 12 | Contig215 | DNA-directed RNA polymerases II, IV and V subunit 6A-like |
| 13 | Contig225 | copper transport protein ATX1-like |
| 14 | Contig233 | peptidyl-prolyl cis-trans isomerase CYP20-1 |
| 15 | Contig235 | dnaJ protein homolog |
| 16 | Contig264 | universal stress protein A-like protein |
| 17 | Contig273 | stem-specific protein TSJT1 |
| 18 | Contig281 | thioredoxin H-type |
| 19 | Contig285 | tubulin alpha chain-like isoform X2 |
| 20 | Contig289 | NAC domain-containing protein 72 |
| 21 | Contig291 | protein EXORDIUM-like 2 |
| 22 | Contig313 | thaumatin-like protein |
| 23 | Contig320 | 21 kDa protein-like |
| 24 | Contig321 | auxin-repressed 12.5 kDa protein-like |
| 25 | Contig331 | CBL-interacting serine/threonine-protein kinase 6-like |
| 26 | Contig338 | acylpyruvase FAHD1, mitochondrial |
| 27 | Contig350 | asparagine synthetase [glutamine-hydrolyzing] 1 |
| 28 | Contig371 | heavy metal-associated isoprenylated plant protein 3-like |
| 29 | Contig395 | non-specific lipid-transfer protein 2-like |
| 30 | Contig405 | putative phosphatidylglycerol/phosphatidylinositol transfer protein DDB_G0282179 |
| 31 | Contig409 | 14 kDa proline-rich protein DC2.15-like |
| 32 | Contig411 | heat shock factor protein HSF24 |
| 33 | Contig418 | Asp_protease_2 domain-containing protein |
| 34 | Contig419 | polyubiquitin-like |
| 35 | Contig425 | ---NA--- |
| 36 | Contig433 | proteasome subunit alpha type-3 |
| 37 | Contig444 | pathogenesis-related protein PR-4-like |
| 38 | Contig445 | ubiquitin-conjugating enzyme E2 36 |
| 39 | Contig454 | berberine bridge enzyme-like 17 |
| 40 | Contig461 | 60S ribosomal protein L18a-like |
| 41 | Contig463 | 14-3-3-like protein |
| 42 | Contig465 | 60S ribosomal protein L13-1-like |
| 43 | Contig470 | putative methyltransferase DDB_G0268948 |
| 44 | Contig475 | GEM-like protein 4 |
| 45 | Contig480 | UMP-CMP kinase 3-like isoform X1 |
| 46 | Contig484 | lipid transfer-like protein VAS |
| 47 | Contig491 | 40S ribosomal protein S9-2-like |
| 48 | Contig493 | 40S ribosomal protein S24-1 |
| 49 | Contig495 | UPF0329 protein ECU05_1680/ECU11_0050 |
| 50 | Contig509 | 40S ribosomal protein SA-like |
| 51 | Contig511 | protein TORNADO 2 |
| 52 | Contig512 | fructose-bisphosphate aldolase 6, cytosolic |
| 53 | Contig514 | ---NA--- |
| 54 | Contig524 | 60S ribosomal protein L28-2-like |
| 55 | Contig529 | 40S ribosomal protein S13 |
| 56 | Contig550 | glutathione S-transferase-like |
| 57 | Contig565 | endochitinase PR4-like |
| 58 | Contig580 | E3 ubiquitin-protein ligase ATL4 |
| 59 | Contig582 | proteasome subunit beta type-3-A |
| 60 | Contig590 | RGG repeats nuclear RNA binding protein A-like |
| 61 | Contig593 | vesicle-associated membrane protein 721 |
| 62 | Contig594 | 40S ribosomal protein S3a-1 |
| 63 | Contig598 | actin-depolymerizing factor 2-like |
| 64 | Contig608 | ---NA--- |
| 65 | Contig609 | glycine-rich protein DC7.1-like |
| 66 | Contig613 | peroxidase 3-like |
| 67 | Contig621 | DUF674 domain-containing protein |
| 68 | Contig624 | cysteine proteinase inhibitor-like |
| 69 | Contig630 | ADP-ribosylation factor 2 |
| 70 | Contig633 | polyubiquitin 11 |
| 71 | Contig634 | polyubiquitin 3 |
| 72 | Contig636 | histone H1-like |
| 73 | Contig640 | universal stress protein PHOS32-like |
| 74 | Contig647 | inactive beta-amylase 9 |
| 75 | Contig656 | 40s ribosomal protein s6 |
| 76 | Contig658 | low-temperature-induced cysteine proteinase-like |
| 77 | Contig659 | 60S acidic ribosomal protein P2 |
| 78 | Contig660 | eukaryotic translation initiation factor 5A |
| 79 | Contig663 | inosine triphosphate pyrophosphatase |
| 80 | Contig669 | ---NA--- |
| 81 | Contig676 | 60S ribosomal protein L17-2 |
| 82 | Contig683 | histone H2B-like |
| 83 | Contig691 | late embryogenesis abundant protein Lea5 |
| 84 | Contig696 | stem-specific protein TSJT1-like |
| 85 | Contig704 | alpha-amylase isoform X2 |
| 86 | Contig710 | polyubiquitin isoform X1 |
| 87 | Contig721 | translation machinery-associated protein 7 |
| 88 | Contig727 | putative lipid-transfer protein DIR1 |
| 89 | Contig728 | Calmodulin |
| 90 | Contig730 | histone H3.3 |
| 91 | Contig734 | protein SPIRAL1-like 1 |
| 92 | Contig736 | ---NA--- |
| 93 | Contig737 | calmodulin-7-like isoform X2 |
| 94 | Contig738 | polyadenylate-binding protein 8-like |
| 95 | Contig741 | major allergen Pru ar 1-like |
| 96 | Contig746 | ---NA--- |
| 97 | Contig748 | ATP synthase subunit beta, mitochondrial |
| 98 | Contig751 | small EDRK-rich factor 2-like |
| 99 | Contig755 | elongation factor 1-alpha |
| 100 | Contig759 | elongation factor 1-alpha-like |
| 101 | Contig765 | major allergen Pru ar 1 |
| 102 | Contig766 | major allergen Pru ar 1 |
| 103 | Contig767 | major allergen Pru ar 1 |
| 104 | Contig768 | 40S ribosomal protein S27-2 |
| 105 | Contig794 | ubiquitin receptor RAD23c-like |
| 106 | Contig808 | ---NA--- |
| 107 | Contig838 | 60S ribosomal protein L35 |
| 108 | Contig865 | 17.3 kDa class I heat shock protein-like |
| 109 | Contig930 | bark storage protein A |
| 110 | Contig968 | 40S ribosomal protein S8-like |
| 111 | Contig969 | 40S ribosomal protein S8-like |
| 112 | Contig970 | probable calcium-binding protein CML36 |
| 113 | Contig972 | cytochrome b-c1 complex subunit Rieske-4, mitochondrial-like |
| 114 | Contig1046 | alcohol-forming fatty acyl-CoA reductase-like |
| 115 | Contig1052 | ---NA--- |
| 116 | Contig1082 | histone H3.2 |
| 117 | Contig1132 | histone H4 |

***Vernicia fordii***

| **Sl No.** | **ID** | **Function** |
| --- | --- | --- |
| 1 | Contig2 | 2S albumin |
| 2 | Contig4 | F-box protein CPR30 |
| 3 | Contig5 | F-box protein CPR30 |
| 4 | Contig9 | oleosin 18.2 kDa-like |
| 5 | Contig11 | oleosin 16.4 kDa-like |
| 6 | Contig21 | ninja-family protein AFP3-like |
| 7 | Contig63 | Ureidoglycolate hydrolases |
| 8 | Contig65 | metallothionein-like protein type 2 |
| 9 | Contig73 | uncharacterized protein LOC110668259 |
| 10 | Contig81 | major allergen Pru ar 1-like |
| 11 | Contig82 | 40S ribosomal protein S5-like |
| 12 | Contig84 | AMP-dependent synthetase/ligase |
| 13 | Contig85 | metallothionein-like protein type 2 |
| 14 | Contig91 | late embryogenesis abundant protein-like |
| 15 | Contig94 | ABC transporter G family member 8-like |
| 16 | Contig101 | auxin-repressed 12.5 kDa protein-like isoform X2 |
| 17 | Contig110 | protein DCL, chloroplastic |
| 18 | Contig111 | 11S globulin seed storage protein 2-like |
| 19 | Contig113 | major allergen Pru ar 1-like |
| 20 | Contig117 | ultraviolet-B receptor UVR8 |
| 21 | Contig124 | 1-Cys peroxiredoxin |
| 22 | Contig125 | membrane steroid-binding protein 2-like |
| 23 | Contig131 | peptidyl-prolyl cis-trans isomerase |
| 24 | Contig161 | protein BUD31 homolog 2 |
| 25 | Contig171 | 60S ribosomal protein L10 |
| 26 | Contig172 | 2S albumin |
| 27 | Contig173 | bark storage protein A |
| 28 | Contig174 | 11S globulin seed storage protein 2-like |
| 29 | Contig176 | 11S globulin seed storage protein 2-like |
| 30 | Contig179 | legumin B |
| 31 | Contig180 | thioredoxin-like 3-2, chloroplastic isoform X1 |
| 32 | Contig182 | major allergen Pru ar 1-like |
| 33 | Contig183 | legumin A |
| 34 | Contig184 | vicilin-like antimicrobial peptides 2-2 |
| 35 | Contig185 | ADP-ribosylation factor 1 |
| 36 | Contig186 | uncharacterized protein LOC110646458 isoform X2 |
| 37 | Contig187 | casein kinase II subunit alpha-2 |
| 38 | Contig189 | probable aquaporin TIP3-2 |
| 39 | Contig190 | dof zinc finger protein DOF4.6-like |
| 40 | Contig193 | late embryogenesis abundant protein Lea5-D |
| 41 | Contig196 | probable protein disulfide-isomerase A6 |
| 42 | Contig197 | 18.1 kDa class I heat shock protein-like |
| 43 | Contig198 | probable polygalacturonase |
| 44 | Contig200 | cell number regulator 8-like |
| 45 | Contig202 | bark storage protein A-like |
