# Supplementary material for: In Silico Mining and Characterization of High-Quality SNP/Indels in Some Agro-Economically Important Species Belonging to the Family Euphorbiaceae
Source: Genes (Basel). 2023 Jan 27;14(2):332. doi: 10.3390/genes14020332 (PMC9956114; doi:10.3390/genes14020332)
Supplement: Supplementary file 1 [file genes-14-00332-s001.zip › genes-2109399-Supplementary Tables.docx]

**Table S1.** Distribution of high quality exonic single nucletide polymorphisms (SNPs).

| **Species** | **Transitions (ts)** | **Transversions (tv)** | **InDels** | **TOTAL** |
| --- | --- | --- | --- | --- |
|  |  |  |  |  |
| ***Euphorbia esula*** | **1393** | **905** | **189** | 2487 |
| ***Euphorbia fischeriana*** | 15 | 4 | 5 | 24 |
| ***Euphorbia lagascae*** | 0 | 0 | 4 | 4 |
| ***Euphorbia tirucalli*** | 60 | 36 | 21 | 117 |
| ***Hevea brasiliensis*** | 495 | 302 | 51 | 848 |
| ***Jatropha curcas*** | 430 | 221 | 50 | 701 |
| ***Manihot esculenta*** | 1619 | 1301 | 230 | 3150 |
| ***Ricinus communis*** | 495 | 427 | 535 | 1457 |
| ***Vernicia fordii*** | 28 | 27 | 6 | 61 |

**Table S2.** Distribution of high quality intronic single nucletide polymorphisms (SNPs).

| **Species** | **Transitions (ts)** | **Transversions (tv)** | **InDels** | **TOTAL** |
| --- | --- | --- | --- | --- |
|  |  |  |  |  |
| ***Euphorbia esula*** | 1393 | 905 | 189 | 2487 |
| ***Euphorbia fischeriana*** | 15 | 4 | 5 | 24 |
| ***Euphorbia lagascae*** | 0 | 0 | 4 | 4 |
| ***Euphorbia tirucalli*** | 60 | 36 | 21 | 117 |
| ***Hevea brasiliensis*** | 495 | 302 | 51 | 848 |
| ***Jatropha curcas*** | 430 | 221 | 50 | 701 |
| ***Manihot esculenta*** | 1619 | 1301 | 230 | 3150 |
| ***Ricinus communis*** | 495 | 427 | 535 | 1457 |
| ***Vernicia fordii*** | 28 | 27 | 6 | 61 |

**Table S3.** Distribution of nucleotide substitutions in nine selected species belonging to the family Euphorbiaceae.

| **Species** | **[A/G]** | | **[C/T]** | **[A/C]** | **[A/T]** | **[G/C]** | **[G/T]** | **[A]** | **[C]** | **[G]** | **[T]** |
| --- | --- | --- | --- | --- | --- | --- | --- | --- | --- | --- | --- |
| ***Euphorbia esula*** | 758 | 970 | | 281 | 353 | 228 | 317 | 117 | 70 | 95 | 81 |
| ***Euphorbia fischeriana*** | 7 | 11 | | 0 | 2 | 4 | 1 | 2 | 1 | 2 | 1 |
| ***Euphorbia lagascae*** | 0 | 0 | | 0 | 0 | 0 | 0 | 3 | 2 | 1 | 1 |
| ***Euphorbia tirucalli*** | 40 | 37 | | 20 | 22 | 10 | 16 | 13 | 2 | 11 | 5 |
| ***Hevea brasiliensis*** | 394 | 439 | | 155 | 172 | 154 | 157 | 54 | 20 | 30 | 60 |
| ***Jatropha curcas*** | 364 | 228 | | 64 | 96 | 72 | 94 | 47 | 12 | 27 | 39 |
| ***Manihot esculenta*** | 1193 | 1286 | | 527 | 660 | 503 | 546 | 240 | 134 | 146 | 240 |
| ***Ricinus communis*** | 367 | 476 | | 218 | 301 | 141 | 267 | 215 | 176 | 232 | 181 |
| ***Vernicia fordii*** | 34 | 12 | | 13 | 25 | 14 | 26 | 5 | 2 | 4 | 5 |
